# Unraveling daily dynamics of supraglacial lakes in response to hydrological pulses and anomalous climate signals

**DOI:** 10.1038/s41467-026-75669-3

**Published:** 2026-07-17

**Authors:** Jian Song, Haijun Qiu, Adam Emmer, Guoqing Zhang, Ninglian Wang, Ya Liu, Guoqing Yang, Yiwen Liang

**Affiliations:** 1 Shaanxi Provincial Key Laboratory of Earth Surface System and Environmental Carrying Capacity, College of Urban and Environmental Sciences, Northwest University, Xi’an, China; 2 Institute of Earth Surface System and Hazards, College of Urban and Environmental Sciences, Northwest University, Xi’an, China; 3 Department of Physical Geography and Geoecology, Faculty of Science, Charles University, Prague, Czechia; 4 Key Laboratory of Biodiversity and Environment on the Qinghai-Tibetan Plateau, Ministry of Education, School of Ecology and Environment, Xizang University, Lhasa, China; 5 State Key Laboratory of Tibetan Plateau Earth System, Environment and Resources (TPESER), Institute of Tibetan Plateau Research, Chinese Academy of Sciences, Beijing, China

**Keywords:** Natural hazards, Cryospheric science

## Abstract

Mass loss from debris-covered glaciers in High Mountain Asia drives the expansion of small supraglacial lakes (SGLs) and changes the occurrence patterns of hazardous processes including glacial lake outburst floods. Here, we analyse surface area changes of small SGLs ( > 0.045×10^4^ m^2^) in the Sagarmatha region, Eastern Himalaya, integrating data with daily resolution from 2016 to 2024. The results show SGLs expanded at a mean rate of 5.90 ± 1.49×10^4^ m^2^·yr⁻¹. Starting from October 2022, SGLs in the study area have exhibited anomalous expansion, with the total lake area reached its maximum during this period, 67.35% higher than during the second peak (2020). The seasonal fluctuations of SGLs are synchronous with short-term variations in global temperature. By mid-century, SGL expansion in High Mountain Asia is expected to continue. Given their rapid response to climate change, small SGLs could support the development of early-warning frameworks for hydrological pulses and extreme climate events.

## Abstract (web summary)

This study shows that small supraglacial lakes not only reflect the increasingly frequent hazards associated with small-lake outburst events, but also indicate that their seasonal dynamics serve as a precursor signal of global warming.

## Introduction

Glaciers are an integral component of the Earth’s climate and hydrological systems, governed by atmospheric and oceanic variations, and represent one of the most sensitive and early-warning indicators of climate warming^1–4^. Driven by global warming, global glacier mass has declined substantially, between 2000 and 2023, the annual loss of glaciers reached 273 ± 16 gigatonnes, providing direct and unequivocal evidence of global climate change^5–7^. Against this backdrop, cascading hazards arising from the ongoing collapses or detachments and glacial lake outburst floods (GLOFs) have attracted considerable attention in global climate change research^1,8–11^. Seasonal fluctuations of glaciers and lakes exert persistent impacts on downstream runoff, landslides, and sediment deposition, disrupting transportation and communication networks, and damaging infrastructure and farmland^12–14^. Although the seasonality of glacial lakes is a key characteristic of these hazards, the extent and temporal trends of their impacts remain poorly understood in most mountainous regions. In recent years, advances in optical and radar remote sensing have accelerated investigations into seasonal to interannual variations in glacier flow and glacial lake dynamics. A global study shows that basal hydrological systems not only modulate seasonal glacier flow but also that seasonal variations are weakly yet significantly correlated with interannual variations^15^, providing critical insights into seasonal anomalies in glaciers and glacial lakes and their associated extreme hazards under global warming.

It is estimated that around 1.4 billion people in the High Mountain Asia (HMA) regions rely on freshwater derived from snow and ice melt for their livelihoods^16–18^. Moreover, glaciers in these regions are highly sensitive to climate change and monsoon variations, making them among the most climate-vulnerable areas^19,20^. Currently, glaciers on the HMA are primarily influenced by two major circulation systems. The summer Indian monsoon and the winter mid-latitude westerlies, together with the region’s complex topography, control the distribution and heterogeneity of glaciers^21^. As Asia’s “water tower,” the HMA also exhibits increasing imbalance in its cryosphere–hydrosphere–atmosphere (solid–liquid–gas) water storage. This imbalance is expressed as water gains (lake expansion) in endorheic basins and water losses (lake shrinkage) in exorheic basins or in endorheic basins influenced by climate, glaciers, or human activities, forming a pronounced north–south dipole pattern^20^. With the intensification of this imbalance, the dynamic equilibrium between glaciers and lakes, driven by the interaction between the mid-latitude westerlies and the Indian summer monsoon, has been disrupted. Since 1990, the number, area, and volume of global glacial lakes have increased by 53%, 51%, and 48%, respectively. However, this parallel pattern of accelerated glacier mass loss and lake expansion remains unsustainable^1,22,23^.

In most regions of HMA, glaciers experience dynamic changes in regional hydrology and surface erosion during terminus retreat. The resulting glacial depressions and debris create an ice-rich environment that promotes the formation and expansion of glacial lakes and supraglacial ponds^24,25^. Debris-covered glaciers with ponds often transport meltwater downstream through supraglacial drainage and englacial drainage pathways, which further enhances ice ablation and subglacial melting, causing additional glacier mass loss and retreat, and thereby driving further lake expansion and merging of small supraglacial lakes (SGLs) into larger proglacial lakes^18,26^. This dynamic hydrological connectivity is frequently modulated by pronounced seasonal variability in SGLs, as evidenced by their marked seasonal fluctuations in area and depth on HMA glaciers, which typically reach their maximum extent during the mid-monsoon period before subsequently declining^27^. During this phase, debris redistribution and evolving surface topography drive the migration and dynamic reorganization of SGLs. When these water bodies approach local hydrological thresholds, rapid drainage may occur. Concentrated discharge is particularly likely where multiple small SGLs are hydraulically connected through supraglacial drainage networks and collectively feed into a larger proglacial lake, increasing the likelihood of catastrophic GLOFs^18,26^. A typical example by Miles^26^, based on Planet imagery, documents an SGL outburst on the Khumbu Glacier. Multiple small SGLs rapidly coalesced prior to sudden drainage, reshaping the glacier surface and triggering debris-flow erosion and landslides within the glacier hydrological system, forming a cascading hazard chain. This process occurs over very short timescales. However, limitations in satellite revisit frequency and spatial resolution mean that uncertainties associated with such small SGL drainage and related debris-flow hazards are often overlooked^28^. In HMA, 13–36% of glacierized areas are covered by surface debris and feature SGLs, storing water equivalent to roughly 23% of the average daily observed runoff^18^. Frequent hydrological activity and lake evolution are key features of SGLs systems under environmental forcing. During these processes, debris flows and SGL outbursts driven by hydrological pulses are often difficult to capture using satellite observations. Consequently, the frequency of GLOFs is likely to have been substantially underestimated^18,29,30^.

**Fig. 1:**
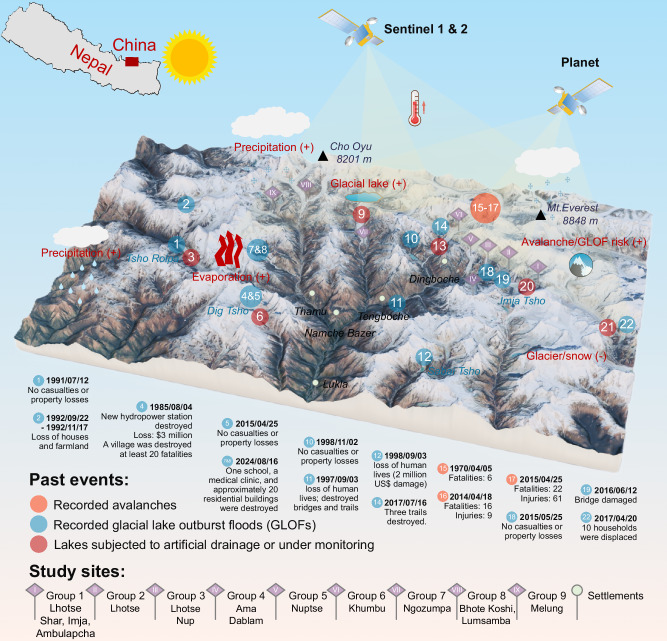
Diagram illustrating lake evolution and geological hazards in Sagarmatha National Park. The red labels and text represent the climate-related risks facing this region, namely that under global warming, increased snowfall/rainfall and the melting of glaciers and snowpack lead to the expansion of lake boundaries and heightened risks of GLOFs (Glacial Lake Outburst Floods) and avalanches. The numbers in the figure indicate the locations and associated hazard losses of past GLOFs, avalanches, and manually monitored lakes (Data source: Suppl. Table 1). Glaciers sharing the same drainage outlet were grouped together and labeled as Groups 1–9. The glaciers included in each group are listed as follows: Group 1, Lhotse Shar, Imja, and Ambulapcha; Group 2, Lhotse; Group 3, Lhotse Nup; Group 4, Ama Dablam; Group 5, Nuptse; Group 6, Khumbu; Group 7, Ngozumpa; Group 8, Bhote Koshi and Lumsamba; Group 9, Melung Glaciers. Supplementary Fig. 1 serves as a supplement to this figure, documenting the spatial information of clean glaciers, debris-covered glaciers, and runoff. Satellite, lake and avalanche graphics by Tracey Saxby (Integration and Application Network, ian.umces.edu/media-library).

In Sagarmatha National Park, the ablation zones of glaciers are almost entirely covered by continuous debris, which plays a key role in modulating glacier responses to climatic forcing^18,31^. During the retreat of debris-covered glaciers, debris can either accelerate or retard ice melt depending on its thickness; together with the dynamic glacier surface topography, this promotes enhanced differential ablation and the formation of SGLs, thereby increasing the risk of mountain hazards^18^. Over the past few decades, more than 16 GLOFs and ice avalanches have been reported in the region, resulting in over 340 deaths and missing persons^9,32^. For example, the 1985 Dig Tsho GLOF alone destroyed 14 bridges and the nearly completed Namche hydropower station. To mitigate the potential outburst risk of large moraine-dammed lakes, long-term monitoring and engineering interventions have been implemented, successfully preventing lake outburst events to some extent^33,34^. However, small SGLs, owing to their rapid evolution and unpredictable seasonal variability, are increasingly becoming a major source of GLOFs; since 2000, five of the seven recorded GLOF events in the region have originated from SGLs^9^. Such frequent and hard-to-monitor geohazards are rarely included in global compilations of glacial lake outburst events (Fig. 1). As critical indicators of global climate change, seasonal fluctuations in lake area are considered one of the most sensitive characteristics of small SGLs. Some case studies show that, prior to reaching critical outburst thresholds, some SGLs can expand rapidly over short timescales, with surface area increases exceeding eightfold, and reaching up to 70-fold over a complete seasonal expansion cycle^35^. However, due to the trade-off between spatial and temporal resolution in single-sensor satellite data, such short-term fluctuations are often overlooked^29^. Therefore, urgent investigations into the seasonal dynamics of small SGLs and their implications for mountain hazard management are needed^36^.

Here, we aim to capture nearly daily-resolution fluctuations and trends in SGL surface areas in Sagarmatha National Park from 2016 to 2024, and explore its link to glacier velocity, surface deformations and sea surface temperatures (SST). We incorporate long-term trends, differences across monsoon phases, intra-group differences at the same phase in different years, and monthly lake area standard deviations and standard errors to assess anomalous lake events, i.e., lake area variations that deviate significantly from their typical seasonal patterns. Basnet found that the formation of proglacial lakes accelerates glacier mass loss and promotes glacier retreat, thereby affecting glacier velocity^37^. Therefore, glacier velocity is incorporated into the analytical framework of SGL expansion. Cumulative surface deformation at terminus regions is used to detect potential hazards associated with cryospheric mass movement, such as avalanches, debris flows, or GLOFs. SST anomalies and moisture flux were employed to quantify the early-warning signals of small SGL expansion in response to global climate change.

## Results

### Dynamics and lifecycle of supraglacial lakes

**Fig. 2:**
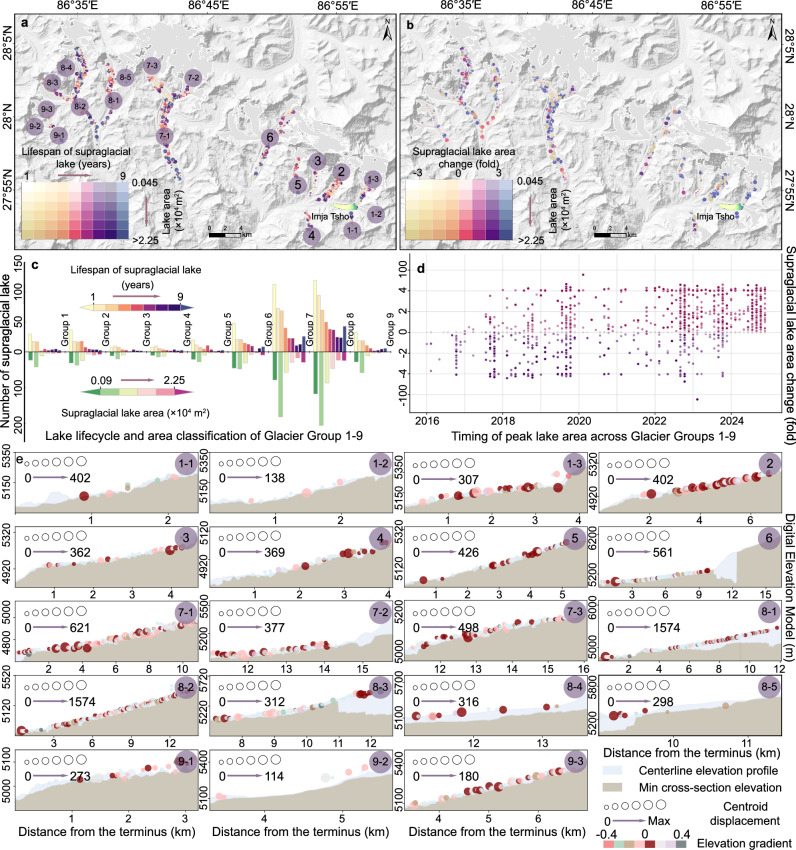
Spatial distribution, temporal dynamics, and lifecycle characteristics of supraglacial lakes between 2016 and 2024. **a** Spatial patterns and lifecycles of supraglacial lakes (SGLs) of different sizes (2016–2024), due to the branching structure of some glaciers, each branch was assigned a unique identifier to facilitate analysis of the spatiotemporal distribution of lakes (Suppl. Figs. 9–12). The correspondence between glacier systems, glacier names, glacier branches, and the number of lakes in each group is provided in Suppl. Table 2. **b** Spatial patterns of lake peaks and peak-to-trough change rates. In panels (**a**, **b**), circle size represents lake area, which is classified into six levels: of 0.045 × 10^4^, 0.09 × 10^4^, 0.36 × 10^4^, 0.81 × 10^4^, 1.14 × 10^4^, 2.25 × 10^4^, and greater than 2.25 × 10^4^ m^2^, respectively. **c** Lifecycle, number, and size distribution characteristics of lakes in each glacier group. **d** Timing of peak SGL area for expanding and shrinking lakes across nine glacier groups. **e** Lake centroids’ migration distances and elevation gradients, with panel e labels matching glacier numbers in panel a. In panel (**e**), circle size indicates lake migration distance, divided into six levels using equal intervals. Imja Tsho, a glacial lake in the Everest region of Nepal, has been recognized as a potentially hazardous glacial lake. To reduce the risk of its outburst, the Nepalese government, together with the United Nations Development Programme (UNDP) and the Nepal Army, constructed a drainage channel from June to September 2016. Over a cumulative period of two months, approximately 3.4 m of water was lowered, discharging about 4–5 million cubic meters.

**Fig. 3:**
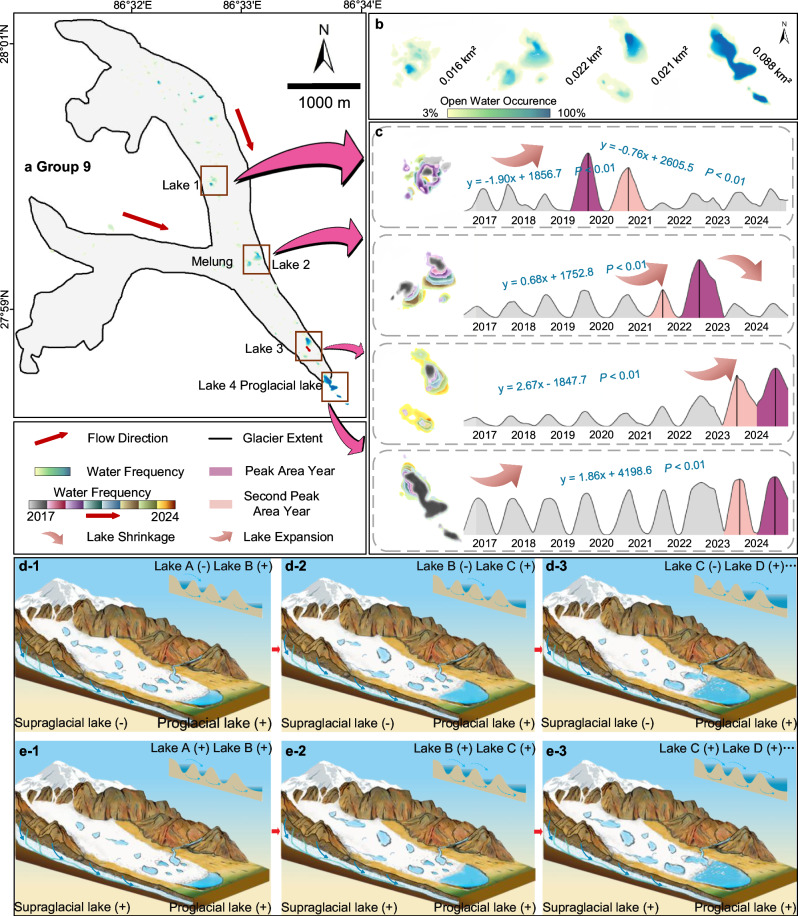
Hydrological connectivity and cascading drainage among supraglacial lakes. **a** Frequency of lake occurrence in Glacier Group 9 from 2016 to 2024, with the four largest lakes highlighted (the four largest lakes). **b** Lakes are arranged from left to right corresponding to Lakes 1–4 in (**a**), with the maximum area and occurrence frequency shown for each lake over the study period. **c** Annual frequency of supraglacial lakes (SGLs) and their cascading drainage processes, with locally weighted scatterplot smoothing fitted curves for lakes of interest. The x-axis of the ridgeline plot represents the time series of SGL area, with filled areas indicating the first and second peaks of lake area. The frequency plots on the left use different gradient colors to show the annual distribution of lakes, illustrating upstream lake incision and downstream proglacial lake aggradation. **d** A pattern of downstream lake expansion accompanied by upstream lake decline. **e** A pattern of downstream lake expansion without upstream lake decline. Arrow graphics by Alexandra Fries (Integration and Application Network, ian.umces.edu/media-library).

From 2016 to 2024, we detected 1493 SGLs ( > 0.045 × 10^4^ m^2^) across nine glacier groups, with 42–502 lakes per group (Fig. 2), the largest covering 196 × 10^4^ m^2^. Only 98 lakes were perennial during the study period (2016–2024), 40 of which exceeded 2.25 × 10^4^ m^2^. The recurrence of ephemeral lakes closely correlated with their size: 928 lakes (62.16%) persisted 1–3 years, of which 779 (83.94%) were smaller than 0.36 × 10^4^ km^2^ (Fig. 2c). The area of SGLs shows pronounced fluctuations, with peak-to-trough variability reaching up to fourfold, and in some lakes even several tens of times. (Fig. 2d). At the individual lake level, we observed that peak lake areas of expanding lakes were primarily concentrated during 2022–2024, whereas those of shrinking lakes were mainly concentrated during 2017–2019 (Fig. 2d). We examined the spatial dynamics of these SGLs (Fig. 2e), using the displacement and slope of lake centroids to quantify lake-related hazards. At the terminus of Glacier Group 8, the cumulative displacement of terminus lakes reached 1574 m, resulting from the merger of two terminus lakes, while the maximum displacement of lakes at other glaciers ranged between 114 and 621 m. Lake expansion also depends on the relatively gentle slopes of glacier termini and the coalescence of small SGLs. For instance, Imja Tsho, a large proglacial lake that developed from five supraglacial ponds in the 1950s^38^, primarily expanded within ~2 km up-glacier of the glacier terminus. Since then, the lake has continued to expand up-glacier, currently reaching a length of approximately ~3 km. During the initial 20 years, Imja Tsho evolved from multiple small supraglacial ponds into a single proglacial lake, and subsequently expanded from ~0.3 km^2^ to ~1.96 km^2^ by 2024 over the following six decades. We further assessed whether other glacier–lake systems follow a similar evolutionary trajectory to Imja Tsho and found that Glacier Groups 2, 5 and 7–9 have progressed beyond the early stage of proglacial lake formation, characterized by a transition from short-lived, spatially dispersed SGLs to a persistent and relatively stable, continuous lake at the glacier terminus (Fig. 3 and Suppl. Figs. 2–5). In contrast, Glacier Groups 3 and 4 show no clear tendency toward proglacial lake formation, as their termini lack the capacity to accumulate meltwater, and water bodies persist only as short-lived SGLs (Suppl. Figs. 6, 7). By comparison, Glacier Group 6 represents a distinct hydrological end-member, characterized by widespread SGLs across the glacier surface but lacking any evidence of convergence or accumulation at the glacier terminus (Suppl. Fig. 8). Therefore, in assessing the risk of GLOFs, continuous monitoring of the expansion of individual lakes is required, while particular attention should also be paid to the potential for abrupt coalescence and concentrated drainage of small SGLs through existing supraglacial drainage networks during the monsoon season.

### Seasonal anomaly in supraglacial lake

**Fig. 4:**
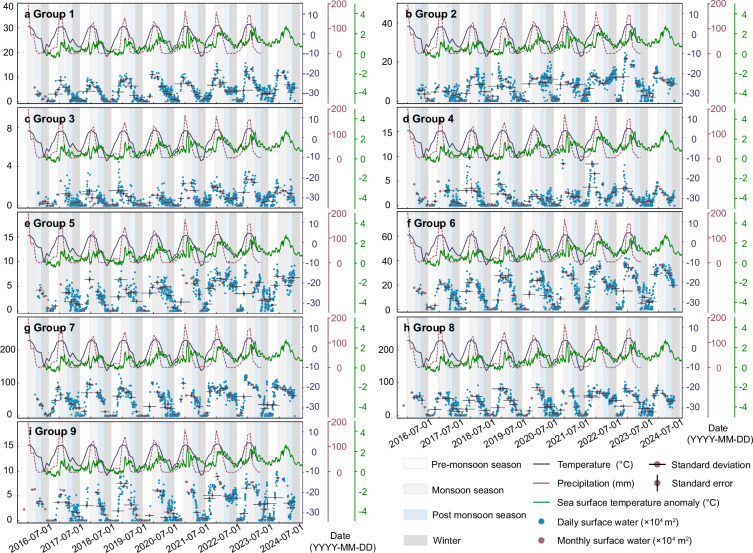
Highlight the time series of surface water resources, station temperature, precipitation, and Pacific Ocean sea surface temperature anomalies. Time series are shown for (**a**) Group 1, Lhotse Shar、Imja and Ambulapcha; (**b**) Group 2, Lhotse; (**c**) Group 3, Lhotse Nup; **d** Group 4, Ama Dablam; (**e**) Group 5, Nuptse; (**f**) Group 6, Khumbu; (**g**) Group 7, Ngozumpa; (**h**) Group 8, Bhote Koshi and Lumsamba and (**i**) Group 9, Melung Glaciers. Highlight supraglacial lakes (SGLs) in (**a**–**f**) were selected based on their stronger correlations with sea surface temperature (SST) anomalies when leading by 1–2 months (correlation coefficients of 0.72, 0.65, 0.64, 0.41, 0.68, 0.71, 0.63, 0.58, and 0.62, respectively). Pre-monsoon season (Mar 1–Jun 15), Monsoon season (Jun 15–Sep 30), Post-monsoon season (Oct 1–Nov 30), winter (Dec 1–Feb 28). The horizontal error bars in the scatter plot represent the normalized standard deviation, while the vertical error bars represent the standard error. They are used to measure the variability of monthly fluctuations in SGL area, and to examine day-scale differences of SGLs across different years in the long-term time series.

We observed the temporal variations of nine groups of SGLs in Sagarmatha National Park from 2016 to 2024 (Fig. 4). Our surface water time series indicate that the interannual rate of change variability of SGLs was 5.90 ± 1.49 × 10^4^ m^2^·yr^−1^ across the nine glacier groups studied. The two highest mean annual growth rates were observed at SGLs in Glacier 7 and 6, reaching 1.44 ± 0.99 × 10^4^ m^2^·yr^−1^ (Fig. 4g) and 0.654 ± 0.317 × 10^4^ m^2^·yr^−1^ (Fig. 4f), respectively. Among the other observed SGLs, all exhibited accelerating expansion trends except Glacier Group 8, which showed a mean annual decline of −0.531 ± 1.33 × 10^4^ m^2^·yr^−1^ (Fig. 4h). Overall trends for the remaining glaciers were as follows: Glacier Group 1, 0.336 ± 0.063 × 10^4^ m^2^·yr^−1^ (Fig. 4a); Glacier Group 2, 0.501 ± 0.159 × 10^4^ m^2^·yr^−1^ (Fig. 4b); Glacier Group 3, 0.046 ± 0.015 × 10^4^ m^2^·yr^−1^ (Fig. 4c); Glacier Group 4, 0.025 ± 0.043×10^4^ m^2^·yr^−1^ (Fig. 4d); Glacier Group 5, 0.208 ± 0.094 × 10^4^ m^2^·yr^−1^ (Fig. 4e); and Group 9, 0.0059 ± 0.0121 × 10^4^ m^2^·yr^−1^ (Fig. 4i). During the study period, the annual rates of increase and decrease of the total area of SGLs on the nine glacier groups exhibited heterogeneity. For example, in 2017, the total area of Glacier Group 1 expanded, while the remaining eight glacier groups showed decreasing trends. Only in 2022 did the total lake area on all nine glacier groups exhibit increasing trend (Suppl. Table 3). Using normalized standard deviation and standard error to measure the monthly dispersion of SGLs, fluctuations during the post-monsoon and winter phases of the nine glacier groups in 2022–2023 remained at relatively low levels. For example, in Glacier Group 6 from October to December, the normalized standard deviations and standard errors were lower than in other years (Fig. 4a–i). The time series of lake area changes, together with on-site meteorological observations, indicate that SGLs in the Sagarmatha National Park exhibit typical seasonal variations, with lake areas reaching their annual maximum during the summer monsoon, followed by continuous shrinkage in the post-monsoon and winter months. Overall, the lake area shows an increasing long-term trend and exhibits a lagged correlation with SST anomalies.

### Drainage events from supraglacial lakes

We used complete Planet imagery from 2016 to 2024 to capture all downstream drainage events of the nine glacier groups by tracking intra-annual dynamics of lake extent, together with full Sentinel-1 data to measure surface deformation at glacier termini. Multiple lakes on the same glacier were observed to exhibit interconnected behaviors, including pre-monsoon lake filling, rapid drainage during the monsoon, and slow drainage in winter (Fig. 3). Among the four largest lakes in Glacier Group 9 (1.6 × 10^4^ m^2^, 2.2 × 10^4^ m^2^, 2.1 × 10^4^ m^2^, and 8.8 × 10^4^ m^2^), we recorded a stepped downstream drainage sequence of upstream lakes from 2019 to 2024. Lake 1 reached its peak capacity on 4 September 2020, followed by rapid drainage in October 2020. During the drainage of Lake 1, Lake 2 began storing water in August 2021, reaching its peak in August 2022 before undergoing drainage. Concurrently with Lake 2’s drainage, Lake 3 exhibited a similar storage–drainage cycle, attaining storage peaks in August 2023 and August 2024, while the proglacial lake displayed a continuous storage pattern. As a glacier experiencing significant retreat and relatively slow ice flow, all lakes showed a persistent decline in volume following the completion of drainage events (Suppl. Figs. 2, 3 and 6–8). In contrast to Glacier Group 9, retreating glaciers with relatively fast ice flow, such as Glacier Group 6, exhibited statistically significant persistent water storage following drainage events, as indicated by least squares linear regression analysis (*P* < 0.01) (Suppl. Figs. 4, 5 and 13). For glaciers with proglacial lakes, the lake area in terminus regions showed a significant increasing trend (*P* < 0.01). Simultaneously, we observed two main development patterns of SGL drainage over multiple years: one in which upstream drainage is accompanied by the expansion of downstream lakes and proglacial lakes (Fig. 3d), and another in which upstream lake drainage occurs simultaneously with the synchronous expansion of the original lake, downstream lakes, and proglacial lakes (Fig. 3e).

### Inter-seasonal change of ice velocity

Snow and ice ablation and precipitation expanded the extent of surface water, while increasing meltwater infiltrated the glacier bed, reduced basal friction and accelerated ice flow seasonally^39,40^. We obtained surface velocity variations for nine glacier groups by extracting ice surface velocity along their centerlines. Extensive evidence indicates that seasonal variations in SGLs and outburst floods are closely linked to glacier flow (Suppl. Figs. 14d, 15)^41–43^. Using the interquartile range (IQR) of ice velocity to quantify the amplitude of variations, we observed substantial heterogeneity in the temporal and magnitude characteristics of the nine glacier groups. For instance, the mean interannual velocity variability of Glacier Group 6 was 13.0 ± 23.5 m·yr^−1^, with an annual mean velocity of 25.3 m·yr^−1^, whereas Glacier Group 4 exhibited only 3.0 ± 2.6 m·yr^−1^ variability and a mean annual velocity of 3.6 m·yr^−1^. For glaciers that experienced SGL outburst events between 2016 and 2024, the mean velocities for Glacier Group 1-1, 1-2, and 1-3, as well as Groups 2 and 6, were 8.5, 32.3, 11.6, 17.1, and 25.3 m·yr^−1^, respectively, whereas Glacier Group 3, 4, 5, 7, 8, and 9, which have not experienced SGL outburst events, exhibited mean velocities of 4.2, 3.6, 3.9, 19.6, 8.2, and 3.9 m·yr^−1^ (Suppl. Table 4).

Increased meltwater input accelerated glacier flow. Along the 5–6 km section of Glacier Group 5, the mean flow velocity reached 0.685 m day^−1^ between May and October 2016, compared with only 0.06 m day⁻¹ in winter and 0.11 m day^−1^ prior to the monsoon (Suppl. Fig. 15g). In Glacier Group 9, we detected pronounced flow accelerations during March–May 2017 (0.19 m day^−1^) and April–May 2019 (0.18 m day^−1^). In comparison, the velocities were 0.04 m day^−1^ from May to October 2017, 0.10 m day^−1^ from November 2017 to January 2018, and 0.06 m day^−1^ from January to March 2018 (Suppl. Fig. 15p). Notably, during 2019 the area of SGLs reached a relative peak compared with other years (Fig. 4i). Based on the seasonal fluctuations of glacier velocity, glacier flow accelerations far exceeding the contemporaneous levels occurred during the 2015 and 2016 SGL outburst events along Glacier Group 1, as well as the 5–7 km segment of Glacier Group 2 (Suppl. Fig. 15a–d). For Glacier Group 6, which experienced a lake outburst on 16 June 2017, we observed glacier flow accelerations far exceeding the contemporaneous baseline. Glacier flow exhibits pronounced seasonality, with higher velocities during the pre-monsoon period. Additionally, in 2022, we captured anomalous velocities at 1–3 km on Glacier Group 2 near the terminus, coinciding with the timing of new lake formation. (Suppl. Fig. 14d).

### Surface deformations

Multi-year observations of terminus lake regions across the nine glacier groups revealed varying degrees of surface deformations in areas with proglacial lakes; for instance, the average cumulative displacement in the terminus regions of Glacier Groups 8 and 9 along the radar line-of-sight (LOS) exceeded −40 cm (motion away from the satellite). Glaciers without proglacial lakes exhibited minor subsidence or deposition of terminal moraine debris (Suppl. Figs. 16D, E and 17D, E). Specifically, the feature-tracking displacement maps around Imja Tsho reveal that the mean cumulative pixel displacement in the LOS direction is −17.59 cm (Suppl. Figs. 16A and 17A), higher displacement magnitudes are observed near the lake margin. The largest mean cumulative displacement occurs at the proglacial lake margin of Glacier Group 9, reaching −46.84 cm (Suppl. Fig. 16J). For glaciers without proglacial lakes, the terminal zones of Glacier Groups 3 and 4 exhibit mean cumulative displacements of 2.73 cm (motion toward the satellite) (Suppl. Figs. 16D and 17D) and −2.38 cm (Suppl. Figs. 16E and 17E), respectively, which are substantially lower than those of glaciers with proglacial lakes, including Glacier Groups 5 ( − 25.81 cm) (Suppl. Figs. 16F and 17F), Groups 7 ( − 31.70 cm) (Suppl. Figs. 16H and 17H), Groups 8-I and 8-J ( − 27.48 and −48.19 cm) (Suppl. Figs. 16I, J and 17I, J), and Groups 9-K and Groups 9-L ( − 20.11 and −46.84 cm) (Suppl. Figs. 16K, L and 17K, L). Although Glacier Group 6 hosts numerous SGLs, in the absence of a large proglacial lake, its mean displacement ( − 12.89 cm) remains lower than that of other glaciers with proglacial lakes (Suppl. Figs. 16G and 17G). Moreover, localized asymmetric erosion features are evident. For example, in Glacier Group 2, higher displacements are observed along the left side of the lake margin, reaching −42.22 cm (Suppl. Figs. 16B and 17B), whereas the right side shows only 12.83 cm (Suppl. Figs. 16C and 17C). A similar pattern is observed in Glacier Group 5, where the lower side of the lake margin exhibits greater displacement than the upper side.

### Link between supraglacial lake dynamics and SST anomalies

Between 2016 and 2024, we observed multi-year stable seasonal fluctuations in lakes, along with anomalous deviations from previous patterns during 2022–2023. During the post-monsoon season (Oct 1–Nov 30) and winter (Dec 1–Feb 28) of 2022–2023, the daily mean lake area reached 181.1 × 10^4^ m^2^, the highest value observed, with the second-highest being 108.24 × 10^4^ m^2^ in 2020–2021, and the minimum occurring in 2019–2020, with a daily mean of only 73.06 × 10^4^ m^2^. For the SGL in the Glacier Group 7, the daily mean area during 2022–2023 was 61.07 × 10^4^ m^2^, whereas the historical maximum in other years was only 37.56×10^4^ m^2^, a difference of 62.60%. To highlight the anomalous expansions in 2022–2023, we report, for each SGL on a glacier, the maximum lake area observed during 2022–2023, alongside the maximum area recorded in all other years (i.e., the second-largest recorded extent). Corresponding values were as follows: Glacier Group 1, 4.24 × 10^4^ m^2^ vs. 4.25 × 10^4^ m^2^ (0.23%); Group 2, 11.72 × 10^4^ m^2^ vs. 5.59 × 10^4^ m^2^ (109.66%); Group 3, 7.11 × 10^4^ m^2^ vs. 6.07 × 10^4^ m^2^ (17.13%); Group 4, 20.62 × 10^4^ m^2^ vs. 12.69 × 10^4^ m^2^ (62.49%); Group 5, 57.62 × 10^4^ m^2^ vs. 34.52 × 10^4^ m^2^ (66.93%); Group 6, 18.87 × 10^4^ m^2^ vs. 14.48 × 10^4^ m^2^ (28.57%); Group 8, 53.84 × 10^4^ m^2^ vs. 40.58 × 10^4^ m^2^ (32.68%); and Group 9, 4.72 × 10^4^ m^2^ vs. 2.71 × 10^4^ m^2^ (74.28%). Notably, only Glacier Groups 2, 3, and 4 exhibited an increasing trend in lake area during 2022–2023, while all other lakes showed a decline over time, and the rate of decline for all SGLs in 2022–2023 was lower than in previous years (Suppl. Figs. 18 and 19).

**Fig. 5:**
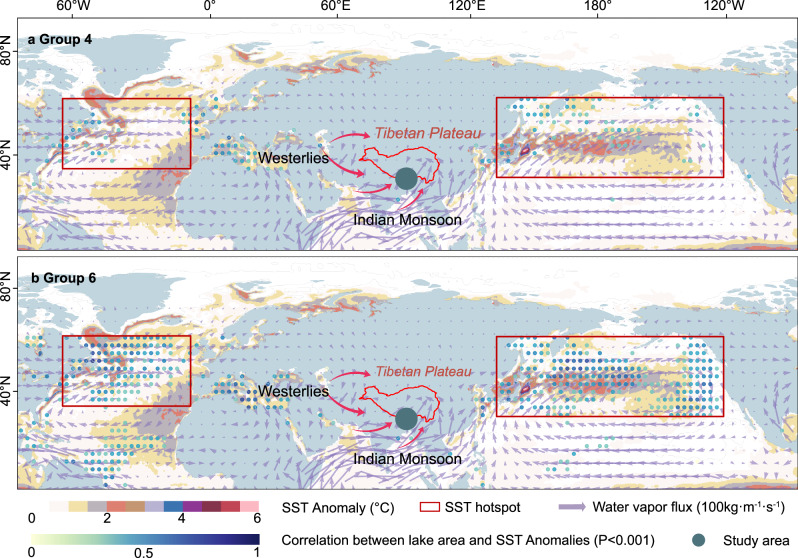
Teleconnections of supraglacial lakes area with monsoonal moisture transport and SST anomalies. **a** Group 4, Ama Dablam Glacier (Few supraglacial lakes (SGLs) with weak catchment capacity); **b** Group 6, Khumbu Glacier (Many SGLs with strong catchment capacity). Background map showing the spatial distribution of 2023 sea surface temperatures (SST) anomaly peaks. Red arrows: monsoon influence on HMA glaciers (Adapted from reference^21^). Scatter points indicate regions where seasonal fluctuations of lake area exhibit lagged correlations with SST anomalies. Credit: arrow icon, Flaticon.com.

Our data analysis indicates that the lake anomalies on the eastern Himalaya from October 2022 to February 2023 are linked to the record-breaking global SST anomalies in 2023. Based on these observations, we mapped post-monsoon and winter lake fluctuations. In the context of global warming, the seasonal fluctuations of surface waters on the HMA exhibit a lead response to SST anomalies. We quantified the linkage between Atlantic and Pacific SST anomalies and lake area along the same moisture transport pathways. SGLs on the nine glacier groups showed lagged correlations with SST anomalies, with the strongest correlations occurring 1–2 months ahead of the SST anomalies, with values of 0.72, 0.65, 0.64, 0.41, 0.68, 0.71, 0.63, 0.58, and 0.62. These correlations are particularly evident in regions where SST anomalies are relatively high (Fig. 5 and Suppl. Fig. 20). Recent research shows that warming in Asia’s high-altitude regions is modifying Northern Hemisphere weather variability and exhibits an amplification effect, whereby surface temperatures on the plateau rise faster than the global average^44^. In addition, extreme warming of water bodies has been observed in other regions worldwide in 2023^45^. These findings highlight the critical role of glacial meltwater from the HMA in providing early warning signals for extreme natural hazards.

## Discussion

Our observations indicate that pre-monsoon ice flow acceleration coincides with enhanced surface meltwater flux, while deceleration of glacier velocity is accompanied by accelerated surface water loss during the monsoon season (Fig. 4 and Suppl. Fig. 15). Meanwhile, significant differences in the amplitude of variability between ice flow and SGLs were observed on adjacent glaciers. Such heterogeneity in SGLs and glacier velocities driven by snow and ice resources has been widely observed^42,46^. By comparing the annual and seasonal glacier velocities of all nine upstream glaciers in Sagarmatha National Park as external forcing mechanisms influencing SGL variability and topographic reshaping, we conducted daily monitoring of surface area changes of SGLs in the region during 2016–2024. This allowed us to derive monthly changes of surface water fluctuations and their monthly distribution relative to SST anomalies throughout the observation period. Our results indicate pronounced seasonal dynamics in both glacier velocity and SGL area in the eastern Himalaya, with a clear linkage between lake expansion and glacier behaviour. Previous studies indicate that seasonal melting of debris-covered surfaces and glacier ice generates substantial meltwater, which drains through crevasses into the englacial hydrological system and is routed toward the glacier margins or bed. This process reduces basal traction and induces hydraulic uplift, both of which promote ice-flow acceleration^47^. Under the combined effects of accelerated flow, enhanced longitudinal strain, dynamic thinning and crevasse-driven frontal retreat, calving becomes dominant. Continued extensional flow deepens crevasses in the glacier terminus, while thinning reduces freeboard height (the elevation of the ice surface above the lake level), thereby promoting mechanical calving and frontal retreat and increasing the likelihood of ice-dam instability and failure. These processes collectively alter the geometry of glacier termini and are accompanied by surface lowering driven by ice thinning and dynamic adjustment^41,47–49^. We observed distinct water-supply patterns associated with glacier retreat. Fast-flowing glaciers exhibit overflow-type drainage, while slow-flowing glaciers display stepwise drainage (Fig. 3 and Suppl. Fig. 13). These patterns support King’s two-stage model of glacier-lake evolution in the Himalaya^47^. During the lake expansion stage, rising lake levels or thinning of the host glacier induce crevassing, accelerating frontal retreat, steepening the glacier surface, and enhancing ice-flow velocity. In the later stage of lake growth, sustained ice-front retreat and glacier thinning narrow the glacier, reducing driving stresses while increasing lateral and basal resistance, thereby slowing ice flow and diminishing retreat rates^47^. This interplay highlights the short-term concentration of meltwater and the unsustainable contribution of glacier mass loss to runoff under climate-driven glacier retreat and permafrost thaw^23^. At the same time, with future mass loss of glaciers in the Himalaya, glacier retreat and thinning will be accompanied by the formation and expansion of new lakes, as well as accelerated ice flow and instability at glacier termini^45^. For example, the correspondence between accelerated ice flow fluctuations and new lake formation at the 1–3 km segment of Glacier Group 2 in 2022 (Suppl. Fig. 14). These observations underscore the critical role of the coupled mechanism between glacier dynamics and external environmental forcing.

The occurrence of GLOFs depends on preconditions, triggers, and mechanisms. When glacier dynamics disrupt the geometry of individual glaciers, subglacial conditions, or the stability of supraglacial debris, specific triggering events (such as earthquakes or avalanches) or deglaciation and lake evolution can initiate the hazard^24,50^. In glaciers hosting SGLs and debris cover, seasonal fluctuations are primarily driven by basal sliding (subglacial water flow), the level of effective pressure – the difference between ice overburden pressure and subglacial water pressure – and the stabilizing structure formed by supraglacial debris, ice, and terminal moraines^51,52^. During the monsoon, rising temperatures and increased precipitation induce melting of ice covered by supraglacial debris and ice margins, as well as meltwater erosion, which compromise the support of supraglacial debris and weaken the stability of lake-adjacent moraine or ice dams. This inherently unstable geological configuration, combined with the occurrence of extreme climatic conditions before and during the monsoon, underlies the concentration of GLOFs in this period (Fig. 1). With progressive global warming, the regulatory role of lakes in ecosystems—including climate, wetlands, and oceans—has become increasingly recognized. Long-term monitoring of glacial lake number and area has become central to assessing outburst flood hazards. Rick et al. reported 1150 drainage events from 106 ice-dammed lakes in Alaska (1985–2020), showing stable event frequency but declining magnitude^53^. A global synthesis of 1569 events by Veh et al. further reveals an order-of-magnitude decrease in extreme peak discharge and flood volume across most regions since 1900^9^. Despite the growing completeness of global ice-dammed lake databases, the spatiotemporal response of supraglacial and small glacial lakes to climate change remains poorly constrained. Based on our analysis of seasonal SGL dynamics, we find that their short-term variability is highly sensitive to climate forcing, providing near-real-time, within-year feedback and potential early indicators of extreme climate anomalies. Meanwhile, Himalayan SGLs primarily form from surface meltwater accumulation and exhibit pronounced seasonality, whereas glacial lakes in the Andes are predominantly moraine- or ice-dammed. Alaskan lake systems are more complex, comprising ice-dammed, proglacial, and some small SGLs. In Greenland, intense summer meltwater input leads to widespread surface ponding and the formation of SGLs. Whether such spatial heterogeneity shares similarities with, or differs fundamentally from, the characteristics of Himalayan SGLs remains to be further investigated^12,42,54,55^. Therefore, future work should focus on quantifying the impacts of small SGLs on global ecosystems, high-mountain water resources, and hazard risks under different glacier retreat scenarios.

Based on the importance of small lakes, the dynamics of supraglacial and small glacial lakes still require further integration into global governance and conservation frameworks. Meanwhile, small SGLs have great destructive potential due to sudden drainage, posing severe risks to downstream landforms and infrastructure^33^. Seasonality is an important characteristic of outburst floods in high-mountain regions. Sattar et al. documented at least seven flood events in the Liming Valley, all occurring during summer, a similar seasonal pattern is also observed in the glacial lake outburst events identified in this study. This is primarily because seasonal meltwater plays an important role in destabilizing drainage systems. These findings underscore the importance of closely monitoring glacier dynamics and the seasonal evolution of SGLs^36^. Notably, as ice flow velocities in some glaciers gradually decrease, our observations indicate that the surface area of certain SGLs is progressively declining, following a rapid expansion–depletion pattern of water resources. The reduction of meltwater from the Asian “water towers” could lead to freshwater shortages affecting billions of people. Our results indicate that the contribution of glacier mass loss to runoff has yet to reach peak water (Peak contribution of glacier to runoff) (Fig. 1 and Suppl. Table 3). It is estimated that peak glacier meltwater in HMA will occur around the mid-twenty-first century^53^. To prevent the kind of resource depletion observed in the Andes after passing peak water, glacier meltwater should remain a research priority. Developing sustainable management pathways is crucial to mitigate the adverse impacts of an unsustainable cryospheric meltwater supply on high-mountain ecosystems^56–58^.

Surface runoff and meltwater infiltration represent characteristic dynamic hydrological systems on glaciers. We simultaneously observed that subtle variations in these two water-generation modes significantly influence freshwater supply in HMA. When debris-covered drainage systems primarily rely on subglacial drainage and permafrost thaw, we observed a top-down transfer of lake water resources rather than overall expansion (i.e., the expansion of water in downstream lakes originates from the depletion of upstream lakes) (Fig. 3d). For glaciers with extensive surface drainage networks and relatively high ice flow velocities, we observed the expansion and coalescence of surface lakes, whereby individual lakes gradually developed into larger lakes through hydrological connectivity (Fig. 3e and Suppl. Fig. 3). This indicates that active glaciers are coupled with subglacial drainage systems and surface hydrological networks. The evolution and redistribution of supraglacial drainage systems and debris cover can lead to shifts in lake locations, and in our study, the observed lake displacements and short-lived lifecycles reflect this phenomenon (Fig. 2). Previous studies^21,59–61^ suggest that long-term increases in debris cover and pond area accelerate glacier mass loss and enhance the storage capacity of proglacial lakes. In our observations, rapid expansion of terminus-region lakes was accompanied by local surface subsidence, whereas glaciers without lakes did not exhibit such subsidence, supporting the theory that pond expansion drives rapid loss of permafrost in glacier forefields (Suppl. Figs. 8 and 13).

During our nine-year study period, we observed pronounced seasonal fluctuations in lake area. In August 2023, a global SST anomaly generated a new peak record, with early signals emerging in March–April^62^. Interestingly, from October 2022 to February 2023, anomalous surface water conditions were already detected in the eastern Himalaya. In 2024, as SST anomalies decreased, surface water returned to the normal freeze–thaw cycle. Previous studies^63,64^ suggest that seasonal variations in glacier velocity are driven by enhanced energy fluxes within the ice–atmosphere–ocean system, and as temperature anomalies in the atmospheric circulation persistently increase, basal drag reduction and hydraulic uplift induced by surface meltwater infiltration are gradually becoming important drivers of glacier acceleration^47,63^. Upper-ocean temperatures are closely linked to high melt rates at glacier termini, and the mass balance of HMA glaciers is controlled by the intensity of the summer Indian monsoon and winter mid-latitude westerlies, as well as topographic influences. Our analysis indicates that summer 850 hPa moisture flux over the eastern Himalaya is primarily sourced from the Indian Ocean, partly explaining the frequent summer hydrological events in the region. However, SST anomalies in the Indian Ocean show no significant correlation with lake expansion on the HMA. Recent studies report a weakening Indian monsoon and strengthening westerlies^21^. In our observations, we found that lake areas on retreating glaciers were significantly correlated with Atlantic and Pacific SST anomalies, with the ocean temperature anomalies lagging the lake area by 1–2 months (Fig. 5 and Suppl. Fig. 20). Previous work^20,65^ indicates that asymmetrical lake expansion under warming is primarily driven by the interaction between westerlies and monsoons. Consistent with this, we observed that lakes in our study region shifted from a stable drainage phase to an anomalously stable phase during the autumn–winter season under global warming, with this fluctuation manifested as anomalous lake stability or abnormal expansion during October–February of 2022–2023 (Suppl. Figs. 18 and 19). These results further support the synchrony and precursor role of HMA Lake anomalies relative to global SST anomalies.

Lakes and hydrological systems are highly sensitive to long-term climate warming. With the increasing frequency and intensity of extreme events worldwide, lake and river ecosystems are emerging as important indicators for the early warning of global hazards. Previous studies^66–68^ suggest that the rapid rise in lake surface water temperature is closely linked to intensified summer heat extremes, with anomalies primarily driven by surface air temperature and further modulated by local lake characteristics and ice-cover dynamics. Riverine heatwaves (weekly mean water temperature > 90th percentile) are also intensifying, highlighting the growing vulnerability of hydrological systems to climate extremes^69^. Current global lake models and early-warning frameworks remain largely focused on large lakes and river systems. However, small mountain lakes have been shown to exhibit amplified responses to climate variability. Consistent with this, our results demonstrate that small SGLs respond rapidly to climate change, with short-term fluctuations acting as sensitive indicators of environmental variability. Incorporating the seasonal dynamics of small SGLs into existing early-warning frameworks, and developing integrated systems across multiple lake scales, is therefore critical for improving the prediction and response capacity for extreme climate events and abrupt hydrological hazards.

## Methods

### Supraglacial lake areas

Planet imagery, with its high spatiotemporal resolution and frequent revisit capability, enables the analysis of individual lake filling and drainage events. To assess the dynamics of lake surface area over time, it is necessary to delineate lake boundaries. From June 2016 to December 2024, we acquired 13,135 daily PlanetScope images across nine glacier groups in the eastern Himalaya to map the dynamic extent of surface water (Suppl. Table 5). Extraction of small SGLs and ponds remains particularly challenging due to the limited generalization capability of current deep learning models. Therefore, we manually interpreted all remote sensing images to ensure the usability of surface water data across the nine glacier groups. For each image, we applied an improved normalized difference water index (NDWI) approach, establishing an initial threshold to extract glacier water boundaries. SGLs exhibit substantial variability in color due to differences in the content of icebergs or floating ice, suspended sediments, and other properties. Highly turbid water can resemble wet sediments or morainic material, which may introduce bias during visual interpretation and render manual annotation prone to subjective errors. To avoid such issues, we conducted a second round of review, systematically re-examining and adjusting the image thresholds and individual lakes within each glacier, thereby minimizing potential human errors. Visual interpretation of lakes can be affected by cloud cover, snow and ice interference, lake-surface noise, and topographic variability, leading to non-negligible errors in certain areas. Moreover, in high-altitude regions, rapid temperature fluctuations can cause small lakes to freeze or thaw within a short period, making their states undetectable. Therefore, when missing values could not be reliably recovered through manual interpretation, we used remote sensing imagery from adjacent dates to fill the gaps. Using the above approach, we calculated the annual change frequency of each SGL. After generating a total of 81 time-series images across the nine glacier groups, we again manually delineated the lake boundaries on each image. Four rounds of screening were conducted to ensure the robustness and reliability of the hydrological dataset used in this study. Concurrently, to capture long-term seasonal fluctuations of SGLs, we employed monthly standard deviation and standard error of water area to quantify the ice–water state and spatiotemporal dynamics, thereby characterizing variations in lake spatial coverage and seasonal fluctuations (Fig. 4)

Seasonal intra-annual fluctuations of SGLs may create gaps within a single lake, which in some cases may split a lake into multiple boundaries, resulting in several lakes instead of one. In rare instances, genuinely isolated or closely adjacent lakes may be erroneously merged; however, given the relatively high resolution of our imagery, such errors are considered minor compared with the benefits of avoiding incorrect lake splitting. For these reasons, we selected 0.045 × 10^4^ m^2^ (0.5 Landsat pixels) as the minimum mapping unit, and classified lake sizes into 0.5, 1, 4, 9, 16, and 25 Landsat pixels. To investigate variations across different glacier-lake gradients and track temporal changes of individual lakes, we extracted surface elevations of glacier centerlines from the Copernicus Digital Elevation Model (COP-DEM, 30 m resolution)^70^. Centroids of each lake were extracted from annual lake extents from 2016 to 2024, and the maximum migration distance and corresponding elevation change over the study period were quantified to characterize centroid displacement and migration gradients. For each lake, transects perpendicular to the glacier centerline were constructed, and the spatial distribution of lake displacement gradients was mapped (Fig. 2).

Twelve independent glaciers were classified into nine groups based on whether they share a common outlet (Suppl. Table 2). As these glaciers comprise 19 glacier branches, we extracted annual displacements of SGLs along transects corresponding to each glacier branch, and quantified the transfer effects of upstream lakes on downstream lake water volumes over their lifecycles (Figs. 2 and 3). Using trend analysis via least-squares linear regression, we constructed the relationships between the original and downstream lake surface extents surrounding drainage events in retreating and depleting SGLs. This was used to analyze the lake’s change trends before the drainage events and to assess whether the area continued to exhibit water storage trends afterward, and corresponding daily time series for the lakes were also generated (Fig. 3c). These fine-scale spatial characteristics of lakes are highly sensitive to surface deformation and temperature changes, providing an effective indicator of local mountain hazards and potential risks associated with extreme global climate events.

### Ice velocity

We investigated the correlations between lake area dynamics and ice velocity, while considering seasonal to multi-year timescales. To this end, 94 Sentinel-2 optical images from the European Space Agency were employed to map the surface velocity of nine upstream glaciers. For interannual glacier flow, all possible image pairs along the same orbit were formed with a repetition period of one year (340–390 days). Monthly velocities were analyzed to capture the periodic fluctuations of the South Asian summer monsoon over the HMA, with specific periods listed in Suppl. Table 7. Observations were processed following the methods described in refs. ^41,71^. Specifically, surface feature displacements were tracked using a frequency-domain cross-correlation algorithm, with an initial search window of 128 × 128 pixels and a final sub-pixel refinement using 32 × 32 pixels templates with two coarsely estimated offsets. A step size of 2 pixels was applied, yielding glacier centerline surface velocities at 20 m resolution. Non-glaciated areas were assumed to have zero displacement to correct for systematic errors associated with the optical tracking results. For each velocity field, denoising was performed using a non-local means filter, excluding all pixels with a signal-to-noise ratio below 0.9. The filter parameters were set to 1.6 times the mean standard deviation, with a search window of 21 × 21 pixels and a patch size of 5 × 5 pixels^41,71^.

### Surface deformation

When optical or laser instruments relying on visible light are used to derive optical flow fields for glacier monitoring, cloud cover and shadows can induce temporal discontinuities in the data. Radar instruments, in contrast, can penetrate glacier snow, but the penetration depth (also referred to as the scattering horizon) exhibits spatiotemporal heterogeneity. The presence of surface melt, combined with snow density and liquid water content, can elevate the scattering horizon closer to the snow surface due to the side-looking geometry of radar sensors. This can introduce subtle apparent horizontal displacements along the radar line of sight, resulting in measurement errors in glacier surface velocity. Therefore, we applied Sentinel-2 imagery to monitor the seasonal dynamics of glacier flow (glacier surface velocity) and used 233 Sentinel-1 synthetic aperture radar (SAR) images to map terminal moraine deformation at the glacier termini.

The SBAS-InSAR algorithm is a time-series InSAR approach that generates a set of small-baseline interferograms using a multi-master strategy with appropriately defined spatiotemporal baseline thresholds, enhancing interferometric coherence and enabling millimeter- to centimeter-scale monitoring of surface deformation. N + 1 single-look complex (SLC) SAR images acquired over the study area at consecutive time points $t0,\,\cdots,{t}N$ are processed by selecting a single master image, to which all other images are co-registered and a unified reference grid is established. Differential interferograms are generated from the SLC images, and topographic phase is removed using an external digital elevation model (e.g., AW3D 30 m). Flat and highly coherent stable points (e.g., bedrock) are used as zero-displacement reference points. Following filtering and phase unwrapping, continuous phase time series are obtained for each coherent target, comprising deformation, residual topography, atmospheric, orbital, and noise components. Orbital errors are corrected via least-squares fitting, residual topography is estimated from the linear relationship between perpendicular baseline and elevation, and atmospheric contributions are separated using temporal high-pass and spatial low-pass filtering. Singular value decomposition (SVD) is then applied to convert the multi-master interferogram set into a single-reference time series^72^, with unwrapped phase converted into LOS displacement. In the LOS direction, negative values indicate motion away from the satellite, whereas positive values indicate motion toward it. In this study, these deformation patterns manifest as subsidence and deposition in the glacier terminus zone, respectively. Given that InSAR measurements are acquired along the LOS direction and based on the geometric projection relationship, the vertical displacement can be approximated as $d{vertical}=d{LOS}/{COS}\theta$, where θ is the incidence angle^73^. All processing was performed using GAMMA software.

### Statistical assessment of lakes and SST anomalies

The observed SST anomalies were derived from the NOAA Optimum Interpolation (OI) SST dataset, version 2, with a 0.25° gridded resolution. This dataset integrates satellite, ship-based, and buoy observations, providing global coverage and effectively capturing spatial and temporal variability^68^. Since the constructed SST anomalies are based on both the temporal warming trends and local spatial deviations, this approach accounts for spatial heterogeneities in radiative forcing, monsoon dynamics, and heat capacity. Consequently, lag effects were incorporated into the lagged correlation analysis between SST anomalies and SGL dynamics. Additionally, daily atmospheric data at 2.5° spatial resolution were obtained from the NOAA National Centers for Environmental Information, including wind, specific humidity, and other variables. This dataset provides 17 standard pressure levels from the surface (1000 hPa) to the upper atmosphere (10 hPa).

Variations in moisture flux are jointly determined by wind speed and specific humidity, representing dynamic and thermodynamic processes, respectively^74^. Following the theoretical framework of references^21,62,64,74^, we constructed schematic diagrams illustrating the influence of the westerlies and Indian Summer Monsoon on HMA lakes. Low-level (850 hPa) moisture fluxes were calculated from meridional and zonal wind components and specific humidity to quantify the external forcing effects of the westerlies and monsoon over the HMA. Global mean SST reached a new single-day record in March 2023 and subsequently remained at historically high levels, with additional record-breaking daily values in April, July, and August, attributed to unprecedented global surface warming^62,75,76^. Although extreme temperatures over land and ocean surfaces are not necessarily correlated, such near-synchronous extreme temperature excursions carry particular significance for both the public and the scientific community^77,78^.

### Sources of error

We focused on quantifying the seasonal dynamics of surface water bodies from 2016 to 2024. The primary sources of uncertainty stem from biases in manual image interpretation and gaps in the remote sensing data. To assess the dynamic surface extent of each lake, it is necessary to delineate lake boundaries. For manually delineated lakes, uncertainty is typically it is calculated as lake perimeter multiplied by 0.5-pixel size^67,69^. Therefore, we considered a 1.5 m perimeter around the lake, extracted from Planet imagery, as the error zone. In previous studies on SGLs volume, lakes were often approximated as three-dimensional ellipsoids; in this study, we treat lake area as a two-dimensional ellipse. Accordingly, the main source of error is concentrated within the 1.5 m boundary around the elliptical lakes. The error was calculated using the formula $1-\frac{(l-1.5)\times (h-1.5)}{h\,l}$ (Here, l and h represent the semi-major and semi-minor axes of the ellipse, respectively). For example, when $h=l=100\,{{{\rm{m}}}}$, the error is approximately 2.98%. In this study, a total of 13,135 satellite images were acquired, covering approximately 1500 images per glacier and about 170 images per year, corresponding to roughly half of the days in a year. However, due to high climate variability in the region, brief periods of rain or snow could alter surface water conditions, and thus the annual peak lake extents may not have been fully captured in some years. Given the high spatial resolution of the satellite imagery and the fact that roughly half of the days in each month were covered, our results are expected to be robust.

### Reporting summary

Further information on research design is available in the Nature Portfolio Reporting Summary linked to this article.

## Supplementary information

Supplementary Information

Peer Review File

Reporting Summary

## Source data

Source Data

## Data Availability

Source data used in this study are available as follows: Copernicus Sentinel-1A/B & Sentine-2, available directly from the European Space Agency https://scihub.copernicus.eu/; Planet images are available under a basic license from the Education and Research programme from Planet Labs Inc. through the Planet Explorer https://www.planet.com/explorer/. The GLOF Database V4.1 is freely available at http://glofs.geo ecology.uni-potsdam.de. The RGI V6.0 is freely available at https://www.glims.org/RGI/rgi60_dl.html. NOAA OI SST v2 data are available online at http://www.esrl.noaa.gov/psd/data/gridded/data.noaa.oisst.v 2.html. Meteorological monitoring data from stations near the glaciers are freely available at https://zenodo.org/records/15203896. The specific humidity and wind speed data are freely available at https://downloads.psl.noaa.gov/Datasets/. The Copernicus DEM data are freely available at https://dataspace.copernicus.eu/. The ALOS AW3D30 DSM data are freely available at https://www.eorc.jaxa.jp/ALOS/en/dataset/aw3d30/. The supraglacial lake data generated in this study have been deposited in the Zenodo repository and are available at 10.5281/zenodo.20428717. Source data are provided with this paper.

## Code availability

The Python code and Origin software used for figure generation in this study are available on Zenodo (10.5281/zenodo.19708433).
