# Supplementary material for: Unraveling daily dynamics of supraglacial lakes in response to hydrological pulses and anomalous climate signals
Source: Nat Commun. 2026 Jul 17;17:7930. doi: 10.1038/s41467-026-75669-3 (PMC13448815; doi:10.1038/s41467-026-75669-3)
Supplement: Supplementary file 1 [file 41467_2026_75669_MOESM1_ESM.pdf]

Supplementary Information for

Unraveling daily dynamics of supraglacial lakes in response to hydrological pulses and anomalous climate signals

Jian Song[1, †], Haijun Qiu[1, 2 *], Adam Emmer[3, †], Guoqing Zhang[4, 5, †], Ninglian Wang[1, 2], Ya Liu[1], Guoqing Yang[1], Yiwen Liang[1]

[1]Shaanxi Provincial Key Laboratory of Earth Surface System and Environmental Carrying Capacity, College of Urban and Environmental Sciences, Northwest University, Xi'an 710127, China

[2]Institute of Earth Surface System and Hazards, College of Urban and Environmental Sciences, Northwest University, Xi'an 710127, China

[3]Department of Physical Geography and Geoecology, Faculty of Science, Charles University, 128 43 Prague, Czechia

[4]Key Laboratory of Biodiversity and Environment on the Qinghai-Tibetan Plateau, Ministry of Education, School of Ecology and Environment, Xizang University, Lhasa, China

[5]State Key Laboratory of Tibetan Plateau Earth System, Environment and Resources (TPESER), Institute of Tibetan Plateau Research, Chinese Academy of Sciences, Beijing, China

These authors contributed equally (†): Jian Song, Adam Emmer, Guoqing Zhang

Corresponding author (*):

Haijun Qiu (haijunqiu@nwu.edu.cn)

This file includes:

Supplementary Figures 1–20

Supplementary Tables 1–7

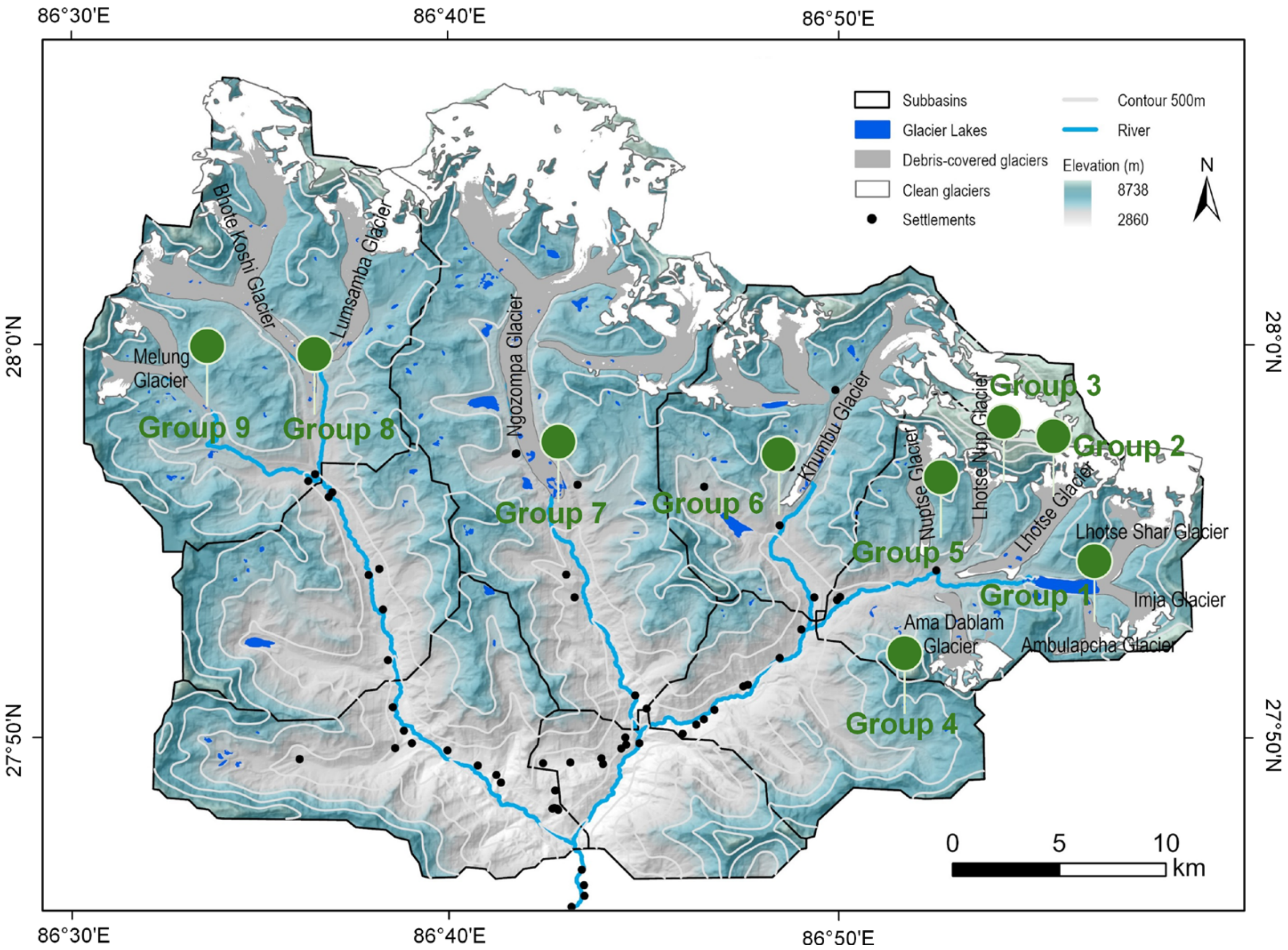

Supplementary Figure 1: Spatial distribution and classification of glaciers. Glaciers sharing the same drainage outlet were grouped together and labeled as Glacier Group 1–9. Except for Glacier Groups 1 and 8, which contain two and three individual glaciers, respectively, each of the remaining groups consists of a single glacier. The details are as follows: Group 1, Lhotse Shar、Imja and Ambulapcha; Group 2, Lhotse; Group 3, Lhotse Nup; Group 4, Ama Dablam; Group 5, Nuptse; Group 6, Khumbu; Group 7, Ngozumpa; Group 8, Bhote Koshi and Lumsamba and Group 9, Melung Glaciers.

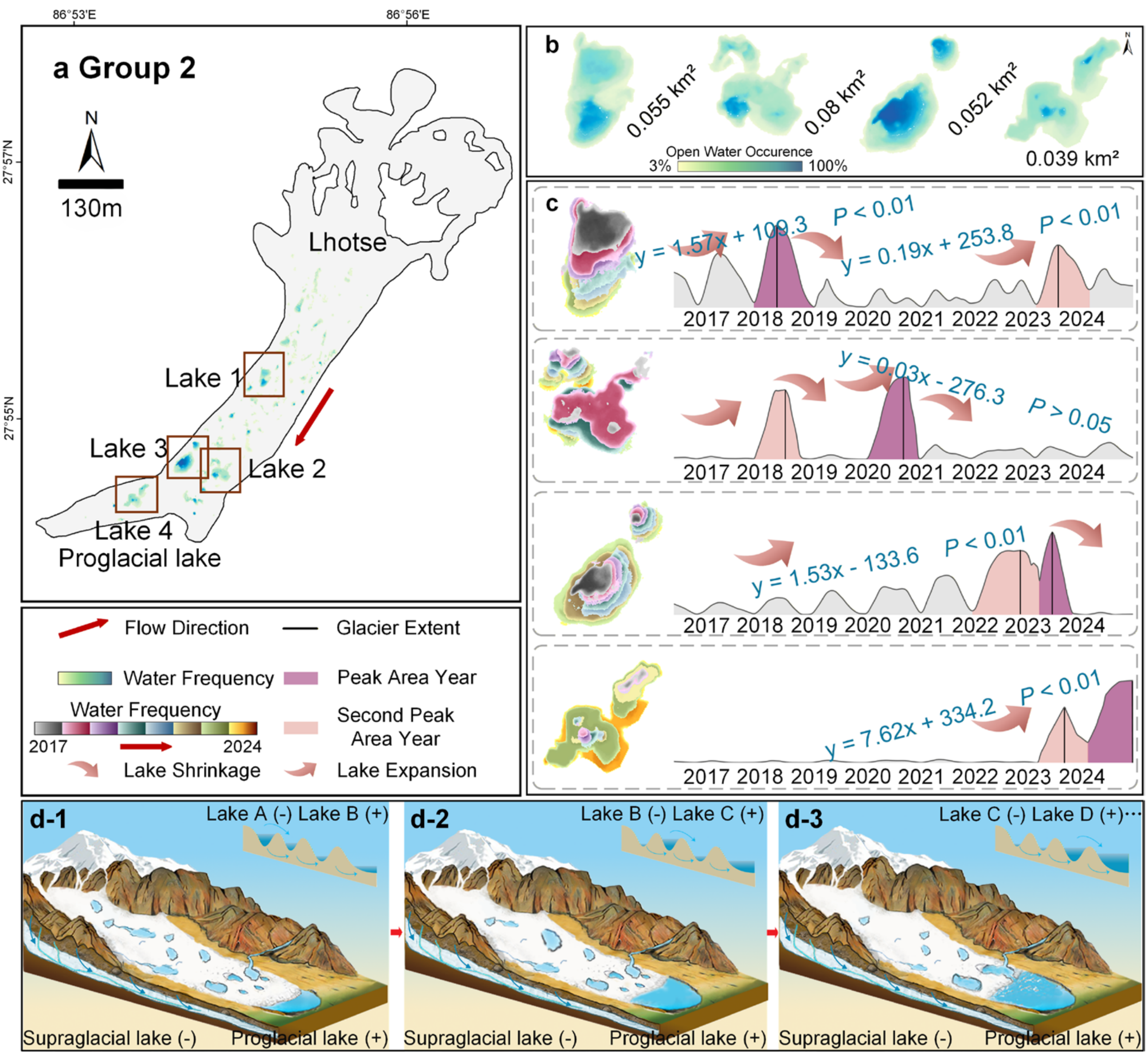

Supplementary Figure 2: Hydrological connectivity and cascading drainage among supraglacial lakes. a Frequency of lake occurrence in Glacier Group 2 from 2016 to 2024, with the four typical lakes highlighted. **b** Lakes are arranged from left to right corresponding to Lakes 1–4 in panel a, with the maximum area and occurrence frequency shown for each lake over the study period. **c** Annual frequency of supraglacial lakes and their cascading drainage processes, with locally weighted scatterplot smoothing fitted curves for lakes of interest. The x-axis of the ridgeline plot represents the time series of glacial lake area, with filled areas indicating the first and second peaks of lake area. The frequency plots on the left use different gradient colors to show the annual distribution of lakes, illustrating upstream lake incision and downstream proglacial lake aggradation. **d** a pattern of downstream lake expansion accompanied by upstream lake decline.

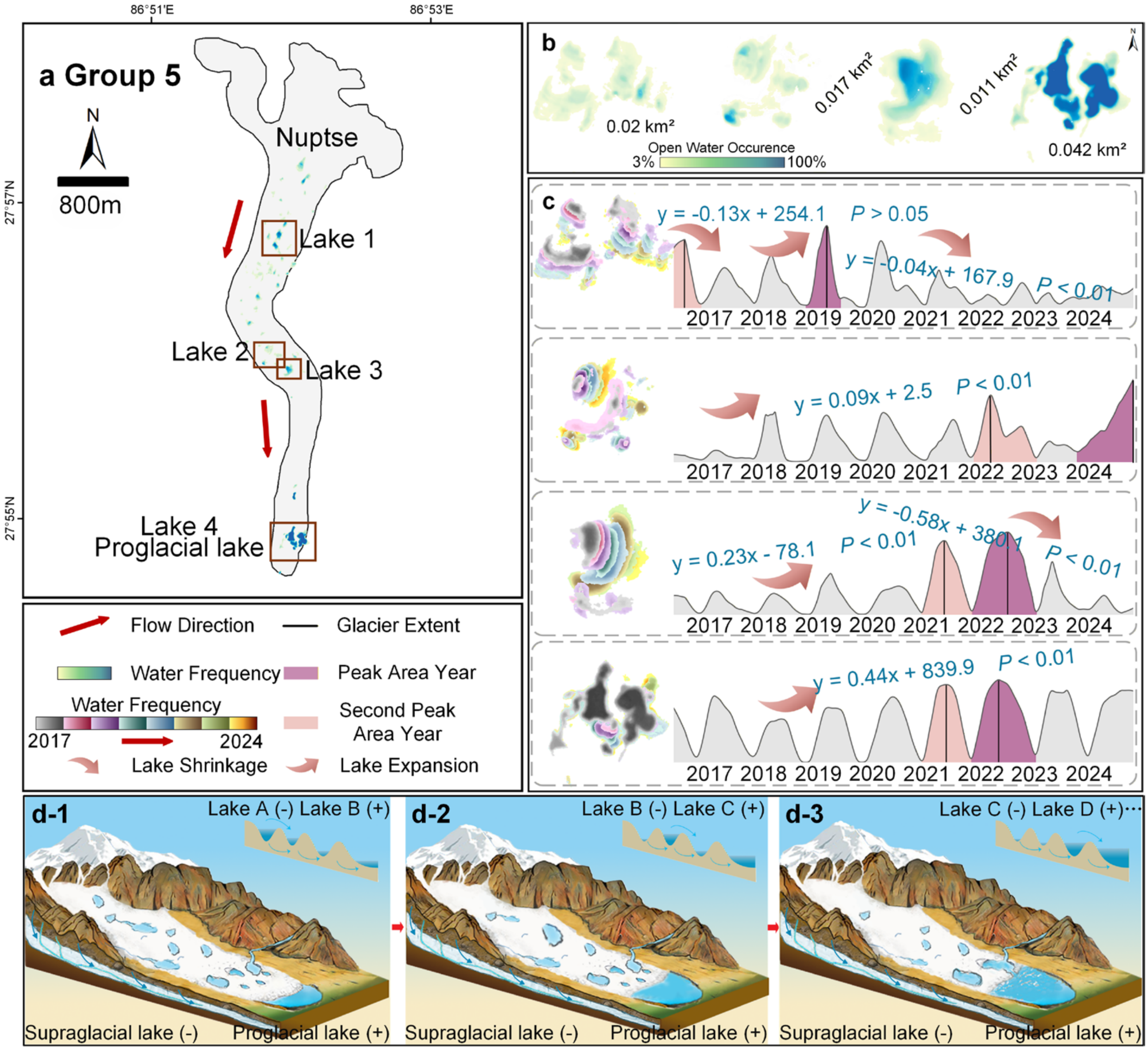

Supplementary Figure 3: Hydrological connectivity and cascading drainage among supraglacial lakes. a Frequency of lake occurrence in Glacier Group 5 from 2016 to 2024, with the four typical lakes highlighted. **b** Lakes are arranged from left to right corresponding to Lakes 1–4 in panel a, with the maximum area and occurrence frequency shown for each lake over the study period. **c** Annual frequency of supraglacial lakes and their cascading drainage processes, with locally weighted scatterplot smoothing fitted curves for lakes of interest. The x-axis of the ridgeline plot represents the time series of glacial lake area, with filled areas indicating the first and second peaks of lake area. The frequency plots on the left use different gradient colors to show the annual distribution of lakes, illustrating upstream lake incision and downstream proglacial lake aggradation. **d** a pattern of downstream lake expansion accompanied by upstream lake decline.

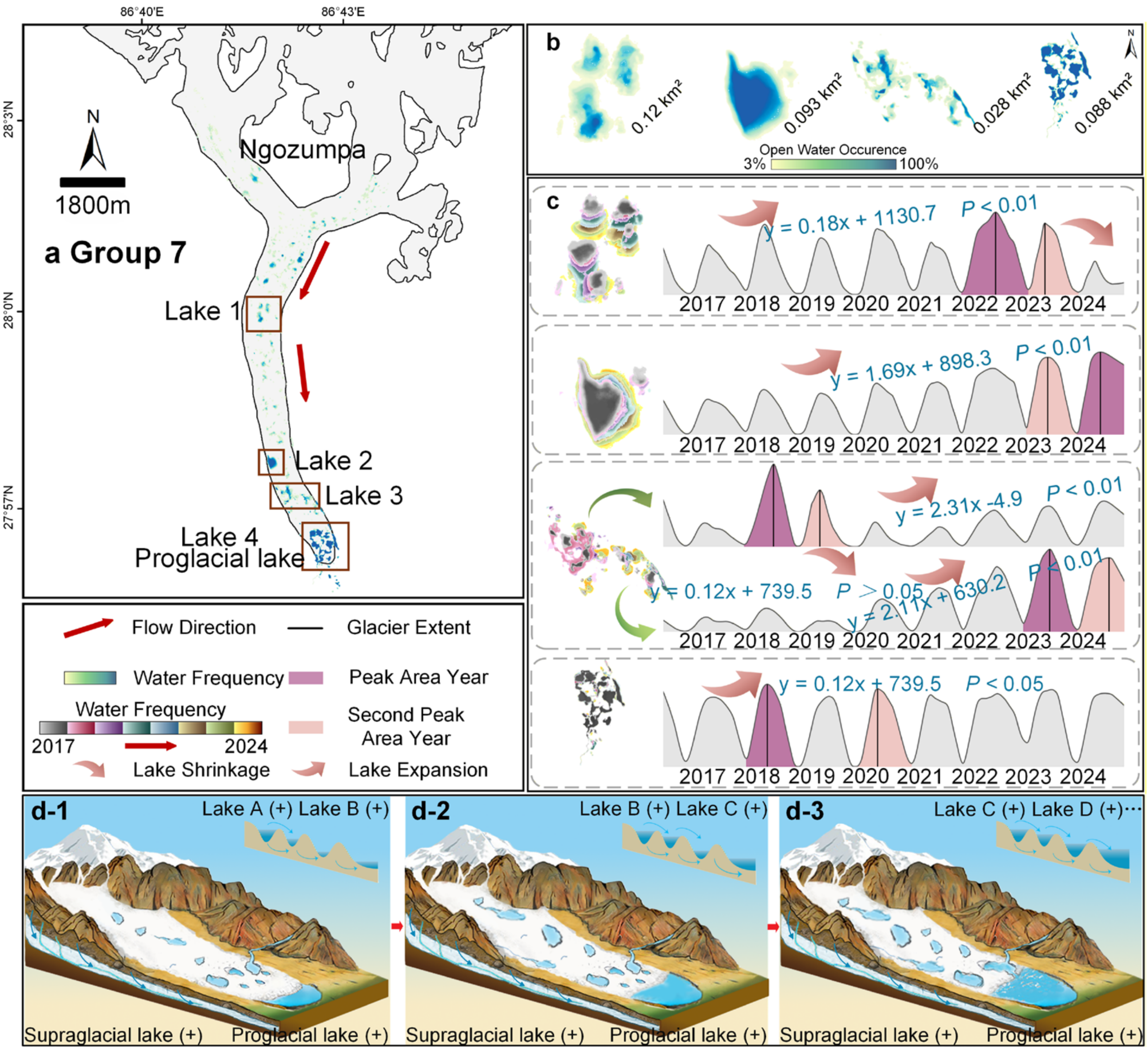

Supplementary Figure 4: Hydrological connectivity and cascading drainage among supraglacial lakes. **a** Frequency of lake occurrence in Glacier Group 7 from 2016 to 2024, with the four typical lakes highlighted. **b** Lakes are arranged from left to right corresponding to Lakes 1–4 in panel a, with the maximum area and occurrence frequency shown for each lake over the study period. **c** Annual frequency of supraglacial lakes and their cascading drainage processes, with locally weighted scatterplot smoothing fitted curves for lakes of interest. The x-axis of the ridgeline plot represents the time series of glacial lake area, with filled areas indicating the first and second peaks of lake area. The frequency plots on the left use different gradient colors to show the annual distribution of lakes, illustrating upstream lake incision and downstream proglacial lake aggradation. **d** a pattern of downstream lake expansion without upstream lake decline.

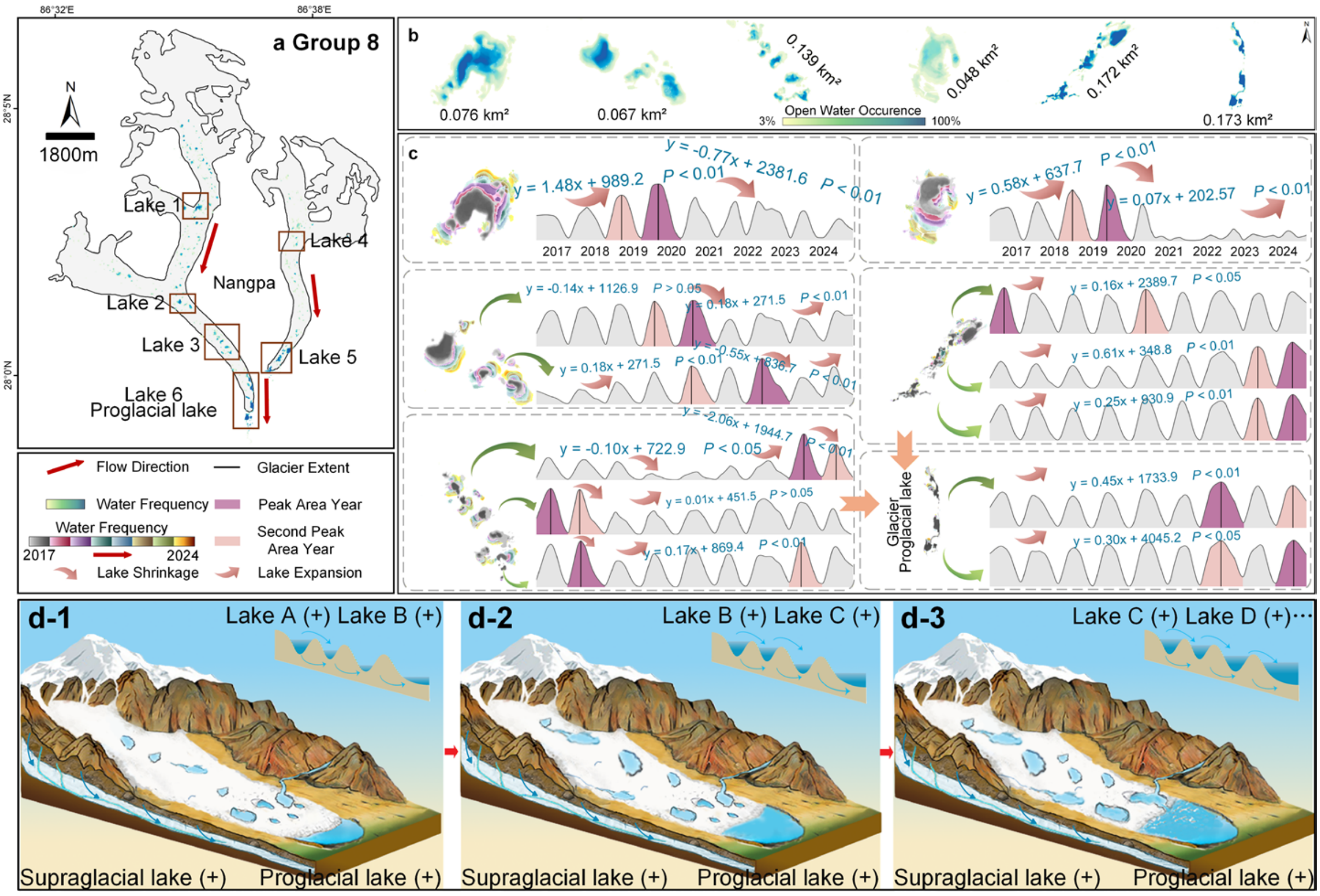

Supplementary Figure 5: Hydrological connectivity and cascading drainage among supraglacial lakes. a Frequency of lake occurrence in Glacier Group 8 from 2016 to 2024, with the four typical lakes highlighted. **b** Lakes are arranged from left to right corresponding to Lakes 1–4 in panel a, with the maximum area and occurrence frequency shown for each lake over the study period. **c** Annual frequency of supraglacial lakes and their cascading drainage processes, with locally weighted scatterplot smoothing fitted curves for lakes of interest. The x-axis of the ridgeline plot represents the time series of glacial lake area, with filled areas indicating the first and second peaks of lake area. The frequency plots on the left use different gradient colors to show the annual distribution of lakes, illustrating upstream lake incision and downstream proglacial lake aggradation. **d** a pattern of downstream lake expansion without upstream lake decline.

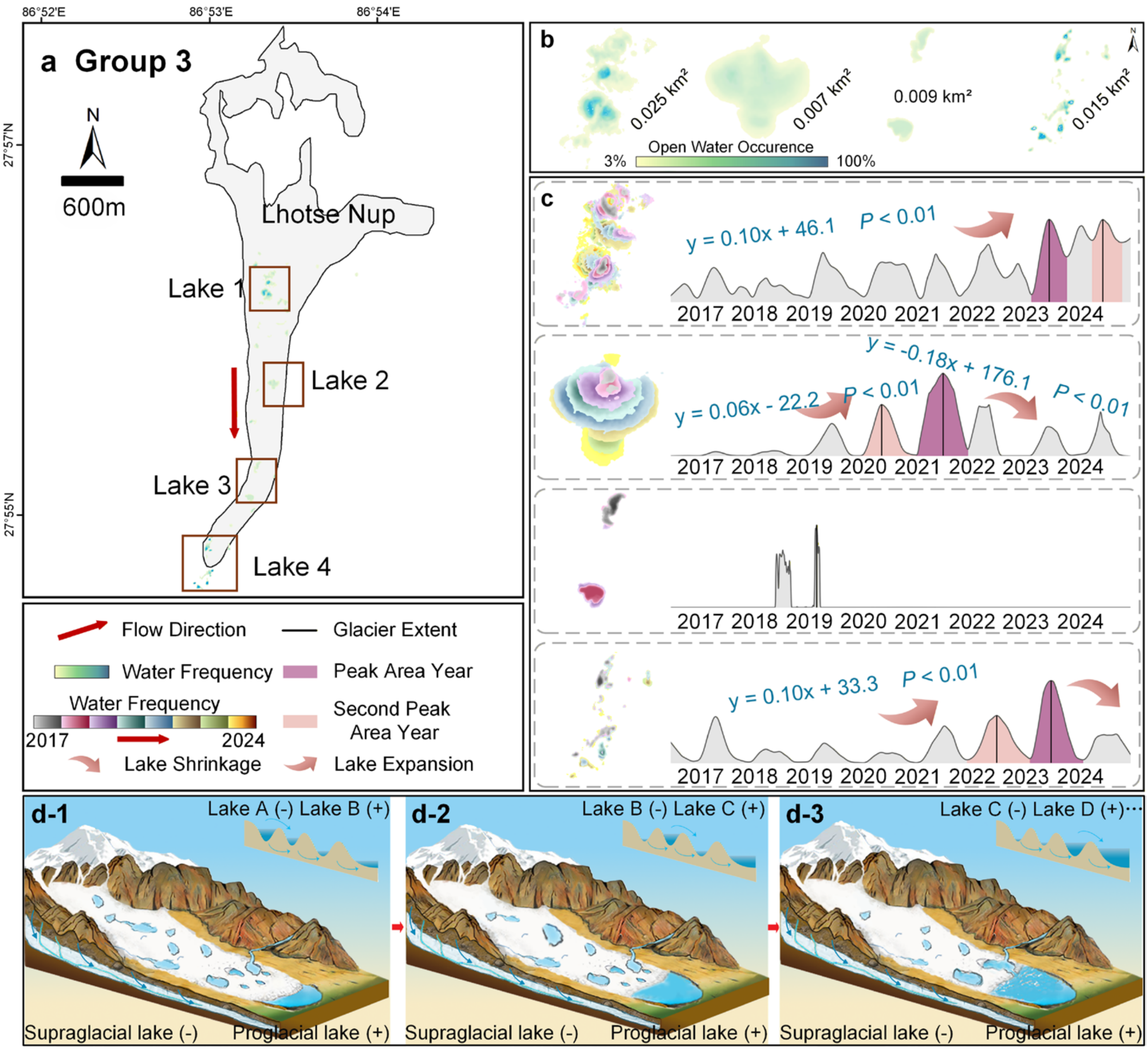

Supplementary Figure 6: Hydrological connectivity and cascading drainage among supraglacial lakes. a Frequency of lake occurrence in Glacier Group 3 from 2016 to 2024, with the four typical lakes highlighted. **b** Lakes are arranged from left to right corresponding to Lakes 1–4 in panel a, with the maximum area and occurrence frequency shown for each lake over the study period. **c** Annual frequency of supraglacial lakes and their cascading drainage processes, with locally weighted scatterplot smoothing fitted curves for lakes of interest. The x-axis of the ridgeline plot represents the time series of glacial lake area, with filled areas indicating the first and second peaks of lake area. The frequency plots on the left use different gradient colors to show the annual distribution of lakes, illustrating upstream lake incision and downstream proglacial lake aggradation. **d** a pattern of downstream lake expansion accompanied by upstream lake decline.

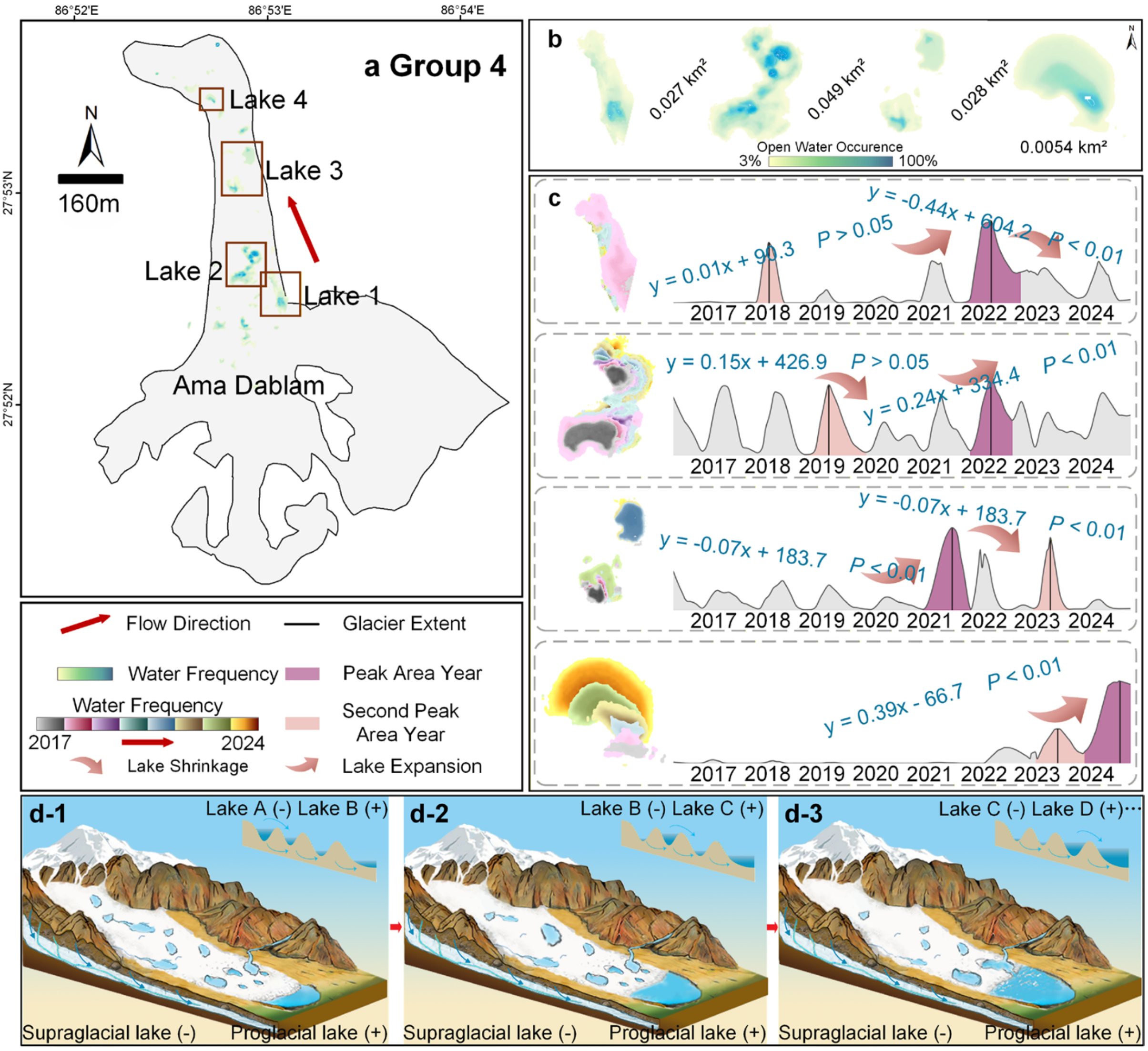

Supplementary Figure 7: Hydrological connectivity and cascading drainage among supraglacial lakes. a Frequency of lake occurrence in Glacier Group 4 from 2016 to 2024, with the four typical lakes highlighted. **b** Lakes are arranged from left to right corresponding to Lakes 1–4 in panel a, with the maximum area and occurrence frequency shown for each lake over the study period. **c** Annual frequency of supraglacial lakes and their cascading drainage processes, with locally weighted scatterplot smoothing fitted curves for lakes of interest. The x-axis of the ridgeline plot represents the time series of glacial lake area, with filled areas indicating the first and second peaks of lake area. The frequency plots on the left use different gradient colors to show the annual distribution of lakes, illustrating upstream lake incision and downstream proglacial lake aggradation. **d** a pattern of downstream lake expansion accompanied by upstream lake decline.

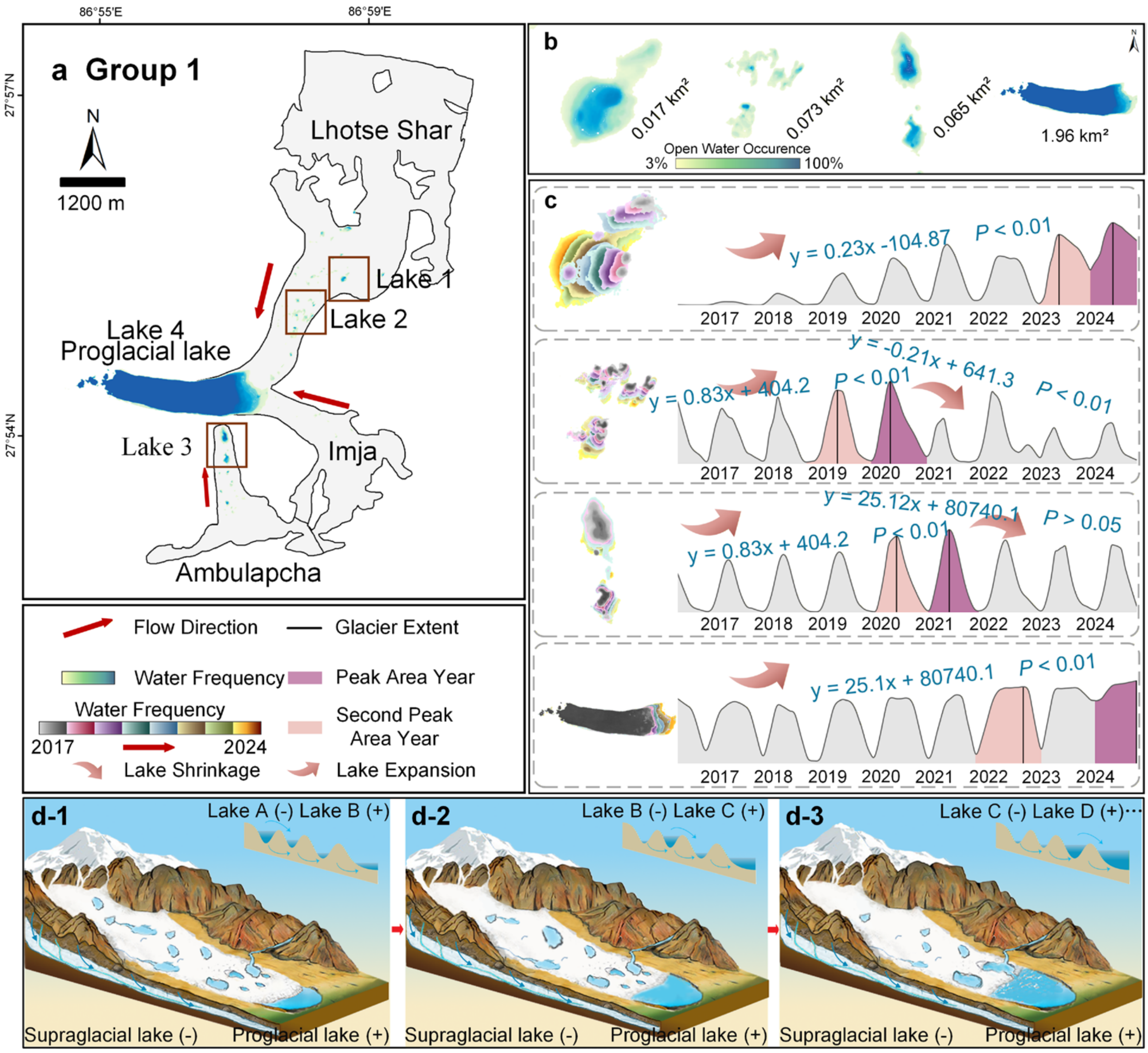

Supplementary Figure 8: Hydrological connectivity and cascading drainage among supraglacial lakes. a Frequency of lake occurrence in Glacier Group 1 from 2016 to 2024, with the four typical lakes highlighted. **b** Lakes are arranged from left to right corresponding to Lakes 1–4 in panel a, with the maximum area and occurrence frequency shown for each lake over the study period. **c** Annual frequency of supraglacial lakes and their cascading drainage processes, with locally weighted scatterplot smoothing fitted curves for lakes of interest. The x-axis of the ridgeline plot represents the time series of glacial lake area, with filled areas indicating the first and second peaks of lake area. The frequency plots on the left use different gradient colors to show the annual distribution of lakes, illustrating upstream lake incision and downstream proglacial lake aggradation. **d** a pattern of downstream lake expansion accompanied by upstream lake decline.

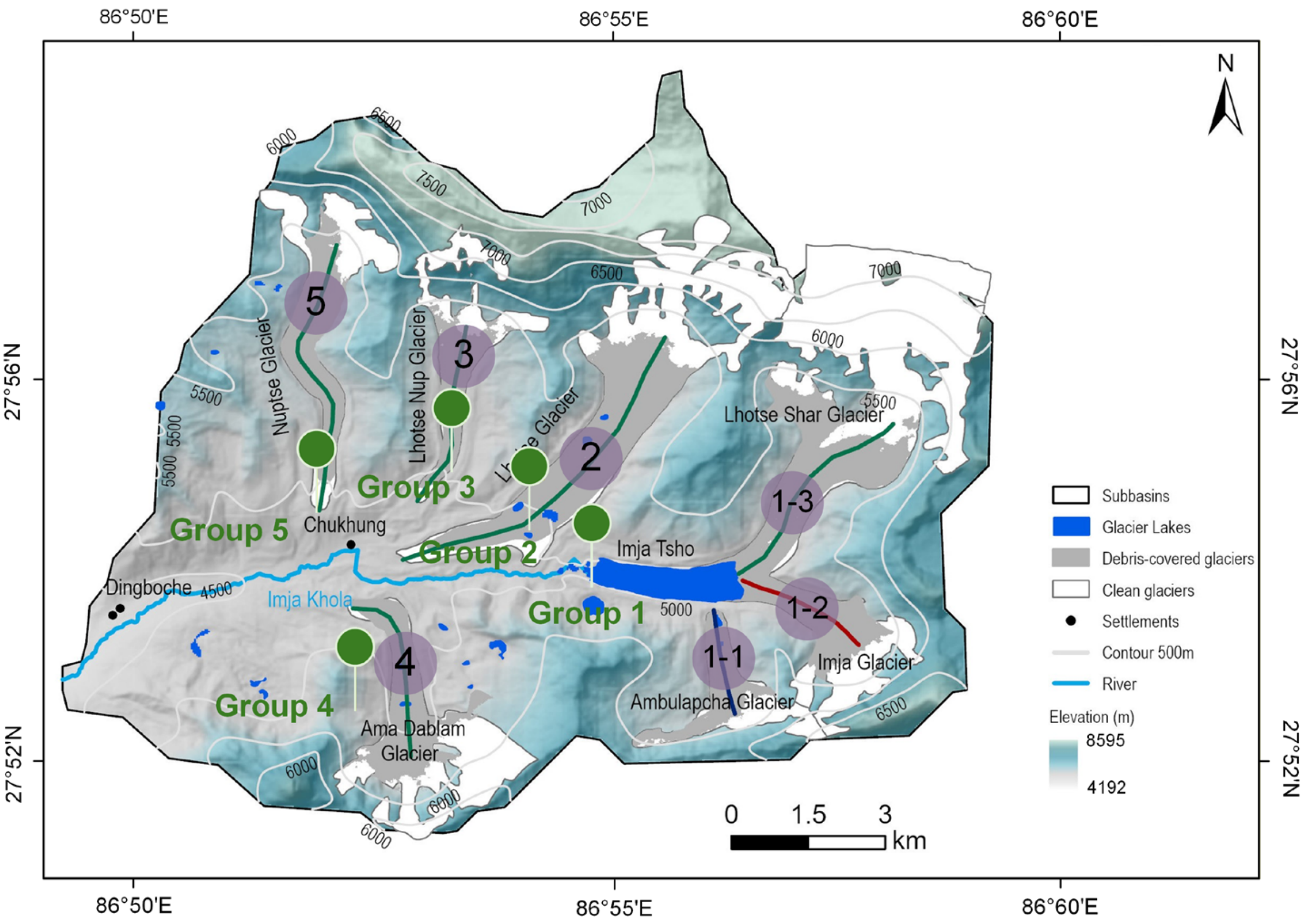

Supplementary Figure 9: Criteria for glacier system classification and spatial distribution of glaciers. Group 1-5 represent the spatial distribution of five glacier systems, and the numbers correspond to the glacier branches in Fig. 2. The relationships are as follows: Group 1, Lhotse Shar Glacier (circled 1-1), Imja Glacier (circled 1-2), and Ambulapcha Glacier (circled 1-3); Group 2, Lhotse Glacier (circled 2); Group 3, Lhotse Nup Glacier (circled 3); Group 4, Ama Dablam Glacier (circled 4); and Group 5 Nuptse Glacier (circled 5).

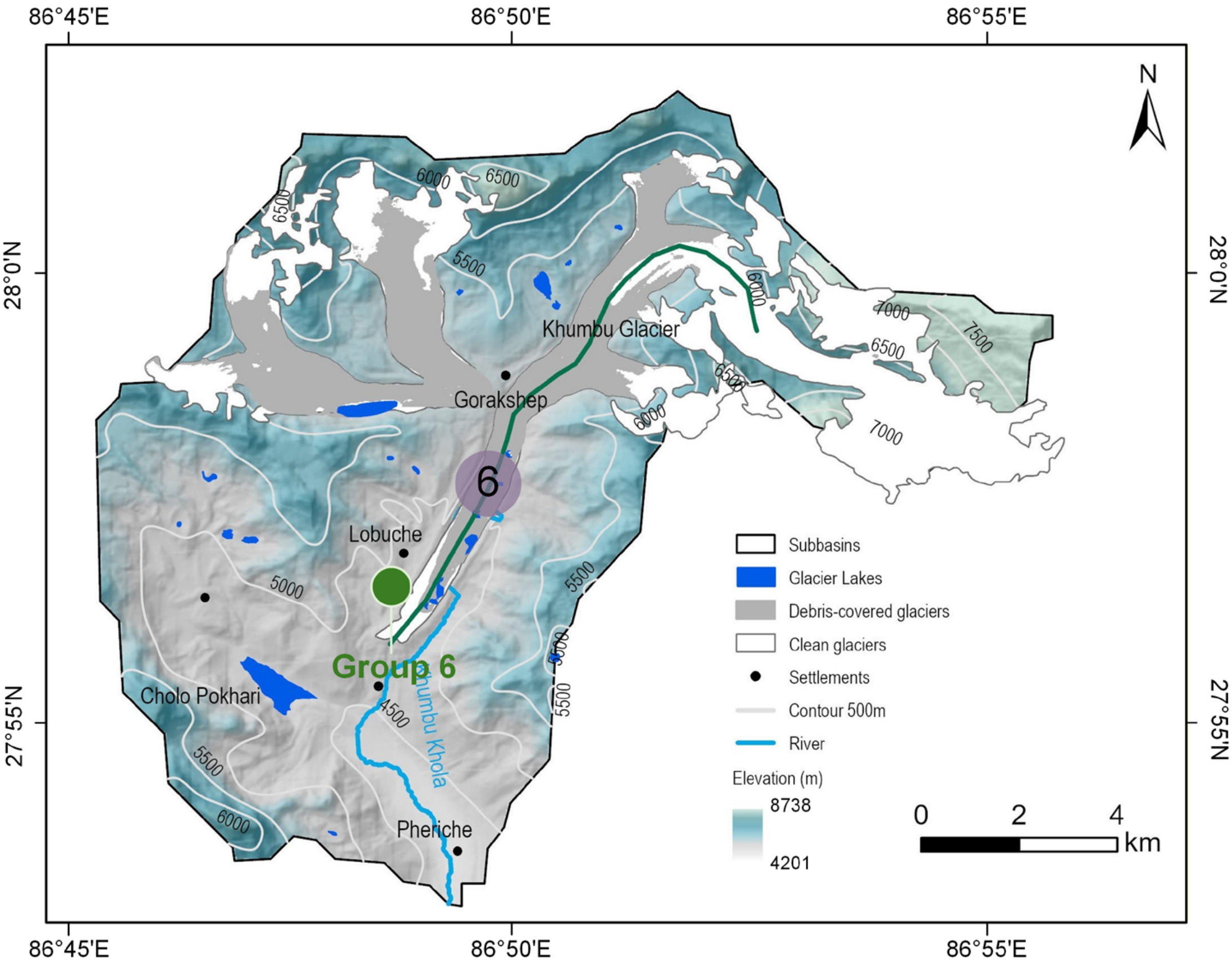

Supplementary Figure 10: Criteria for glacier system classification and spatial distribution of glaciers. Group 6 represents the spatial distribution of the glacier group, and the number corresponds to the glacier branch in Fig. 2. The correspondence is as follows: Group 6, Khumbu Glacier (circled 6).

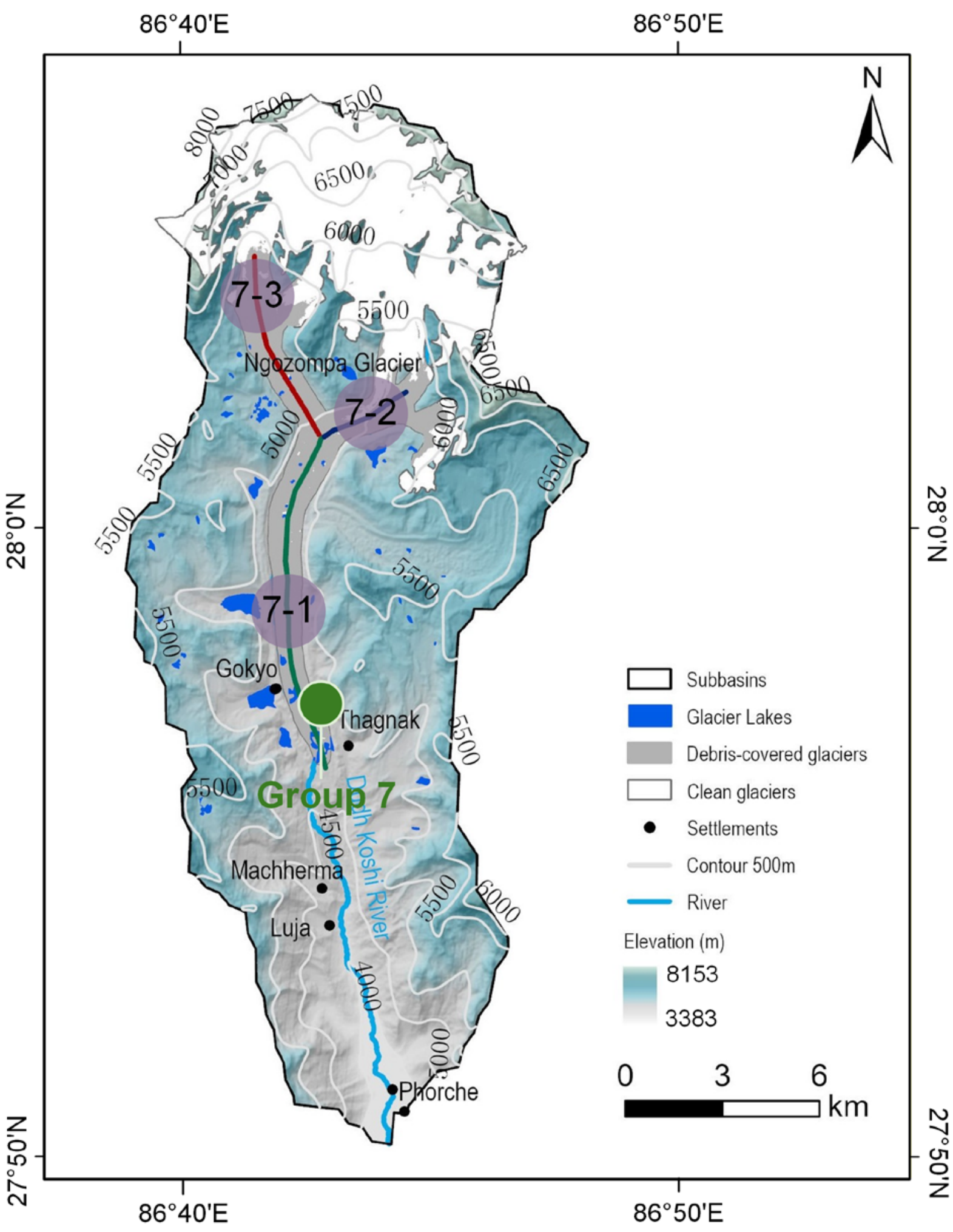

Supplementary Figure 11: Criteria for glacier system classification and spatial distribution of glaciers. Group 7 represents the spatial distribution of the glacier group, and the number corresponds to the glacier branch in Fig. 2. The correspondence is as follows: Group 7, Ngozumpa Glacier (circled 7-1, 7-2 and 7-3).

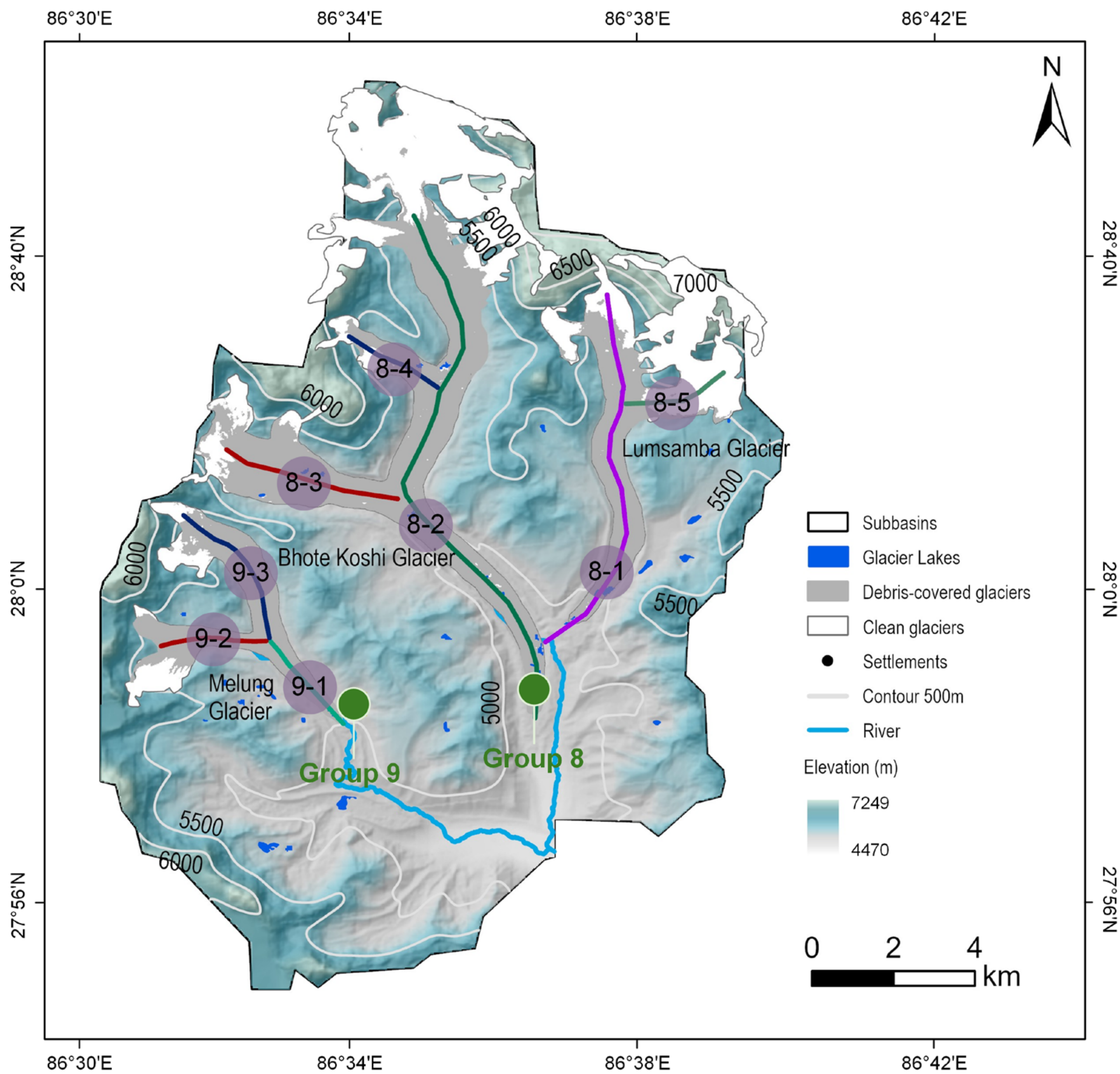

Supplementary Figure 12: Criteria for glacier system classification and spatial distribution of glaciers. Group 8-9 represents the spatial distribution of the glacier group, and the number corresponds to the glacier branch in Fig. 2. The correspondence is as follows: Group 8, Bhote Koshi Glacier (circled 8-2, 8-3 and 8-4) and Lumsamba Glacier (circled 8-1 and 8-5). Group 9, Melung Glacier (circled 9-1, 9-2 and 9-3)

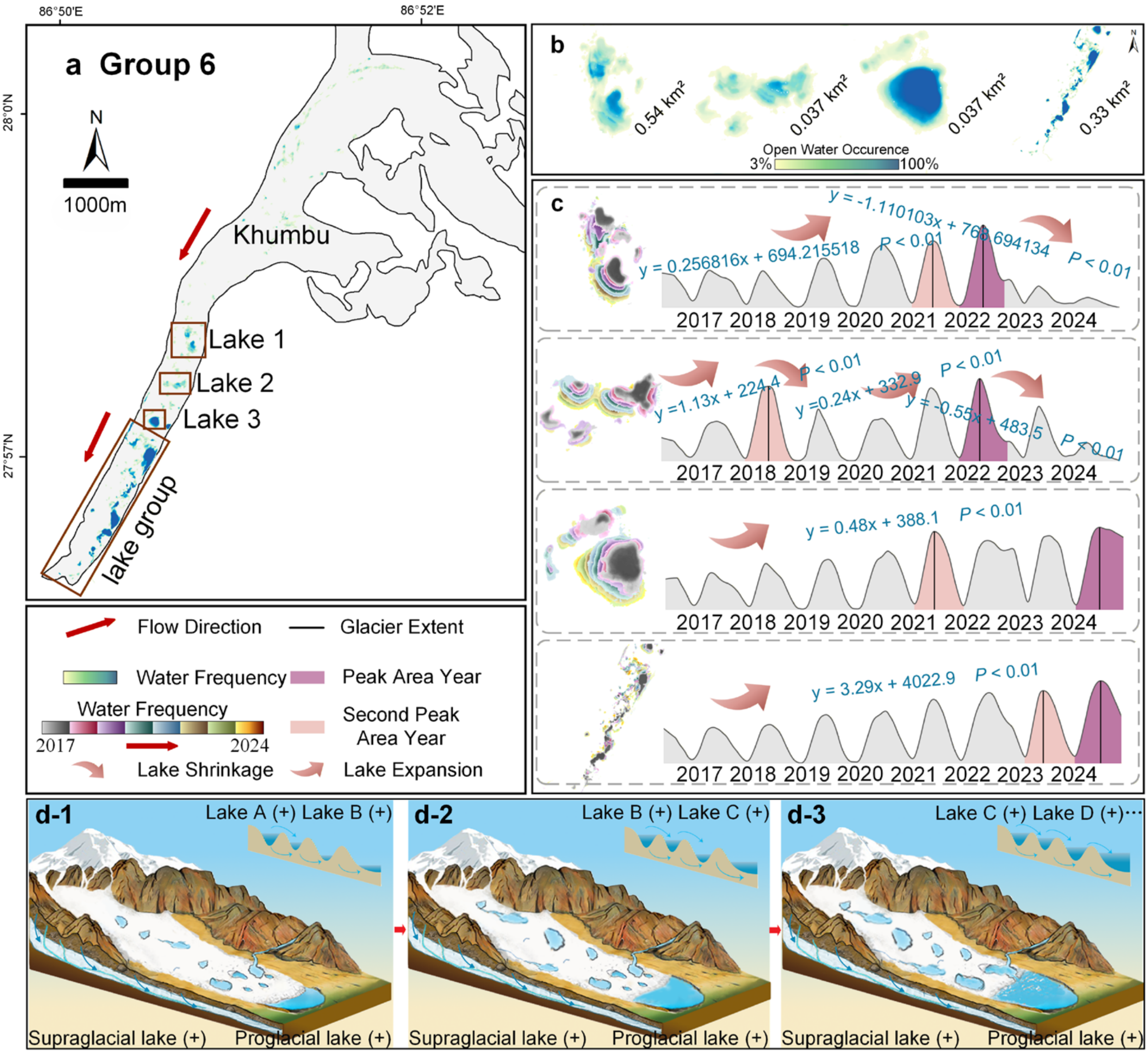

Supplementary Figure 13: Hydrological connectivity and cascading drainage among supraglacial lakes. a Frequency of lake occurrence in Glacier Group 6 from 2016 to 2024, with the four typical lakes highlighted. **b** Lakes are arranged from left to right corresponding to Lakes 1–4 in panel a, with the maximum area and occurrence frequency shown for each lake over the study period. **c** Annual frequency of supraglacial lakes and their cascading drainage processes, with locally weighted scatterplot smoothing fitted curves for lakes of interest. The x-axis of the ridgeline plot represents the time series of glacial lake area, with filled areas indicating the first and second peaks of lake area. The frequency plots on the left use different gradient colors to show the annual distribution of lakes, illustrating upstream lake incision and downstream proglacial lake aggradation. **d** a pattern of downstream lake expansion without upstream lake decline.

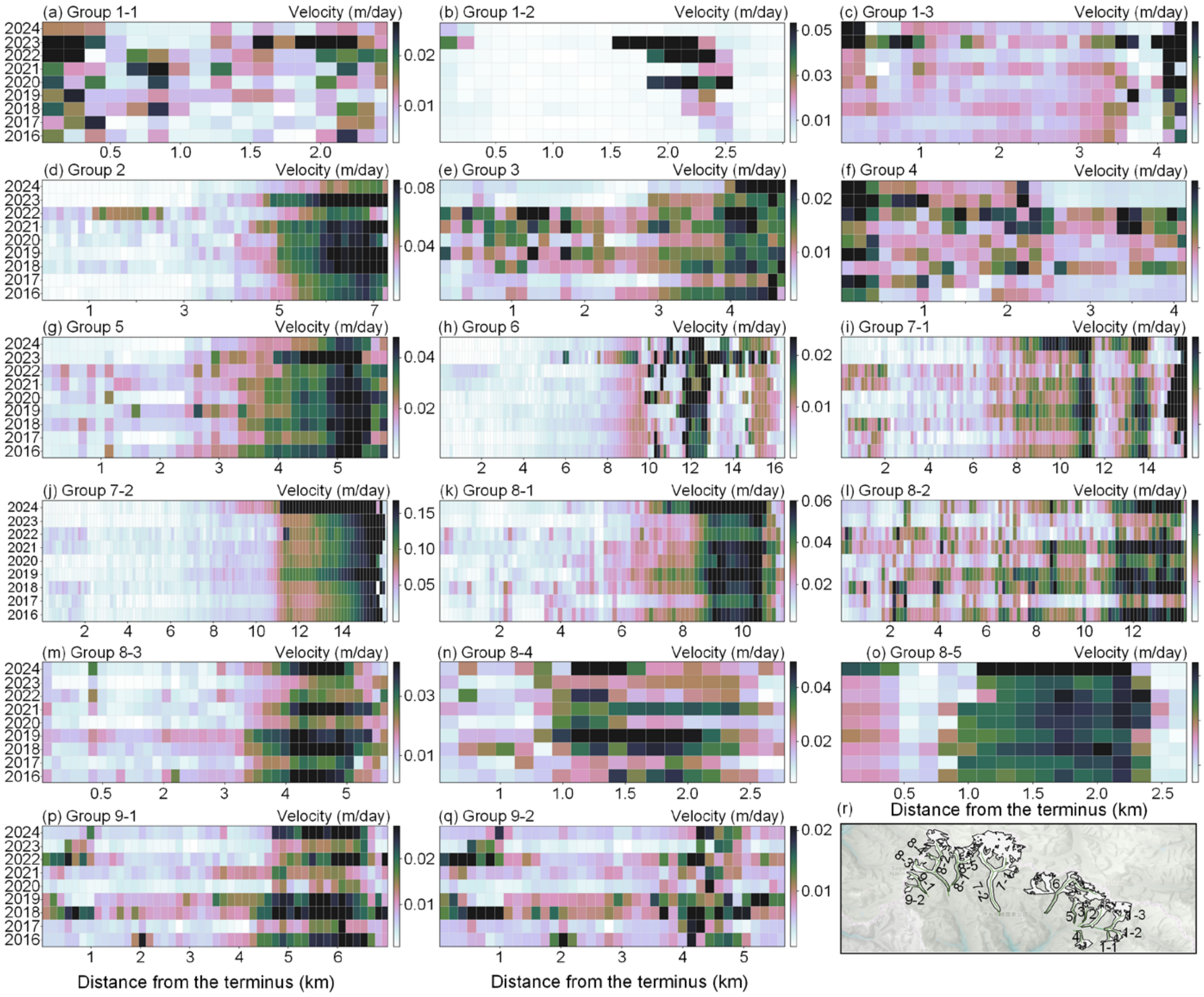

Supplementary Figure 14: Annual surface velocity of nine glacier groups from 2016 to 2024. a-c Group 1, Lhotse Shar、Imja and Ambulapcha; **d** Group 2, Lhotse; **e** Group 3, Lhotse Nup; **f** Group 4, Ama Dablam; **g** Group 5, Nuptse; **h** Group 6, Khumbu; **i-j** Group 7, Ngozumpa; **k-o** Group 8, Bhote Koshi and Lumsamba and **p-q** Group 9, Melung Glaciers. **a-q** Flow velocities of different glacier branches, where the number x (Group x) corresponds to the glacier with the same number in **r**. **r** Glacier numbers and their spatial distribution. Glacier flow velocity dates and image acquisition times are listed in Supplementary Table 6. The x-axis in the subfigure represents the distance from the centerline to the glacier terminus.

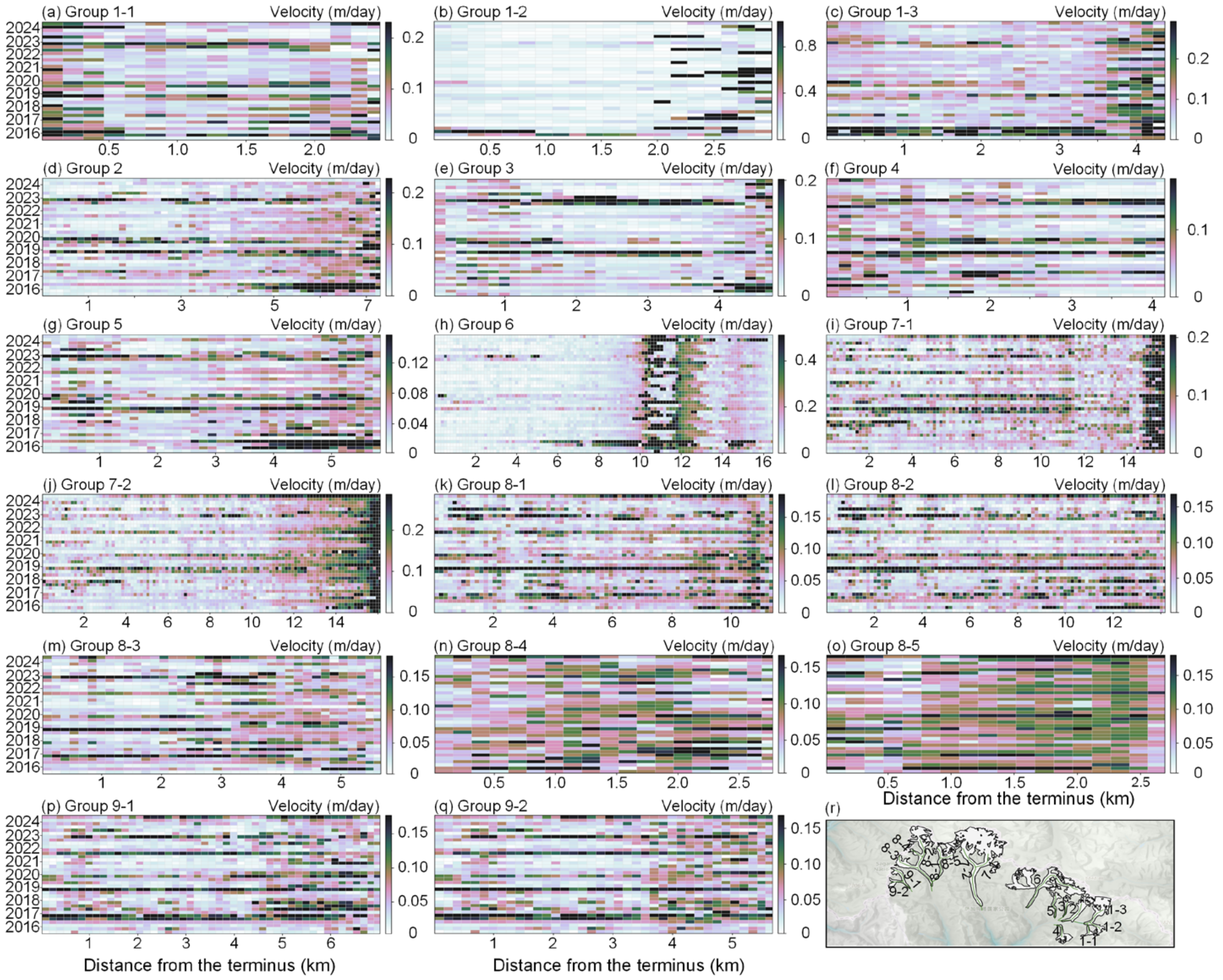

Supplementary Figure 15: Seasonal surface velocity of nine glacier groups from 2016 to 2024. a-c Group 1, Lhotse Shar、Imja and Ambulapcha; **d** Group 2, Lhotse; **e** Group 3, Lhotse Nup; **f** Group 4, Ama Dablam; **g** Group 5, Nuptse; **h** Group 6, Khumbu; **i-j** Group 7, Ngozumpa; **k-o** Group 8, Bhote Koshi and Lumsamba and **p-q** Group 9, Melung Glaciers. **a-q** Flow velocities of different glacier branches, where the number x (Group x) corresponds to the glacier with the same number in **r**. **r** Glacier numbers and their spatial distribution. Glacier flow velocity dates and image acquisition times are listed in Supplementary Table 7. The x-axis in the subfigure represents the distance from the centerline to the glacier terminus.

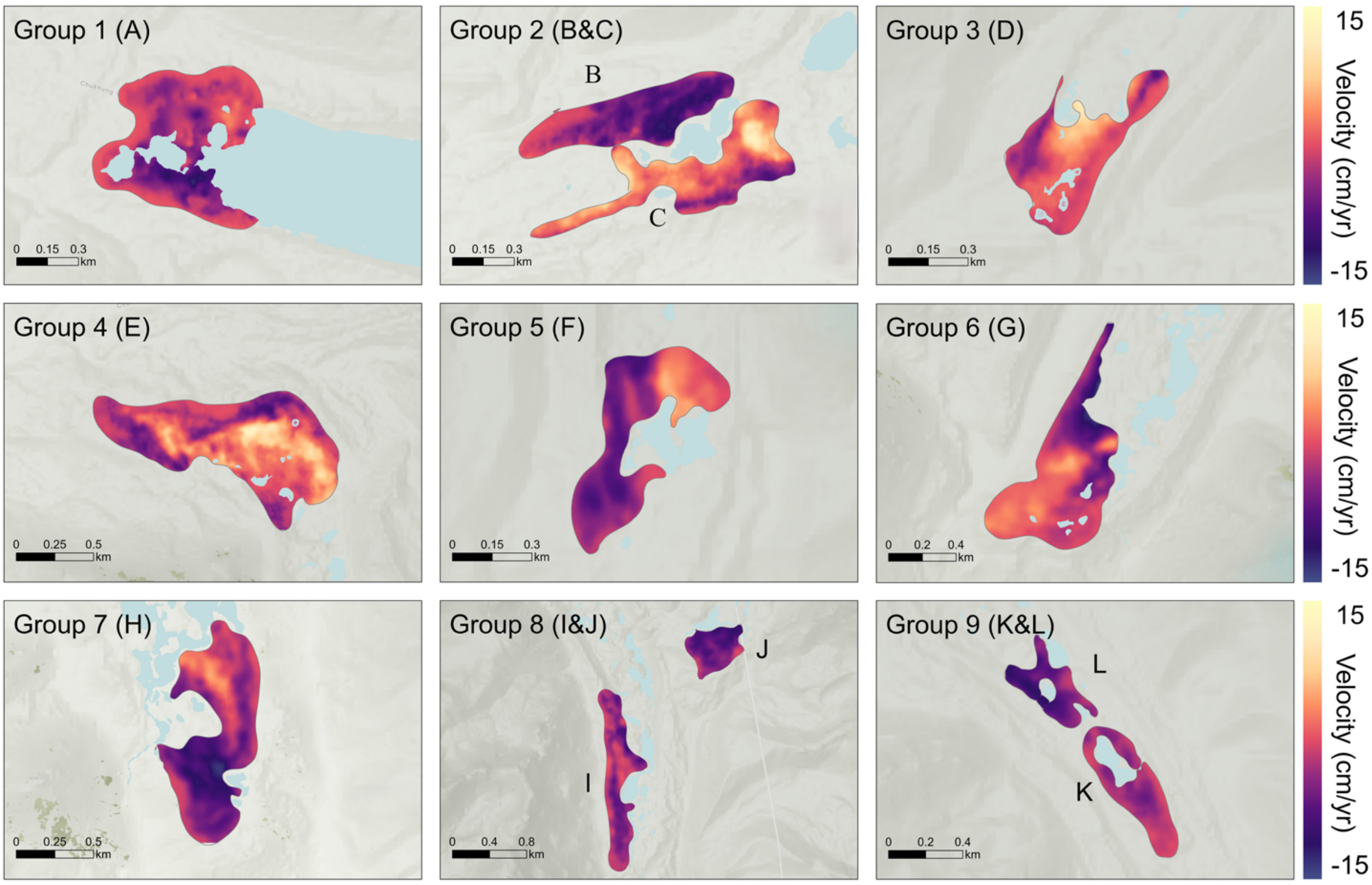

Supplementary Figure 16: Spatial distribution of moraine deformation at the glacier termini across nine glacier groups. Group 1, Lhotse Shar、Imja and Ambulapcha; Group 2, Lhotse; Group 3, Lhotse Nup; Group 4, Ama Dablam; Group 5, Nuptse; Group 6, Khumbu; Group 7, Ngozumpa; Group 8, Bhote Koshi and Lumsamba and Group 9, Melung Glaciers. Glacier Groups 1, 2, 5 and 7–9 contain proglacial lakes at their termini, whereas glacier Groups 3, 4 and 6 lack proglacial lakes. **A–L** represent study areas distributed across nine glacier groups.

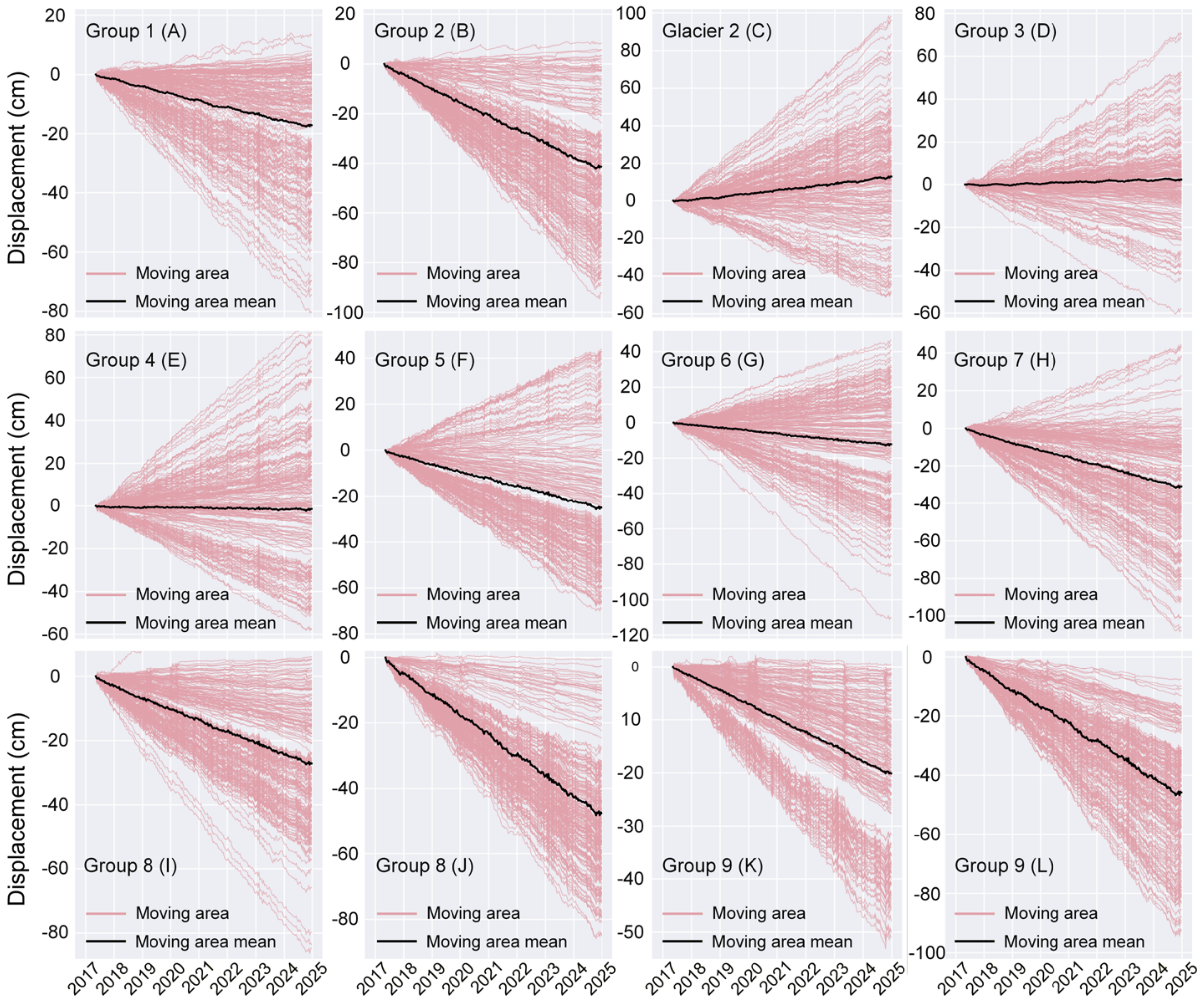

Supplementary Figure 17: Cumulative deformation time series of moraine deformation at the termini of nine glacier groups. Group 1, Lhotse Shar、Imja and Ambulapcha; Group 2, Lhotse; Group 3, Lhotse Nup; Group 4, Ama Dablam; Group 5, Nuptse; Group 6, Khumbu; Group 7, Ngozumpa; Group 8, Bhote Koshi and Lumsamba and Group 9, Melung Glaciers. Glacier Groups 1, 2, 5 and 7–9 contain proglacial lakes at their termini, whereas glacier Groups 3, 4 and 6 lack proglacial lakes. **A-L** correspond to the spatial distribution of study areas in Supplementary Figure 16.

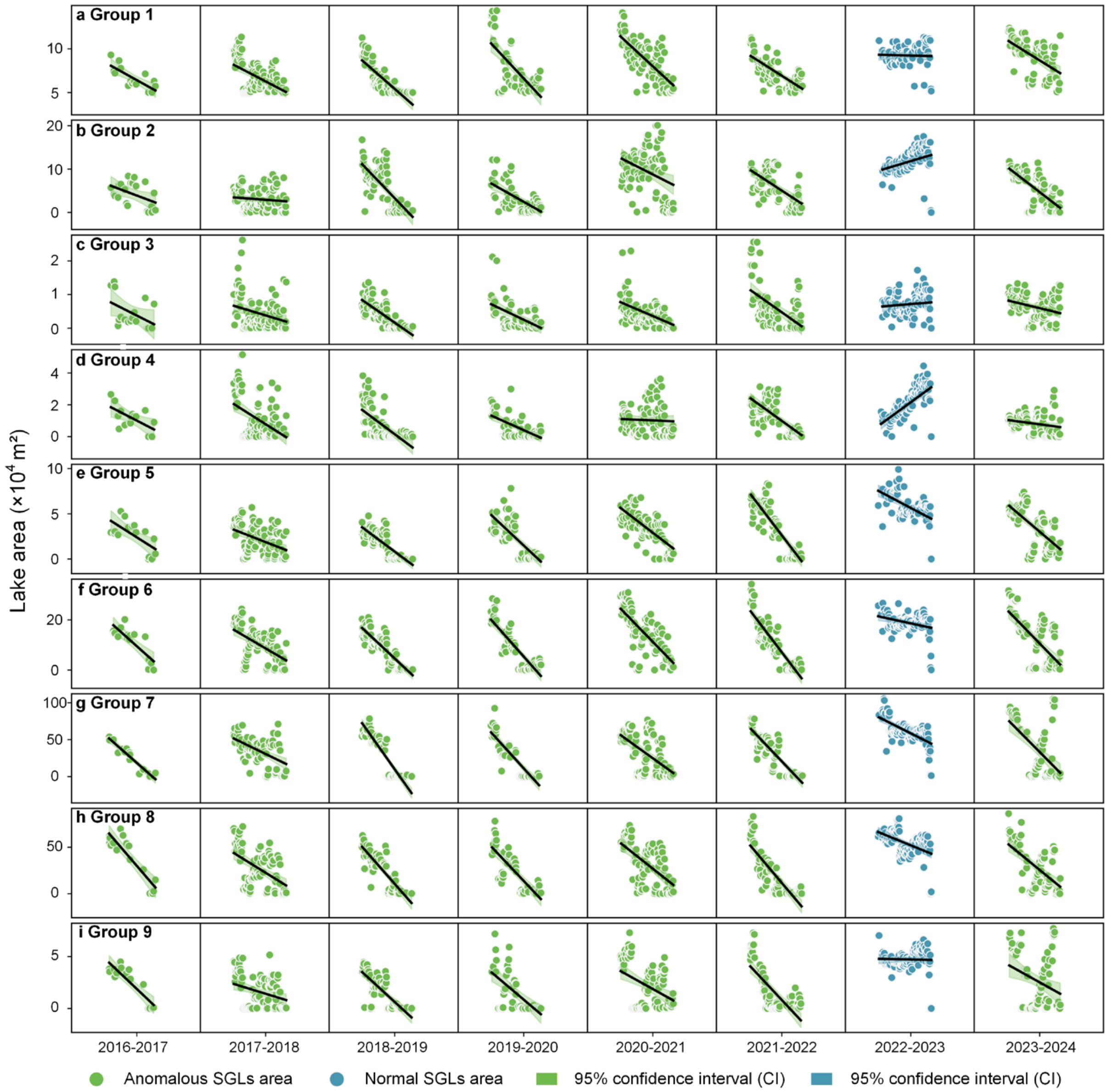

Supplementary Figure 18: Time series of supraglacial lake anomalies during the post-monsoon and winter seasons (October 1 – February 28). a Group 1, Lhotse Shar、Imja and Ambulapcha; **b** Group 2, Lhotse; **c** Group 3, Lhotse Nup; **d** Group 4, Ama Dablam; **e** Group 5, Nuptse; **f** Group 6, Khumbu; **g** Group 7, Ngozumpa; **h** Group 8, Bhote Koshi and Lumsamba and **i** Group 9, Melung Glaciers. The x-axis in the figure represents the daily time series of supraglacial lakes area from October 1 to February 28.

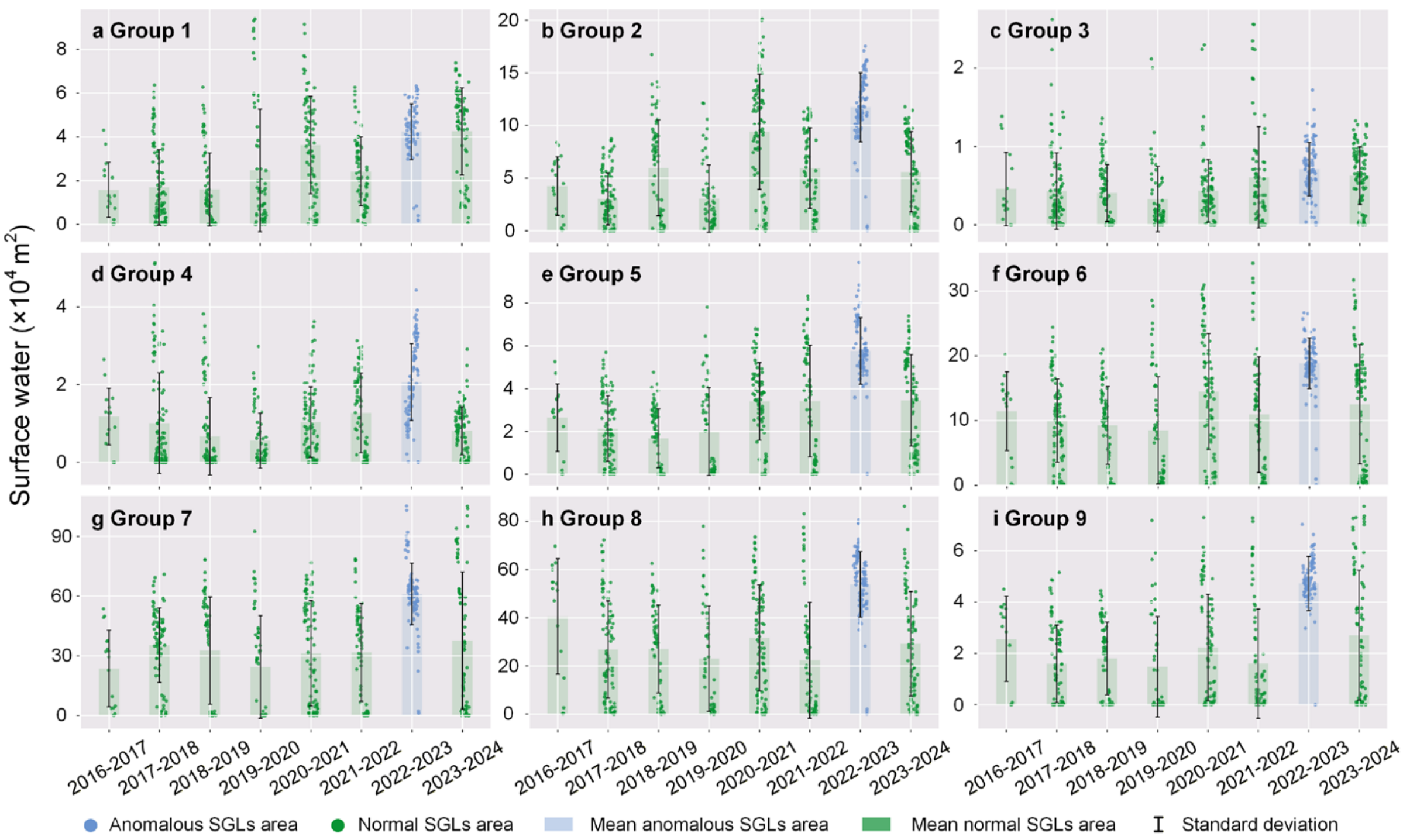

Supplementary Figure 19: Post-monsoon and winter lake freeze–thaw anomalies. **a** Group 1 Lhotse Shar、Imja and Ambulapcha; **b** Group 2, Lhotse; **c** Group 3, Lhotse Nup; **d** Group 4, Ama Dablam; **e** Group 5, Nuptse; **f** Group 6, Khumbu; **g** Group 7, Ngozumpa; **h** Group 8, Bhote Koshi and Lumsamba and **i** Group 9, Melung Glaciers. The x-axis in the figure represents the daily time series of supraglacial lakes area from October 1 to February 28.

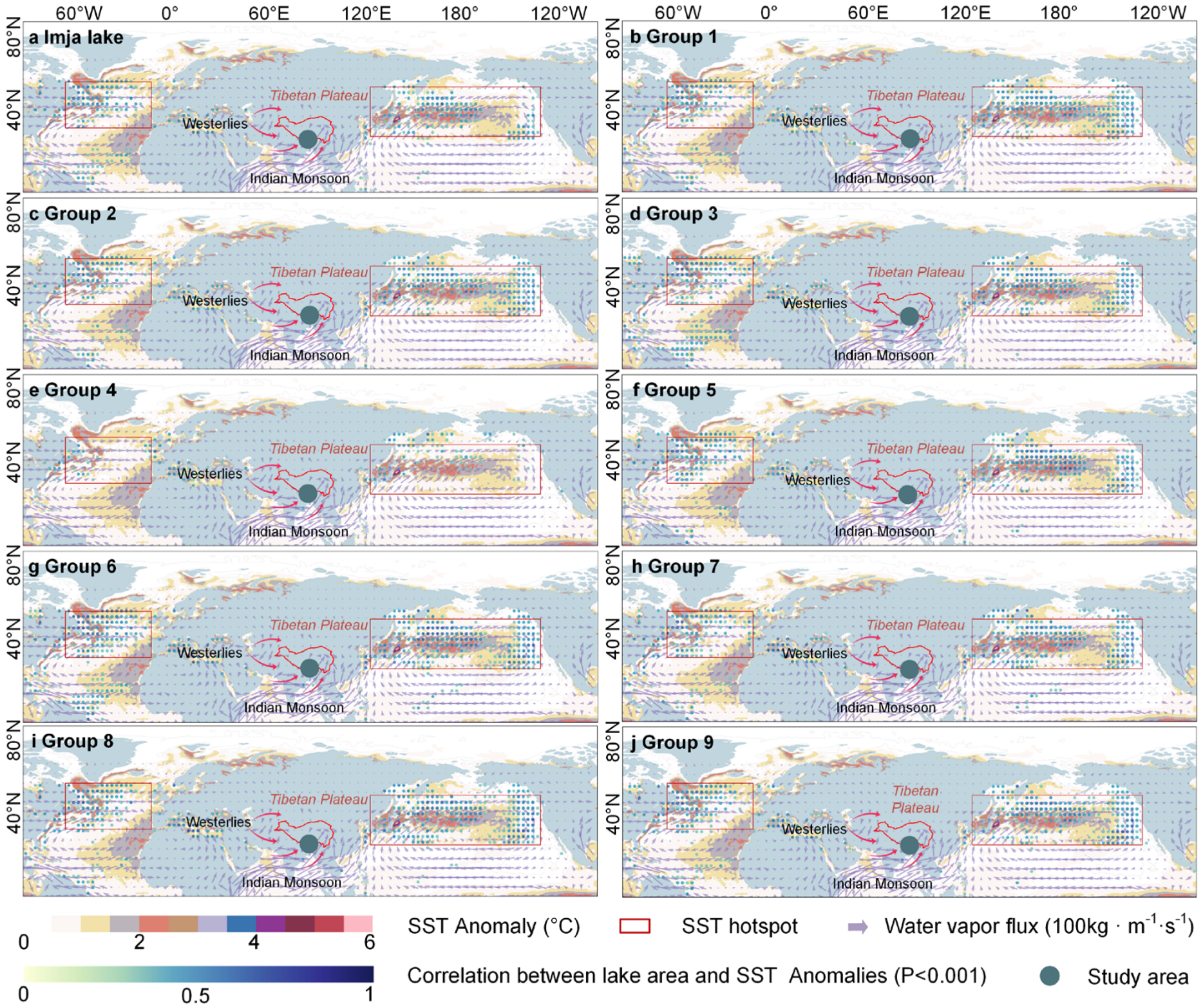

Supplementary Figure 20 Teleconnections of supraglacial lakes area with monsoonal moisture transport and SST anomalies. a Imja Lake; **b** Group 1, Lhotse Shar, Imja, and Ambulapcha; **c** Group 2, Lhotse; **d** Group 3, Lhotse Nup; **e** Group 4, Ama Dablam; **f** Group 5, Nuptse; **g** Group 6, Khumbu; **h** Group 7, Ngozumpa; **i** Group 8, Bhote Koshi and Lumsamba; **j** Group 9, Melung Glaciers. Background map showing the spatial distribution of 2023 sea surface temperatures (SST) anomaly peaks. Red arrows: monsoon influence on HMA glaciers (Adapted from reference[1]). Scatter points indicate regions where seasonal fluctuations of lake area exhibit lagged correlations with SST anomalies. Credit: arrow icon, Flaticon.com.

Supplementary Table 1 Overview of disaster events and sources

Number	Date	Type	Data source
1	1991-07-12	GLOF	Reference 2
2	1992-09-22	GLOF	Reference 2
3	1985-08-04	GLOF	Reference 2
4&5	2015-04-25	GLOF	Reference 2
6&7	2024-08-16	GLOF	News
8	1998-09-03	GLOF	Reference 2
9	1997-09-03	GLOF	Reference 2
10	2017-07-16	GLOF	Reference 2
11	1970-04-05	Avalanche	Wikipedia
12	2014-04-18	Avalanche	Wikipedia
13	2015-04-25	Avalanche	Wikipedia
14	2015-05-25	GLOF	Reference 2
15	2016-06-12	GLOF	Reference 2
16	2017-04-20	GLOF	Reference 2
others	1900-2024	serac collapses, avalanches, and blizzards	Wikipedia

Supplementary Table 2 Correspondence between Glacier Systems, Glacier Names, Glacier Branches, and Lake Numbers in Each Glacier Group

Glacier System	Glacier Name	Glacier Branch (Fig. 2)	Number of Lakes
Group 1	Ambulapcha	1-1	88
	Imja	1-2	
	Lhotse Shar	1-3	
Group 2	Lhotse	2	114
Group 3	Lhotse Nup	3	42
Group 4	Ama Dablam	4	42
Group 5	Nuptse	5	66
Group 6	Khumbu	6	144
Group 7	Ngozumpa	7-1	391
		7-2	
		7-3	
Group 8	Bhote Koshi	8-2	502
		8-3	
		8-4	
	Lumsamba	8-1	
		8-5	
Group 9	Melung	9-1	104
		9-2	
		9-3	

Glaciers sharing the same outlet were classified into the same group. The nine groups together contain 12 independent glaciers. Due to branching structures in some glaciers, a total of 19 glacier branch units were identified. The spatial correspondence between the identifiers and specific glacier branches is shown in Fig. 2a and Supplementary Figs. 9–S12.

Supplementary Table 3 Acceleration trends of nine supraglacial lakes

Glacier	Lhotse Shar、Imja and Ambulapcha	Lhotse	Lhotse Nup
Lake area ($m^2 \cdot yr^{-1}$)	3356.60 ± 627.86	5008.99 ± 1585.29	464.11 ± 145.94
Glacier	Ama Dablam	Nuptse	Khumbu
Lake area ($m^2 \cdot yr^{-1}$)	254.29 ± 430.38	2075.64 ± 942.63	6543.56 ± 3169.27
Glacier	Ngozumpa	Bhote Koshi and Lumsamba	Melung
Lake area ($m^2 \cdot yr^{-1}$)	14388.28 ± 9971.53	-5312.35 ± 13344.55	591.38 ± 1207.56

Supplementary 4 Mean and median ± IQR of annual glacier flow velocities for nine glacier groups.

Date	Average (m/yr)	Median ± IQR (m/yr)	Date	Average (m/yr)	Median ± IQR (m/yr)
**Ambulapcha**	8.5 ± 40.3	3.1 ± 3.5	20200113-20210107	3.2 ± 1.6	2.9 ± 1.4
20160114-20170118	3.2 ± 2.5	2.3 ± 2.3	20210107-20220127	4.4 ± 2.3	3.8 ± 3.7
20170118-20180123	3.2 ± 2.8	2.4 ± 2.0	20220127-20230107	5.1 ± 2.4	4.4 ± 3.0
20180123-20190118	3.9 ± 3.4	2.6 ± 2.7	20230107-20240112	4.1 ± 3.5	3.0 ± 4.4
20190118-20200113	3.8 ± 2.3	3.3 ± 1.7	20240112-20241227	3.0 ± 2.3	2.4 ± 2.3
20200113-20210107	3.9 ± 3.5	2.7 ± 3.1	**Nuptse**	3.9 ± 3.0	3.0 ± 3.5
20210107-20220127	5.2 ± 3.6	3.6 ± 3.4	20160114-20170118	4.5 ± 2.9	3.4 ± 4.6
20220127-20230107	4.9 ± 3.3	3.9 ± 3.7	20170118-20180123	2.8 ± 2.3	2.1 ± 2.1
20230107-20240112	46.0 ± 114.7	6.3 ± 8.4	20180123-20190118	6.3 ± 3.5	5.3 ± 4.7
20240112-20241227	3.0 ± 1.9	2.5 ± 2.1	20190118-20200113	5.2 ± 2.6	4.7 ± 4.2
**Imja**	32.3 ± 84.8	5.8 ± 8.6	20200113-20210107	2.2 ± 1.7	1.5 ± 1.6
20160114-20170118	8.1 ± 11.4	4.8 ± 6.0	20210107-20220127	3.6 ± 1.6	3.2 ± 2.3
20170118-20180123	10.1 ± 17.2	4.3 ± 7.1	20220127-20230107	4.4 ± 3.3	3.2 ± 3.7
20180123-20190118	16.0 ± 21.0	7.7 ± 9.9	20230107-20240112	2.5 ± 2.3	1.4 ± 3.3
20190118-20200113	13.0 ± 32.1	4.1 ± 4.5	20240112-20241227	4.0 ± 3.2	2.3 ± 3.7
20200113-20210107	53.3 ± 108.2	5.1 ± 14.7	**Khumbu**	25.3 ± 39.3	13.0 ± 23.5
20210107-20220127	14.7 ± 22.2	5.8 ± 7.1	20160114-20170118	20.9 ± 24.1	13.0 ± 20.7
20220127-20230107	68.6 ± 130.3	5.0 ± 10.5	20170118-20180123	21.1 ± 30.0	12.0 ± 23.1
20230107-20240112	99.1 ± 157.2	13.8 ± 160.6	20180123-20190118	21.7 ± 22.0	15.4 ± 22.6
20240112-20241227	7.8 ± 10.1	3.8 ± 6.3	20190118-20200113	20.5 ± 22.7	12.5 ± 17.6
**Lhotse Shar**	17.1 ± 35.5	12.6 ± 7.1	20200113-20210107	27.7 ± 44.1	11.3 ± 27.9
20160114-20170118	12.9 ± 5.3	13.2 ± 4.0	20210107-20220127	22.0 ± 25.4	13.6 ± 15.6
20170118-20180123	11.7 ± 5.6	11.0 ± 4.1	20220127-20230107	29.9 ± 58.6	13.1 ± 19.7
20180123-20190118	15.0 ± 5.6	15.1 ± 5.2	20230107-20240112	41.9 ± 60.8	17.4 ± 53.0
20190118-20200113	14.4 ± 9.3	12.7 ± 3.2	20240112-20241227	22.2 ± 37.6	10.4 ± 22.0
20200113-20210107	14.4 ± 11.4	11.6 ± 7.2	**Ngozumpa**	19.6 ± 23.8	11.2 ± 21.9
20210107-20220127	15.4 ± 8.4	16.6 ± 8.8	20160114-20170118	17.5 ± 19.9	11.4 ± 20.4
20220127-20230107	15.3 ± 31.0	9.6 ± 6.7	20170118-20180123	14.7 ± 15.3	8.9 ± 15.9
20230107-20240112	42.9 ± 96.5	16.7 ± 20.0	20180123-20190118	18.8 ± 24.2	13.0 ± 18.1
20240112-20241227	12.7 ± 9.9	10.6 ± 6.5	20190118-20200113	23.1 ± 25.7	13.9 ± 32.8
**Lhotse**	11.6 ± 14.5	7.0 ± 16.6	20200113-20210107	18.7 ± 20.8	10.3 ± 25.7
20160114-20170118	9.4 ± 9.0	5.3 ± 15.2	20210107-20220127	20.8 ± 18.9	13.6 ± 21.4
20170118-20180123	8.9 ± 9.4	3.0 ± 15.4	20220127-20230107	17.9 ± 22.0	9.7 ± 14.0
20180123-20190118	12.6 ± 10.8	7.0 ± 17.0	20230107-20240112	17.2 ± 26.3	8.5 ± 21.3
20190118-20200113	15.3 ± 28.2	7.0 ± 20.5	20240112-20241227	27.6 ± 33.9	12.7 ± 29.9
20200113-20210107	11.2 ± 10.0	7.2 ± 18.4	**Bhote Koshi Lumsamba**	8.2 ± 8.2	5.5 ± 7.1
20210107-20220127	14.6 ± 14.1	9.1 ± 21.0	20160114-20170118	8.9 ± 7.0	6.7 ± 6.9
20220127-20230107	11.5 ± 6.3	11.0 ± 11.0	20170118-20180123	6.4 ± 7.2	3.7 ± 5.8

20230107-20240112	14.5 ± 18.8	5.5 ± 24.9
20240112-20241227	6.4 ± 6.2	3.0 ± 8.8
Lhotse Nup	4.2 ± 4.4	3.5 ± 3.7
20160114-20170118	4.0 ± 2.4	3.5 ± 4.1
20170118-20180123	2.6 ± 2.1	1.9 ± 1.9
20180123-20190118	4.5 ± 2.2	4.1 ± 2.6
20190118-20200113	5.1 ± 2.4	4.8 ± 2.0
20200113-20210107	3.6 ± 2.3	3.1 ± 3.0
20210107-20220127	5.9 ± 2.7	5.4 ± 3.1
20220127-20230107	5.4 ± 3.0	4.5 ± 3.1
20230107-20240112	4.0 ± 10.5	1.3 ± 3.1
20240112-20241227	2.6 ± 3.4	1.5 ± 0.7
Ama Dablam	3.6 ± 2.4	3.0 ± 2.6
20160114-20170118	2.4 ± 1.5	1.9 ± 1.4
20170118-20180123	3.1 ± 2.0	2.2 ± 1.9
20180123-20190118	3.8 ± 1.7	3.6 ± 2.6
20190118-20200113	3.5 ± 2.1	2.7 ± 1.8
20180123-20190118	9.4 ± 7.3	7.5 ± 7.4
20190118-20200113	9.4 ± 7.2	6.7 ± 7.5
20200113-20210107	7.2 ± 7.4	4.8 ± 5.5
20210107-20220127	9.6 ± 8.0	7.0 ± 7.7
20220127-20230107	6.5 ± 6.5	4.4 ± 5.1
20230107-20240112	6.0 ± 7.1	3.4 ± 5.6
20240112-20241227	10.0 ± 12.9	5.0 ± 8.6
Melung	3.9 ± 3.0	3.0 ± 3.5
20160114-20170118	4.5 ± 2.9	3.4 ± 4.6
20170118-20180123	2.8 ± 2.3	2.1 ± 2.1
20180123-20190118	6.3 ± 3.5	5.3 ± 4.7
20190118-20200113	5.2 ± 2.6	4.7 ± 4.2
20200113-20210107	2.2 ± 1.7	1.5 ± 1.6
20210107-20220127	3.6 ± 1.6	3.2 ± 2.3
20220127-20230107	4.4 ± 3.3	3.2 ± 3.7
20230107-20240112	2.5 ± 2.3	1.4 ± 3.3
20240112-20241227	4.0 ± 3.2	2.3 ± 3.7

Supplementary Table 5 Number and temporal distribution of planet satellite images

Year	Number	Year	Number	Year	Number
2016	162	2019	1601	2022	1223
2017	2004	2020	1409	2023	1304
2018	2566	2021	1613	2024	1253

Supplementary Table 6 Annual glacier velocity image pairs

Image	Date	Date	Date
Reference Image	2016-01-14	2017-01-18	2018-01-23
Secondary Image	2017-01-18	2018-01-23	2019-01-18
Reference Image	2019-01-08	2020-01-13	2021-01-07
Secondary Image	2020-01-13	2021-01-07	2022-01-27
Reference Image	2022-01-27	2023-01-07	2024-01-12
Secondary Image	2023-01-07	2024-01-12	2024-12-27

Supplementary Table 7 Seasonal glacier velocity image pairs

Image	Date	Date	Date
Reference Image	2016-01-14	2016-04-13	2016-05-03
Secondary Image	2016-04-13	2016-05-03	2016-10-30
Reference Image	2016-10-30	2016-12-29	2017-03-29
Secondary Image	2016-12-29	2017-03-29	2017-05-08
Reference Image	2017-05-08	2017-10-30	2017-12-29
Secondary Image	2017-10-30	2017-12-29	2018-03-19
Reference Image	2018-03-19	2018-05-08	2018-09-25
Secondary Image	2018-05-08	2018-09-25	2018-12-29
Reference Image	2018-12-29	2019-03-29	2019-05-08
Secondary Image	2019-03-29	2019-05-08	2019-10-15
Reference Image	2019-10-15	2019-12-24	2020-03-08
Secondary Image	2019-12-24	2020-03-08	2020-05-17
Reference Image	2020-05-17	2020-08-30	2020-12-28
Secondary Image	2020-08-30	2020-12-28	2021-03-03
Reference Image	2021-03-03	2021-05-22	2021-09-19
Secondary Image	2021-05-22	2021-09-19	2021-12-23
Reference Image	2021-12-23	2022-03-08	2022-07-16
Secondary Image	2022-03-08	2022-07-16	2022-10-24
Reference Image	2022-10-24	2022-12-23	2023-03-28
Secondary Image	2022-12-23	2023-03-28	2023-05-22
Reference Image	2023-05-22	2023-08-30	2023-12-03
Secondary Image	2023-08-30	2023-12-03	2024-03-17
Reference Image	2024-03-17	2024-06-10	2024-10-28
Secondary Image	2024-06-10	2024-10-28	2024-12-27

References

1. Yao, T.D. et al. Different glacier status with atmospheric circulations in Tibetan Plateau and surroundings. *Nature Climate Change* **2,** 663-667 (2012).
2. Veh, G. *et al.* Less extreme and earlier outbursts of ice-dammed lakes since 1900. *Nature* **614**, 701-707 (2023).
