# Supplementary material for: Unraveling daily dynamics of supraglacial lakes in response to hydrological pulses and anomalous climate signals
Source: Nat Commun. 2026 Jul 17;17:7930. doi: 10.1038/s41467-026-75669-3 (PMC13448815; doi:10.1038/s41467-026-75669-3)

nature portfolio

Peer Review File

Unraveling daily dynamics of supraglacial lakes in response to hydrological pulses and anomalous climate signals

Corresponding Author: Professor Haijun Qiu

Parts of this Peer Review File have been redacted as indicated to remove third-party material.

This file contains all reviewer reports in order by version, followed by all author rebuttals in order by version.

Version 0:

Reviewer comments:

Reviewer #1

(Remarks to the Author)
This paper presents a comprehensive and thorough study of the long-term hydrological dynamics of glacial lakes, glacier flow changes, lake-margin deformations, and the precursor effects of small lakes under the context of global warming. It provides an in-depth understanding of the role of small lakes in nature. The research is novel, the workload is substantial, the results are well presented, and the interpretations and conclusions are clear. However, some aspects still require revision and improvement. Therefore, after addressing the suggested modifications below, I recommend this paper for publication.

Summary of the manuscript
The authors used a large amount of remote sensing imagery from high-mountain regions in Asia to observe the dynamic fluctuations of glacial lakes and their responses to global warming. By leveraging the daily revisit capability and high spatial resolution of Planet imagery, they were able to monitor the daily variations of the lakes with fine detail, which is a very interesting and time-consuming task. Observations indicate that, with global warming, the seasonal variability of glacial lakes and their lifecycle evolution exhibit significant dynamism. According to the authors, among the 1,493 lakes they monitored, only 98 were able to persist long-term, while 928 had lifecycles shorter than three years; they referred to this phenomenon as the hydrological pulsation of lakes. Based on these observations, they found that the daily fluctuations in lake area in the high-mountain regions of Asia could serve as early anomalous signals of global temperature changes. Meanwhile, the authors used optical imagery to construct annual and seasonal glacier flow velocity datasets and linked these with the daily dynamics of the glacial lakes, finding that increases in glacier flow velocity often coincide with dynamic changes in the lakes. Additionally, Sentinel-1 radar data were employed to monitor the relationship between the presence of terminal lakes and surface deformation in the region. The authors focused on analyzing the anomalous fluctuations of glacial lakes during 2022–2023 under the context of global warming, and through comparisons with previous years, they found that high-mountain lakes are able to respond to temperature anomalies earlier than lakes in other regions as temperatures rise.

Assessment of the manuscript
This paper is very comprehensive, which is in large part due to the extensive dataset, and I commend the authors for completing this impressive and massive amount of work. According to the authors, the daily fluctuation dataset of glacial lakes relies primarily on visual interpretation, with a total of 13,135 images. In my view, this is an extremely large and time-consuming task. I wonder why the authors did not employ the latest research techniques, such as deep learning or existing water monitoring methods (e.g., A Glacial Lake Mapping Framework in High Mountain Areas: A Case Study of the Southeastern Tibetan Plateau; Automatic Extraction of Glacial Lakes from Landsat Imagery Using Deep Learning across the Third Pole Region; Surface Water Mapping by Deep Learning). Currently, most studies on glacial lakes focus on annual changes, with only a few addressing seasonal-scale variations. In my opinion, the authors' study of glacial lakes at a daily timescale is very novel. Therefore, could this approach be extended to broader regions, such as the high-latitude Arctic, the Andes, or Alaska? This would be highly valuable for future related research.
This study primarily focuses on small lakes (indeed, understanding their dynamics addresses a clear research gap, and this study provides unprecedented detailed insights). However, do the outbursts of larger lakes often cause greater hazards? I acknowledge that recent studies suggest this is not necessarily the case. Nevertheless, the lakes examined in this study are much smaller than those involved in current glacial lake outburst cases. Although the authors also focus on lake drainage and lifecycle issues (Figs. 3 & 4), the discussion should emphasize the role of these lakes in natural environments and mountain hazards, as this is one of the core aspects of the paper. Please refer to Flood Risk from Small Mountain Lakes.

After these points, I would like to emphasize that the authors' efforts on small glacial lakes are valuable. These data are novel and provide new insights and reference cases for discussing mountain hazards in the study region as well as trends in global glacial lakes. However, I suggest that the authors could expand the discussion to highlight the broader significance of this study for related global research. Moreover, the spatial heterogeneity of glacial lake distribution varies across different dimensions and regions. The authors could, by drawing on other studies, appropriately compare the characteristics of glacial lakes in regions such as the Tibetan Plateau, the Andes, Alaska, and Greenland, thereby emphasizing the distinctiveness of their research. Additionally, the authors could use this study as a basis to outline prospects for future related research (I am particularly interested in the application of this study to short-term variations of glacial lakes globally), which would be highly valuable. The following are some specific comments and suggestions.
Minor comments/ questions:
Abstract
L32: In your other regions, you refer to supraglacial lakes, but here you wrote glacial lake. Are you trying to emphasize that you are studying a specific type of glacial lake, or is this a mistake? Please check.
Results
L132: Delete "ponds" from "From 2016 to 2024, we detected 1,493 supraglacial lakes and ponds" and check the entire manuscript for consistency.
L162: "Glacial lake" or "supraglacial lakes"?
L202: No. 9 Glacier? Does this glacier not have a name? I noticed that the others all have names—please confirm.
Discussion
L331-337: Please add references in the appropriate places.
L338-339: Please include the table of disaster information from your appendix here.
L338-362: In this section, you mention glacial lakes in the specific study area and glacial lake outbursts in other regions. You could briefly highlight some characteristics of small glacial lakes in other regions, keeping the discussion concise, but try to provide a comparative analysis and emphasize the distinct features of your study area.
L379-381: Please add references to support this conclusion.
L401: At the end of the discussion, briefly highlight the limitations of your study and outline future prospects and points of interest, keeping it concise and insightful.
Methods
L404: Please create a figure and include it as an appendix to show the specific acquisition dates of the imagery you used.
L432: Why is there an emphasis on 0.5 Landsat pixels here? Your manuscript does not seem to use Landsat imagery.
Figures
Fig.1: Compared with 4 & 5, the font size of 7 & 8 is smaller; please adjust them to be the same size.
Fig.2: Very beautiful and informative, but what does the size of the circles in subplots a and b represent-lake size? If so, please indicate this in the title.
Fig.3: Like Fig. 2, it is very beautiful and informative, but some of the tick labels on the left are centered while others are left-aligned. Please make them consistent.
Fig.4: The schematic at the bottom is very nice, but the arrows are too thin and a bit hard to see. Please adjust them appropriately to better convey your meaning. Also, your figure has a scale bar in the top right corner, while the other subfigures do not; please make them consistent.
Fig.5: Indicate the location of your study area in the figure.
Extended Data Fig. 1&2: The same problem applies to Fig. 4.
Extended Data Fig. 7&8: The font sizes of the x-axis labels in the two figures are quite different. Please adjust them appropriately for better visual consistency.

Reviewer #2

(Remarks to the Author)
This study presents an analysis of supraglacial lake changes in the Sagarmatha National Park at a high temporal resolution, linking observations to glacier changes (velocity, surface changes). It is suggested that increasing lake sizes since 2022 demonstrates that the lakes respond to climate change variability ahead of this being recorded by sea surface temperatures, and thus lake changes could provide important early warning indicators of climate changes. This therefore represents an important topic of study and the dataset should be of value to the community. However, there are quite a few places where I struggled to follow the overall way this study has been written up, presenting a barrier to these potentially useful results being accessible to the community. The figures are also hard to follow or seemingly lacking important details. I think these substantial issues will need to be considered by the author team.

General comments:

The paper title indicates that this study is about supraglacial lakes. However, in places, e.g. Figure 4, it appears that it is the connectivity between supraglacial lakes and proglacial lakes that is important – but this is not really explained fully in the introduction. The introduction is largely focused on glacial lakes in general, with no clear distinction made between different types, e.g. moraine-dammed, ice-dammed, proglacial, supraglacial. Whilst all glacial lakes are of course extremely important, for the reasons outlined, I think the explanation of the focus on supraglacial lakes, and their connection to proglacial lakes (if that is important here) needs to come sooner. Clearer use of terminology and a greater focus on supraglacial lakes as the subject of the paper would improve the introduction.

On a similar note, there are a lot of references to "small glacial lakes" in the paper. I am assuming from the paper title that these are "small supraglacial lakes", so this should be explicitly stated throughout to avoid any possible confusion.

Surface deformation is used throughout the paper, but I am not sure it is the best term (although it is not easy to understand exactly what it is referring to). Is this simply downglacier surface movement as the ice flows? Or is there a vertical element to this? In some places this refers to moraine ridges deformation. A clearer explanation of this term is needed, or perhaps better still, use of another term that is less ambiguous.

A better location map is needed to clearly show the spatial context of the study area glaciers and lakes – Figure 1 does not do this.

Throughout the paper and figures, the labelling of glaciers, glacier systems, the mix of glacier numbers/glaciers systems numbers/glacier names – this is all very confusing and could be much more straightforward.

Specific comments:

Abstract

Line 33: It would be useful to state where in HMA the Sagarmatha region is – perhaps using the Hindu Kush Himalayan Monitoring and Assessment Programme (HIMAP) sub-regions: https://link.springer.com/chapter/10.1007/978-3-319-92288-1_7

Lines 42-43: "The presence of proglacial lakes governs the surface deformation rate at glacier termini, with mean cumulative displacement along the radar line-of-sight up to 46.84 cm." – this sentence seems out of place here? Before now the focus has been on supraglacial lakes, so the introduction of proglacial lakes here seems odd. This should be integrated into the text more clearly or removed.

Lines 43-44: "In High Mountain Asia, the expansion of supraglacial lakes is expected to continue." – why, and on what timescale is this suggestion being made?

Lines 44-46: "…they can be incorporated into the global early-warning system for hydrological pulses and extreme climate" – is this early-warning system something that already exists, and is it to detect both hydrological pulses and extreme climate? I think there could be more clarity here.

Introduction

Line 53: "glacier collapses" – suggest "collapses or detachments".

Line 54: add citations to global studies of glacier collapses/detachments and GLOFs here: for example, Kääb et al. (2021: https://doi.org/10.5194/tc-15-1751-2021) and Taylor et al. (2023: https://www.nature.com/articles/s41467-023-36033-x)

Lines 58-59: "In recent years, advances in optical and radar remote sensing have accelerated investigations into seasonal to interannual variations in glacier flow and glacial lake dynamics" – add citations to one or two example studies here.

Line 78: Suggest adding "supraglacial ponds" so it is unambiguous.

Lines 79: "near-surface and shallow subsurface drainage pathways" – does this mean supraglacial and englacial drainage pathways? It is not clear what the difference between near-surface and shallow subsurface is here, so the use of clearer terminology would help.

Lines 82-90: "Glacial retreat has lead to…" to "…reservoirs and dams." – these sentences seem somewhat tangential to the focus of this paper on supraglacial lakes. As noted elsewhere, I think the introduction needs to get to the focus of the paper on supraglacial lakes on debris-covered glaciers much quicker – why are these important to study?

Lines 90-91: "Another important factor is the limitation of satellite observations, which do not account for the uncertainties associated with the drainage of small lakes and debris flow hazards." – this needs to be explained more clearly. Why is this? What is the link between small lakes and debris flow hazards?

Line 92: "feature glacial lakes" – state that these are supraglacial lakes for clarity.

Lines 92-93: The link between these two sentences is not clear because the link between supraglacial lakes and GLOFs has not really been clearly articulated to this point. See elsewhere for comments on the need for a clearer focus in the introduction on supraglacial lakes, particularly small supraglacial ponds on debris-covered glaciers, and GLOF hazards.

Lines 95-99: The sentences here need rephrasing. Suggest adding key citations to studies on the listed hazards.

Line 99: "Bridges and houses have frequently been damaged." – add citation to studies/examples of this.

Lines 100-101: "…small glacial lakes have increasingly become the primary source of GLOFs, with dozens of occurrences" – are these small supraglacial lakes or a mix of lake types? Given the focus of the paper, it is important to clarify.

Lines 101-103: "Such frequent and hard-to-monitor geohazards are rarely included in global compilations of glacial lake outburst events" – can you give an indication here of how frequent? Why are they hard to monitor?

Line 103: what are "global lakes"?#

Lines 104-105: "…seasonally dominated lakes comprising approximately 66% of the total global lake area" –there should be a citation here to the source of the reported data.

Lines 105-108: "Seasonal fluctuations in lake area are considered one of the most sensitive characteristics of small glacial lakes. However, they are often overlooked due to the trade-off between spatial and temporal resolution in single-sensor satellite data30. Therefore, urgent investigations into the seasonal dynamics of small glacial lakes and their implications for mountain hazard management are needed" – references here to "small glacial lakes": are you referring here to supraglacial lakes? This should be stated.

Line 109: "glacial lake" – should this be "supraglacial lake"?

Lines 110-111: "surface deformations" – what is meant by this? It could have several meanings so it would be best to be clear. Is this ice surface or other?

Line 111: "SST" not explained yet.

Line 113: what are "anomalous lake events"? – this could have several meanings so please provide clarity.

Lines 113-114: "Glacier velocity was included in the framework regulating glacial lake expansion.." – I don't follow this, more clarity needed.

Line 116: "small glacier lake" – small supraglacial lakes?

Results

Lines 129-130: "In recent decades, the rapid expansion of supraglacial lakes has attracted considerable attention, though most studies have focused on lakes larger than 0.05 km², largely overlooking small lakes" – this sentence seems like it should be in the introduction, not results.

Lines 130-132: "The high spatiotemporal resolution and frequent revisit of Planet imagery allow analysis of individual lake filling and drainage events." – this should be in the methods.

Line 133: "nine glacier groups" – how many individual glaciers? Is it the 19 shown in Fig. 2? "with 42–502 lakes per group" – how many per glacier?

Lines 133-134: "Only 98 lakes were perennial" – suggest adding that this is for the duration of the study period

Line 136: "Under global warming.." – this isn't required here, delete.

Lines 132-136: The constant change in unit order of magnitude makes it hard to gauge the differences between lake sizes, e.g. 10^{-4} km², km², 10^{-2} km², 10^{-3} km² on four different lines. Figure 2 then uses a fixed 103 km². Please use the same unit of measurement throughout.

Line 139: what are the "peaks of contracting lakes"? This seems counterintuitive.

Line 140: What is "displacement" in this context? Horizontal movement of ice? And other what timescale is this displacement measured? I have read the section in the methods on this and I am no clearer.

Lines 141-142: "At the terminus of No. 9 Glacier, the cumulative displacement of terminus lakes reached 1,574 m, resulting from the merger of two terminus lakes, while the maximum displacement of lakes at other glaciers ranged between 114 and 621 m." – I don't understand what this is measuring, this needs to be clearly explained.

Lines 144-145: "…Imja Lake primarily expanded within ~2 km of the terminus, where surface slopes are as low as 2°" – can you clarify if this is a supraglacial lake? Within ~2 km of the terminus – is this referring to upglacier of the terminus?

Line 146: is "Imja Tsho" the same as "Imja Lake"?

Lines 146-147: "…although Lhotse and No. 9 glaciers have substantial water supply, they lack riverbeds suitable for forming

large lakes." – The use of 'riverbed' here suggests this is not discussing supraglacial lakes, which is confusing given that is the focus of the paper. Clarification needed.

Lines 147-152: I don't understand where the inferences of "lack stable water sources" and "potential water supply" come from. In general, I am finding it difficult to follow the main points that these sentences are trying to make.

Line 163: "…nine groups of supraglacial lakes" – I do not understand the context of these nine groups as this has not been clearly defined before this. There are 19 labelled glaciers in Figure 2, but how these are subdivided into nine groups is not clear and needs to be explained. No need to mention the study area again here.

Line 164-165: "Snow and ice ablation together with precipitation expanded the extent of surface water, while the continuous increase in meltwater promoted the seasonal acceleration of ice flow." – how are you able to distinguish these different components from your dataset? This needs to be clearly explained.

Lines 166-175: Similar to a previous comment, the constant change in unit order of magnitude makes it hard to gauge the differences between reported lake sizes. Use the same units throughout the paper. Here, you also name a large number of glaciers – these should be clearly identified on your figures in some way.

Line 177: clarify if you mean that the number of lakes expanded, or individual lakes expanded.

Lines 177-181: "while the remaining six glaciers showed decreasing trends" – with the named three glaciers in this sentence, this suggests there are nine glaciers. The following sentence also refer to "nine glaciers" – but elsewhere you refer to "nine glacier groups" and Figure 2 suggest there are up to 19 glaciers. Clarification needed here and elsewhere – how many glaciers form part of the study area?

Lines 181-183: The reported values here are hard to understand out of context so I would just cite the figure.

Lines 183-185: "The time series of lake area changes, together with meteorological observations at the sites, indicate that supraglacial lake expansion and seasonal signals are highly sensitive to climate anomalies." – how is this conclusion arrived at? To me it doesn't seem to follow logically from the text immediately beforehand, or Figure 3. More explanation and justification is required.

Line 198: How were "downstream drainage events" determined from the optical satellite images? Reduction in lake area? I don't find an explanation of this in the methods.

Lines 219-222: The captions for Figure 4 panels a and b are not very informative at all.

Lines 229-230: This seems to be methods.

Line 233: "…temporal and magnitude characteristics of adjacent glaciers" – of the lakes on those glaciers?

Line 236: "During historical periods…" – when is this referring to? It's a bit confusing because you then seem to report data, presumably from this study. Clarification is needed.

Lines 236-240: I'm not sure what these mean velocities are being reported for here? Some context is needed.

Lines 243-245: "On Glacier No. 9, we detected pronounced flow accelerations during March–May 2017 (0.19 m day^{-1}) and April–May 2019 (0.18 m day^{-1})" – for comparison, what are representative velocities outside of these periods?

Line 251: "…we recorded recurrent glacier surges" – I'm not sure which glacier/plot this is referring to as Khumbu is not seemingly plotted in the referenced figure – but either way, I would suggest the authors reflect on whether these are 'glacier surges' based on existing definitions, e.g. https://doi.org/10.3189/2015JoG14J136. To me, none of the velocity plots seem to identify glacier surges (orders of magnitude velocity increases for extended periods of time, e.g. months to years). What are the timescales of the "recurrent glacier surges", i.e. how often do they recur, for how long. All of this information would help to support such an interpretation. That said, I understand this is not the main focus of the paper, so I would perhaps suggest the authors reconsider whether introducing this here with insufficient detail is necessary for the overall study.

Line 255: "surface deformations" - as noted previously, I think a clearer definition of this term is needed. Is this horizontal, vertical change or both? Is this on the glacier itself, or in moraine areas, or both (e.g. debris-covered ice)?

Line 257: "average cumulative displacement" - is this vertical or horizontal change? I am finding it hard to understand what these changes are referring to.

Line 259: "minor subsidence or deposition of terminal debris" - this suggests vertical change at least for 'minor subsidence'. What impact does "deposition of terminal debris" have on the surface change?

Lines 256-276: The reported values of change in this section seems very small, in all cases <0.5 m. I remain unclear if this is vertical or horizontal change, and over what timescales, but also the source data used appears to be Sentinel 1/2 - which has considerably larger pixel sizes than the reported changes. I presume there is a methodological explanation for this, but

having read the associated methods section I am still struggling to understand this (the methods largely relate to ice velocities).

Lines 284-288: I am finding it hard to follow these values as they are reported here. What dates are being compared? I understand one of the values is 2022-23 in each case, but what is the other?

Discussion

Line 325: “in turn reshapes the bedrock at glacier termini” - what evidence is this based on? Where are you measuring bedrock changes?

Lines 325-326: “such irregular and sudden collapses...” - I’m not clear what you are referring to here. What is collapsing? The previous sentence mentions only ice acceleration. The rest of the sentence seems to suggest this is related to glacier retreat (creating room for lakes) but this is really not clear from these sentences.

Lines 332-333: “The reduction in basal traction drives surface subsidence, with glacier advance creating the necessary bedrock depressions and moraine dams.” - I am struggling to follow this argument. Ice acceleration leads to lower surface elevations? Why is this? And how does advance create “necessary bedrock depressions and moraine dams”? This all needs to be explained in a much clearer way.

Lines 338-339: “In Sagarmatha National Park, the ablation zones of glaciers are almost entirely covered by continuous debris, which contributes to slower ice flow and frequent geohazards.” - this is important context for the study glaciers that should come earlier in the paper.

Lines 343-344: “seasonal fluctuations are primarily driven by basal sliding (subglacial water flow) and the support provided by supraglacial debris” - what does “support provided by supraglacial debris” mean here?

Figures

Figure 1: – I am struggling to understand this figure. It is not clear if this is a schematic diagram or a location map. There is no spatial context beyond a global inset, which is not much help. There are no named locations/peaks/glaciers that would help the reader. I don’t understand what the different numbered locations are there do not appear to be any links to supporting datasets that presumably this information has been derived from – and they do not seem to relate to the numbered glaciers in Figure 2. The significance of the precipitation/evaporation/glacial lake/glacier/snow/avalanche/GLOF risk labels is not clear – are these mapped in specific locations for specific reasons? In summary, this figure needs a substantial rethink.

Figure 2: In both panels a and b, there is a yellowy-green polygon in the bottom right corner of the maps – what is this and what are the colours showing? This is not explained. Panels a-1 and b-1 are hard to see and would benefit from being made larger as separate panels below the maps. Panel a-1: what are the x axis units of this plot? The numbered purple circles are presumably individual glaciers but this is not explained anywhere - and these seem to have no connection to the numbered circles in Figure 1. Panel c: this shows “lake centroids’ migration distances and elevation gradients” according to the caption. By migration (which I am assuming is the same thing as ‘displacement’ mentioned elsewhere), is this the downglacier movement of the lake as the ice flows? If so, the arrow pointing upglacier “0àMax” is counterintuitive as it appear to imply upglacier movement. For the profiles in this panel, what do the different colours brown and grey-blue signify?

Figure 3: What is the source of the meteorological data plotted for each glacier? Is this measured in the field at each site? I can’t find mention of these datasets in the methods. I can’t detect any difference between the shadings for pre-monsoon/monsoon and post monsoon/winter – I only see white or grey bands. What is showing on the y axes of these plots? This is not labelled. The x axes suggests the date format is YYYY-MM-DD but on the plots it is YYYY-M. Labelling the plots directly with the glacier names, and how these relate to the numbered glaciers in Figure 2, would be useful.

Figure 4: Panel a: What is the “Melung” label? Is this part of the overall glacier? This needs to be clarified (and might explain the confusion over glacier numbers elsewhere in the paper). The inclusion of the proglacial lake here indicates that this paper isn’t only about supraglacial lakes, as the title seems to state. Glacier No. 9 here doesn’t appear to be the same number 9 glacier on Figure 2 – is it? This could be confusing. Panel b: label the lakes here. What do the reported values here relate to? The caption does not explain this. Panels d and e: what are Lakes A, B, C and D on these diagrams? What are the curved blue arrows under the glacier signifying?

Figure 5: This seems to be the same plot repeated ten times. I am struggling to see any difference between the panels, or how these relate to the lakes that are labelled on each panel. It is hard to see the purple arrows “water vapor flux” or anything related to the “correlation between lake area and SST anomalies” colour scheme. The figure caption describes scatter plots

but I cannot see these.

Harold Lovell
University of Portsmouth
Monday 16th February 2026

Version 1:

Reviewer comments:

Reviewer #1

(Remarks to the Author)
All my comments in the last time have been fully addressed. Therefore, this manuscript is recommended to be accepted as its current version.

Reviewer #2

(Remarks to the Author)
The author team should be commended on their clear and comprehensive response to reviewer comments. The major issues that were raised have been dealt with expertly. For example, the adjustment of the glacier naming/numbering means this is now much clearer to follow. Equally, the introduction is now much better focused on the central themes of the study, and the methods have been improved so that issues of clarity raised in later stages of the paper are now addressed.

I have only a handful of minor comments on the figures, which I think could be improved further, but otherwise I am happy that this is ready for publication.

Further comments on figures
Figure 1 – I would add numbers to the study site circles on the map – perhaps in a different colour to differentiate from the numbered event circles. Better still, use a different numbered shape for the study sites, e.g. square or diamond.
Figure 3 – technically the date format YYYY-MM-DD is not followed on the x axis – this would be, for example, 2016-07-01.
Figure 4, panels (d) and (e) – the blue line identified as subglacial meltwater transport remains confusing – this looks like groundwater flow on the diagram as it is within the substrate rather than at the ice-bed interface. I suggest some further improvements to make this clearer.
Extended Data Fig. 1 – the pin for Group 4 appears to be located in a subbasin without glaciers, and also overlays and partially obscures a glacier name. Suggest some minor adjustments to improve this. Perhaps moving the Group 4 label to immediately below the line extending from the circle would help, and moving the circle down slightly so I doesn’t obscure the text.

Version 2:

Reviewer comments:

Reviewer #2

(Remarks to the Author)
All further suggestions for changes to figures have been addressed well, this is ready for acceptance in my view.

Open Access This Peer Review File is licensed under a Creative Commons Attribution 4.0 International License, which permits use, sharing, adaptation, distribution and reproduction in any medium or format, as long as you give appropriate credit to the original author(s) and the source, provide a link to the Creative Commons license, and indicate if changes were made.
In cases where reviewers are anonymous, credit should be given to 'Anonymous Referee' and the source.
The images or other third party material in this Peer Review File are included in the article's Creative Commons license, unless indicated otherwise in a credit line to the material. If material is not included in the article's Creative Commons license and your intended use is not permitted by statutory regulation or exceeds the permitted use, you will need to obtain permission directly from the copyright holder.
To view a copy of this license, visit https://creativecommons.org/licenses/by/4.0/

Authors' responses to reviewers' comments

Unraveling daily dynamics of supraglacial lakes in response to hydrological pulses and anomalous climate signals

Jian Song, Haijun Qiu, Adam Emmer, Guoqing Zhang, Ninglian Wang, Ya Liu, Guoqing Yang, Yiwen Liang

Submission ID: **NCOMMS-26-000007**

Note：

Response to the reviewer's comments (Reviewer 1): Please refer to Page 2-20.

Response to the reviewer's comments (Reviewer 2): Please refer to Page 21-122.

Response to the reviewer’s comments (Reviewer 1)

General comments:

Comment 1.1: This paper is very comprehensive, which is in large part due to the extensive dataset, and I commend the authors for completing this impressive and massive amount of work. According to the authors, the daily fluctuation dataset of glacial lakes relies primarily on visual interpretation, with a total of 13,135 images. In my view, this is an extremely large and time-consuming task. I wonder why the authors did not employ the latest research techniques, such as deep learning or existing water monitoring methods (e.g., A Glacial Lake Mapping Framework in High Mountain Areas: A Case Study of the Southeastern Tibetan Plateau; Automatic Extraction of Glacial Lakes from Landsat Imagery Using Deep Learning across the Third Pole Region; Surface Water Mapping by Deep Learning). Currently, most studies on glacial lakes focus on annual changes, with only a few addressing seasonal-scale variations. In my opinion, the authors’ study of glacial lakes at a daily timescale is very novel. Therefore, could this approach be extended to broader regions, such as the high-latitude Arctic, the Andes, or Alaska? This would be highly valuable for future related research.

Response：We sincerely thank you for your comment. As you mentioned, this is a highly labor-intensive study; just visually interpreting 13,135 images took us more than half a year. The four studies you cited were partially attempted during the initial stage of our work. However, their datasets were mainly based on Landsat or Sentinel imagery. This leads to lower accuracy when performing deep learning simulations compared to Planet imagery. Moreover, since the revisit intervals of Landsat and Sentinel are longer than Planet imagery, models trained on Landsat or Sentinel data may exhibit significant deviations when applied to new data, especially across different seasons and years. The distinctive feature of our study is that it focuses on the daily variations of small glacial lakes, which requires very high precision. Therefore, we opted for this time-consuming approach, which, however, yields much more accurate results. This study provides important reference value for related global and regional research. Currently, global datasets for glacial lake changes still rely on Landsat and Sentinel

imagery. Although previous studies are abundant, their lower-resolution imagery often overlooks small glacial lakes, which are numerous and cover significant areas. Additionally, our work emphasizes daily fluctuations of lake area, addressing the lack of short-term lake area dynamics in previous research and providing more detailed information on glacial lake changes. Regarding the question, "Can this approach be extended to broader regions, such as high-latitude areas in Antarctica, the Andes, Alaska, and Greenland?"—there is no doubt that this study has great potential for broader application, and we are indeed working in this direction. However, different regions have their own specific characteristics, which require obtaining and validating relevant data from these areas. This is a highly labor-intensive task, and completing it will require more time. Nonetheless, this work is undoubtedly valuable, has significant potential for future research, and we will strive to complete it as soon as possible.

Comment 1.2: This study primarily focuses on small lakes (indeed, understanding their dynamics addresses a clear research gap, and this study provides unprecedented detailed insights). However, do the outbursts of larger lakes often cause greater hazards? I acknowledge that recent studies suggest this is not necessarily the case. Nevertheless, the lakes examined in this study are much smaller than those involved in current glacial lake outburst cases. Although the authors also focus on lake drainage and lifecycle issues (Figs. 3 & 4), the discussion should emphasize the role of these lakes in natural environments and mountain hazards, as this is one of the core aspects of the paper. Please refer to Flood Risk from Small Mountain Lakes.

Response： We sincerely thank you for your comments and suggestions. Based on the issues you pointed out, we have emphasized the role of small glacial lakes in mountain hazards, provided comparative examples for illustration, and highlighted the importance of monitoring their seasonal dynamics *(For details, please see the revised manuscript: Lines 84-88, Lines 102-107, Lines 108-113, Lines 412-417, Lines 465-478 (This has also been presented in this response))*.

> ***Lines 84-88 IN THE REVISED MANUSCRIPT:*** *A typical example by Miles[27], based on Planet imagery, documents an SGL outburst on the Khumbu Glacier. Multiple small SGLs rapidly coalesced prior to sudden drainage, reshaping the glacier surface and triggering*

debris-flow erosion and landslides within the glacier hydrological system, forming a cascading hazard chain. This process occurs over very short timescales.

Lines 102-107: *For example, the 1985 Dig Tsho GLOF alone destroyed 14 bridges and the nearly completed Namche hydropower station. To mitigate the potential outburst risk of large moraine-dammed lakes, long-term monitoring and engineering interventions have been implemented, successfully preventing lake outburst events to some extent34,35. However, small SGLs, owing to their rapid evolution and unpredictable seasonal variability, are increasingly becoming a major source of GLOFs; since 2000, five of the seven recorded GLOF events in the region have originated from SGLs*[9].

Lines 108-113: *As critical indicators of global climate change, seasonal fluctuations in lake area are considered one of the most sensitive characteristics of small SGLs. Some case studies show that, prior to reaching critical outburst thresholds, some SGLs can expand rapidly over short timescales, with surface area increases exceeding eightfold, and reaching up to 70-fold over a complete seasonal expansion cycle*[36].

Lines 412-417: *Seasonality is an important characteristic of outburst floods in high-mountain regions. Sattar et al. documented at least seven flood events in the Liming Valley, all occurring during summer, a similar seasonal pattern is also observed in the glacial lake outburst events identified in this study. This is primarily because seasonal meltwater plays an important role in destabilizing drainage systems. These findings underscore the importance of closely monitoring glacier dynamics and the seasonal evolution of SGLs*[37].

Lines 465-478: *Lakes and hydrological systems are highly sensitive to long-term climate warming. With the increasing frequency and intensity of extreme events worldwide, lake and river ecosystems are emerging as important indicators for the early warning of global hazards. Previous studies*[68-70] *suggest that the rapid rise in lake surface water temperature is closely linked to intensified summer heat extremes, with anomalies primarily driven by surface air temperature and further modulated by local lake characteristics and ice-cover dynamics. Riverine heatwaves (weekly mean water temperature > 90th percentile) are also*

intensifying, highlighting the growing vulnerability of hydrological systems to climate extremes[71]. Current global lake models and early-warning frameworks remain largely focused on large lakes and river systems. However, small mountain lakes have been shown to exhibit amplified responses to climate variability. Consistent with this, our results demonstrate that small SGLs respond rapidly to climate change, with short-term fluctuations acting as sensitive indicators of environmental variability. Incorporating the seasonal dynamics of small SGLs into existing early-warning frameworks, and developing integrated systems across multiple lake scales, is therefore critical for improving the prediction and response capacity for extreme climate events and abrupt hydrological hazards.

Comment 1.3: After these points, I would like to emphasize that the authors' efforts on small glacial lakes are valuable. These data are novel and provide new insights and reference cases for discussing mountain hazards in the study region as well as trends in global glacial lakes. However, I suggest that the authors could expand the discussion to highlight the broader significance of this study for related global research. Moreover, the spatial heterogeneity of glacial lake distribution varies across different dimensions and regions. The authors could, by drawing on other studies, appropriately compare the characteristics of glacial lakes in regions such as the Tibetan Plateau, the Andes, Alaska, and Greenland, thereby emphasizing the distinctiveness of their research. Additionally, the authors could use this study as a basis to outline prospects for future related research (I am particularly interested in the application of this study to short-term variations of glacial lakes globally), which would be highly valuable.

Response: We sincerely thank you for your comment. Following your suggestion, we have emphasized the characteristics of glacial lake changes reported in previous global studies, highlighted the significance of our study on small supraglacial lakes, and compared the features of glacial lakes across different regions to underscore the uniqueness of supraglacial lakes in the Himalayas. At the same time, we have highlighted the implications of our work for future research *(For details, please see the revised manuscript: Lines 466-479(This has also been presented in this response))*.

Lines 466-479 IN THE REVISED MANUSCRIPT: *Lakes and hydrological systems are highly sensitive to long-term climate warming. With the increasing frequency and intensity of extreme events worldwide, lake and river ecosystems are emerging as important indicators for the early warning of global hazards. Previous studies[68-70] suggest that the rapid rise in lake surface water temperature is closely linked to intensified summer heat extremes, with anomalies primarily driven by surface air temperature and further modulated by local lake characteristics and ice-cover dynamics. Riverine heatwaves (weekly mean water temperature > 90th percentile) are also intensifying, highlighting the growing vulnerability of hydrological systems to climate extremes[71]. Current global lake models and early-warning frameworks remain largely focused on large lakes and river systems. However, small mountain lakes have been shown to exhibit amplified responses to climate variability. Consistent with this, our results demonstrate that small SGLs respond rapidly to climate change, with short-term fluctuations acting as sensitive indicators of environmental variability. Incorporating the seasonal dynamics of small SGLs into existing early-warning frameworks, and developing integrated systems across multiple lake scales, is therefore critical for improving the prediction and response capacity for extreme climate events and abrupt hydrological hazards.*

Minor comments/ questions:

Abstract

Comment 1.4: L32: In your other regions, you refer to supraglacial lakes, but here you wrote glacial lake. Are you trying to emphasize that you are studying a specific type of glacial lake, or is this a mistake? Please check.

Response: Thank you for your comment. This paper primarily focuses on supraglacial lakes; however, some of the references we cited study glacial lakes. Therefore, when citing these studies, we used the terminology from the original papers, while in the context of our paper we consistently use “supraglacial lakes (SGL)”. Based on your feedback, we have carefully checked the entire manuscript to ensure that our terminology is accurate and appropriate.

Results

Comment 1.5: L132: Delete “ponds” from “From 2016 to 2024, we detected 1,493 supraglacial lakes and ponds” and check the entire manuscript for consistency.

Response：Thank you for your comment. We have made the revisions according to your suggestions.

Comment 1.6: L162: “Glacial lake” or “supraglacial lakes”?

Response: We sincerely thank you for pointing out our error. Here, we should have used “supraglacial lakes,” and we have made the corresponding corrections.

Comment 1.7: L202: No. 9 Glacier? Does this glacier not have a name? I noticed that the others all have names—please confirm.

Response: Thank you very much for pointing out this issue. The manuscript involves multiple classification and labeling schemes, including glacier numbers, glacier system numbers, and glacier names, which can indeed lead to confusion. The main reason for this issue is that some glaciers have branched structures, resulting in inconsistencies in the classification system used for different types of data analysis. Therefore, we have redefined and unified the classification rules: when upstream glaciers share a single drainage outlet, they are treated as one glacier system and designated as Group 1, Group 2, …, Group 9.

Most glacier groups consist of a single glacier, such as Group 2 (Lhotse glacier), Group 3 (Lhotse Nup glacier), Group 4 (Ama Dablam glacier), Group 5 (Nuptse glacier), Group 6 (Khumbu glacier), Group 7 (Ngozumpa glacier), and Group 9 (Melung glacier). Some groups include two or more glaciers, such as Group 1 (Lhotse Shar, Imja, and Ambulapcha glacier) and Group 8 (Bhote Koshi and Lumsamba glacier)

Regarding the glacier numbering in Fig. 2, due to the branched structure of many glaciers, we redefined the numbering scheme to track lake dynamics across different glacier branches. **Each branch unit was renumbered using a group-consistent labeling system, replacing the**

original 1–19 numbering scheme. For example, Group 9 contains three branches, labeled as Group 9-1, 9-2, and 9-3, whereas Group 2 has no branches and therefore remains as Group 2. To clarify the correspondence between each number and the glacier branches, we have added four supplementary figures in the Supplementary Information, which provide a detailed mapping between the numbering system and the glacier branches.

As this study involves a large number of glaciers and glacier branches, frequent use of individual glacier names in the main text would increase reading complexity and reduce readability. Therefore, in the manuscript, we consistently use glacier group numbers when presenting specific analyses (e.g., "the total area of Glacier Group 1 expanded …" instead of "the total area of Lhotse Shar, Imja and Ambulapcha Glaciers expanded …"). In the figures, we also uniformly adopt the nine glacier group labels (Group 1–9), while glacier names are only provided in the figure captions. This approach enables readers to refer to the corresponding figures and captions when needed, thereby easily linking each glacier group with its constituent glaciers and improving overall readability.

We have revised the paragraph as follows:

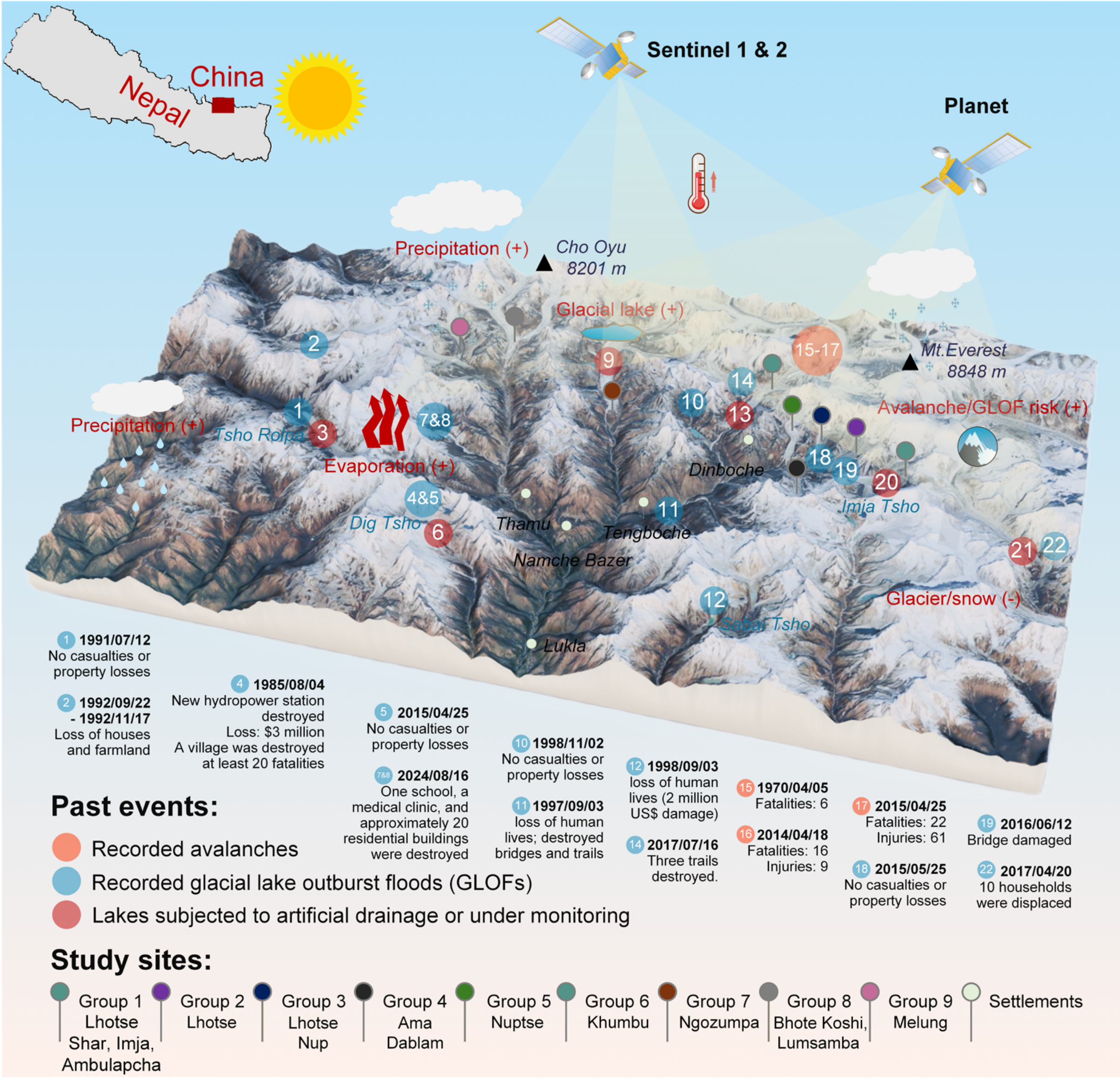

Fig. 1 | Diagram illustrating lake evolution and geological hazards in Sagarmatha National Park. The red labels and text represent the climate-related risks facing this region, namely that under global warming, increased snowfall/rainfall and the melting of glaciers and snowpack lead to the expansion of lake boundaries and heightened risks of GLOFs and avalanches. The numbers in the figure indicate the locations and associated hazard losses of past GLOFs, avalanches, and manually monitored lakes (Data source: Table S1). Glaciers sharing the same drainage outlet were grouped together and labeled as Groups 1–9. The glaciers included in each group are listed as follows: **Group 1** Lhotse Shar, Imja, and Ambulapcha; **Group 2** Lhotse; **Group 3** Lhotse Nup; **Group 4** Ama Dablam; **Group 5** Nuptse; **Group 6** Khumbu; **Group** 7 Ngozumpa; **Group 8** Bhote Koshi and Lumsamba; **Group 9** Melung Glaciers. Extended Data Fig. 1 serves as a supplement to this figure, documenting the spatial

information of clean glaciers, debris-covered glaciers, and runoff.

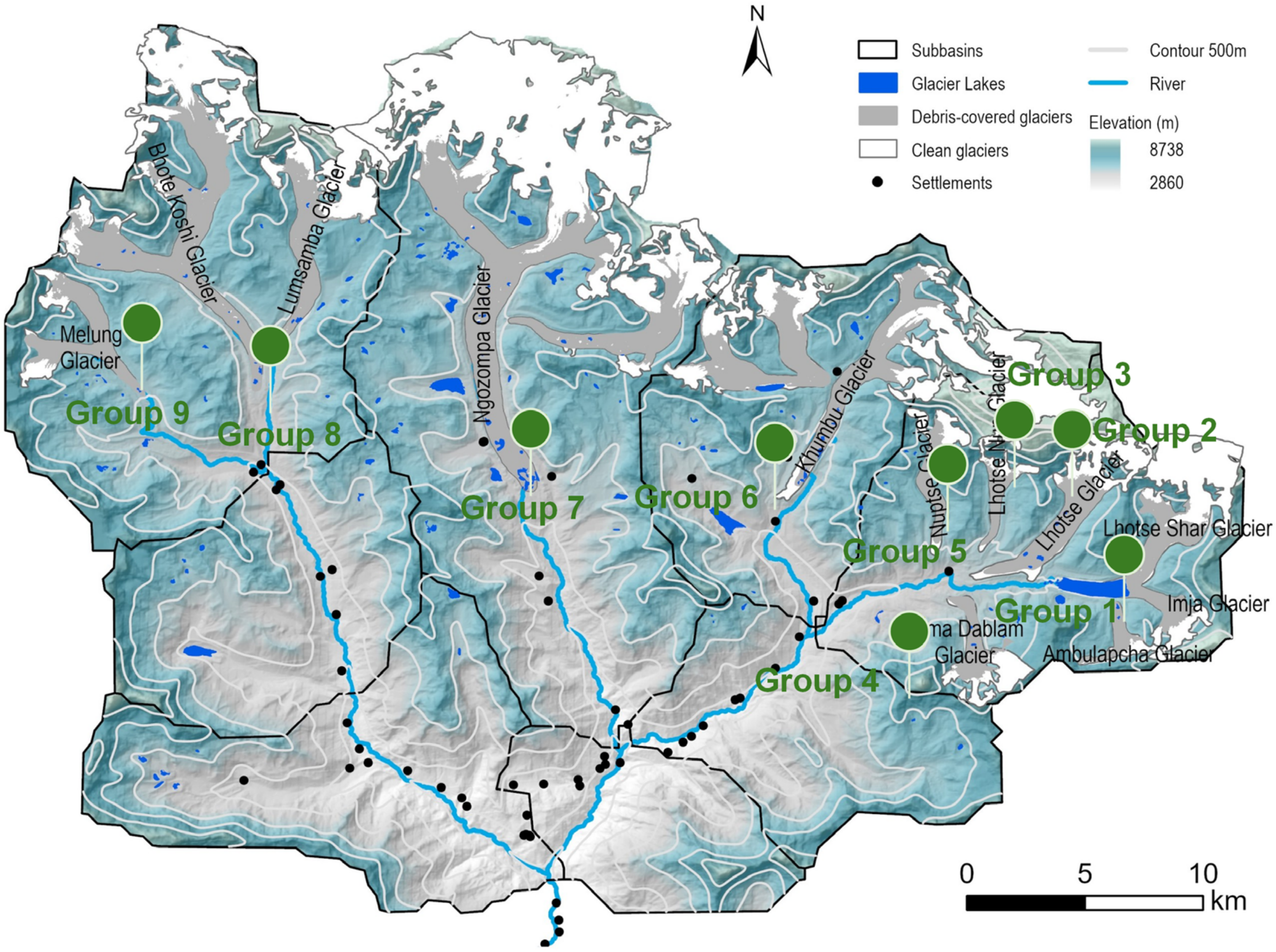

Extended Data Fig. 1 | Spatial distribution and classification of glaciers. Glaciers sharing the same drainage outlet were grouped together and labeled as Glacier Group 1–9. Except for Glacier Groups 1 and 8, which contain two and three individual glaciers, respectively, each of the remaining groups consists of a single glacier. The details are as follows: **Group 1** Lhotse Shar、Imja and Ambulapcha; **Group 2** Lhotse; **Group 3** Lhotse Nup; **Group 4** Ama Dablam; **Group 5** Nuptse; **Group 6** Khumbu; **Group 7** Ngozumpa; **Group 8** Bhote Koshi and Lumsamba and **Group 9** Melung Glaciers**.**

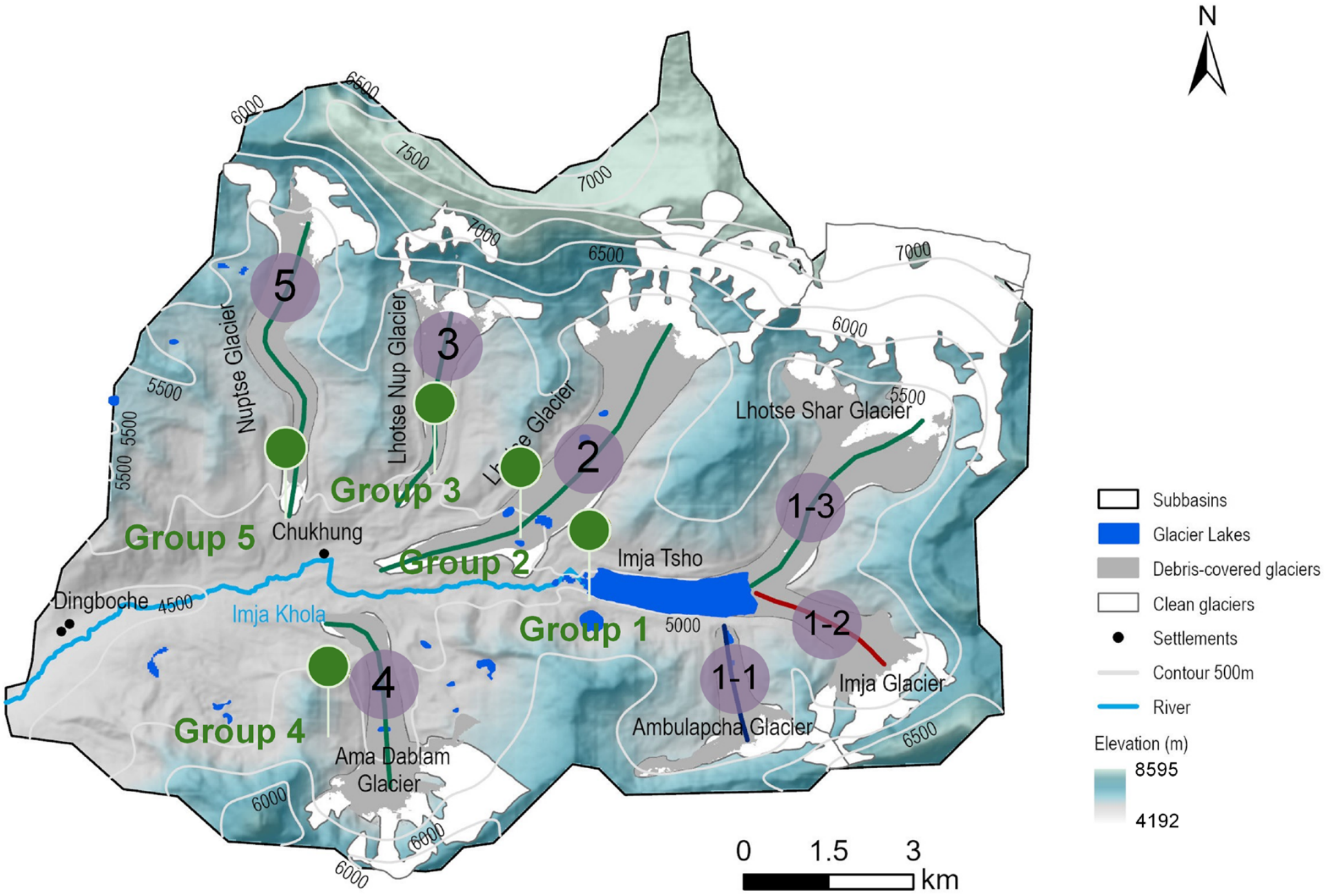

Fig. S1 | Criteria for glacier system classification and spatial distribution of glaciers. Group 1-5 represent the spatial distribution of five glacier systems, and the numbers correspond to the glacier branches in Fig. 2. The relationships are as follows: **Group 1** Lhotse Shar Glacier (circled 1-1), Imja Glacier (circled 1-2), and Ambulapcha Glacier (circled 1-3); **Group** 2 Lhotse Glacier (circled 2); **Group** 3 Lhotse Nup Glacier (circled 3); **Group** 4 Ama Dablam Glacier (circled 4); and **Group** 5 Nuptse Glacier (circled 5).

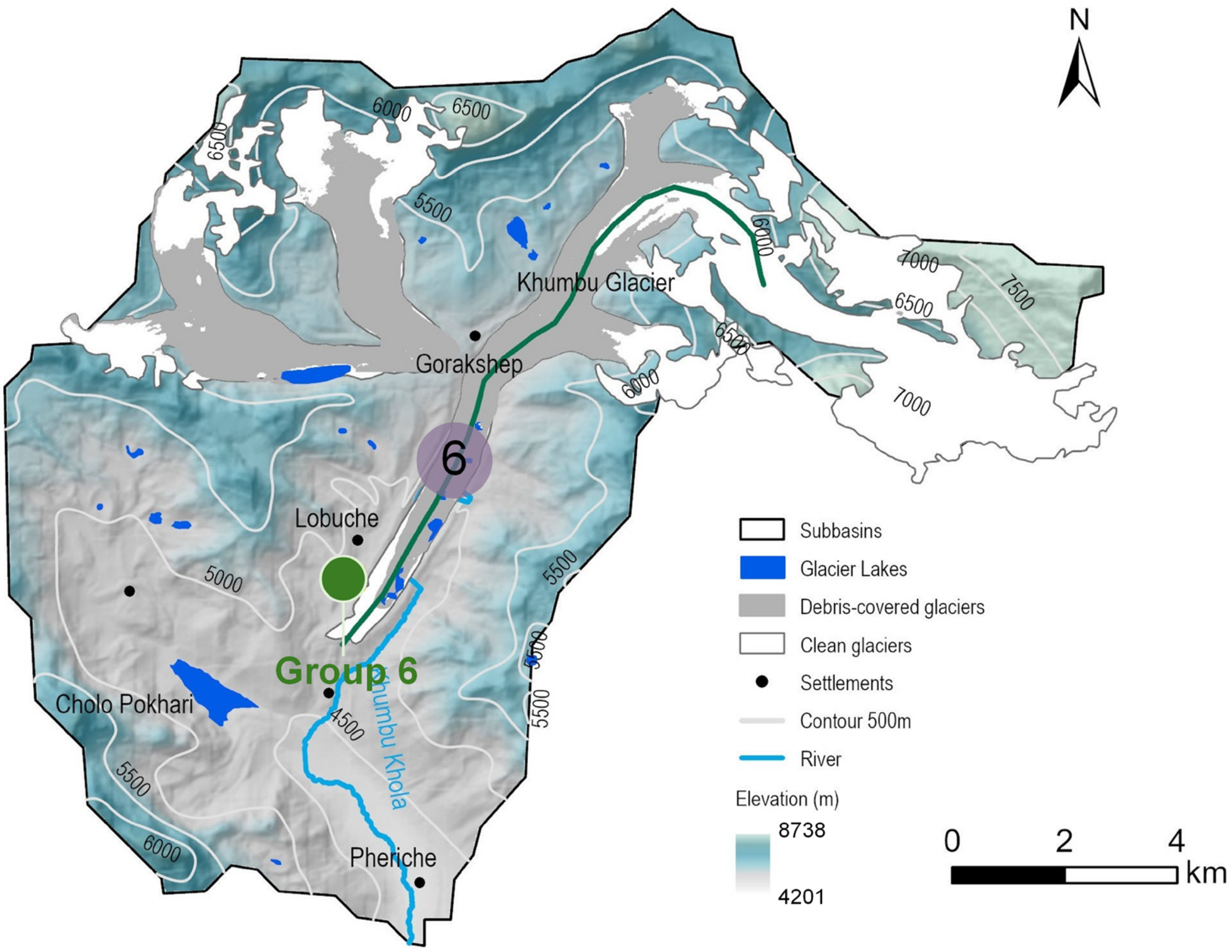

Fig. S2 | Criteria for glacier system classification and spatial distribution of glaciers. Group 6 represents the spatial distribution of the glacier group, and the number corresponds to the glacier branch in Fig. 2. The correspondence is as follows: **Group 6** Khumbu Glacier (circled 6).

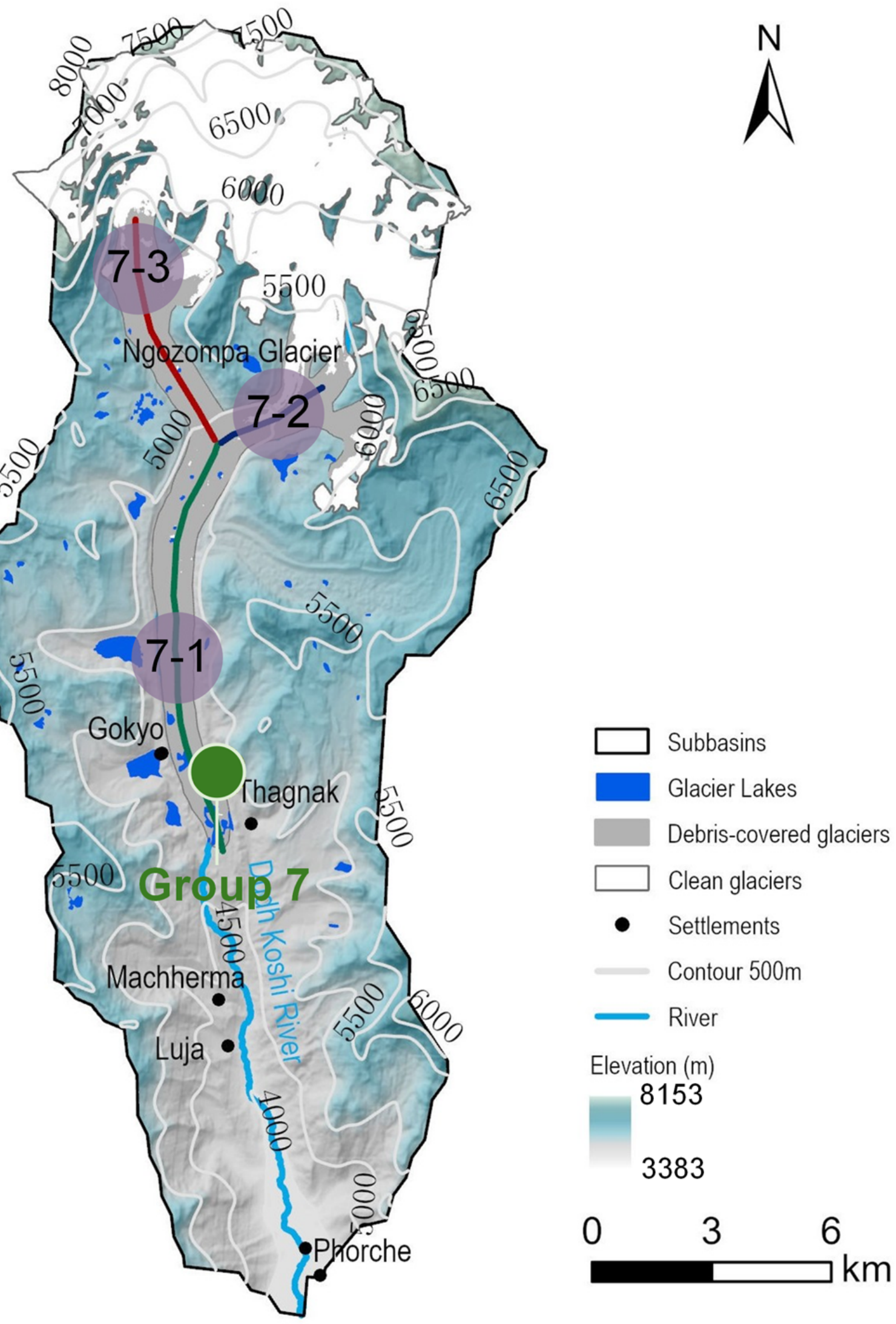

Fig. S3 | Criteria for glacier system classification and spatial distribution of glaciers. Group 7 represents the spatial distribution of the glacier group, and the number corresponds to the glacier branch in Fig. 2. The correspondence is as follows: **Group** 7 Ngozumpa Glacier (circled 7-1, 7-2 and 7-3).

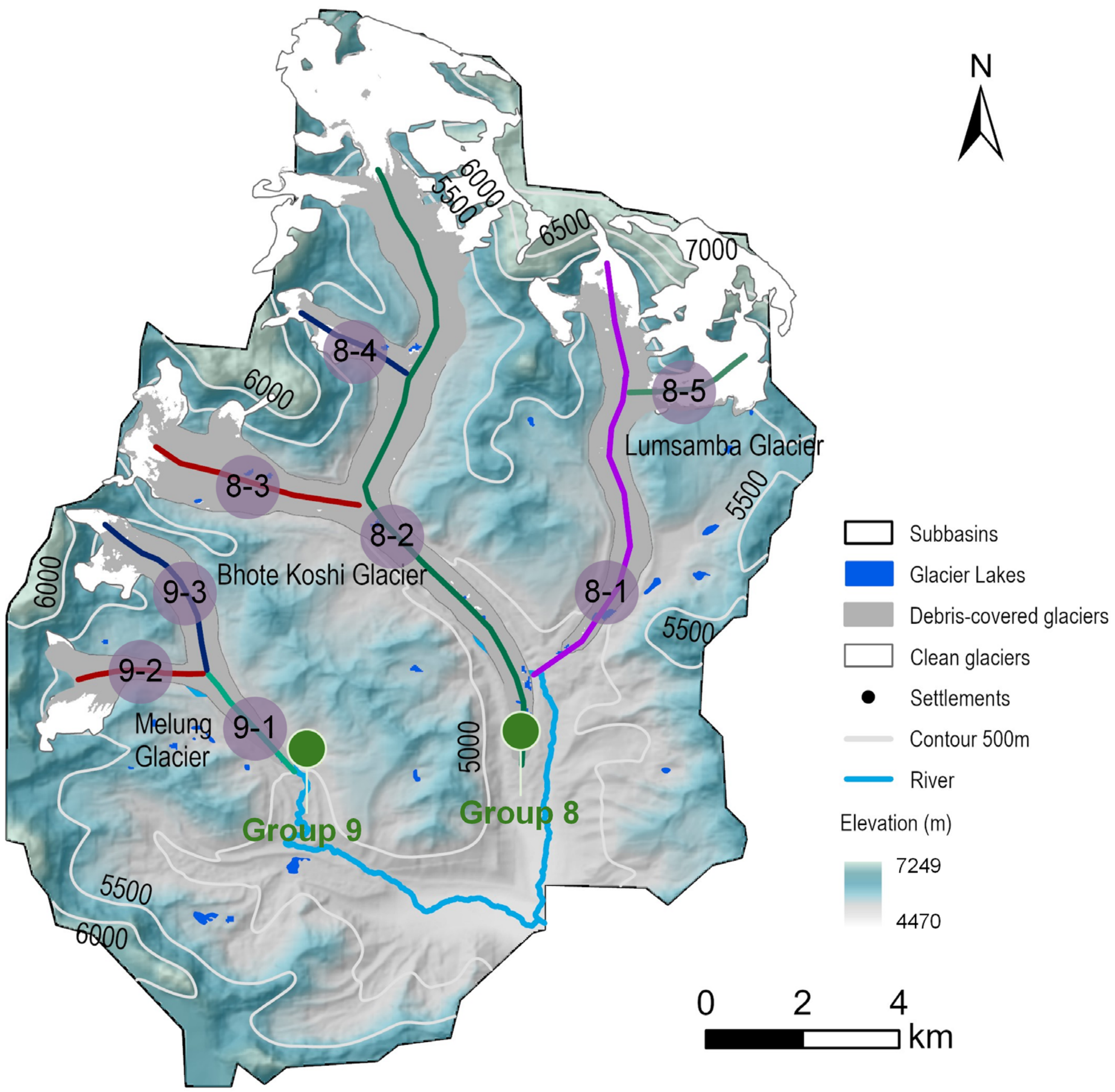

Fig. S4 | Criteria for glacier system classification and spatial distribution of glaciers. Group 8-9 represents the spatial distribution of the glacier group, and the number corresponds to the glacier branch in Fig. 2. The correspondence is as follows: **Group** 8 Bhote Koshi Glacier (circled 8-2, 8-3 and 8-4) and Lumsamba Glacier (circled 8-1 and 8-5). **Group** 9 Melung Glacier (circled 9-1, 9-2 and 9-3).

Discussion

Comment 1.8: L331-337: Please add references in the appropriate places.

Response: Thank you for your comment. We have made some revisions to this section and, following your suggestion, have added the relevant references *(For details, please see the revised manuscript: Lines 357-366, Lines 369-373, Lines 374-377 (This has also been presented in this response))*.

We have revised the paragraph as follows:

> ***Lines 357-366 IN THE REVISED MANUSCRIPT:*** *Previous studies indicate that seasonal melting of debris-covered surfaces and glacier ice generates substantial meltwater. which drains through crevasses into the englacial hydrological system and is routed toward the glacier margins or bed. This process reduces basal traction and induces hydraulic uplift, both of which promote ice-flow acceleration48. Under the combined effects of accelerated flow, enhanced longitudinal strain, dynamic thinning and crevasse-driven frontal retreat, calving becomes dominant. Continued extensional flow deepens crevasses in the glacier terminus, while thinning reduces freeboard height (the elevation of the ice surface above the lake level), thereby promoting mechanical calving and frontal retreat and increasing the likelihood of ice-dam instability and failure. These processes collectively alter the geometry of glacier termini and are accompanied by surface lowering driven by ice thinning and dynamic adjustment*[42,48-50].

> ***Lines 369-373:*** *These patterns support King's two-stage model of glacier-lake evolution in the Himalaya*[48]*. During the lake expansion stage, rising lake levels or thinning of the host glacier induce crevassing, accelerating frontal retreat, steepening the glacier surface, and enhancing ice-flow velocity. In the later stage of lake growth, sustained ice-front retreat and glacier thinning narrow the glacier, reducing driving stresses while increasing lateral and basal resistance, thereby slowing ice flow and diminishing retreat rates*[48].

Lines 374-377: *At the same time, with future mass loss of glaciers in the Himalaya, glacier retreat and thinning will be accompanied by the formation and expansion of new lakes, as well as accelerated ice flow and instability at glacier termini*[46].

Comment 1.9: L338-339: Please include the table of disaster information from your appendix here.

Response: Thank you for your comment. This section was somewhat leaning toward introductory content, so we have moved the descriptions related to hazards to the Introduction. In addition, following your suggestion, we have included the hazard information table in the Supplementary Information (Table S1).

Comment 1.10: L338-362: In this section, you mention glacial lakes in the specific study area and glacial lake outbursts in other regions. You could briefly highlight some characteristics of small glacial lakes in other regions, keeping the discussion concise, but try to provide a comparative analysis and emphasize the distinct features of your study area.

Response: We sincerely thank you for your valuable comment. Following your suggestion, we have outlined and compared the characteristics of lakes in other regions with a higher abundance of glacial lakes, and clarified the future research directions as well as the significance of this study *(For details, please refer to the revised manuscript: Lines 393-409 (This has also been presented in this response))*.

We have revised the paragraph as follows:

Lines 393-409 IN THE REVISED MANUSCRIPT: *Long-term monitoring of glacial lake number and area has become central to assessing outburst flood hazards. Rick et al. reported 1,150 drainage events from 106 ice-dammed lakes in Alaska (1985–2020), showing stable event frequency but declining magnitude*[54]*. A global synthesis of 1,569 events by Veh et al. further reveals an order-of-magnitude decrease in extreme peak discharge and flood volume across most regions since 1900*[9]*. Despite the growing completeness of global ice-dammed lake databases, the spatiotemporal response of*

supraglacial and small glacial lakes to climate change remains poorly constrained. Based on our analysis of seasonal SGL dynamics, we find that their short-term variability is highly sensitive to climate forcing, providing near-real-time, within-year feedback and potential early indicators of extreme climate anomalies. Meanwhile, Himalayan SGLs primarily form from surface meltwater accumulation and exhibit pronounced seasonality, whereas glacial lakes in the Andes are predominantly moraine- or ice-dammed. Alaskan lake systems are more complex, comprising ice-dammed, proglacial, and some small SGLs. In Greenland, intense summer meltwater input leads to widespread surface ponding and the formation of SGLs. Whether such spatial heterogeneity shares similarities with, or differs fundamentally from, the characteristics of Himalayan SGLs remains to be further investigated[12,43,55,56]. Therefore, future work should focus on quantifying the impacts of small SGLs on global ecosystems, high-mountain water resources, and hazard risks under different glacier retreat scenarios.

Comment 1.11: L379-381: Please add references to support this conclusion.

Response: Thank you for your comment. Following your suggestion, we have added the relevant references.

Comment 1.12: L401: At the end of the discussion, briefly highlight the limitations of your study and outline future prospects and points of interest, keeping it concise and insightful.

Response: We sincerely thank you for your valuable suggestion. As you pointed out, there are many potential directions to extend this study, and we are currently conducting related work. However, given that the focus of this paper is the seasonal dynamics of small glacial lakes, we have placed the future perspectives, outlook, and research priorities on the spatial heterogeneity of small glacial lakes worldwide and their responses to climate. Specifically, future research will focus on the significance of small glacial lakes for high-mountain ecosystems, mountain hazards, and water resources *(For details, please refer to the revised manuscript: Lines 466-479 (This has also been presented in this response))*.

We have revised the paragraph as follows:

> ***Lines 466-479 IN THE REVISED MANUSCRIPT:*** *Lakes and hydrological systems are highly sensitive to long-term climate warming. With the increasing frequency and intensity of extreme events worldwide, lake and river ecosystems are emerging as important indicators for the early warning of global hazards. Previous studies[68-70] suggest that the rapid rise in lake surface water temperature is closely linked to intensified summer heat extremes, with anomalies primarily driven by surface air temperature and further modulated by local lake characteristics and ice-cover dynamics. Riverine heatwaves (weekly mean water temperature > 90th percentile) are also intensifying, highlighting the growing vulnerability of hydrological systems to climate extremes[71]. Current global lake models and early-warning frameworks remain largely focused on large lakes and river systems. However, small mountain lakes have been shown to exhibit amplified responses to climate variability. Consistent with this, our results demonstrate that small SGLs respond rapidly to climate change, with short-term fluctuations acting as sensitive indicators of environmental variability. Incorporating the seasonal dynamics of small SGLs into existing early-warning frameworks, and developing integrated systems across multiple lake scales, is therefore critical for improving the prediction and response capacity for extreme climate events and abrupt hydrological hazards.*

Methods

Comment 1.13: L404: Please create a figure and include it as an appendix to show the specific acquisition dates of the imagery you used.

Response: Thank you for your comment. Following your suggestion, we have added a table in the supplementary materials that provides a detailed record of the dates of the images used *(For details, please see the Supplementary Information: Table S5, Table S6)*.

Comment 1.14: L432: Why is there an emphasis on 0.5 Landsat pixels here? Your manuscript does not seem to use Landsat imagery.

Response: Thank you for your comment. Although this study did not use Landsat imagery, many existing studies on glacial lake areas are based on Landsat data. We adopted this classification approach primarily to facilitate comparative analysis with previous results, thereby enhancing the comparability and applicability of our dataset. Consequently, the methodological design of this study maintains a certain level of consistency with the Landsat data framework.

Figures

Comment 1.15: Fig.1: Compared with 4 & 5, the font size of 7 & 8 is smaller; please adjust them to be the same size.

Response: Thank you for pointing out this issue. Based on your comment, we have revised Figure 1 and added additional information about the study area *(For details, please see the revised manuscript: Fig .1)*.

Comment 1.16: Fig.2: Very beautiful and informative, but what does the size of the circles in subplots a and b represent-lake size? If so, please indicate this in the title.

Response: Thank you for your comment. As you pointed out, the size of the circles represents the area of the lakes. Following your suggestion, we have clarified this in the figure caption *(For details, please see the revised manuscript: Fig .2)*.

Comment 1.17: Fig.3: Like Fig. 2, it is very beautiful and informative, but some of the tick labels on the left are centered while others are left-aligned. Please make them consistent.

Response: Thank you very much for your comment. Following your suggestion, we have revised Figure 3 accordingly *(For details, please see the revised manuscript: Fig. 3)*.

Comment 1.18: Fig.4: The schematic at the bottom is very nice, but the arrows are too thin and a bit hard to see. Please adjust them appropriately to better convey your meaning. Also, your figure has a scale bar in the top right corner, while the other subfigures do not; please make them consistent.

Response: Thank you very much for your comment. Following your suggestion, we have revised Figure 4 and ensured consistency between the main text figures and the supplementary figures *(For details, please see the revised manuscript: Fig. 4)*.

Comment 1.19: Fig.5: Indicate the location of your study area in the figure.

Response: Thank you for pointing out this issue. Following your suggestion, we have indicated the exact location of the study area in the figure *(For details, please see the revised manuscript: Fig .5)*.

Extended Data Fig. 1&2: The same problem applies to Fig. 4.

Response: Thank you very much for your comment. Following your suggestion, we have revised Extended Data Figs. 1 and 2 accordingly *(For details, please see the revised manuscript: Extended Data Fig. 1&2)*.

Comment 1.20: Extended Data Fig. 7&8: The font sizes of the x-axis labels in the two figures are quite different. Please adjust them appropriately for better visual consistency.

Response: Thank you for pointing out this issue. Following your suggestion, we have adjusted the font size to ensure consistency between the two figures *(For details, please see the revised manuscript: Extended Data Fig. 7&8)*.

Response to the reviewer's comments (Reviewer 2)

General comments:

Comment 2.1: This study presents an analysis of supraglacial lake changes in the Sagarmatha National Park at a high temporal resolution, linking observations to glacier changes (velocity, surface changes). It is suggested that increasing lake sizes since 2022 demonstrates that the lakes respond to climate change variability ahead of this being recorded by sea surface temperatures, and thus lake changes could provide important early warning indicators of climate changes. This therefore represents an important topic of study and the dataset should be of value to the community. However, there are quite a few places where I struggled to follow the overall way this study has been written up, presenting a barrier to these potentially useful results being accessible to the community. The figures are also hard to follow or seemingly lacking important details. I think these substantial issues will need to be considered by the author team.

Response: We sincerely thank you for your valuable comments and for recognizing the importance of our study. After carefully reviewing your suggestions, we have identified the main issues as follows:

(1) the Introduction was not sufficiently focused and did not clearly present the research objectives at an early stage;

(2) some language expressions and technical terminology were inaccurate;

(3) there was confusion in the naming of glacier systems, individual glaciers, and glacier branches;

(4) the InSAR methodology was not described clearly, which made the interpretation of surface deformation terminology difficult to follow.

(5) Issues related to figures and other specific details.

In response to these comments, we have thoroughly revised and refined the manuscript to improve the overall logic, clarity, and readability. All comments have been carefully addressed point by point. **Although some responses are similar in content, we have provided a**

complete reply to each comment for ease of review. In addition, similar comments have been clearly indicated to facilitate selective reading.

Comment 2.2: The paper title indicates that this study is about supraglacial lakes. However, in places, e.g. Figure 4, it appears that it is the connectivity between supraglacial lakes and proglacial lakes that is important – but this is not really explained fully in the introduction. The introduction is largely focused on glacial lakes in general, with no clear distinction made between different types, e.g. moraine-dammed, ice-dammed, proglacial, supraglacial. Whilst all glacial lakes are of course extremely important, for the reasons outlined, I think the explanation of the focus on supraglacial lakes, and their connection to proglacial lakes (if that is important here) needs to come sooner. Clearer use of terminology and a greater focus on supraglacial lakes as the subject of the paper would improve the introduction.

Response: Thank you for your valuable comment. As you pointed out, the Introduction did not sufficiently focus on supraglacial lakes, nor did it clearly explain their relationship with proglacial lakes, resulting in an unclear presentation of the study's main focus. This study primarily focuses on supraglacial lakes, as well as proglacial lakes that evolve from the development and coalescence of supraglacial lakes under glacier retreat. Following your suggestion, we have introduced the concepts of supraglacial and proglacial lakes, along with their interrelationships, earlier in the Introduction, and adopted clearer and more consistent terminology to distinguish between different types of glacial lakes *(Please see the revised manuscript: Lines 75-77, Lines 81-84, Lines 121-122, Lines 153-154, Lines 438-439 (This has also been presented in this response))*. In addition, given that the term "supraglacial lakes" appears frequently in the manuscript (over 100 times), to improve conciseness and readability and to better distinguish it from other related terms, we follow previous studies in abbreviating it as "SGLs," thereby emphasizing its distinction from other types of glacial lakes.

We have revised the paragraph as follows:

Lines 75-77 IN THE REVISED MANUSCRIPT: *[…] which further enhances ice ablation and subglacial melting, causing additional glacier mass loss and retreat, and*

thereby driving further lake expansion, and merging of small supraglacial lakes (SGLs) into larger proglacial lakes

Lines 81-84: *When these water bodies approach local hydrological thresholds, rapid drainage may occur. Concentrated discharge is particularly likely where multiple small SGLs are hydraulically connected through supraglacial drainage networks and collectively feed into a larger proglacial lake, increasing the likelihood of catastrophic GLOFs*[18, 27].

Lines 121-122: *Basnet found that the formation of proglacial lakes accelerates glacier mass loss and promotes glacier retreat, thereby affecting glacier velocity*[38].

Lines 153-154: *For instance, Imja Tsho, a large proglacial lake that developed from five supraglacial ponds in the 1950s*[39], *[…]*

Lines 438-439: *Previous studies*[21,61-63] *suggest that long-term increases in debris cover and pond area accelerate glacier mass loss and enhance the storage capacity of proglacial lakes.*

Comment 2.3: On a similar note, there are a lot of references to "small glacial lakes" in the paper. I am assuming from the paper title that these are "small supraglacial lakes", so this should be explicitly stated throughout to avoid any possible confusion.

Response: Thank you for pointing out this issue. As you noted, there was indeed inconsistency in terminology usage in the manuscript. Following your suggestion, we have carefully reviewed the entire manuscript and made corresponding revisions. **For content related to this study, we consistently use the term "supraglacial lake (SGL)"; when citing previous studies, we retain the original terminology used in the sources,** in order to avoid misinterpretation of the original research.

Comment 2.4: Surface deformation is used throughout the paper, but I am not sure it is the best term (although it is not easy to understand exactly what it is referring to). Is this simply

downglacier surface movement as the ice flows? Or is there a vertical element to this? In some places this refers to moraine ridges deformation. A clearer explanation of this term is needed, or perhaps better still, use of another term that is less ambiguous.

Response: Thank you for pointing out this issue. First, the term surface deformation is a standard academic expression in InSAR studies *(e.g., Unraveling the relationship between surface deformation and groundwater dynamics in karst terrains using multi-source remote sensing data. https://doi.org/10.1016/j.jhydrol.2025.134129; Prominent creep characteristics of thermokarst landslides on central Tibetan Plateau under climate warming conditions. https://doi.org/10.1016/j.catena.2024.108457)*. The main reason for the confusion is that we did not clearly distinguish in the Methods section between the glacier surface velocity derived from Sentinel-2 (representing the movement of the glacier surface driven by ice flow) and the surface deformation in the glacier terminus area derived from Sentinel-1 (which is more sensitive to vertical displacement). Specifically, **Sentinel-2** primarily captures **surface velocity** along the glacier centerline, covering the glacier from its head to its terminus **(The commonly used term is "velocity")**, whereas **Sentinel-1** mainly reflects **surface deformation** in the ice-free areas at the glacier terminus, particularly in regions associated with proglacial lakes **(The commonly used term is "surface deformation")**. To avoid ambiguity, we have revised the Methods section to clearly describe this workflow and research *(Please see the revised manuscript: Lines 553-556, Lines 557-576 (This has also been presented in this response))*.

We have revised the paragraph as follows (Comments 2.4 & 2.28 & 2.61–2.64 are similar issues)

> ***Lines 553-556 IN THE REVISED MANUSCRIPT:*** *Therefore, we applied Sentinel-2 imagery to monitor the seasonal dynamics of glacier flow (glacier surface velocity) and used 233 Sentinel-1 synthetic aperture radar (SAR) images to map terminal moraine deformation at the glacier termini.*
>
> ***Lines 557-576:*** *The SBAS-InSAR algorithm is a time-series InSAR approach that generates a set of small-baseline interferograms using a multi-master strategy with*

appropriately defined spatiotemporal baseline thresholds, enhancing interferometric coherence and enabling millimeter- to centimeter-scale monitoring of surface deformation. N+1 single-look complex (SLC) SAR images acquired over the study area at consecutive time points $t0, \cdots, tN$ *are processed by selecting a single master image, to which all other images are co-registered and a unified reference grid is established. Differential interferograms are generated from the SLC images, and topographic phase is removed using an external digital elevation model (e.g., AW3D 30 m). Flat and highly coherent stable points (e.g., bedrock) are used as zero-displacement reference points. Following filtering and phase unwrapping, continuous phase time series are obtained for each coherent target, comprising deformation, residual topography, atmospheric, orbital, and noise components. Orbital errors are corrected via least-squares fitting, residual topography is estimated from the linear relationship between perpendicular baseline and elevation, and atmospheric contributions are separated using temporal high-pass and spatial low-pass filtering. Singular value decomposition (SVD) is then applied to convert the multi-master interferogram set into a single-reference time series[74], with unwrapped phase converted into LOS displacement. In the LOS direction, negative values indicate motion away from the satellite, whereas positive values indicate motion toward it. In this study, these deformation patterns manifest as subsidence and deposition in the glacier terminus zone, respectively. Given that InSAR measurements are acquired along the LOS direction and based on the geometric projection relationship, the vertical displacement can be approximated as* $dvertical = dLOS/COS\theta$, *where* θ *is the incidence angle[75]. All processing was performed using GAMMA software.*

Comment 2.5: A better location map is needed to clearly show the spatial context of the study area glaciers and lakes – Figure 1 does not do this.

Response: Thank you for pointing out this issue. Following your suggestion, we have added location information to Fig. 1, including labels for representative peaks in the study area, as well as the locations and names of glaciers, and the names of typical lakes. Meanwhile, since Fig. 1 is presented as a combination of a 3D terrain map and a conceptual diagram, it cannot fully display all relevant information. Therefore, we have added a planar map in the Extended

Data Figures, numbered as Extended Data Fig. 1, showing rivers, settlements, debris-covered glaciers, and clean glaciers as a supplement to Fig. 1, to ensure that readers can fully understand the study area *(Please see the revised manuscript: Fig. 1, Extended Data Fig. 1 (Fig. 1 and Extended Data Fig. 1 are also presented in this response))*. Regarding the conceptual aspects, we mainly referred to Fig. 1 in Xu et al. *(Widespread societal and ecological impacts from projected Tibetan Plateau Lake expansion, https://doi.org/10.1038/s41561-024-01446-w), as shown in the figure below*. Furthermore, detailed explanations have been added to the figure caption of Fig. 1, including the meaning of the meteorological labels and the sources of the data *(For more detailed and specific responses regarding Fig. 1, please refer to our response to* ***Comment 2.71****)*.

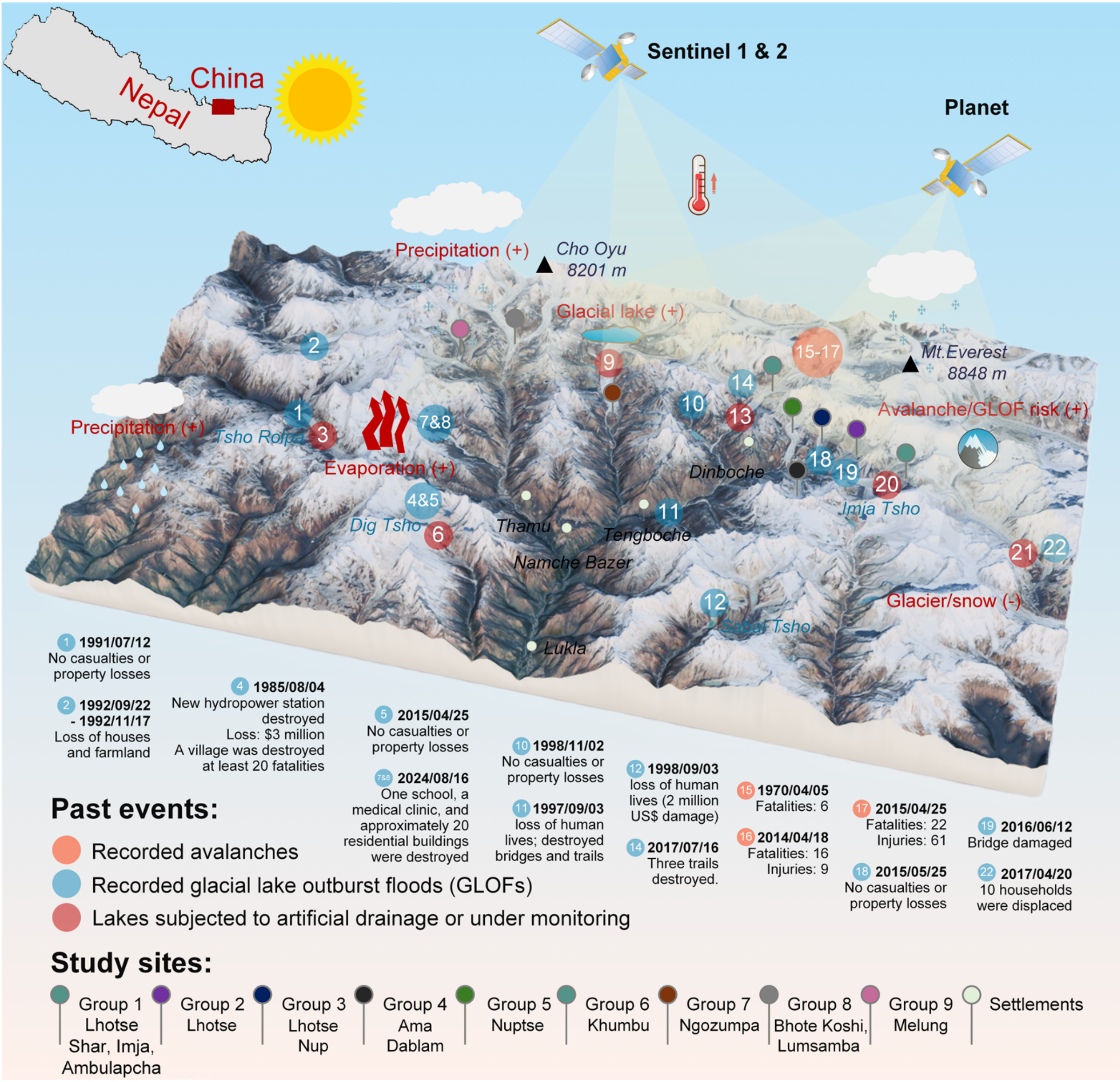

Fig. 1 | Diagram illustrating lake evolution and geological hazards in Sagarmatha National Park. The red labels and text represent the climate-related risks facing this region, namely that under global warming, increased snowfall/rainfall and the melting of glaciers and snowpack lead to the expansion of lake boundaries and heightened risks of GLOFs and avalanches. The numbers in the figure indicate the locations and associated hazard losses of past GLOFs, avalanches, and manually monitored lakes (Data source: Table S1). Glaciers sharing the same drainage outlet were grouped together and labeled as Groups 1–9. The glaciers included in each group are listed as follows: **Group 1** Lhotse Shar, Imja, and Ambulapcha; **Group 2** Lhotse; **Group 3** Lhotse Nup; **Group 4** Ama Dablam; **Group 5** Nuptse; **Group 6** Khumbu; **Group** 7 Ngozumpa; **Group 8** Bhote Koshi and Lumsamba; **Group 9** Melung Glaciers. Extended Data Fig. 1 serves as a supplement to this figure, documenting the spatial information of clean glaciers, debris-covered glaciers, and runoff.

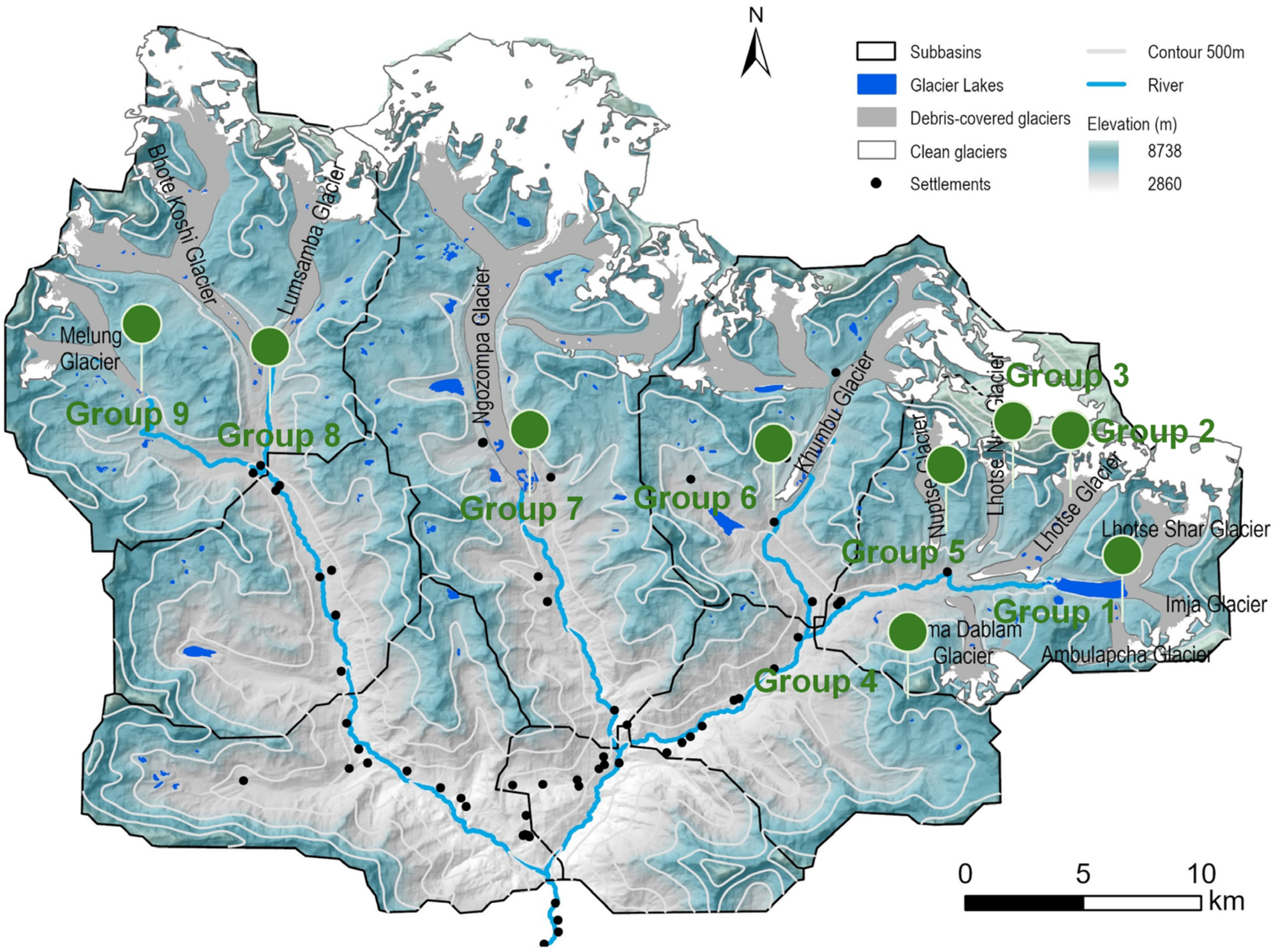

Extended Data Fig. 1 | Spatial distribution and classification of glaciers. Glaciers sharing the same drainage outlet were grouped together and labeled as Glacier Group 1–9. Except for Glacier Groups 1 and 8, which contain two and three individual glaciers, respectively, each of

the remaining groups consists of a single glacier. The details are as follows: **Group 1** Lhotse Shar、Imja and Ambulapcha; **Group 2** Lhotse; **Group 3** Lhotse Nup; **Group 4** Ama Dablam; **Group 5** Nuptse; **Group 6** Khumbu; **Group 7** Ngozumpa; **Group 8** Bhote Koshi and Lumsamba and **Group 9** Melung Glaciers**.**

[Figure Redacted]

Comment 2.6: Throughout the paper and figures, the labelling of glaciers, glacier systems, the mix of glacier numbers/glaciers systems numbers/glacier names – this is all very confusing and could be much more straightforward.

Response: Thank you very much for pointing out this issue. As you noted, the manuscript involves multiple classification and labeling schemes, including glacier numbers, glacier system numbers, and glacier names, which can indeed lead to confusion. The main reason for this issue is that some glaciers have branched structures, resulting in inconsistencies in the classification system used for different types of data analysis. Therefore, we have redefined and unified the classification rules: **when upstream glaciers share a single drainage outlet, they are treated as one glacier system and designated as Group 1, Group 2, …, Group 9.**

Most glacier groups consist of a single glacier, such as Group 2 (Lhotse glacier), Group 3 (Lhotse Nup glacier), Group 4 (Ama Dablam glacier), Group 5 (Nuptse glacier), Group 6 (Khumbu glacier), Group 7 (Ngozumpa glacier), and Group 9 (Melung glacier). Some groups include two or more glaciers, such as Group 1 (Lhotse Shar, Imja, and Ambulapcha glacier) and Group 8 (Bhote Koshi and Lumsamba glacier)

Regarding the glacier numbering in Fig. 2, due to the branched structure of many glaciers, we redefined the numbering scheme to track lake dynamics across different glacier branches. **Each branch unit was renumbered using a group-consistent labeling system, replacing the original 1–19 numbering scheme. For example, Group 9 contains three branches, labeled as Group 9-1, 9-2, and 9-3, whereas Group 2 has no branches and therefore remains as Group 2.** To clarify the correspondence between each number and the glacier branches, we have added four supplementary figures in the Supplementary Information, which provide a detailed mapping between the numbering system and the glacier branches.

Meanwhile, our team has carefully discussed your comment regarding the "difficulty in understanding the presentation". As this study involves a large number of glaciers and glacier branches, frequent use of individual glacier names in the manuscript would increase reading complexity and reduce readability. **Therefore, in the manuscript, we consistently use glacier**

group numbers when presenting specific analyses (e.g., "the total area of Glacier Group 1 expanded …" instead of "the total area of Lhotse Shar, Imja and Ambulapcha Glaciers expanded …"). In the figures, we also uniformly adopt the nine glacier group labels (Group 1–9), while glacier names are only provided in the figure captions. This approach enables readers to refer to the corresponding figures and captions when needed, thereby easily linking each glacier group with its constituent glaciers and improving overall readability.

Note: The above provides an explanation. To improve clarity, we have added Extended Data Table 1, which allows readers to quickly understand the classification scheme at a glance.

We have revised the paragraph as follows (This is a similar issue to Comment 2.6 & 2.35 & 2.46 & 2.50):

Extended Data Table 1 | Correspondence between Glacier Systems, Glacier Names, Glacier Branches, and Lake Numbers in Each Glacier Group

Glacier System	Glacier Name	Glacier Branch (Fig.2)	Number of Lakes
Group 1	Ambulapcha	1-1	88
	Imja	1-2	
	Lhotse Shar	1-3	
Group 2	Lhotse	2	114
Group 3	Lhotse Nup	3	42
Group 4	Ama Dablam	4	42
Group 5	Nuptse	5	66
Group 6	Khumbu	6	144
Group 7	Ngozumpa	7-1	391
		7-2	
		7-3	
Group 8	Bhote Koshi	8-2	502
		8-3	
		8-4	
	Lumsamba	8-1	
		8-5	
Group 9	Melung	9-1	104
		9-2	
		9-3	

Glaciers sharing the same outlet were classified into the same group. The nine groups together contain 12 independent glaciers. Due to branching structures in some glaciers, a total of 19 glacier branch units were identified. The spatial correspondence between the identifiers and specific glacier branches is shown in Fig. 2a and Figs.S1–S4.

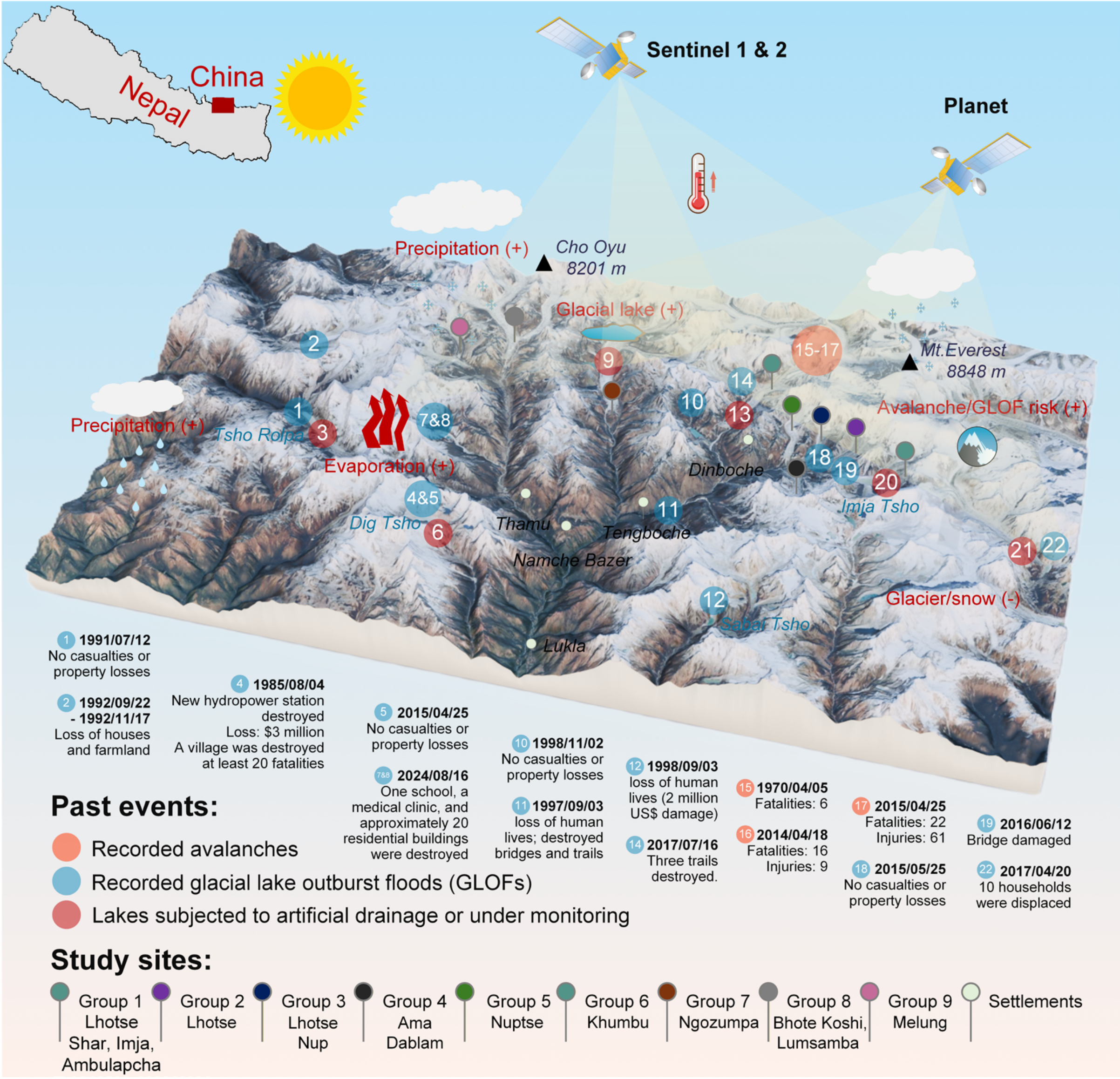

Fig. 1 | Diagram illustrating lake evolution and geological hazards in Sagarmatha National Park. The red labels and text represent the climate-related risks facing this region, namely that under global warming, increased snowfall/rainfall and the melting of glaciers and snowpack lead to the expansion of lake boundaries and heightened risks of GLOFs and avalanches. The numbers in the figure indicate the locations and associated hazard losses of past GLOFs, avalanches, and manually monitored lakes (Data source: Table S1). Glaciers sharing the same drainage outlet were grouped together and labeled as Groups 1–9. The glaciers included in each group are listed as follows: **Group 1** Lhotse Shar, Imja, and Ambulapcha; **Group 2** Lhotse; **Group 3** Lhotse Nup; **Group 4** Ama Dablam; **Group 5** Nuptse; **Group 6** Khumbu; **Group** 7 Ngozumpa; **Group 8** Bhote Koshi and Lumsamba; **Group 9** Melung Glaciers. Extended Data Fig. 1 serves as a supplement to this figure, documenting the spatial

information of clean glaciers, debris-covered glaciers, and runoff.

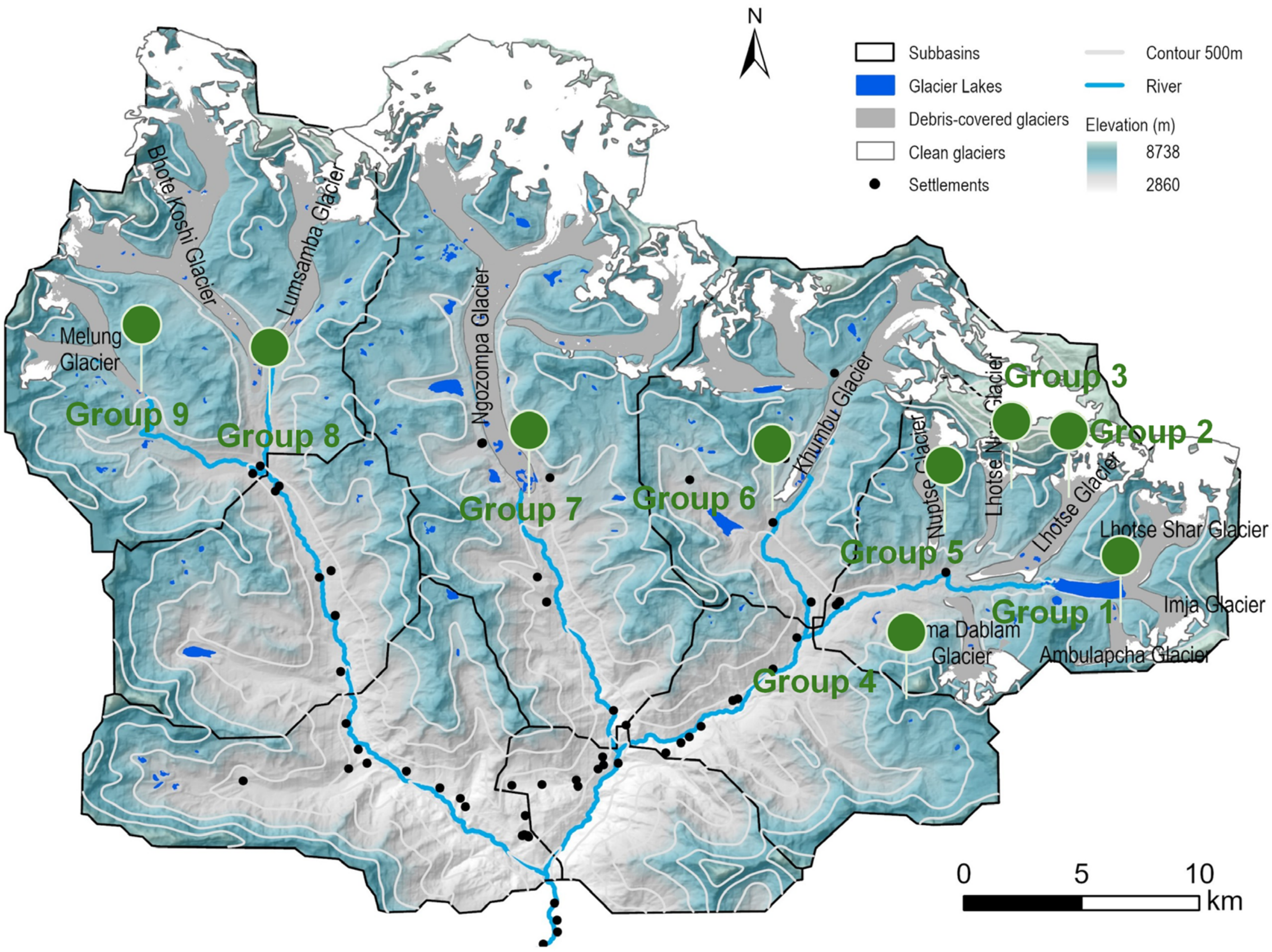

Extended Data Fig. 1 | Spatial distribution and classification of glaciers. Glaciers sharing the same drainage outlet were grouped together and labeled as Glacier Group 1–9. Except for Glacier Groups 1 and 8, which contain two and three individual glaciers, respectively, each of the remaining groups consists of a single glacier. The details are as follows: **Group 1** Lhotse Shar、Imja and Ambulapcha; **Group 2** Lhotse; **Group 3** Lhotse Nup; **Group 4** Ama Dablam; **Group 5** Nuptse; **Group 6** Khumbu; **Group 7** Ngozumpa; **Group 8** Bhote Koshi and Lumsamba and **Group 9** Melung Glaciers**.**

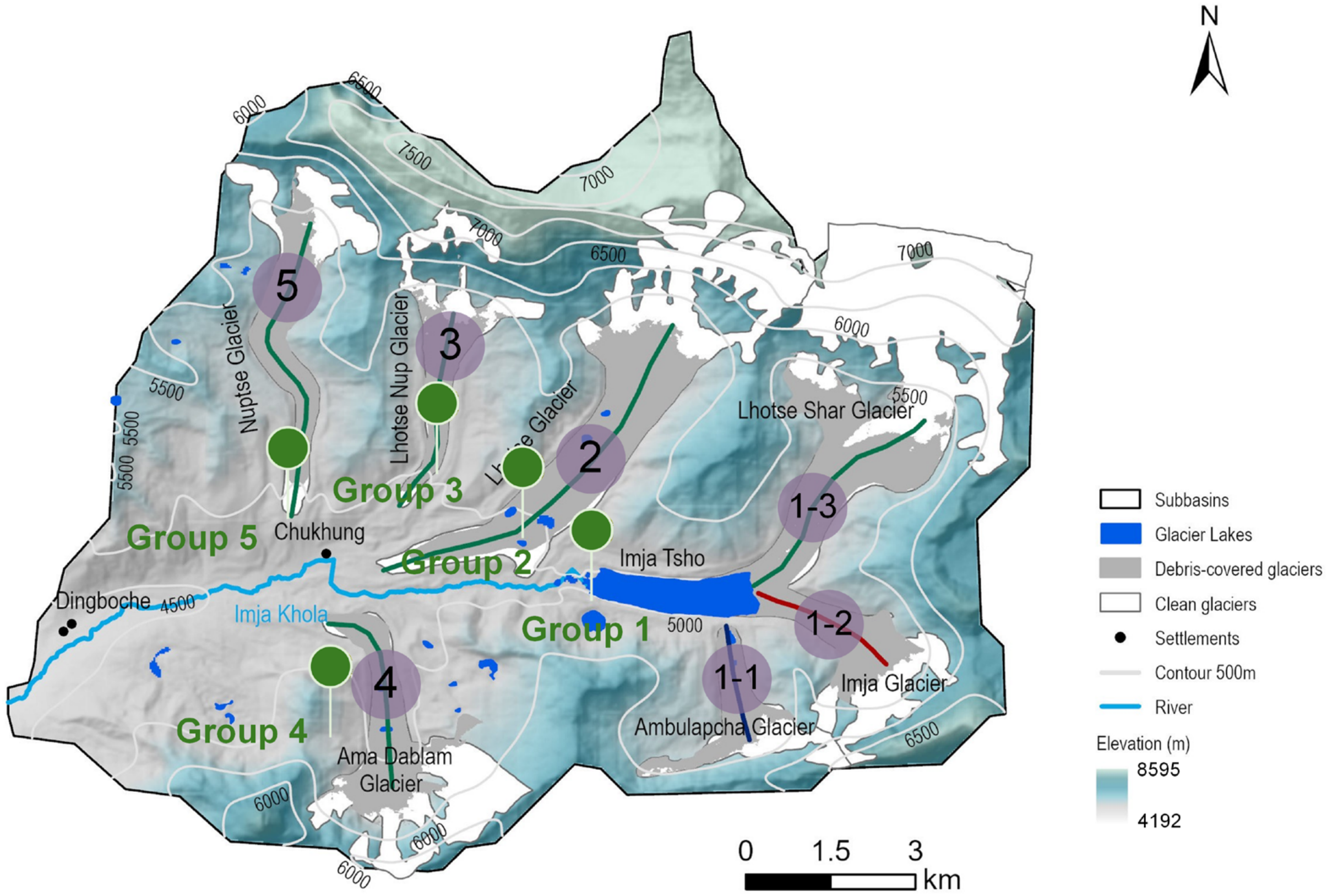

Fig. S1 | Criteria for glacier system classification and spatial distribution of glaciers. Group 1-5 represent the spatial distribution of five glacier systems, and the numbers correspond to the glacier branches in Fig. 2. The relationships are as follows: **Group 1** Lhotse Shar Glacier (circled 1-1), Imja Glacier (circled 1-2), and Ambulapcha Glacier (circled 1-3); **Group** 2 Lhotse Glacier (circled 2); **Group** 3 Lhotse Nup Glacier (circled 3); **Group** 4 Ama Dablam Glacier (circled 4); and **Group** 5 Nuptse Glacier (circled 5).

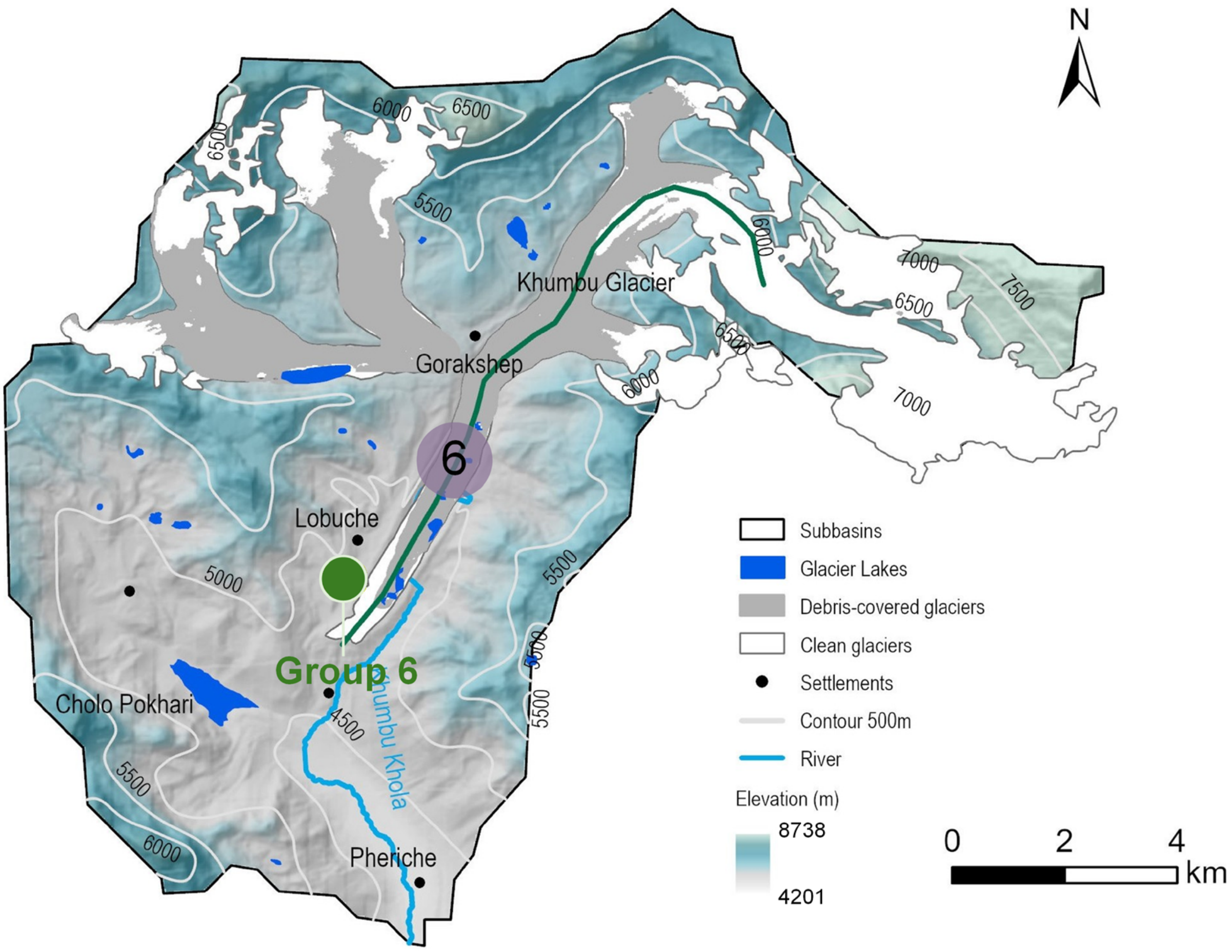

Fig. S2 | Criteria for glacier system classification and spatial distribution of glaciers. Group 6 represents the spatial distribution of the glacier group, and the number corresponds to the glacier branch in Fig. 2. The correspondence is as follows: **Group 6** Khumbu Glacier (circled 6).

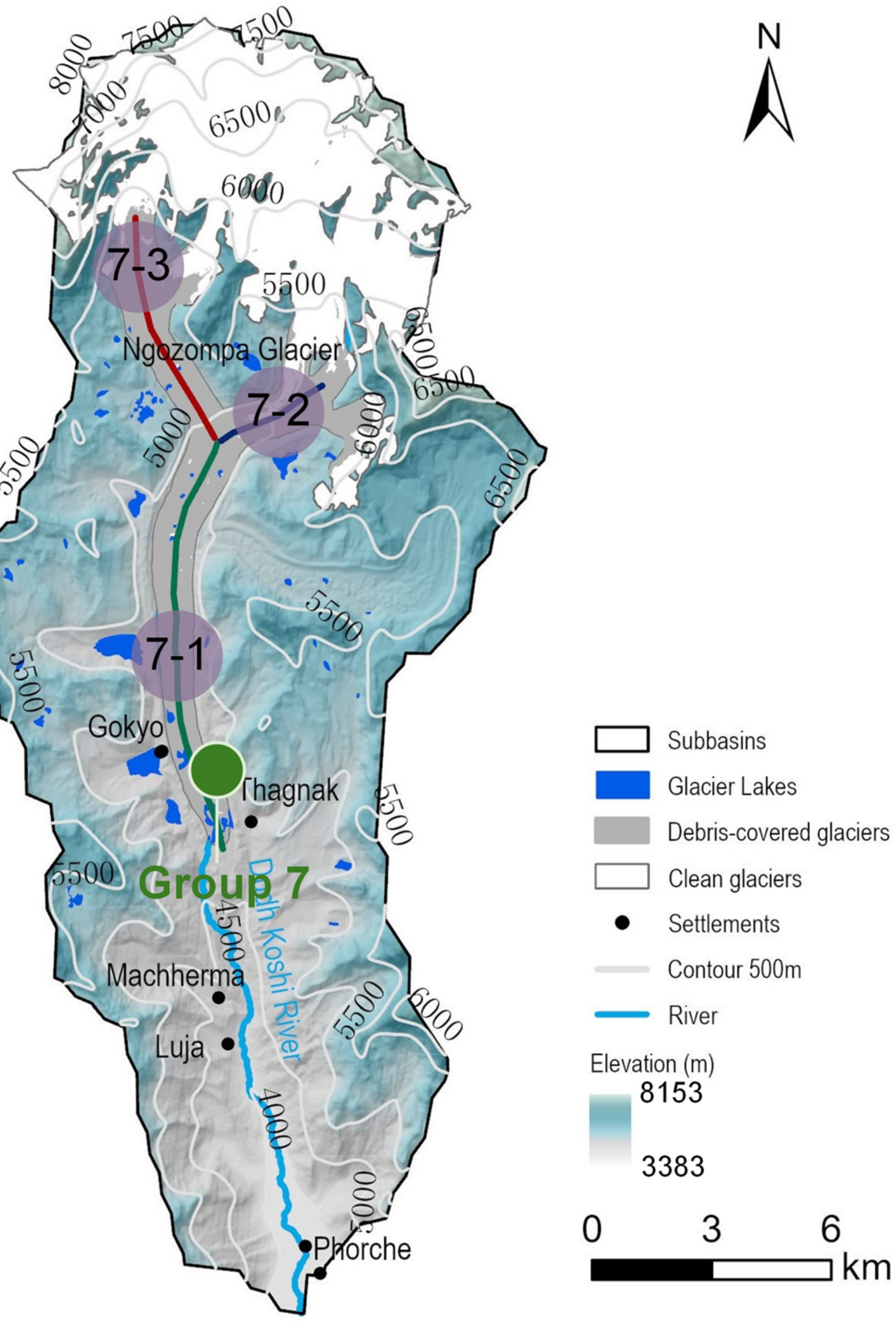

Fig. S3 | Criteria for glacier system classification and spatial distribution of glaciers. Group 7 represents the spatial distribution of the glacier group, and the number corresponds to the glacier branch in Fig. 2. The correspondence is as follows: **Group** 7 Ngozumpa Glacier (circled 7-1, 7-2 and 7-3).

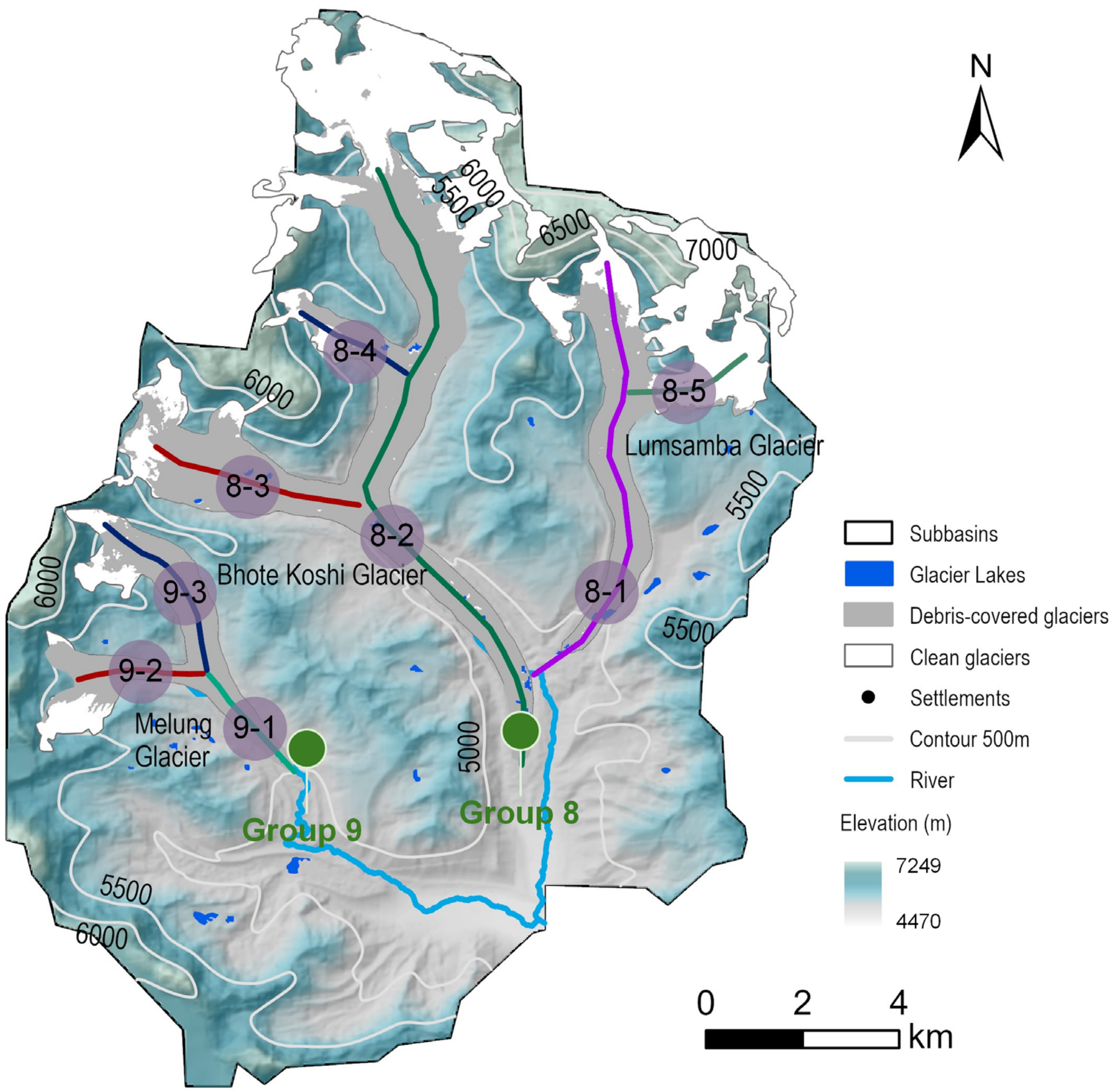

Fig. S4 | Criteria for glacier system classification and spatial distribution of glaciers. Group 8-9 represents the spatial distribution of the glacier group, and the number corresponds to the glacier branch in Fig. 2. The correspondence is as follows: **Group** 8 Bhote Koshi Glacier (circled 8-2, 8-3 and 8-4) and Lumsamba Glacier (circled 8-1 and 8-5). **Group** 9 Melung Glacier (circled 9-1, 9-2 and 9-3)

Specific comments:

Abstract

Comment 2.7: Line 33: It would be useful to state where in HMA the Sagarmatha region is – perhaps using the Hindu Kush Himalayan Monitoring and Assessment Programme (HIMAP) sub-regions: https://link.springer.com/chapter/10.1007/978-3-319-92288-1_7.

Response: Thank you very much for your valuable suggestion. According to the HIMAP, the Sagarmatha region is located in the Eastern Himalaya. The relevant information has been added to the revised manuscript.

Comment 2.8: Lines 42-43: "The presence of proglacial lakes governs the surface deformation rate at glacier termini, with mean cumulative displacement along the radar line-of-sight up to 46.84 cm." – this sentence seems out of place here? Before now the focus has been on supraglacial lakes, so the introduction of proglacial lakes here seems odd. This should be integrated into the text more clearly or removed.

Response: Thank you very much for pointing out this issue. As you noted, this sentence does feel somewhat abrupt here. After careful consideration, we found that the content in question is primarily presented in the supplementary figures, and given the constraints of abstract length and precision, we believe that removing the sentence is more appropriate than expanding it. Accordingly, we have chosen to delete this sentence in response to your suggestion.

Comment 2.9: Lines 43-44: "In High Mountain Asia, the expansion of supraglacial lakes is expected to continue." – why, and on what timescale is this suggestion being made?

Response: Thank you for your comment. This section mainly addresses the finding that, among the nine glacier groups in our study, eight exhibit an increasing trend (Fig. 3 and Table S2). In addition, according to Huss et al. *(Global-scale hydrological response to future, glacier mass loss (2018). https://doi.org/10.1038/s41558-017-0049-x)*, glacier meltwater supply in High

Mountain Asia is expected to continue increasing and will likely reach its peak around 2050. Therefore, we provide a summary here.

Comment 2.10: Lines 44-46: "…they can be incorporated into the global early-warning system for hydrological pulses and extreme climate" – is this early-warning system something that already exists, and is it to detect both hydrological pulses and extreme climate? I think there could be more clarity here.

Response: Thank you very much for your insightful comment. Regarding the "global early-warning system for hydrological pulses and extreme climate," there are indeed existing research frameworks, models, and systems *(e.g., Lake surface water temperature in 2024 (https://doi.org/10.1038/s43017-025-00654-1), Persistent river heatwaves are emerging worldwide under climate change (https://doi.org/10.1038/s41467-025-66868-5), and Lakes are experiencing more severe heatwaves than the atmosphere (https://doi.org/10.1038/s43247-025-02907-9))*. However, these studies have primarily focused on large lakes and river systems. In contrast, small lakes, particularly supraglacial lakes, have received comparatively limited attention. Previous studies *(e.g., Elevation-dependent climate change in mountain environments (https://doi.org/10.1038/s43017-025-00740-4))* have shown that temperature changes in high-altitude regions tend to exhibit amplified responses to global warming. Therefore, small supraglacial lakes may play an important role in improving early-warning systems for hydrological pulses and extreme climate. This also represents one of the key implications of our study. We acknowledge, however, a limitation of this work. Our analysis is based on a representative regional case rather than a global assessment. Accordingly, our conclusions regarding climate responses are primarily descriptive of observed patterns, rather than generalizable definitions at the global scale. Future work will extend this analysis to supraglacial lakes at the global scale. This effort is currently underway, although it is computationally intensive and requires substantial time to complete. Such analyses will help to further clarify the role of small supraglacial lakes in global early-warning systems. Due to space limitations in the abstract, a more detailed discussion and outlook have been included in the

Discussion section *(For details, please refer to the revised manuscript: Lines 466-479 (This has also been presented in this response))*.

We have revised the paragraph as follows:

> ***Lines 466-479 IN THE REVISED MANUSCRIPT:*** *Lakes and hydrological systems are highly sensitive to long-term climate warming. With the increasing frequency and intensity of extreme events worldwide, lake and river ecosystems are emerging as important indicators for the early warning of global hazards. Previous studies[68-70] suggest that the rapid rise in lake surface water temperature is closely linked to intensified summer heat extremes, with anomalies primarily driven by surface air temperature and further modulated by local lake characteristics and ice-cover dynamics. Riverine heatwaves (weekly mean water temperature > 90th percentile) are also intensifying, highlighting the growing vulnerability of hydrological systems to climate extremes[71]. Current global lake models and early-warning frameworks remain largely focused on large lakes and river systems. However, small mountain lakes have been shown to exhibit amplified responses to climate variability. Consistent with this, our results demonstrate that small SGLs respond rapidly to climate change, with short-term fluctuations acting as sensitive indicators of environmental variability. Incorporating the seasonal dynamics of small SGLs into existing early-warning frameworks, and developing integrated systems across multiple lake scales, is therefore critical for improving the prediction and response capacity for extreme climate events and abrupt hydrological hazards.*

Introduction

Comment 2.11: Line 53: “glacier collapses” – suggest “collapses or detachments”.

Response: Thank you for your valuable suggestion. Based on your comment, we have made the corresponding revisions.

Comment 2.12: Line 54: add citations to global studies of glacier collapses/detachments and GLOFs here: for example, Kääb et al. (2021: https://doi.org/10.5194/tc-15-1751-2021) and Taylor et al. (2023: https://www.nature.com/articles/s41467-023-36033-x)

Response: Thank you for your comment. Following your suggestion, we have added the references you recommended in the appropriate sections.

Comment 2.13: Lines 58-59: "In recent years, advances in optical and radar remote sensing have accelerated investigations into seasonal to interannual variations in glacier flow and glacial lake dynamics" – add citations to one or two example studies here.

Response: We sincerely thank you for your valuable suggestions. In response, we have cited recently published studies that are highly relevant to our work and summarized their main findings to further support and strengthen our arguments *(For details, please see the revised manuscript: Lines 52-56 (This has also been presented in this response) and Seasonal dynamics of Earth's glaciers and ice sheets, https://doi.org/10.1126/science.adx6654)*.

We have revised the paragraph as follows:

> ***Lines 52-56 IN THE REVISED MANUSCRIPT:*** *A global study shows that basal hydrological systems not only modulate seasonal glacier flow but also that seasonal variations are weakly yet significantly correlated with interannual variations[15], providing critical insights into seasonal anomalies in glaciers and glacial lakes and their associated extreme hazards under global warming.*

Comment 2.14: Line 78: Suggest adding "supraglacial ponds" so it is unambiguous.

Response: Thank you for your suggestion. Following your advice, we have added "supraglacial ponds" to avoid ambiguity.

Comment 2.15: Lines 79: "near-surface and shallow subsurface drainage pathways" – does this mean supraglacial and englacial drainage pathways? It is not clear what the difference

between near-surface and shallow subsurface is here, so the use of clearer terminology would help.

Response: Thank you very much for your professional comments. We have carefully re-examined the context of the relevant references. As you correctly pointed out, “near-surface or shallow englacial drainage” refers to “supraglacial and englacial drainage pathways.” This interpretation is also clearly explained in the relevant literature and represents a more standard and widely accepted terminology. In response to your suggestion, we have made the corresponding revisions *(For details, please see the revised manuscript: Lines 73-77 (This has also been presented in this response)*.

We have revised the paragraph as follows:

> ***Lines 73-77 IN THE REVISED MANUSCRIPT:*** *Debris-covered glaciers with ponds often transport meltwater downstream through supraglacial drainage and englacial drainage pathways, which further enhances ice ablation and subglacial melting, causing additional glacier mass loss and retreat, and thereby driving further lake expansion, and merging of small supraglacial lakes (SGLs) into larger proglacial lakes[18,27].*

Comment 2.16: Lines 82-90: “Glacial retreat has lead to…” to “…reservoirs and dams.” – these sentences seem somewhat tangential to the focus of this paper on supraglacial lakes. As noted elsewhere, I think the introduction needs to get to the focus of the paper on supraglacial lakes on debris-covered glaciers much quicker – why are these important to study?

Response: Thank you for pointing out this issue. After re-examination, we agree that the original text was not well aligned with the main focus of this study. Therefore, we have completely rewritten the third and fourth paragraphs. The revised version focuses on the formation and evolutionary characteristics of supraglacial lakes on debris-covered glaciers, clarifies the importance of studying supraglacial lakes, and highlights their widespread distribution and research significance in the Himalayan region. It also emphasizes the typical characteristics and representative cases of outburst events of such lakes. In addition, we have

added a figure to show the spatial distribution of debris-covered glaciers in the study area *(For details, please see the revised manuscript: Lines 77-95, Lines 96-113, Extended Data Fig. 1 (This has also been presented in this response))*.

We have revised the paragraph as follows:

> ***Lines 77-95 IN THE REVISED MANUSCRIPT:*** *[...] This dynamic hydrological connectivity is frequently modulated by pronounced seasonal variability in SGLs, as evidenced by their marked seasonal fluctuations in area and depth on HMA glaciers, which typically reach their maximum extent during the mid-monsoon period before subsequently declining[28]. During this phase, debris redistribution and evolving surface topography drive the migration and dynamic reorganization of SGLs. When these water bodies approach local hydrological thresholds, rapid drainage may occur. Concentrated discharge is particularly likely where multiple small SGLs are hydraulically connected through supraglacial drainage networks and collectively feed into a larger proglacial lake, increasing the likelihood of catastrophic GLOFs[18,27]. A typical example by Miles[27], based on Planet imagery, documents an SGL outburst on the Khumbu Glacier. Multiple small SGLs rapidly coalesced prior to sudden drainage, reshaping the glacier surface and triggering debris-flow erosion and landslides within the glacier hydrological system, forming a cascading hazard chain. This process occurs over very short timescales. However, limitations in satellite revisit frequency and spatial resolution mean that uncertainties associated with such small SGL drainage and related debris-flow hazards are often overlooked[29]. In HMA, 13 – 36% of glacierized areas are covered by surface debris and feature SGLs, storing water equivalent to roughly 23% of the average daily observed runoff18. Frequent hydrological activity and lake evolution are key features of SGLs systems under environmental forcing. During these processes, debris flows and SGL outbursts driven by hydrological pulses are often difficult to capture using satellite observations. Consequently, the frequency of GLOFs is likely to have been substantially underestimated[18, 30, 31].*

Lines 96-113: *In Sagarmatha National Park, the ablation zones of glaciers are almost entirely covered by continuous debris, which plays a key role in modulating glacier responses to climatic forcing[18,32]. During the retreat of debris-covered glaciers, debris can either accelerate or retard ice melt depending on its thickness; together with the dynamic glacier surface topography, this promotes enhanced differential ablation and the formation of SGLs, thereby increasing the risk of mountain hazards[18]. Over the past few decades, more than 16 GLOFs and ice avalanches have been reported in the region, resulting in over 340 deaths and missing persons[9,33]. For example, the 1985 Dig Tsho GLOF alone destroyed 14 bridges and the nearly completed Namche hydropower station. To mitigate the potential outburst risk of large moraine-dammed lakes, long-term monitoring and engineering interventions have been implemented, successfully preventing lake outburst events to some extent[34,35]. However, small SGLs, owing to their rapid evolution and unpredictable seasonal variability, are increasingly becoming a major source of GLOFs; since 2000, five of the seven recorded GLOF events in the region have originated from SGLs[9]. Such frequent and hard-to-monitor geohazards are rarely included in global compilations of glacial lake outburst events (Fig. 1). As critical indicators of global climate change, seasonal fluctuations in lake area are considered one of the most sensitive characteristics of small SGLs. Some case studies show that, prior to reaching critical outburst thresholds, some SGLs can expand rapidly over short timescales, with surface area increases exceeding eightfold, and reaching up to 70-fold over a complete seasonal expansion cycle[36]. However, due to the trade-off between spatial and temporal resolution in single-sensor satellite data, such short-term fluctuations are often overlooked[30]. […]*

Comment 2.17: Lines 90-91: "Another important factor is the limitation of satellite observations, which do not account for the uncertainties associated with the drainage of small lakes and debris flow hazards." – this needs to be explained more clearly. Why is this? What is the link between small lakes and debris flow hazards?

Response: We sincerely apologize for the lack of clarity in our previous explanation. Following your suggestion, we have revised this section to emphasize that the filling and drainage processes of small supraglacial lakes and ponds typically occur over very short timescales. However, due to limitations in satellite spatial resolution and revisit frequency, the uncertainties associated with small supraglacial lake drainage and related debris-flow hazards are difficult to effectively monitor. In addition, we have added a real case study to illustrate the link between the drainage of small supraglacial lakes and associated debris-flow hazards, and we have also included an example showing that the area of small supraglacial lakes exhibits strong short-term variability *(For details, please see the revised manuscript: Lines 82-90, Lines 110-112 (This has also been presented in this response))*.

We have revised the paragraph as follows:

> ***Lines 82-90 IN THE REVISED MANUSCRIPT:*** *Concentrated discharge is particularly likely where multiple small SGLs are hydraulically connected through supraglacial drainage networks and collectively feed into a larger proglacial lake, increasing the likelihood of catastrophic GLOFs[18,27]. A typical example by Miles[27], based on Planet imagery, documents an SGL outburst on the Khumbu Glacier. Multiple small SGLs rapidly coalesced prior to sudden drainage, reshaping the glacier surface and triggering debris-flow erosion and landslides within the glacier hydrological system, forming a cascading hazard chain. This process occurs over very short timescales. However, limitations in satellite revisit frequency and spatial resolution mean that uncertainties associated with such small SGL drainage and related debris-flow hazards are often overlooked[29].*
>
> ***Lines 110-112:*** *Some case studies show that, prior to reaching critical outburst thresholds, some SGLs can expand rapidly over short timescales, with surface area increases exceeding eightfold, and reaching up to 70-fold over a complete seasonal expansion cycle[30].*

Comment 2.18: Line 92: “feature glacial lakes” – state that these are supraglacial lakes for clarity.

Response: Thank you very much for pointing out the issues in our manuscript. Based on your suggestion, we have explicitly stated that these are "supraglacial lakes (SGLs)."

Comment 2.19: Lines 92-93: The link between these two sentences is not clear because the link between supraglacial lakes and GLOFs has not really been clearly articulated to this point. See elsewhere for comments on the need for a clearer focus in the introduction on supraglacial lakes, particularly small supraglacial ponds on debris-covered glaciers, and GLOF hazards.

Response: Thank you for your suggestion. Following your advice, we have further emphasized the relationship between supraglacial lakes on debris-covered glaciers and GLOFs, as well as their associated hazard patterns, and illustrated this with representative cases from the study area. This revision highlights the distinct hazard characteristics of supraglacial lakes on debris-covered glaciers. On this basis, we further clarify that "in High Mountain Asia (HMA), 13–36% of glacierized areas are covered by surface debris and feature glacial lakes," which indicates the widespread nature of such hazards and underscores the necessity of this study, thereby strengthening the logical connection between these statements *(For details, please see the revised manuscript: Lines 77-90 (This has also been presented in this response))*.

We have revised the paragraph as follows:

> ***Lines 77-90 IN THE REVISED MANUSCRIPT:*** *This dynamic hydrological connectivity is frequently modulated by pronounced seasonal variability in SGLs, as evidenced by their marked seasonal fluctuations in area and depth on HMA glaciers, which typically reach their maximum extent during the mid-monsoon period before subsequently declining[28]. During this phase, debris redistribution and evolving surface topography drive the migration and dynamic reorganization of SGLs. When these water bodies approach local hydrological thresholds, rapid drainage may occur. Concentrated discharge is particularly likely where multiple small SGLs are hydraulically connected through supraglacial drainage networks and collectively feed into a larger proglacial lake, increasing the likelihood of catastrophic GLOFs[18,27]. A typical example by Miles[27], based on Planet imagery, documents an SGL outburst on the Khumbu Glacier. Multiple small*

SGLs rapidly coalesced prior to sudden drainage, reshaping the glacier surface and triggering debris-flow erosion and landslides within the glacier hydrological system, forming a cascading hazard chain. This process occurs over very short timescales. However, limitations in satellite revisit frequency and spatial resolution mean that uncertainties associated with such small SGL drainage and related debris-flow hazards are often overlooked[29].

Comment 2.20: Lines 95-99: The sentences here need rephrasing. Suggest adding key citations to studies on the listed hazards.

Response: Thank you for your comment. Following your suggestions, we have completely rewritten this paragraph. In addition, based on other related comments, we further enhanced the background information of the study area and the potential hazard risks, and added details on the specific impacts, frequency of the reported hazards, as well as the corresponding references *(For details, please see the revised manuscript: Lines 96-107 (This has also been presented in this response))*.

We have revised the paragraph as follows:

Lines 96-107 IN THE REVISED MANUSCRIPT: *In Sagarmatha National Park, the ablation zones of glaciers are almost entirely covered by continuous debris, which plays a key role in modulating glacier responses to climatic forcing[18,32]. During the retreat of debris-covered glaciers, debris can either accelerate or retard ice melt depending on its thickness; together with the dynamic glacier surface topography, this promotes enhanced differential ablation and the formation of SGLs, thereby increasing the risk of mountain hazards[18]. Over the past few decades, more than 16 GLOFs and ice avalanches have been reported in the region, resulting in over 340 deaths and missing persons[9,33]. For example, the 1985 Dig Tsho GLOF alone destroyed 14 bridges and the nearly completed Namche hydropower station. To mitigate the potential outburst risk of large moraine-dammed lakes, long-term monitoring and engineering interventions have been implemented, successfully preventing lake outburst events to some extent[34,35]. However, small SGLs, owing to their*

rapid evolution and unpredictable seasonal variability, are increasingly becoming a major source of GLOFs; since 2000, five of the seven recorded GLOF events in the region have originated from SGLs[9].

Comment 2.21: Line 99: "Bridges and houses have frequently been damaged." – add citation to studies/examples of this.

Response: Thank you very much for your comment. Based on your suggestion, we have added a representative case study and provided a detailed description of the extent of the damage and its impacts *(For details, please see the revised manuscript: Lines100-103 (This has also been presented in this response))*.

We have revised the paragraph as follows:

Lines 100-103 IN THE REVISED MANUSCRIPT: *Over the past few decades, more than 16 GLOFs and ice avalanches have been reported in the region, resulting in over 340 deaths and missing persons[9,33]. For example, the 1985 Dig Tsho GLOF alone destroyed 14 bridges and the nearly completed Namche hydropower station.*

Comment 2.22: Lines 100-101: "…small glacial lakes have increasingly become the primary source of GLOFs, with dozens of occurrences" – are these small supraglacial lakes or a mix of lake types? Given the focus of the paper, it is important to clarify.

Response: Thank you for pointing out this issue. The "dozens of occurrences" mentioned in the manuscript involve multiple types of glacial lake hazards. Based on your suggestions in other comments, this study should focus on supraglacial lakes; therefore, we have revised the relevant description to emphasize only supraglacial lake outburst events. Specifically, among the seven recorded glacial lake outburst events since 2000, five were associated with supraglacial lakes. Meanwhile, we note that in earlier periods, some large moraine-dammed lakes caused severe disasters; however, with the implementation of artificial monitoring and management measures, the risks of such lakes can now be effectively controlled and early warnings can be issued. In contrast, small supraglacial lakes, due to their rapid evolution and

monitoring difficulties, have gradually become an important source of recent hazards *(For details, please see the revised manuscript: Lines103-107 (This has also been presented in this response))*.

We have revised the paragraph as follows:

> ***Lines 103-107 IN THE REVISED MANUSCRIPT:*** *To mitigate the potential outburst risk of large moraine-dammed lakes, long-term monitoring and engineering interventions have been implemented, successfully preventing lake outburst events to some extent[34,35]. However, small SGLs, owing to their rapid evolution and unpredictable seasonal variability, are increasingly becoming a major source of GLOFs; since 2000, five of the seven recorded GLOF events in the region have originated from SGLs[9]. […]*

Comment 2.23: Lines 101-103: "Such frequent and hard-to-monitor geohazards are rarely included in global compilations of glacial lake outburst events" – can you give an indication here of how frequent? Why are they hard to monitor?

Response: Thank you for your comment. Regarding the term "frequent", we have provided a more detailed explanation. Specifically, among the seven recorded glacial lake outburst events since 2000, five were associated with supraglacial lakes. More precisely, all seven events occurred after 2015, which further highlights the increasing importance of studying supraglacial lake-related hazards. Regarding why these processes are difficult to monitor, we emphasize the strong seasonal variability of supraglacial lakes. We also cite a representative case showing that their short-term and seasonal fluctuations can vary by tens of times. However, due to limitations in satellite revisit intervals, it is difficult to fully capture the complete evolution of supraglacial lakes, from expansion to drainage events *(For details, please refer to the revised manuscript: Lines 106-113 (This has also been presented in this response))*.

We have revised the paragraph as follows:

> ***Lines 106-113 IN THE REVISED MANUSCRIPT:*** *[…] since 2000, five of the seven recorded GLOF events in the region have originated from SGLs[9]. Such frequent and hard-*

to-monitor geohazards are rarely included in global compilations of glacial lake outburst events (Fig. 1). As critical indicators of global climate change, seasonal fluctuations in lake area are considered one of the most sensitive characteristics of small SGLs. Some case studies show that, prior to reaching critical outburst thresholds, some SGLs can expand rapidly over short timescales, with surface area increases exceeding eightfold, and reaching up to 70-fold over a complete seasonal expansion cycle[36]. However, due to the trade-off between spatial and temporal resolution in single-sensor satellite data, such short-term fluctuations are often overlooked[30].

Comment 2.24: Line 103: what are "global lakes"?#

Response: We apologize for the unclear expression that may have caused confusion. The relevant content was derived from reference *(Global dominance of seasonality in shaping lake-surface-extent dynamics. https://doi.org/10.1038/s41586-025-09046-3)* and primarily pertains to global lakes, specifically the statement that "seasonally dominated lakes account for approximately 66% of the total global lake area and 60% of the total number of lakes". However, based on your suggestions in other comments, the introduction of this study should focus on supraglacial lakes. Therefore, to avoid ambiguity, we no longer discuss the seasonal characteristics of global lakes, and instead focus on the seasonal variations of supraglacial lakes. *(For details, please see the revised manuscript: Lines 108-113 (This has also been presented in this response)).*

We have revised the paragraph as follows:

Lines 108-113 IN THE REVISED MANUSCRIPT: *As critical indicators of global climate change, seasonal fluctuations in lake area are considered one of the most sensitive characteristics of small SGLs. Some case studies show that, prior to reaching critical outburst thresholds, some SGLs can expand rapidly over short timescales, with surface area increases exceeding eightfold, and reaching up to 70-fold over a complete seasonal expansion cycle[36]. However, due to the trade-off between spatial and temporal resolution in single-sensor satellite data, such short-term fluctuations are often overlooked[30].*

Comment 2.25: Lines 104-105: "…seasonally dominated lakes comprising approximately 66% of the total global lake area" –there should be a citation here to the source of the reported data.

Response: Thank you very much for pointing out this issue. The reference here is *"Global dominance of seasonality in shaping lake-surface-extent dynamics. https://doi.org/10.1038/s41586-025-09046-3"*, which primarily pertains to all lakes globally. However, based on your suggestion elsewhere that we should introduce the main focus of our study earlier, we have decided to remove this statement. We have instead focused on the core issue—the seasonality of supraglacial lakes—and have revised the text accordingly, adding cases of seasonal supraglacial lake outburst events and clarifying their seasonal characteristics *(For details, please see the revised manuscript: Lines 108-113 (This has also been presented in this response))*.

We have revised the paragraph as follows:

> ***Lines 108-113 IN THE REVISED MANUSCRIPT:*** *As critical indicators of global climate change, seasonal fluctuations in lake area are considered one of the most sensitive characteristics of small SGLs. Some case studies show that, prior to reaching critical outburst thresholds, some SGLs can expand rapidly over short timescales, with surface area increases exceeding eightfold, and reaching up to 70-fold over a complete seasonal expansion cycle[36]. However, due to the trade-off between spatial and temporal resolution in single-sensor satellite data, such short-term fluctuations are often overlooked[30].*

Comment 2.26: Lines 105-108: "Seasonal fluctuations in lake area are considered one of the most sensitive characteristics of small glacial lakes. However, they are often overlooked due to the trade-off between spatial and temporal resolution in single-sensor satellite data30. Therefore, urgent investigations into the seasonal dynamics of small glacial lakes and their implications for mountain hazard management are needed" – references here to "small glacial lakes": are you referring here to supraglacial lakes? This should be stated.

Response: Thank you very much for your comment. As you pointed out, the term here should be supraglacial lakes. Based on your suggestion, we have revised "small glacial lakes" to "small supraglacial lakes" in this section to avoid ambiguity.

Comment 2.27: Line 109: "glacial lake" – should this be "supraglacial lake"?

Response: Thank you very much for pointing out this issue. The correct term here should be "supraglacial lake," and we have made the corresponding revision. At the same time, we have carefully checked the entire manuscript to ensure the accuracy of the scientific terminology.

Comment 2.28: Lines 110-111: "surface deformations" – what is meant by this? It could have several meanings so it would be best to be clear. Is this ice surface or other?

Response: We apologize that our Methods section was not sufficiently clear, which may have led to misunderstandings. Upon review, we confirmed that this is a technical term used in InSAR. Here, the deformation primarily refers to areas surrounding the terminus lakes, rather than the ice surface itself. We have added relevant descriptions of InSAR in the Methods section and revised the original phrasing to help readers better understand the intended meaning *(For details, please refer to the revised manuscript: Lines 553-556, Line 557-576 (This has also been presented in this response))*.

We have revised the paragraph as follows (Comments 2.4 & 2.28 & 2.61–2.64 are similar issues)

> ***Lines 553-556 IN THE REVISED MANUSCRIPT:*** *Therefore, we applied Sentinel-2 imagery to monitor the seasonal dynamics of glacier flow (glacier surface velocity) and used 233 Sentinel-1 synthetic aperture radar (SAR) images to map terminal moraine deformation at the glacier termini.*
>
> ***Lines 557-576:*** *The SBAS-InSAR algorithm is a time-series InSAR approach that generates a set of small-baseline interferograms using a multi-master strategy with appropriately defined spatiotemporal baseline thresholds, enhancing interferometric*

coherence and enabling millimeter- to centimeter-scale monitoring of surface deformation. N+1 single-look complex (SLC) SAR images acquired over the study area at consecutive time points $t0, \cdots, tN$ *are processed by selecting a single master image, to which all other images are co-registered and a unified reference grid is established. Differential interferograms are generated from the SLC images, and topographic phase is removed using an external digital elevation model (e.g., AW3D 30 m). Flat and highly coherent stable points (e.g., bedrock) are used as zero-displacement reference points. Following filtering and phase unwrapping, continuous phase time series are obtained for each coherent target, comprising deformation, residual topography, atmospheric, orbital, and noise components. Orbital errors are corrected via least-squares fitting, residual topography is estimated from the linear relationship between perpendicular baseline and elevation, and atmospheric contributions are separated using temporal high-pass and spatial low-pass filtering. Singular value decomposition (SVD) is then applied to convert the multi-master interferogram set into a single-reference time series*[74]*, with unwrapped phase converted into LOS displacement. In the LOS direction, negative values indicate motion away from the satellite, whereas positive values indicate motion toward it. In this study, these deformation patterns manifest as subsidence and deposition in the glacier terminus zone, respectively. Given that InSAR measurements are acquired along the LOS direction and based on the geometric projection relationship, the vertical displacement can be approximated as* $dvertical = dLOS/COS\theta$, *where* θ *is the incidence angle*[75]*. All processing was performed using GAMMA software.*

Comment 2.29: Line 111: “SST” not explained yet.

Response: Thank you very much for pointing out this issue. In response to your comment, we have provided an explanation of this term in the manuscript.

Comment 2.30: Line 113: what are “anomalous lake events”? – this could have several meanings so please provide clarity.

Response: Thank you very much for raising this question. In response, we have defined "anomalous lake events" as changes in lake area that significantly deviate from their typical seasonal variation patterns *(For details, please refer to the revised manuscript: Lines 120-121 (This has also been presented in this response))*.

We have revised the paragraph as follows:

> ***Lines 120-121 IN THE REVISED MANUSCRIPT:*** *[…] i.e., lake area variations that deviate significantly from their typical seasonal patterns.*

Comment 2.31: Lines 113-114: "Glacier velocity was included in the framework regulating glacial lake expansion." – I don't follow this, more clarity needed.

Response: Thank you for your valuable comment. To clarify the meaning of this statement, we have revised and explained the relevant text in the manuscript *(For details, please refer to the revised manuscript: Lines 121-123 (This has also been presented in this response))*.

We have revised the paragraph as follows:

> ***Lines 121-123 IN THE REVISED MANUSCRIPT:*** *[…] Basnet found that the formation of proglacial lakes accelerates glacier mass loss and promotes glacier retreat, thereby affecting glacier velocity[38]. Therefore, glacier velocity is incorporated into the analytical framework of SGL expansion. […]*

Comment 2.32: Line 116: "small glacier lake" – small supraglacial lakes?

Response: Thank you for pointing out this issue. As you mentioned, this refers to "small supraglacial lakes," and we have made the correction based on your comment.

Results

Comment 2.33: Lines 129-130: "In recent decades, the rapid expansion of supraglacial lakes has attracted considerable attention, though most studies have focused on lakes larger than 0.05

km², largely overlooking small lakes" – this sentence seems like it should be in the introduction, not results.

Response: Thank you very much for your comment. Based on your suggestion, we have removed this statement from the Results section.

Comment 2.34: Lines 130-132: "The high spatiotemporal resolution and frequent revisit of Planet imagery allow analysis of individual lake filling and drainage events." – this should be in the methods.

Response: Thank you for your comment. Based on your suggestion, we have moved this sentence to the methods section.

Comment 2.35: Line 133: "nine glacier groups" – how many individual glaciers? Is it the 19 shown in Fig. 2? "With 42–502 lakes per group" – how many per glacier?

Response: Thank you for your comment. In this study, we define a glacier group as glaciers in the upstream basin that share a common outlet. Due to the branching structure of some glaciers, the 19 panels in Fig. 2 do not represent 19 independent glaciers, but rather different glacier branch units, each of which has been individually numbered. The labels in Fig. 2a correspond to these glacier branch units. To avoid confusion between the numbering system and the glacier branching structure, we have revised the numbering scheme to more clearly track lake dynamics across different glacier branches. **Specifically, each branch unit now follows a numbering system consistent with its glacier group, replacing the original 1–19 scheme. For example, Group 9 contains three branches, labeled as Group 9-1, 9-2, and 9-3, while Group 2, which has no branching structure, remains labeled as Group 2.** To further clarify the correspondence between the numbering system and glacier branches, we have added four figures in the Supplementary information illustrating this mapping. In addition, regarding the number of lakes in each glacier group, this information was originally presented in Fig. 2a-1. Based on your comment that this panel was not sufficiently clear, we have separated it into Fig. 2c to more clearly show the number of lakes in each glacier group. We have also added a table

in the manuscript detailing the number of lakes in each glacier group *(For details, please refer to the revised manuscript: Extended Data Table 1 (This has also been presented in this response))*.

Note: The above provides an explanation. To improve clarity, we have added Extended Data Table 1, which allows readers to quickly understand the classification scheme at a glance.

We have revised the paragraph as follows (This is a similar issue to Comment 2.6 & 2.35 & 2.46 & 2.50):

Extended Data Table 1 | Correspondence between Glacier Systems, Glacier Names, Glacier Branches, and Lake Numbers in Each Glacier Group

Glacier System	Glacier Name	Glacier Branch (Fig.2)	Number of Lakes
Group 1	Ambulapcha	1-1	88
	Imja	1-2	
	Lhotse Shar	1-3	
Group 2	Lhotse	2	114
Group 3	Lhotse Nup	3	42
Group 4	Ama Dablam	4	42
Group 5	Nuptse	5	66
Group 6	Khumbu	6	144
Group 7	Ngozumpa	7-1	391
		7-2	
		7-3	
Group 8	Bhote Koshi	8-2	502
		8-3	
		8-4	
	Lumsamba	8-1	
		8-5	
Group 9	Melung	9-1	104
		9-2	
		9-3	

Glaciers sharing the same outlet were classified into the same group. The nine groups together contain 12 independent glaciers. Due to branching structures in some glaciers, a total of 19 glacier branch units were identified. The spatial correspondence between the identifiers and specific glacier branches is shown in Fig. 2a and Figs.S1–S4.

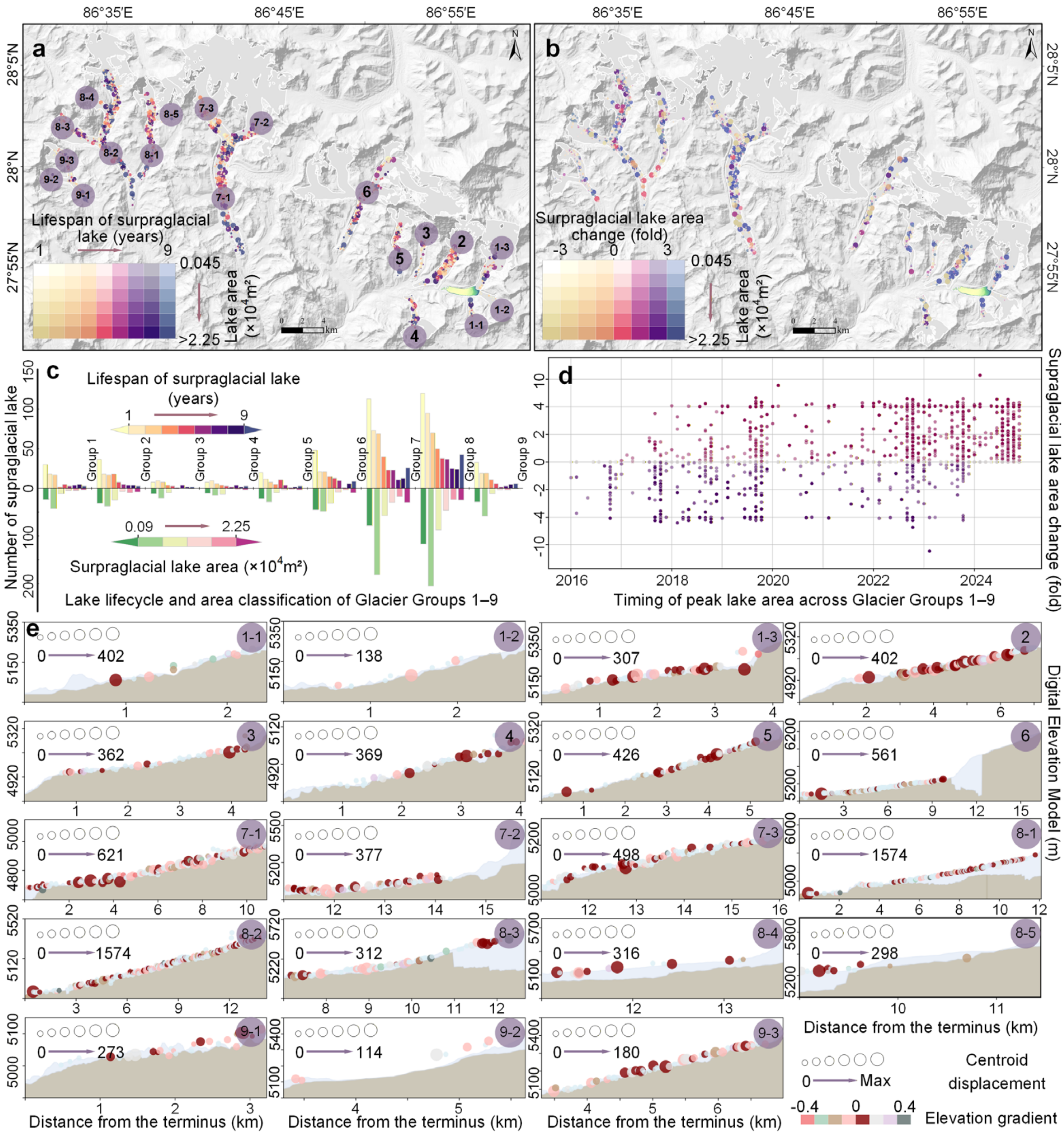

Fig.2 | Spatial distribution, temporal dynamics, and lifecycle characteristics of supraglacial lakes between 2016 and 2024. a Spatial patterns and lifecycles of SGLs of different sizes (2016–2024), due to the branching structure of some glaciers, each branch was assigned a unique identifier to facilitate analysis of the spatiotemporal distribution of lakes (Fig. S1-S4). The correspondence between glacier systems, glacier names, glacier branches, and the

number of lakes in each group is provided in Extended Data Table 1. **b** Spatial patterns of lake peaks and peak-to-trough change rates. In panels **a** and **b**, circle size represents lake area, which is classified into six levels: of 0.045×10^4, 0.09×10^4, 0.36×10^4, 0.81×10^4, 1.14×10^4, 2.25×10^4, and greater than 2.25×10^4 m², respectively. **c** Lifecycle, number, and size distribution characteristics of lakes in each glacier group **d** Timing of peak SGL area for expanding and shrinking lakes across nine glacier groups. **e** Lake centroids' migration distances and elevation gradients, with panel e labels matching glacier numbers in panel a. In panel **e**, circle size indicates lake migration distance, divided into six levels using equal intervals. In the profile plots of this panel e, the grey-blue color represents the elevation profile extracted along the glacier centerline, while the brown color represents the minimum elevation from the corresponding cross-sectional profiles. The green lake in panels a and b is Imja Tsho. To reduce the risk of its outburst, the Nepalese government, together with the United Nations Development Programme (UNDP) and the Nepal Army, constructed a drainage channel from June to September 2016. Over a cumulative period of two months, approximately 3.4 m of water was lowered, discharging about 4–5 million cubic meters.

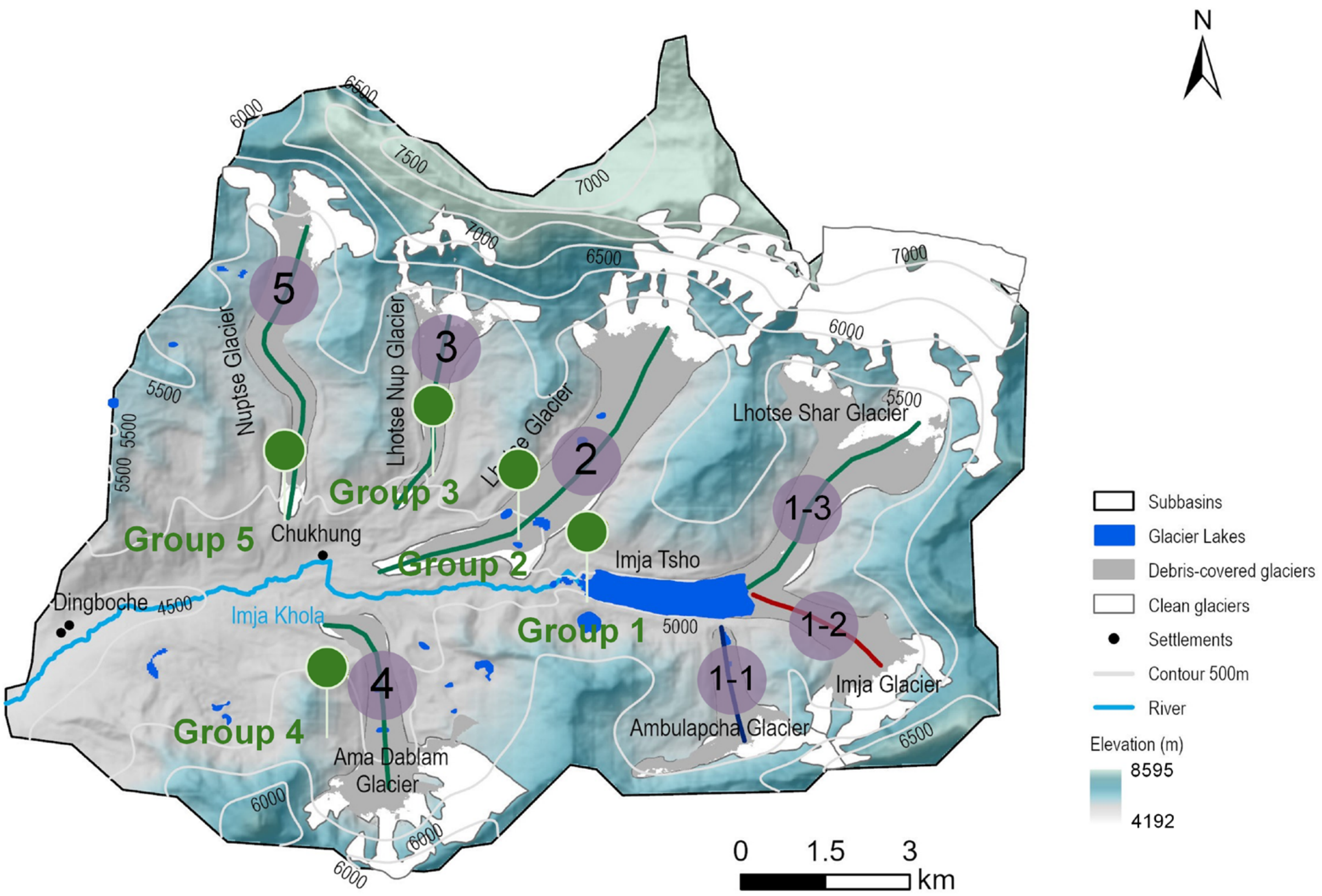

Fig. S1 | Criteria for glacier system classification and spatial distribution of glaciers. Group 1-5 represent the spatial distribution of five glacier systems, and the numbers correspond to the glacier branches in Fig. 2. The relationships are as follows: **Group 1** Lhotse Shar Glacier (circled 1-1), Imja Glacier (circled 1-2), and Ambulapcha Glacier (circled 1-3); **Group** 2 Lhotse Glacier (circled 2); **Group** 3 Lhotse Nup Glacier (circled 3); **Group** 4 Ama Dablam Glacier (circled 4); and **Group** 5 Nuptse Glacier (circled 5).

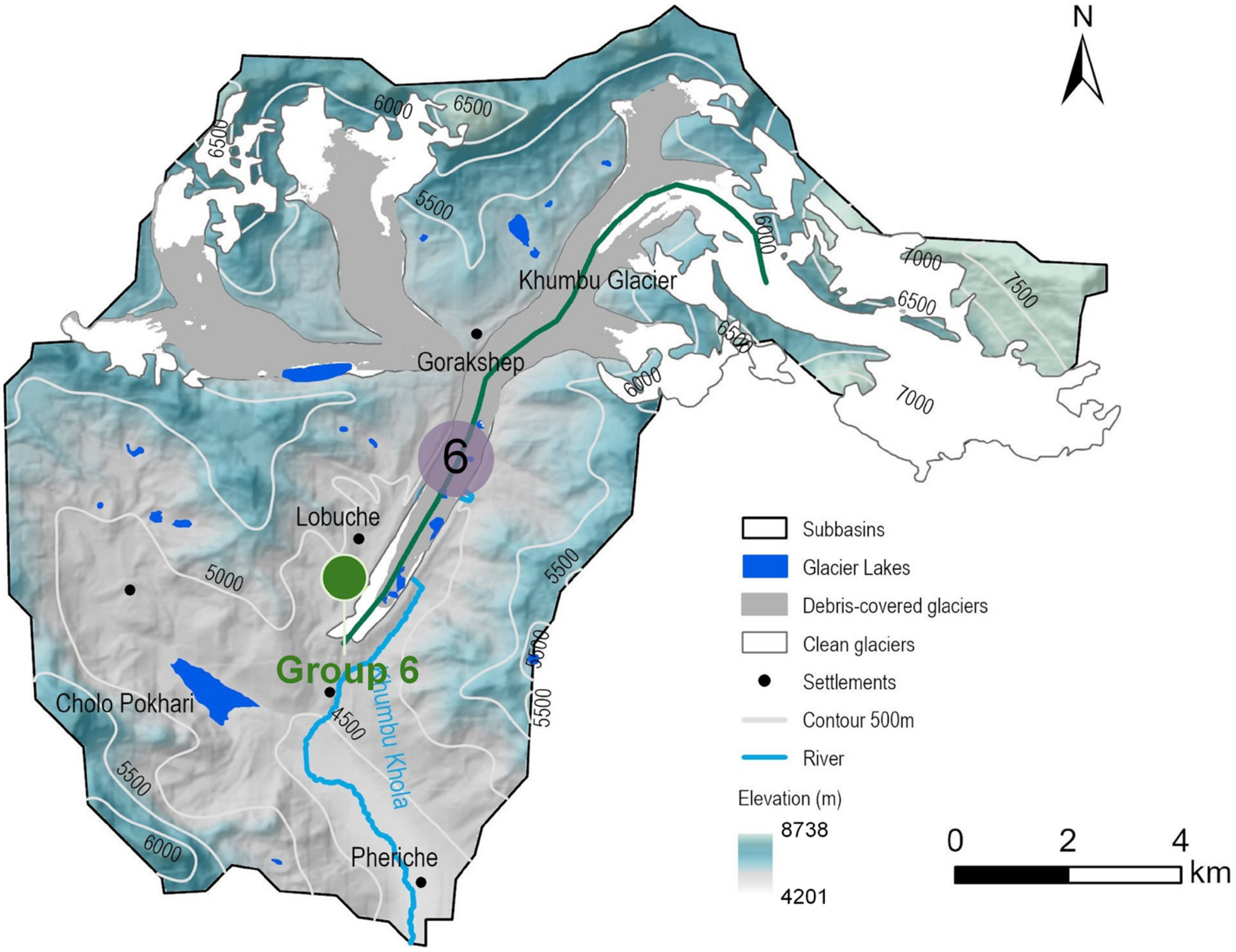

Fig. S2 | Criteria for glacier system classification and spatial distribution of glaciers. Group 6 represents the spatial distribution of the glacier group, and the number corresponds to the glacier branch in Fig. 2. The correspondence is as follows: **Group 6** Khumbu Glacier (circled 6).

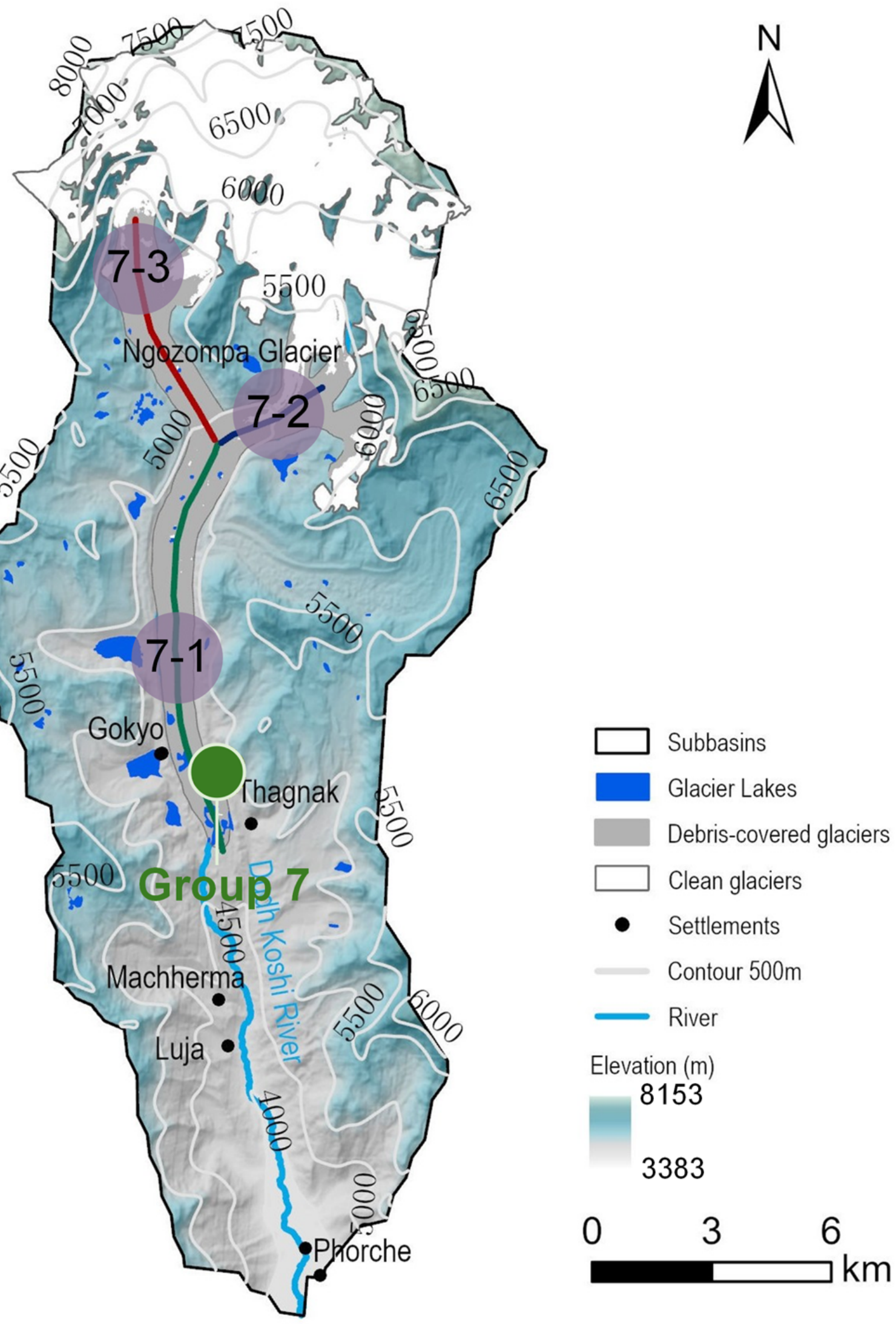

Fig. S3 | Criteria for glacier system classification and spatial distribution of glaciers. Group 7 represents the spatial distribution of the glacier group, and the number corresponds to the glacier branch in Fig. 2. The correspondence is as follows: **Group** 7 Ngozumpa Glacier (circled 7-1, 7-2 and 7-3).

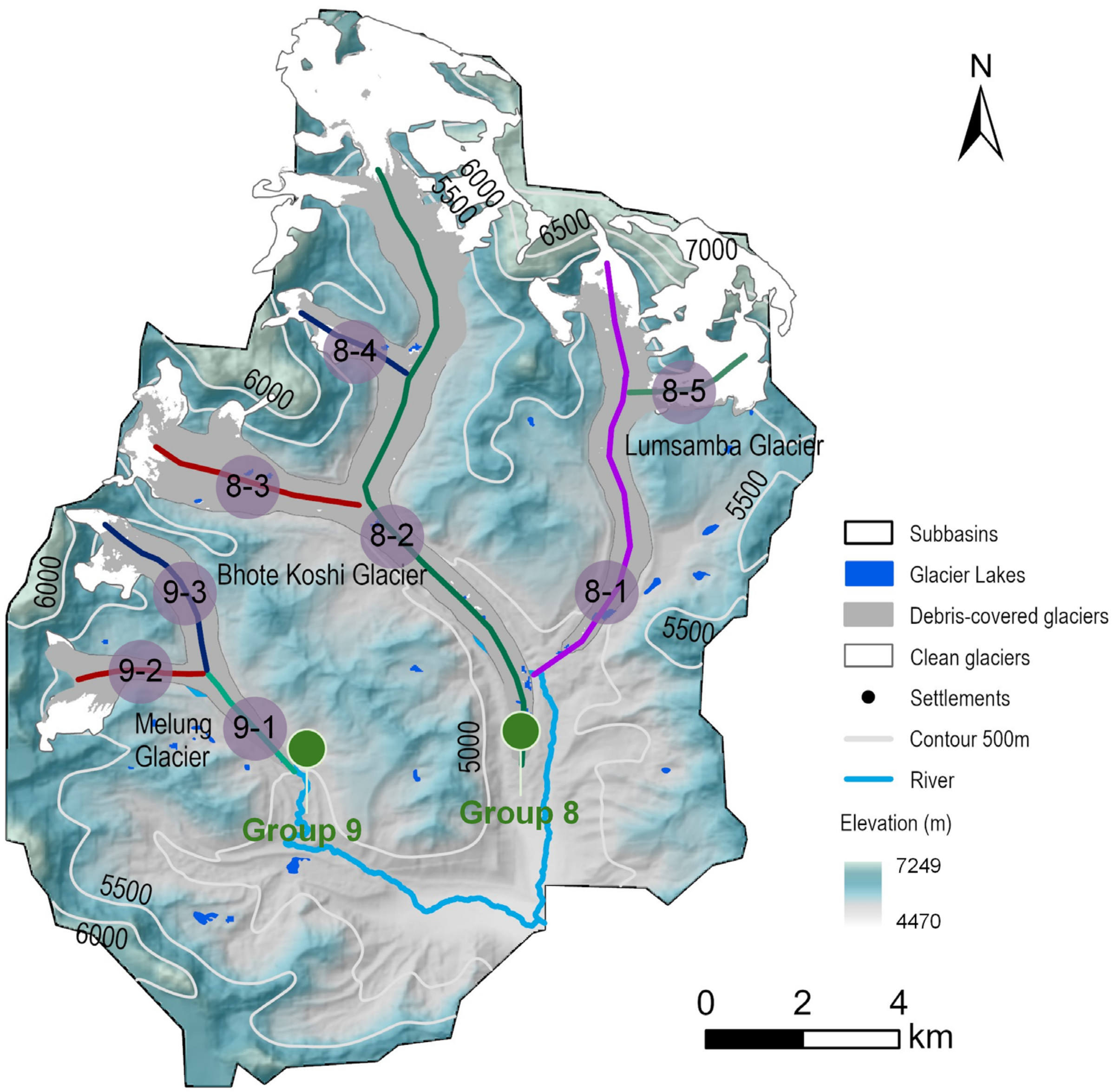

Fig. S4 | Criteria for glacier system classification and spatial distribution of glaciers. Group 8-9 represents the spatial distribution of the glacier group, and the number corresponds to the glacier branch in Fig. 2. The correspondence is as follows: **Group** 8 Bhote Koshi Glacier (circled 8-2, 8-3 and 8-4) and Lumsamba Glacier (circled 8-1 and 8-5). **Group** 9 Melung Glacier (circled 9-1, 9-2 and 9-3)

Comment 2.36: Lines 133-134: "Only 98 lakes were perennial" – suggest adding that this is for the duration of the study period.

Response: Thank you very much for your valuable comment. Following your suggestion, we have added a clarification, emphasizing that it covers the entire study period, from 2016 to 2024.

Comment 2.37: Line 136: "Under global warming." – this isn't required here, delete.

Response: Thank you for pointing out this issue. We have removed it.

Comment 2.38: Lines 132-136: The constant change in unit order of magnitude makes it hard to gauge the differences between lake sizes, e.g. 10^{-4} km², km², 10⁻2 km², 10⁻3 km² on four different lines. Figure 2 then uses a fixed 10^{3} km². Please use the same unit of measurement throughout.

Response: Thank you for your suggestion. Following your advice, we have reviewed the entire manuscript and standardized all units related to lakes to 10^{4} m² throughout, including both figures and the main text.

Comment 2.39: Line 139: what are the "peaks of contracting lakes"? This seems counterintuitive.

Response: We apologize for the unclear expression. What we originally intended to convey is that, at the level of individual lakes, some lakes are expansion-dominated while others are shrinking-dominated during the study period. For expansion-dominated lakes, their peak area is mainly concentrated in the period 2022–2024, whereas for shrinking-dominated lakes, their peak area is mainly concentrated in the period 2017–2019. Based on your comment regarding this lack of clarity, we have revised the original text accordingly. *(For details, please see the revised manuscript: Lines 146-148 (This has also been presented in this response))*.

We have revised the paragraph as follows:

> ***Lines 146-148 IN THE REVISED MANUSCRIPT:*** *At the individual lake level, we observed that peak lake areas of expanding lakes were primarily concentrated during*

2022–2024, whereas those of shrinking lakes were mainly concentrated during 2017–2019 (Fig. 2d).

Comment 2.40: Line 140: What is "displacement" in this context? Horizontal movement of ice? And other what timescale is this displacement measured? I have read the section in the methods on this and I am no clearer.

Response: We sincerely apologize for a serious error in our expression. The original sentence, "We examined the spatial dynamics of these glaciers (Fig. 2e)," should correctly read, "We examined the spatial dynamics of these supraglacial lakes (SGLs)," as the displacement primarily refers to the lakes rather than the ice *(For details, please refer to the revised manuscript: Lines 148-149 (This has also been presented in this response))*. We extracted the annual distribution of each lake from 2016 to 2024 and quantified the displacement of each lake as well as the changes in elevation before and after displacement, thereby quantifying the lake displacement gradient. Additionally, we extracted the elevation of the glacier central line. By comparing the elevation of the glacier central line along the same profile with the lake elevations and displacement, we can reflect the distribution and expansion potential of the lakes. To clearly convey our intention, we have revised the description in the Methods section *(For details, please refer to the revised manuscript: Lines 515-519 (This has also been presented in this response))*.

We have revised the paragraph as follows (This is a similar issue to Comment 2.40 & 2.41):

Line 148-149 IN THE REVISED MANUSCRIPT: *We examined the spatial dynamics of these SGLs (Fig. 2e), [...]*

Lines 515-519: *Centroids of each lake were extracted from annual lake extents from 2016 to 2024, and the maximum migration distance and corresponding elevation change over the study period were quantified to characterize centroid displacement and migration gradients. For each lake, transects perpendicular to the glacier centerline were*

constructed, and the spatial distribution of lake displacement gradients was mapped (Fig. 2).

Comment 2.41: Lines 141-142: "At the terminus of No. 9 Glacier, the cumulative displacement of terminus lakes reached 1,574 m, resulting from the merger of two terminus lakes, while the maximum displacement of lakes at other glaciers ranged between 114 and 621 m." – I don't understand what this is measuring, this needs to be clearly explained.

Response: We apologize for the unclear expression at the beginning of this paragraph, which may have caused confusion. We have revised the opening statement to emphasize that it refers to the displacement of the lake rather than the glacier. Additionally, we have clarified this in the Methods section to ensure that readers can fully understand our description and the focus of the study *(For details, please refer to the revised manuscript: Line148-149, Lines 515-519 (This has also been presented in this response)).*

We have revised the paragraph as follows (This is a similar issue to Comment 2.40 & 2.41):

Line 148-149 IN THE REVISED MANUSCRIPT: *We examined the spatial dynamics of these SGLs (Fig. 2e), [...]*

Lines 515-519: *Centroids of each lake were extracted from annual lake extents from 2016 to 2024, and the maximum migration distance and corresponding elevation change over the study period were quantified to characterize centroid displacement and migration gradients. For each lake, transects perpendicular to the glacier centerline were constructed, and the spatial distribution of lake displacement gradients was mapped (Fig. 2).*

Comment 2.42: Lines 144-145: "…Imja Lake primarily expanded within ~2 km of the terminus, where surface slopes are as low as 2°" – can you clarify if this is a supraglacial lake? Within ~2 km of the terminus – is this referring to upglacier of the terminus?

Response: Thank you very much for your comment. This refers to a proglacial lake that has developed from supraglacial lakes. We apologize for the lack of clarity in our Introduction, which may have caused confusion. We have revised the Introduction to emphasize that supraglacial lakes can potentially evolve into proglacial lakes.

Regarding "Within ~2 km of the terminus – is this referring to upglacier of the terminus?", in the early stage of lake development, this location was indeed situated in the up-glacier direction of the glacier terminus. As the lake continued to expand, it gradually evolved into a proglacial lake. Its expansion has mainly progressed in the up-glacier direction, and the current length has reached approximately 3 km. To improve clarity, we have revised this section and added relevant references. *(For details, please see the revised manuscript: Lines 153-156 and "Evaluating the growth characteristics of a glacial lake and its degree of danger of outburst flooding: Imja Glacier, Khumbu Himal, Nepal" https://doi.org/10.1080/00291950903368367).*

We have revised the paragraph as follows:

> ***Lines 153-156 IN THE REVISED MANUSCRIPT:*** *[…] For instance, Imja Tsho, a large proglacial lake that developed from five supraglacial ponds in the 1950s[35], primarily expanded within ~2 km upglacier of the glacier terminus. Since then, the lake has continued to expand upglacier, currently reaching a length of approximately ~3 km.*

Comment 2.43: Line 146: is "Imja Tsho" the same as "Imja Lake"?

Thank you for your comment. Yes, Imja Tsho and Imja Lake are often used interchangeably, as "Tsho" means "lake" in Tibetan/Nepali. For example, Dig Tsho and Mabu Tsho. We apologize for not maintaining consistent naming throughout the manuscript. Since the manuscript also involves naming other lakes, we have chosen to use Imja Tsho to ensure consistency.

Comment 2.44: Lines 146-147: "…although Lhotse and No. 9 glaciers have substantial water supply, they lack riverbeds suitable for forming large lakes." – The use of 'riverbed' here

suggests this is not discussing supraglacial lakes, which is confusing given that is the focus of the paper. Clarification needed.

Response: Thank you for your comment. As you mentioned, the focus of our study is on supraglacial lakes. However, with global warming and glacier retreat, some supraglacial lakes gradually develop into proglacial lakes. This point was not clearly expressed in the Introduction. Therefore, we have revised the Introduction to emphasize the transformation between the two types of lakes and the characteristics of supraglacial and proglacial lake development, in order to highlight their connection *(For details, please refer to the revised manuscript: Lines 75-77, Lines 82-84, Lines 153-154(This has also been presented in this response))*.

We have revised the paragraph as follows:

> ***Lines 75-77 IN THE REVISED MANUSCRIPT:*** *[…] which further enhances ice ablation and subglacial melting, causing additional glacier mass loss and retreat, and thereby driving further lake expansion, and merging of small supraglacial lakes (SGLs) into larger proglacial lakes*
>
> ***Lines 82-84:*** *[…] Concentrated discharge is particularly likely where multiple small SGLs are hydraulically connected through supraglacial drainage networks and collectively feed into a larger proglacial lake, increasing the likelihood of catastrophic GLOFs[18, 27].*
>
> ***Lines 153-154:*** *For instance, Imja Tsho, a large proglacial lake that developed from five supraglacial ponds in the 1950s[39], […]*

Comment 2.45: Lines 147-152: I don't understand where the inferences of "lack stable water sources" and "potential water supply" come from. In general, I am finding it difficult to follow the main points that these sentences are trying to make.

Response: Thank you for pointing out this issue. What we intended to convey is that the number and area of supraglacial lakes in Group 3 and Group 4 are relatively small compared with those in other glacier groups (Fig. 3c, 3d), making it difficult for them to accumulate into large

proglacial lakes. Upon careful review of the literature, we realized that our original wording was not entirely accurate, as even glaciers with few supraglacial lakes may still have substantial meltwater; it is just less likely to concentrate on the glacier surface or at the terminus to form lakes. Therefore, we have revised our wording to indicate that meltwater from these glaciers is difficult to concentrate *(For details, please refer to the revised manuscript: Lines 158-166 (This has also been presented in this response))*.

We have revised the paragraph as follows:

> ***Lines 158-166 IN THE REVISED MANUSCRIPT:*** *[…] We further assessed whether other glacier–lake systems follow a similar evolutionary trajectory to Imja Tsho and found that Glacier Groups 2, 5 and 7–9 have progressed beyond the early stage of proglacial lake formation, characterized by a transition from short-lived, spatially dispersed SGLs to a persistent and relatively stable, continuous lake at the glacier terminus (Fig. 4, S5 and S8-S10). In contrast, Glacier Groups 3 and 4 show no clear tendency toward proglacial lake formation, as their termini lack the capacity to accumulate meltwater, and water bodies persist only as short-lived SGLs (Fig. S6-S7). By comparison, Glacier Group 6 represents a distinct hydrological end-member, characterized by widespread SGLs across the glacier surface but lacking any evidence of convergence or accumulation at the glacier terminus (Extended Data Fig. 2). […]*

Comment 2.46: Line 163: "…nine groups of supraglacial lakes" – I do not understand the context of these nine groups as this has not been clearly defined before this. There are 19 labelled glaciers in Figure 2, but how these are subdivided into nine groups is not clear and needs to be explained. No need to mention the study area again here.

Response: Thank you for your comment. In this study, we define a glacier group as glaciers in the upstream basin that share a common outlet. Due to the branching structure of some glaciers, the 19 panels in Fig. 2 do not represent 19 independent glaciers, but rather different glacier branch units, each of which has been individually numbered. The labels in Fig. 2a correspond to these glacier branch units. To avoid confusion between the numbering system and the glacier

branching structure, we have revised the numbering scheme to more clearly track lake dynamics across different glacier branches. **Specifically, each branch unit now follows a numbering system consistent with its glacier group, replacing the original 1–19 scheme. For example, Group 9 contains three branches, labeled as Group 9-1, 9-2, and 9-3, while Group 2, which has no branching structure, remains labeled as Group 2.** To further clarify the correspondence between the numbering system and glacier branches, we have added four figures in the Supplementary Information to illustrate this mapping.

Note: The above provides an explanation. To improve clarity, we have added Extended Data Table 1, which allows readers to quickly understand the classification scheme at a glance.

We have revised the paragraph as follows (This is a similar issue to Comment 2.6 & 2.35 & 2.46 & 2.50):

Extended Data Table 1 | Correspondence between Glacier Systems, Glacier Names, Glacier Branches, and Lake Numbers in Each Glacier Group

Glacier System	Glacier Name	Glacier Branch (Fig.2)	Number of Lakes
Group 1	Ambulapcha	1-1	88
	Imja	1-2	
	Lhotse Shar	1-3	
Group 2	Lhotse	2	114
Group 3	Lhotse Nup	3	42
Group 4	Ama Dablam	4	42
Group 5	Nuptse	5	66
Group 6	Khumbu	6	144
Group 7	Ngozumpa	7-1	391
		7-2	
		7-3	
Group 8	Bhote Koshi	8-2	502
		8-3	
		8-4	
	Lumsamba	8-1	
		8-5	
Group 9	Melung	9-1	104
		9-2	
		9-3	

Glaciers sharing the same outlet were classified into the same group. The nine groups together contain 12 independent glaciers. Due to branching structures in some glaciers, a total of 19 glacier branch units were identified. The spatial correspondence between the identifiers and specific glacier branches is shown in Fig. 2a and Figs.S1–S4.

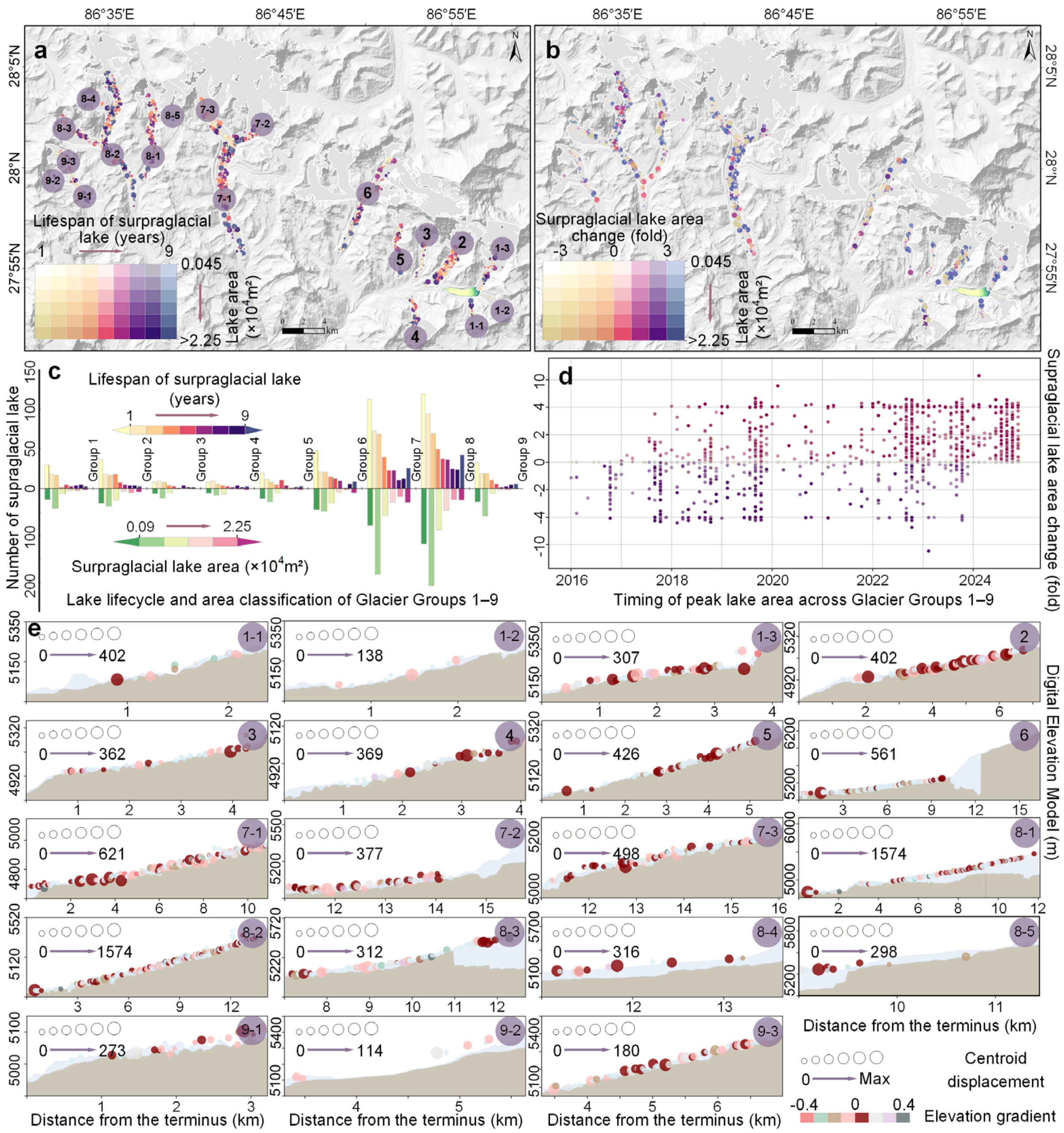

Fig.2 | Spatial distribution, temporal dynamics, and lifecycle characteristics of supraglacial lakes between 2016 and 2024. a Spatial patterns and lifecycles of SGLs of different sizes (2016–2024), due to the branching structure of some glaciers, each branch was assigned a unique identifier to facilitate analysis of the spatiotemporal distribution of lakes (Fig. S1-S4). The correspondence between glacier systems, glacier names, glacier branches, and the number of lakes in each group is provided in Extended Data Table 1. **b** Spatial patterns of lake peaks and peak-to-trough change rates. In panels **a** and **b**, circle size represents lake area, which is classified into six levels: of 0.045×10^4, 0.09×10^4, 0.36×10^4, 0.81×10^4, 1.14×10^4, 2.25×10^4,

and greater than 2.25×10^4 m², respectively. **c** Lifecycle, number, and size distribution characteristics of lakes in each glacier group **d** Timing of peak SGL area for expanding and shrinking lakes across nine glacier groups. **e** Lake centroids' migration distances and elevation gradients, with panel e labels matching glacier numbers in panel a. In panel **e**, circle size indicates lake migration distance, divided into six levels using equal intervals. In the profile plots of this panel e, the grey-blue color represents the elevation profile extracted along the glacier centerline, while the brown color represents the minimum elevation from the corresponding cross-sectional profiles. The green lake in panels a and b is Imja Tsho. To reduce the risk of its outburst, the Nepalese government, together with the United Nations Development Programme (UNDP) and the Nepal Army, constructed a drainage channel from June to September 2016. Over a cumulative period of two months, approximately 3.4 m of water was lowered, discharging about 4–5 million cubic meters.

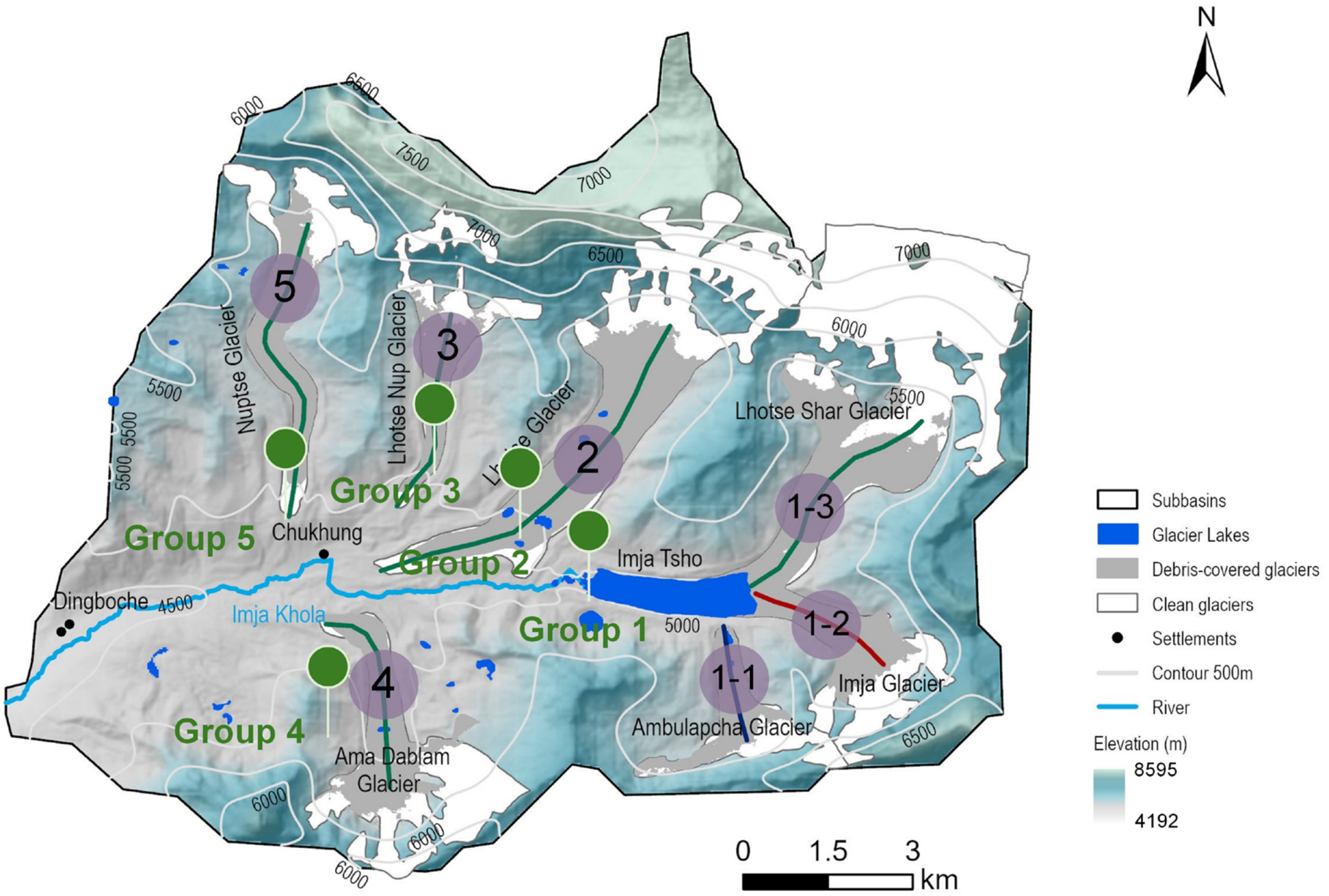

Fig. S1 | Criteria for glacier system classification and spatial distribution of glaciers. Group 1-5 represent the spatial distribution of five glacier systems, and the numbers correspond to the glacier branches in Fig. 2. The relationships are as follows: **Group 1** Lhotse Shar Glacier (circled 1-1), Imja Glacier (circled 1-2), and Ambulapcha Glacier (circled 1-3); **Group** 2

Lhotse Glacier (circled 2); **Group** 3 Lhotse Nup Glacier (circled 3); **Group** 4 Ama Dablam Glacier (circled 4); and **Group** 5 Nuptse Glacier (circled 5).

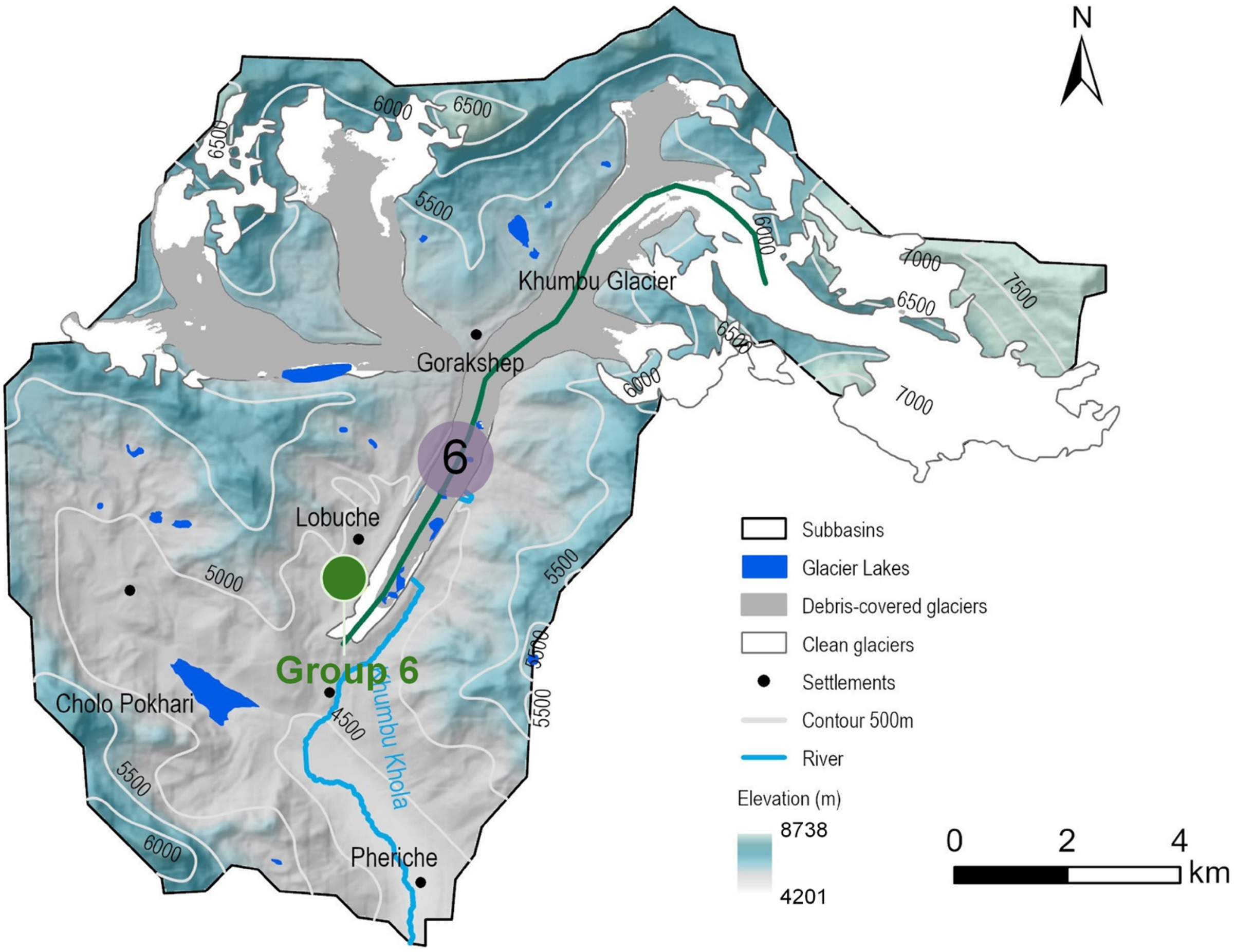

Fig. S2 | Criteria for glacier system classification and spatial distribution of glaciers. Group 6 represents the spatial distribution of the glacier group, and the number corresponds to the glacier branch in Fig. 2. The correspondence is as follows: **Group 6** Khumbu Glacier (circled 6).

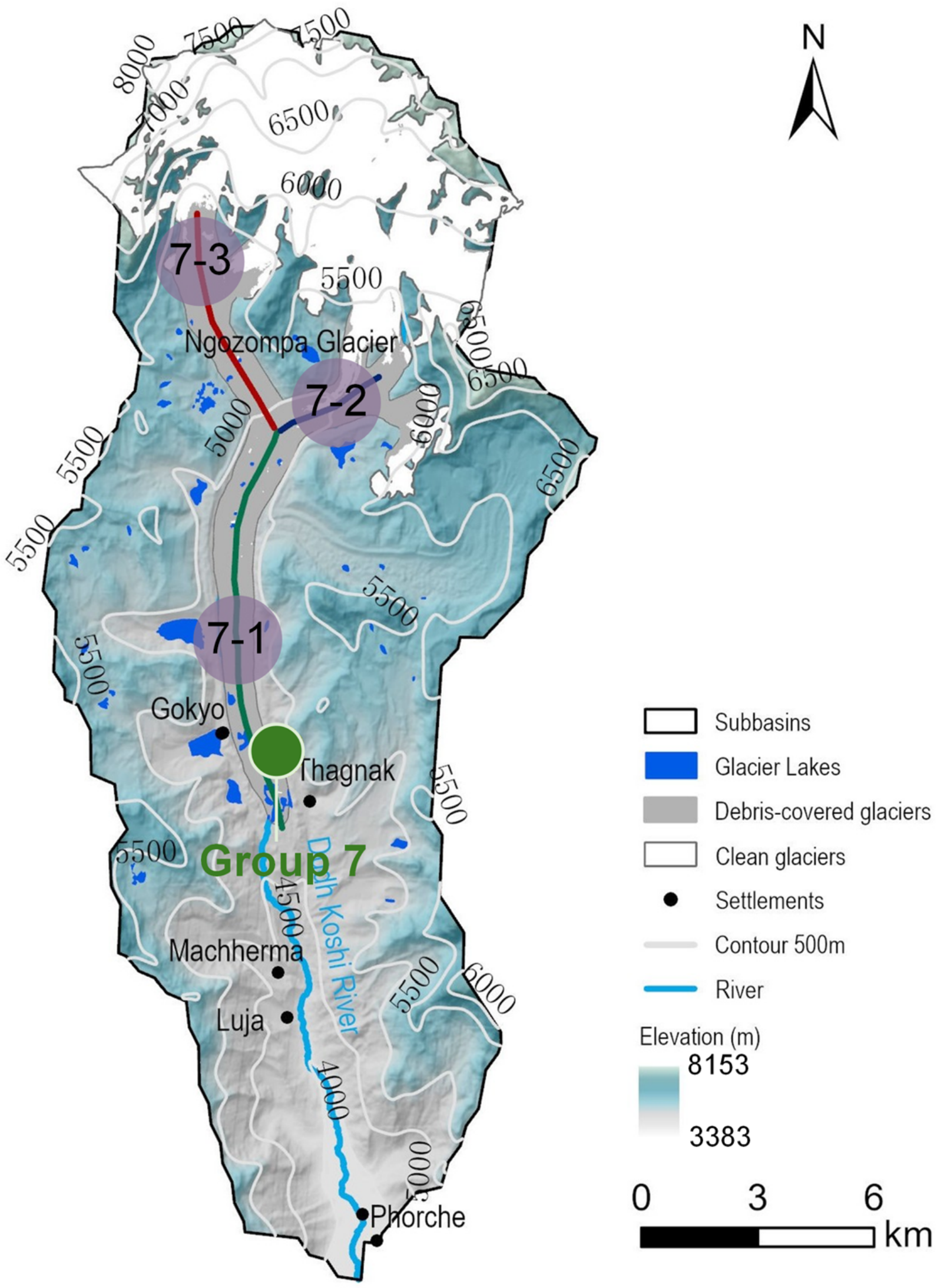

Fig. S3 | Criteria for glacier system classification and spatial distribution of glaciers. Group 7 represents the spatial distribution of the glacier group, and the number corresponds to the glacier branch in Fig. 2. The correspondence is as follows: **Group** 7 Ngozumpa Glacier (circled 7-1, 7-2 and 7-3).

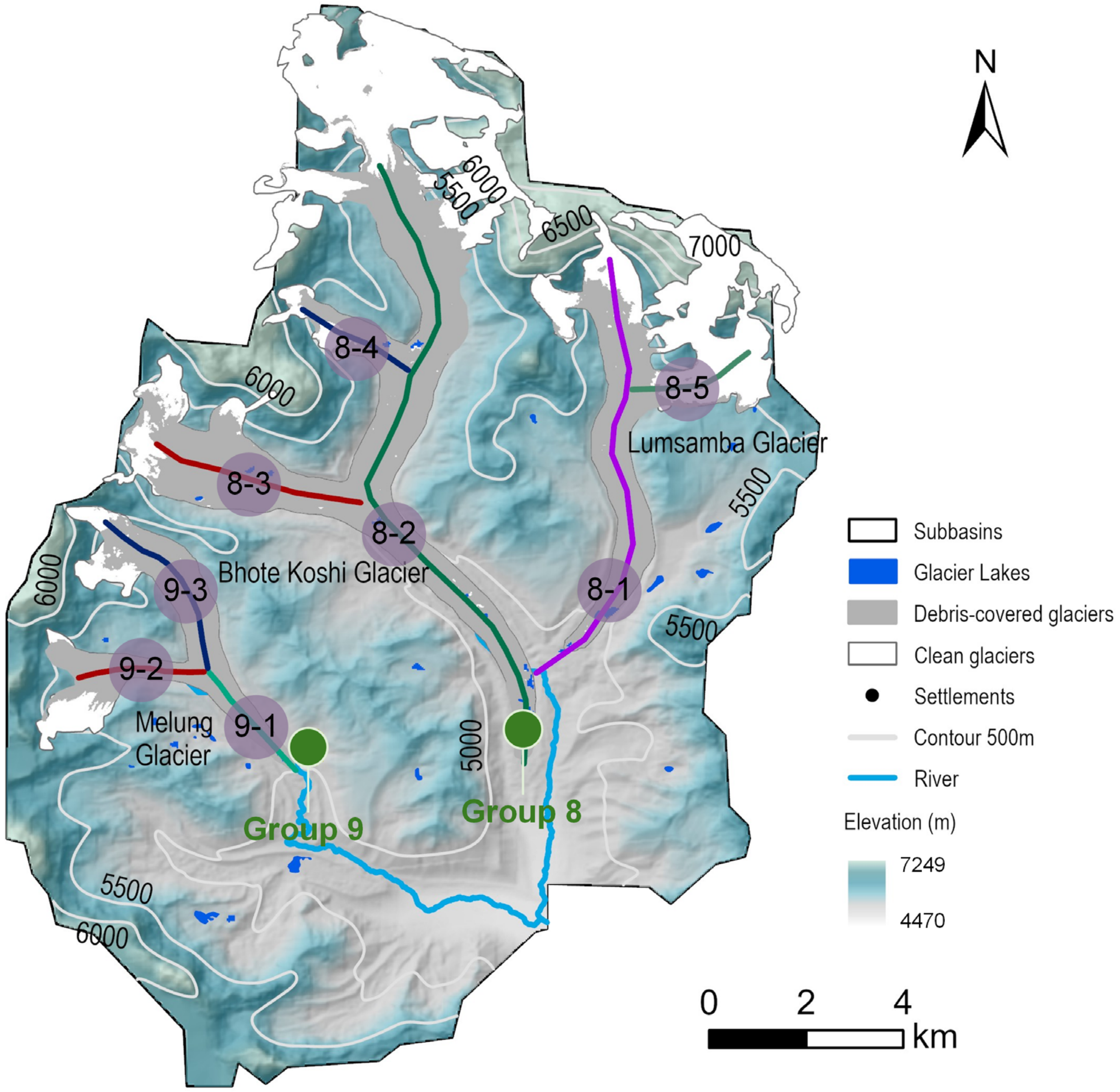

Fig. S4 | Criteria for glacier system classification and spatial distribution of glaciers. Group 8-9 represents the spatial distribution of the glacier group, and the number corresponds to the glacier branch in Fig. 2. The correspondence is as follows: **Group** 8 Bhote Koshi Glacier (circled 8-2, 8-3 and 8-4) and Lumsamba Glacier (circled 8-1 and 8-5). **Group** 9 Melung Glacier (circled 9-1, 9-2 and 9-3)

Comment 2.47: Line 164-165: "Snow and ice ablation together with precipitation expanded the extent of surface water, while the continuous increase in meltwater promoted the seasonal acceleration of ice flow." – how are you able to distinguish these different components from your dataset? This needs to be clearly explained.

Response: Thank you very much for your comment. Our study does not distinguish between snow and ice melt. According to previous studies, the water resources in this region are mainly derived from snow and ice melt as well as precipitation. The part regarding the sustained increase in meltwater, which promotes seasonal acceleration of ice flow, is mainly discussed in the Discussion section. In our study, we primarily use this information to summarize and introduce our results. Based on your comments, we have added the relevant references and made partial revisions to this section to ensure rigor and accuracy. Meanwhile, we carefully reviewed this part of the manuscript and found that it is more appropriate to be included in the section "Inter-seasonal change of ice velocity". Therefore, we have moved it accordingly.

Comment 2.48: Lines 166-175: Similar to a previous comment, the constant change in unit order of magnitude makes it hard to gauge the differences between reported lake sizes. Use the same units throughout the paper. Here, you also name a large number of glaciers – these should be clearly identified on your figures in some way.

Response: Thank you for your comment. Based on your suggestion, we have standardized the units throughout the manuscript to ensure consistency between the figures and the main text. In addition, we provide detailed information on the names and spatial locations of these glaciers in Extended Data Fig. 1 and Supplementary Figs. S1–S4. The corresponding glacier names for each glacier group are also indicated in the captions of the relevant figures. **As noted in our response to Comment 2.6, 2.35, and 2.46,** this study involves a large number of glaciers and their branches. Frequent use of specific glacier names in the main text would increase complexity and reduce readability. Therefore, in the manuscript, we consistently use glacier group identifiers for analysis **(e.g., "the total area of Glacier Group 1 expanded…" rather than "the total area of Lhotse Shar, Imja and Ambulapcha Glaciers expanded …")**. Similarly, in the figures, we adopt a unified labeling system based on the nine glacier groups (Group 1–9), while specific glacier names are provided only in the figure captions. This approach allows readers to refer to the figures and captions for detailed correspondence when needed, thereby improving clarity and overall readability.

Comment 2.49: Line 177: clarify if you mean that the number of lakes expanded, or individual lakes expanded.

Response: Thank you for your comment. Based on your suggestion, we have revised the wording to emphasize the trend in the total area of all lakes *(For details, please see the revised manuscript: Lines 199-201)*.

We have revised the paragraph as follows:

> ***Lines 199-201 IN THE REVISED MANUSCRIPT:*** *During the study period, the annual rates of increase and decrease of the total area of SGLs on the nine glacier groups exhibited heterogeneity.*

Comment 2.50: Lines 177-181: "while the remaining six glaciers showed decreasing trends" – with the named three glaciers in this sentence, this suggests there are nine glaciers. The following sentence also refer to "nine glaciers" – but elsewhere you refer to "nine glacier groups" and Figure 2 suggest there are up to 19 glaciers. Clarification needed here and elsewhere – how many glaciers form part of the study area?

Response: Thank you for your comment. **The study area includes 12 glaciers. Some of these glaciers have branched structures, resulting in a total of 19 glacier branches across the 12 glaciers.** To avoid confusion in terminology, we have revised the classification rule. When multiple upstream glaciers share a single drainage outlet, we treat them as one glacier system, labeled as Group 1, Group 2, …, Group 9.

Most glacier groups contain only a single glacier, such as Group 2 (Lhotse), Group 3 (Lhotse Nup), Group 4 (Ama Dablam), Group 5 (Nuptse), Group 6 (Khumbu), Group 7 (Ngozumpa), and Group 9 (Melung). In contrast, some glacier groups include two or more glaciers, such as Group 1 (Lhotse Shar, Imja, and Ambulapcha) and Group 8 (Bhote Koshi and Lumsamba) *(see revised manuscript Fig. 1 and Extended Data Fig. 1 for details)*.

Regarding the glacier numbering in Figure 2, many glaciers exhibit branched structures. We therefore redefined the numbering scheme to track lake dynamics across different glacier branches. **Each branch unit is now renumbered consistently with its glacier group, replacing the original 1–19 numbering system. For example, Group 9 contains three branches labeled as Group 9-1, 9-2, and 9-3, whereas Group 2 has no branching structure and thus remains labeled as Group 2.** To clarify the correspondence between numbering and glacier branches, we have added four supplementary figures showing the mapping between branch identifiers and glacier systems.

We also carefully considered your comment regarding clarity of presentation. Given that this study involves a relatively large number of glaciers and their branches, frequent use of individual glacier names in the main text would increase complexity and reduce readability. **Therefore, in the manuscript, we consistently use glacier group identifiers for analysis (e.g., "the total area of Glacier Group 1 expanded …" rather than "the total area of Lhotse Shar, Imja and Ambulapcha Glaciers expanded …").** Similarly, in the figures, we use a unified labeling system based on the nine glacier groups (Group 1–9), while specific glacier names are provided only in the figure captions. This approach allows readers to refer to the figures and captions for detailed correspondence when needed, thereby improving clarity and overall readability.

Note: The above provides an explanation. To improve clarity, we have added Extended Data Table 1, which allows readers to quickly understand the classification scheme at a glance.

We have revised the paragraph as follows (This is a similar issue to Comment 2.6 & 2.35 & 2.46 & 2.50):

Extended Data Table 1 | Correspondence between Glacier Systems, Glacier Names, Glacier Branches, and Lake Numbers in Each Glacier Group

Glacier System	Glacier Name	Glacier Branch (Fig.2)	Number of Lakes
Group 1	Ambulapcha	1-1	88
	Imja	1-2	
	Lhotse Shar	1-3	
Group 2	Lhotse	2	114
Group 3	Lhotse Nup	3	42
Group 4	Ama Dablam	4	42
Group 5	Nuptse	5	66
Group 6	Khumbu	6	144
Group 7	Ngozumpa	7-1	391
		7-2	
		7-3	
Group 8	Bhote Koshi	8-2	502
		8-3	
		8-4	
	Lumsamba	8-1	
		8-5	
Group 9	Melung	9-1	104
		9-2	
		9-3	

Glaciers sharing the same outlet were classified into the same group. The nine groups together contain 12 independent glaciers. Due to branching structures in some glaciers, a total of 19 glacier branch units were identified. The spatial correspondence between the identifiers and specific glacier branches is shown in Fig. 2a and Figs.S1–S4.

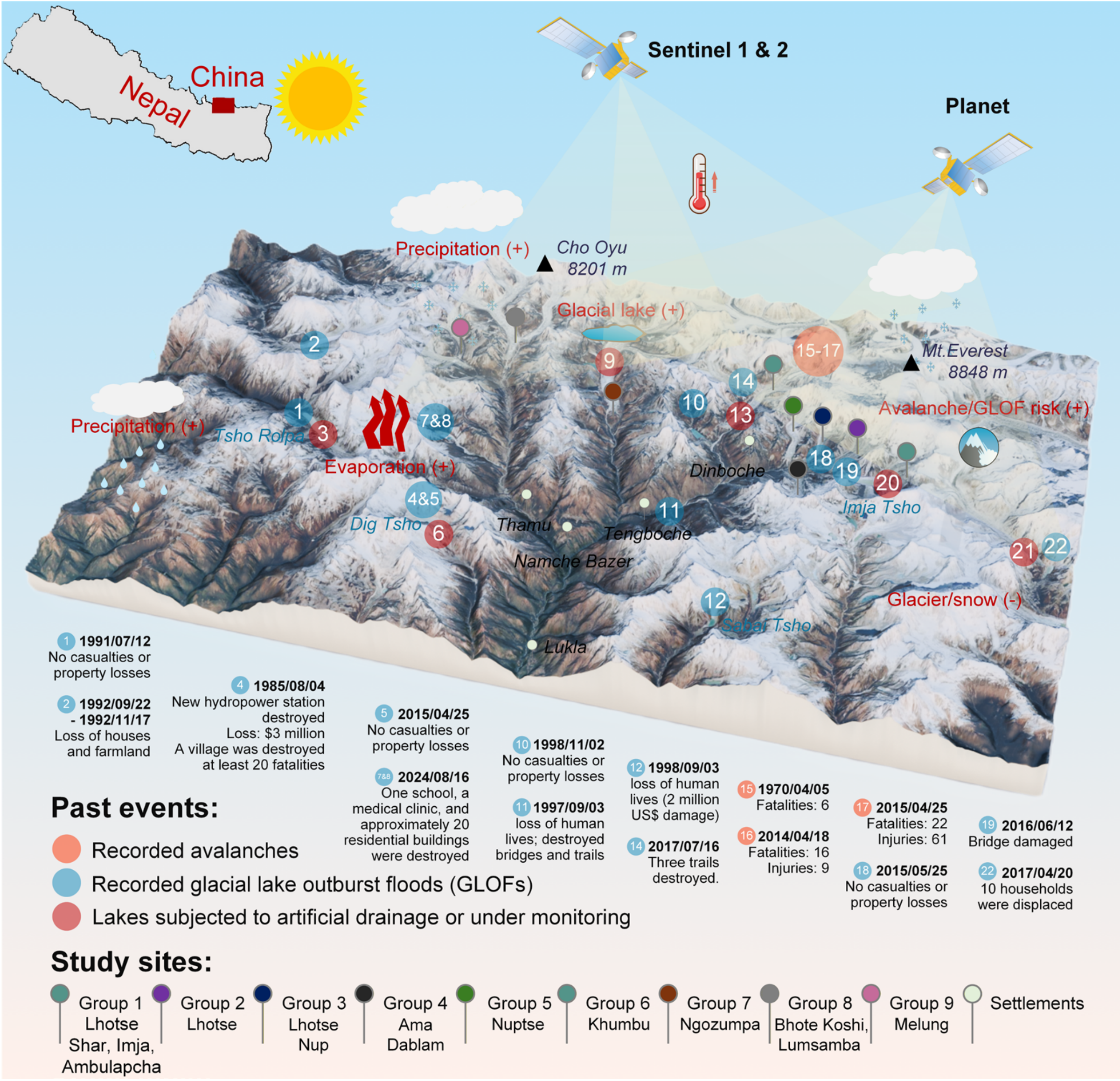

Fig. 1 | Diagram illustrating lake evolution and geological hazards in Sagarmatha National Park. The red labels and text represent the climate-related risks facing this region, namely that under global warming, increased snowfall/rainfall and the melting of glaciers and snowpack lead to the expansion of lake boundaries and heightened risks of GLOFs and avalanches. The numbers in the figure indicate the locations and associated hazard losses of past GLOFs, avalanches, and manually monitored lakes (Data source: Table S1). Glaciers sharing the same drainage outlet were grouped together and labeled as Groups 1–9. The glaciers included in each group are listed as follows: **Group 1** Lhotse Shar, Imja, and Ambulapcha; **Group 2** Lhotse; **Group 3** Lhotse Nup; **Group 4** Ama Dablam; **Group 5** Nuptse; **Group 6** Khumbu; **Group** 7 Ngozumpa; **Group 8** Bhote Koshi and Lumsamba; **Group 9** Melung Glaciers. Extended Data Fig. 1 serves as a supplement to this figure, documenting the spatial

information of clean glaciers, debris-covered glaciers, and runoff.

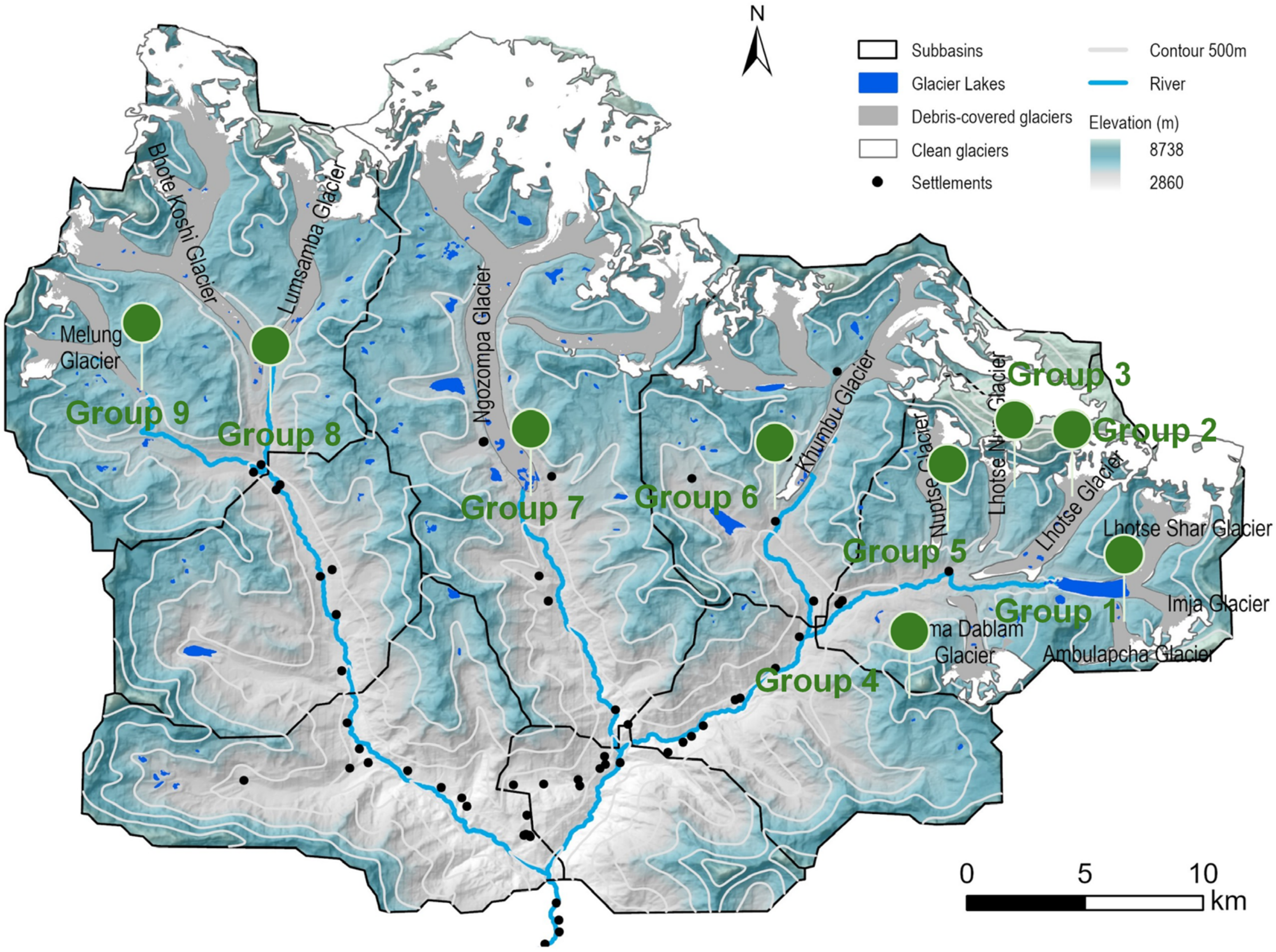

Extended Data Fig. 1 | Spatial distribution and classification of glaciers. Glaciers sharing the same drainage outlet were grouped together and labeled as Glacier Group 1–9. Except for Glacier Groups 1 and 8, which contain two and three individual glaciers, respectively, each of the remaining groups consists of a single glacier. The details are as follows: **Group 1** Lhotse Shar、Imja and Ambulapcha; **Group 2** Lhotse; **Group 3** Lhotse Nup; **Group 4** Ama Dablam; **Group 5** Nuptse; **Group 6** Khumbu; **Group 7** Ngozumpa; **Group 8** Bhote Koshi and Lumsamba and **Group 9** Melung Glaciers**.**

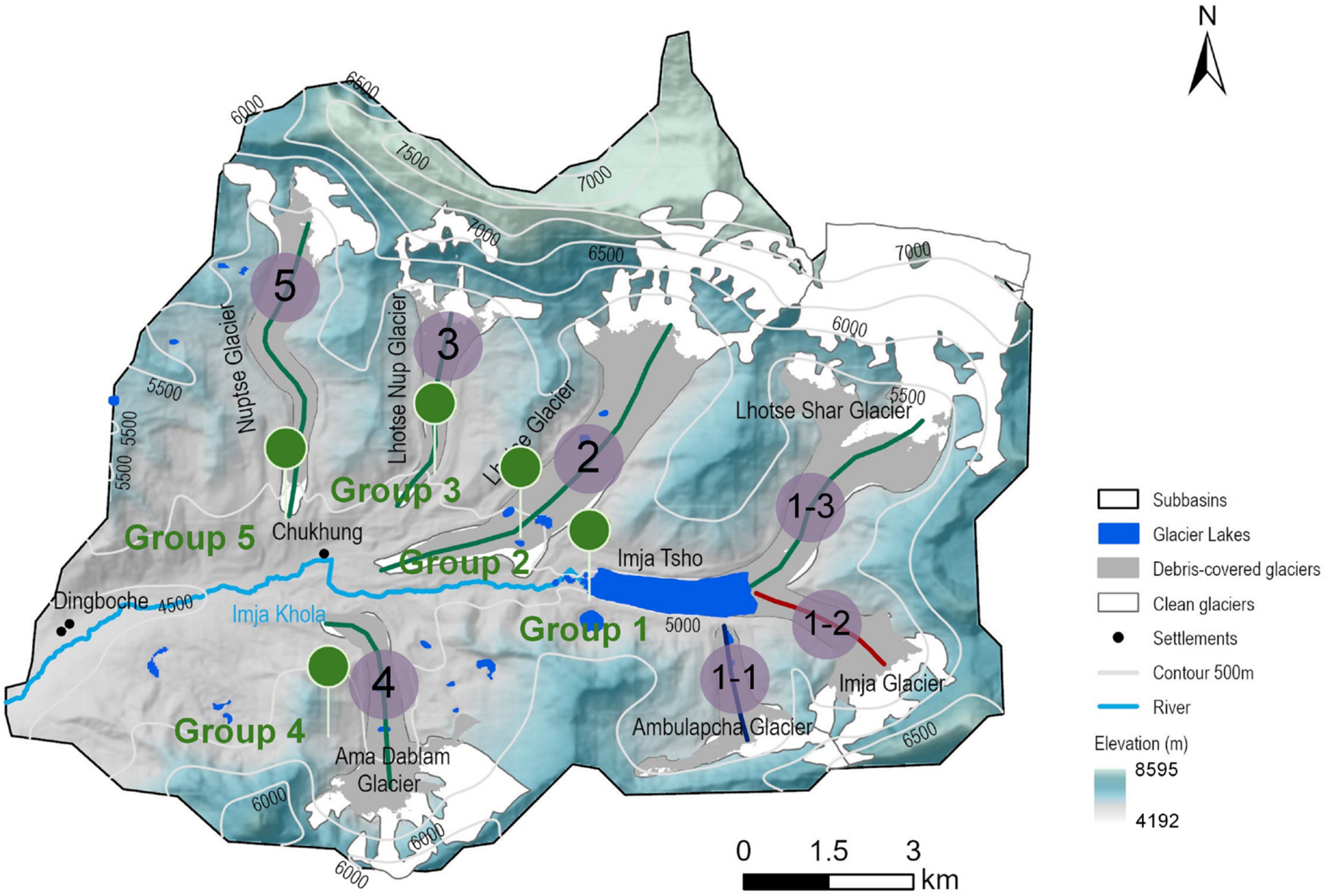

Fig. S1 | Criteria for glacier system classification and spatial distribution of glaciers. Group 1-5 represent the spatial distribution of five glacier systems, and the numbers correspond to the glacier branches in Fig. 2. The relationships are as follows: **Group 1** Lhotse Shar Glacier (circled 1-1), Imja Glacier (circled 1-2), and Ambulapcha Glacier (circled 1-3); **Group** 2 Lhotse Glacier (circled 2); **Group** 3 Lhotse Nup Glacier (circled 3); **Group** 4 Ama Dablam Glacier (circled 4); and **Group** 5 Nuptse Glacier (circled 5).

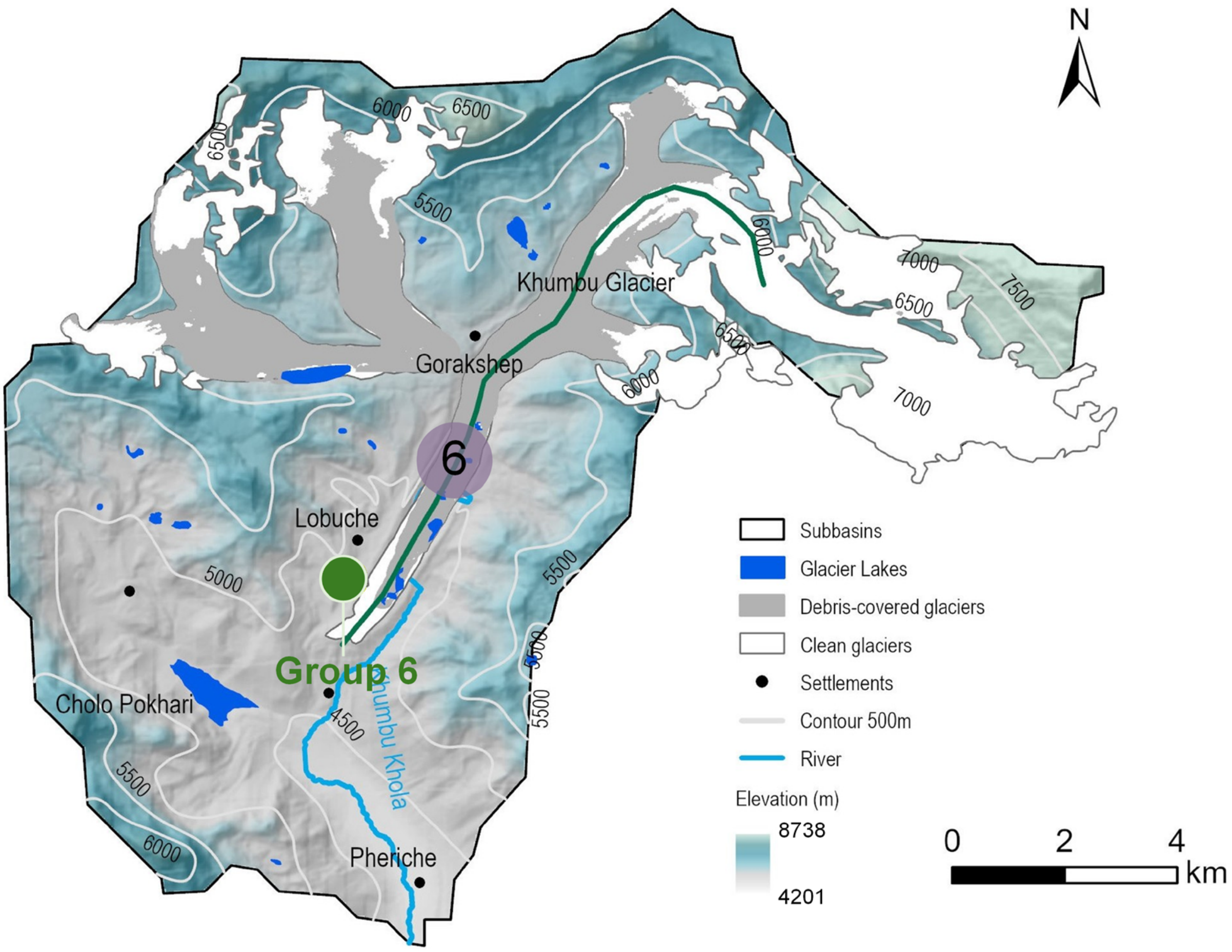

Fig. S2 | Criteria for glacier system classification and spatial distribution of glaciers. Group 6 represents the spatial distribution of the glacier group, and the number corresponds to the glacier branch in Fig. 2. The correspondence is as follows: **Group 6** Khumbu Glacier (circled 6).

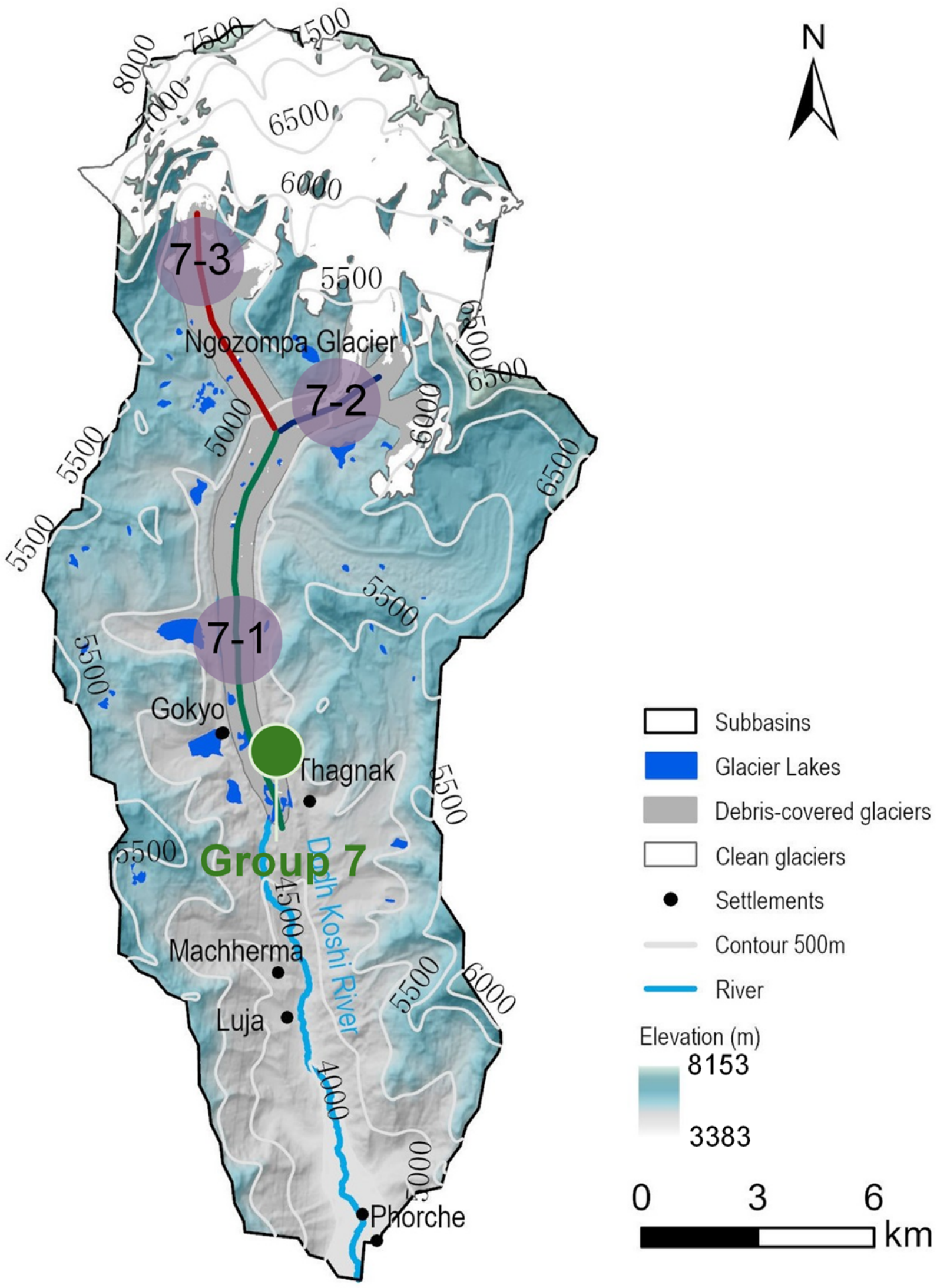

Fig. S3 | Criteria for glacier system classification and spatial distribution of glaciers. Group 7 represents the spatial distribution of the glacier group, and the number corresponds to the glacier branch in Fig. 2. The correspondence is as follows: **Group** 7 Ngozumpa Glacier (circled 7-1, 7-2 and 7-3).

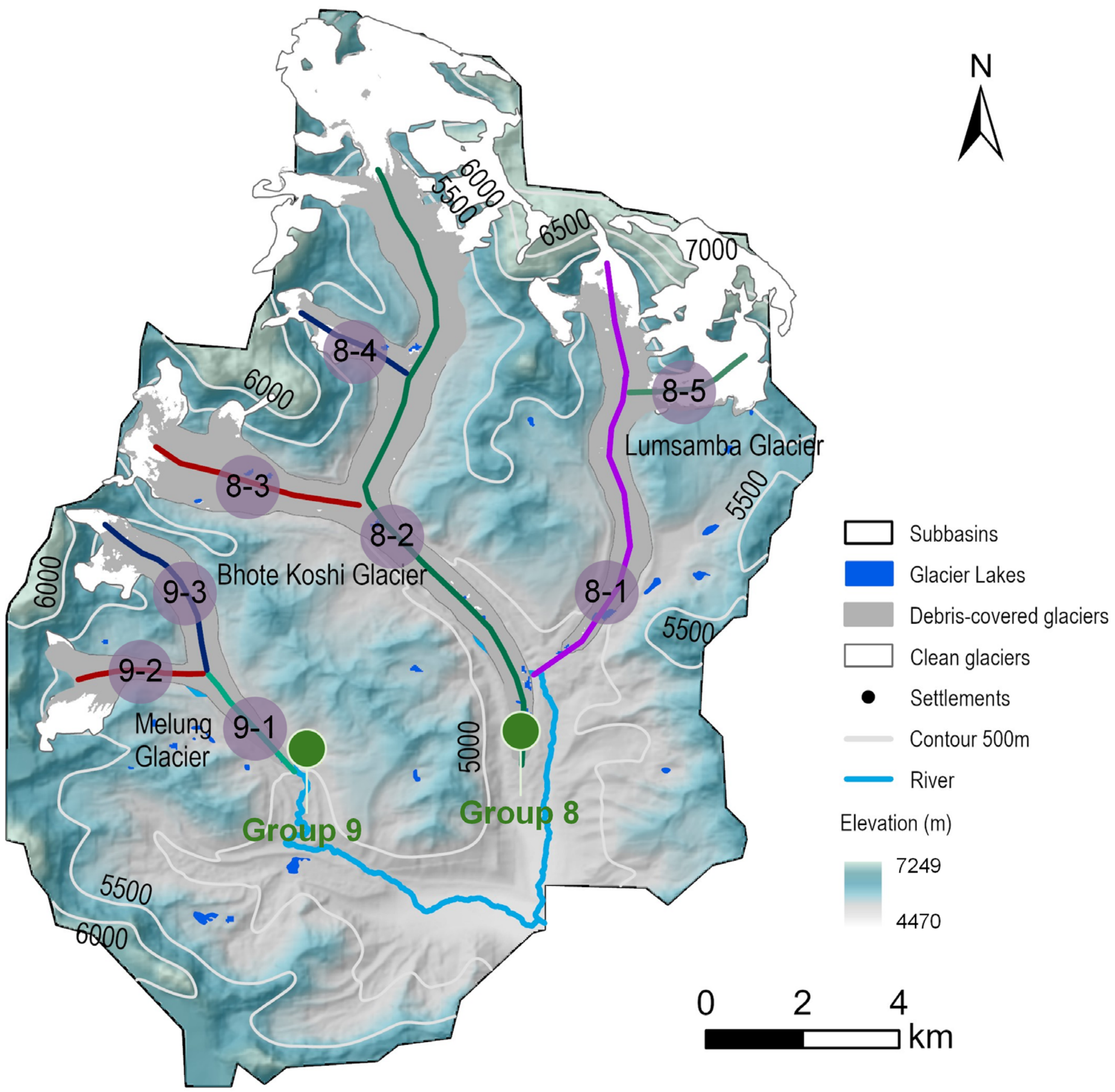

Fig. S4 | Criteria for glacier system classification and spatial distribution of glaciers. Group 8-9 represents the spatial distribution of the glacier group, and the number corresponds to the glacier branch in Fig. 2. The correspondence is as follows: **Group** 8 Bhote Koshi Glacier (circled 8-2, 8-3 and 8-4) and Lumsamba Glacier (circled 8-1 and 8-5). **Group** 9 Melung Glacier (circled 9-1, 9-2 and 9-3)

Comment 2.51: Lines 181-183: The reported values here are hard to understand out of context so I would just cite the figure.

Response: Thank you for your suggestion. Based on your advice, we have removed these statistical figures and directly referred to the figure.

Comment 2.52: Lines 183-185: "The time series of lake area changes, together with meteorological observations at the sites, indicate that supraglacial lake expansion and seasonal signals are highly sensitive to climate anomalies." – how is this conclusion arrived at? To me it doesn't seem to follow logically from the text immediately beforehand, or Figure 3. More explanation and justification is required.

Response: Thank you very much for pointing out this issue. As you mentioned, our original expression was indeed not rigorous and did not clearly convey the seasonal variations of the lakes or their relationship with climate. Therefore, we have revised the wording to be more precise, avoiding overinterpretation and only presenting the conclusions supported by our results *(For details, please see the revised manuscript: Lines 207-211 (This has also been presented in this response))*.

We have revised the paragraph as follows:

> ***Lines 207-211 IN THE REVISED MANUSCRIPT:*** *The time series of lake area changes, together with on-site meteorological observations, indicate that SGLs in the Sagarmatha National Park exhibit typical seasonal variations, with lake areas reaching their annual maximum during the summer monsoon, followed by continuous shrinkage in the post-monsoon and winter months. Overall, the lake area shows an increasing long-term trend and exhibits a lagged correlation with SST anomalies.*

Comment 2.53: Line 198: How were "downstream drainage events" determined from the optical satellite images? Reduction in lake area? I don't find an explanation of this in the methods.

Response: Thank you very much for your comment. The identification of downstream drainage events is based on reductions in lake area. For each glacier group, we extracted lake extents from optical satellite images (Planet data) and identified drainage events by comparing lake

area changes between consecutive years or within a single year. Unlike drainage events of large moraine-dammed lakes, the drainage of supraglacial lakes generally occurs gradually on a seasonal timescale rather than instantaneously. Therefore, the drainage events we report reflect long-term trends in lake area changes. Following your suggestion, we have explicitly clarified that the identification of drainage events is based on lake area changes *(For details, please see the revised manuscript: Lines 225-227, Lines 482-483 (This has also been presented in this response))*. In addition, for Fig. 4, we focused on the four largest lakes and identified changes in lake area that were significantly higher than in normal years as key drainage events, in order to highlight the main drainage events.

We have revised the paragraph as follows:

> ***Lines 225-227 IN THE REVISED MANUSCRIPT:*** *We used complete Planet imagery from 2016 to 2024 to capture all downstream drainage events of the nine glacier groups by tracking intra-annual dynamics of lake extent, together with full Sentinel-1 data to measure surface deformation at glacier termini.*

> ***Lines 482-483:*** *Planet imagery, with its high spatiotemporal resolution and frequent revisit capability, enables the analysis of individual lake filling and drainage events.*

Comment 2.54: Lines 219-222: The captions for Figure 4 panels a and b are not very informative at all.

Response: Thank you for pointing out this issue. Based on your suggestion, we have added detailed descriptions of panels a and b in the figure caption *(As shown in the figure and its caption)*.

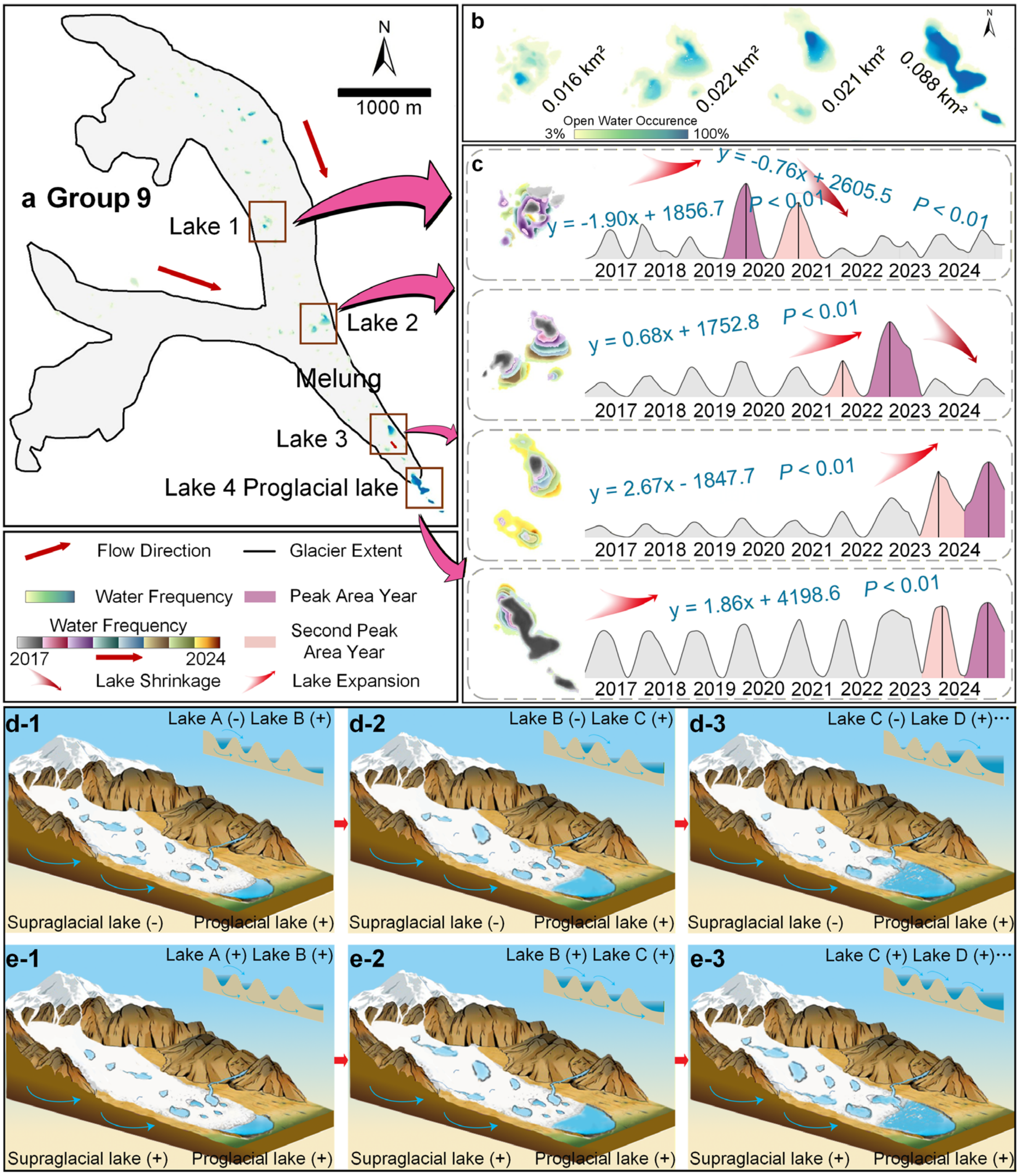

Fig.4 | Hydrological connectivity and cascading drainage among supraglacial lakes. a Frequency of lake occurrence in Glacier Group 9 from 2016 to 2024, with the four largest lakes highlighted (the four largest lakes). **b** Lakes are arranged from left to right corresponding to Lakes 1–4 in panel a, with the maximum area and occurrence frequency shown for each lake over the study period. **c** Annual frequency of SGLs and their cascading drainage processes, with locally weighted scatterplot smoothing fitted curves for lakes of interest. The x-axis of the ridgeline plot represents the time series of SGL area, with filled areas indicating the first and

second peaks of lake area. The frequency plots on the left use different gradient colors to show the annual distribution of lakes, illustrating upstream lake incision and downstream proglacial lake aggradation. **d** a pattern of downstream lake expansion accompanied by upstream lake decline. **e** a pattern of downstream lake expansion without upstream lake decline.

Comment 2.55: Lines 229-230: This seems to be methods.

Response: Thank you for your comment. Following your suggestion, we have moved this description to the methods.

Comment 2.56: Line 233: "…temporal and magnitude characteristics of adjacent glaciers" – of the lakes on those glaciers?

Response: Thank you for your comment. This refers to the differences in flow velocity between adjacent glaciers, specifically the differences between the central lines of glaciers in different groups, rather than the glacial lakes on the glaciers. Based on your suggestion, we have revised this section to improve the readability of our manuscript *(For details, please refer to the revised manuscript: Lines 258-260, Lines 262-263 (This has also been presented in this response)).*

We have revised the paragraph as follows:

> ***Lines 258-260 IN THE REVISED MANUSCRIPT:*** *We obtained surface velocity variations for nine glacier groups by extracting ice surface velocity along their centerlines.*
>
> ***Lines 262-263:*** *[…] we observed substantial heterogeneity in the temporal and magnitude characteristics of the nine groups of glaciers*

Comment 2.57: Line 236: "During historical periods…" – when is this referring to? It's a bit confusing because you then seem to report data, presumably from this study. Clarification is needed.

Response: Thank you for your comment. As you noted, these data come from this study. In response to the issue you raised, we have clarified the time frame, namely from 2016 to 2024.

(For details, please refer to the revised manuscript: Lines 265-266 (This has also been presented in this response)).

We have revised the paragraph as follows:

> ***Lines 265-266 IN THE REVISED MANUSCRIPT:*** *For glaciers that experienced SGL outburst events between 2016 and 2024, […]*

Comment 2.58: Lines 236-240: I'm not sure what these mean velocities are being reported for here? Some context is needed.

Response: Thank you for your comment. We apologize for not clearly specifying the location of velocity extraction, which made the context unclear. The velocities here were all extracted along the glacier central line. To provide readers with a clearer understanding, we have added the relevant explanation in the manuscript *(For details, please refer to the revised manuscript: Lines 258-260 (This has also been presented in this response))*.

We have revised the paragraph as follows:

> ***Lines 258-260 IN THE REVISED MANUSCRIPT:*** *We obtained surface velocity variations for nine glacier groups by extracting ice surface velocity along their centerlines. […]*

Comment 2.59: Lines 243-245: "On Glacier No. 9, we detected pronounced flow accelerations during March–May 2017 (0.19 m day^{-1}) and April–May 2019 (0.18 m day^{-1})" – for comparison, what are representative velocities outside of these periods?

Response: Thank you very much for your comment. Following your suggestion, we have added glacier velocity data for the period between these two intervals, thus covering the entire timeline including pre-monsoon, monsoon, post-monsoon, and winter, to ensure a complete set of comparable data *(For details, please refer to the revised manuscript: Lines 274-275 (This has also been presented in this response))*.

We have revised the paragraph as follows:

> ***Lines 274-275 IN THE REVISED MANUSCRIPT:*** *In comparison, the velocities were 0.04 m day^{-1} from May to October 2017, 0.10 m day^{-1} from November 2017 to January 2018, and 0.06 m day^{-1} from January to March 2018.*

Comment 2.60: Line 251: "…we recorded recurrent glacier surges" – I'm not sure which glacier/plot this is referring to as Khumbu is not seemingly plotted in the referenced figure – but either way, I would suggest the authors reflect on whether these are 'glacier surges' based on existing definitions, e.g. https://doi.org/10.3189/2015JoG14J136. To me, none of the velocity plots seem to identify glacier surges (orders of magnitude velocity increases for extended periods of time, e.g. months to years). What are the timescales of the "recurrent glacier surges", i.e. how often do they recur, for how long. All of this information would help to support such an interpretation. That said, I understand this is not the main focus of the paper, so I would perhaps suggest the authors reconsider whether introducing this here with insufficient detail is necessary for the overall study.

Response: Thank you very much for pointing out this issue. Based on your reference, we have confirmed that this is not a glacier surge, but rather a higher glacier acceleration compared to other regions. We apologize for the inaccurate wording. As you mentioned, this is not the main focus of the paper. Following your suggestion, we have removed the insufficiently detailed discussion to avoid overinterpretation.

Comment 2.61: Line 255: "surface deformations" - as noted previously, I think a clearer definition of this term is needed. Is this horizontal, vertical change or both? Is this on the glacier itself, or in moraine areas, or both (e.g. debris-covered ice)?

Response: We apologize for the insufficient detail in the Methods section. We have revised the Methods to emphasize that the area for deformation monitoring is primarily located at the terminal moraines of the glacier terminus. We also clarified that the deformation here refers to motion in the radar line-of-sight direction and explained the relationship between this motion

and vertical displacement *(Please see the revised manuscript: Lines 553-556, Lines 569-576 (This has also been presented in this response))*.

We have revised the paragraph as follows

We have revised the paragraph as follows (Comments 2.4 & 2.28 & 2.61–2.64 are similar issues):

> ***Lines 553-556 IN THE REVISED MANUSCRIPT:*** *Therefore, we applied Sentinel-2 imagery to monitor the seasonal dynamics of glacier flow (glacier surface velocity) and used 233 Sentinel-1 synthetic aperture radar (SAR) images to map terminal moraine deformation at the glacier termini.*

> ***Lines 569-576:*** *[...] Singular value decomposition (SVD) is then applied to convert the multi-master interferogram set into a single-reference time series[74], with unwrapped phase converted into LOS displacement. In the LOS direction, negative values indicate motion away from the satellite, whereas positive values indicate motion toward it. In this study, these deformation patterns manifest as subsidence and deposition in the glacier terminus zone, respectively. Given that InSAR measurements are acquired along the LOS direction and based on the geometric projection relationship, the vertical displacement can be approximated as* $dvertical = dLOS/COS\theta$*, where* θ *is the incidence angle[75]. All processing was performed using GAMMA software.*

Comment 2.62: Line 257: “average cumulative displacement” - is this vertical or horizontal change? I am finding it hard to understand what these changes are referring to.

Response: We apologize for the lack of detail in the Methods section. We have revised the Methods accordingly. Additionally, we clarified that the cumulative displacement here refers to motion in the radar line-of-sight direction and explained the relationship between this motion and vertical changes *(Please see the revised manuscript: Lines 553-556, Lines 569-576 (This has also been presented in this response))*.

We have revised the paragraph as follows (Comments 2.4 & 2.28 & 2.61–2.64 are similar issues):

> ***Lines 553-556 IN THE REVISED MANUSCRIPT:*** *Therefore, we applied Sentinel-2 imagery to monitor the seasonal dynamics of glacier flow (glacier surface velocity) and used 233 Sentinel-1 synthetic aperture radar (SAR) images to map terminal moraine deformation at the glacier termini.*
>
> ***Lines 569-576:*** *[...] Singular value decomposition (SVD) is then applied to convert the multi-master interferogram set into a single-reference time series[74], with unwrapped phase converted into LOS displacement. In the LOS direction, negative values indicate motion away from the satellite, whereas positive values indicate motion toward it. In this study, these deformation patterns manifest as subsidence and deposition in the glacier terminus zone, respectively. Given that InSAR measurements are acquired along the LOS direction and based on the geometric projection relationship, the vertical displacement can be approximated as* $dvertical = dLOS/COS\theta$, *where* θ *is the incidence angle[75]. All processing was performed using GAMMA software.*

Comment 2.63: Line 259: “minor subsidence or deposition of terminal debris” - this suggests vertical change at least for ‘minor subsidence’. What impact does “deposition of terminal debris" have on the surface change?

Response: Thank you very much for your comment. Although the displacement we retrieved represents deformation along the radar line-of-sight (LOS), it largely reflects vertical surface changes due to the satellite observation geometry. In InSAR observations, surface uplift caused by deposition is typically expressed as motion toward the satellite (positive LOS displacement), whereas subsidence is usually indicated by motion away from the satellite (negative LOS displacement). Regarding the deposition of terminal moraine debris, its effects are mainly manifested through changes in surface elevation and morphology. During glacier retreat or ice mass collapse, large amounts of debris—including boulders, gravel, and fine materials—accumulate in moraine ridges or at the glacier terminus. During glacier retreat or collapse, large

amounts of debris—including boulders, gravel, and fine sediments—accumulate at moraine ridges or the glacier terminus. This process increases debris thickness and leads to localized surface uplift. *(glacial landform https://www.britannica.com/science/glacial-landform)*. In our InSAR-derived results, such processes may be reflected as positive deformation signals along the LOS direction. To clearly convey this principle, we have added a corresponding description of the underlying mechanism in the Methods section *(For details, please see the revised manuscript: Lines 557-576 (This has also been presented in this response))*.

We have revised the paragraph as follows (Comments 2.4 & 2.28 & 2.61–2.64 are similar issues):

> ***Lines 557-576 IN THE REVISED MANUSCRIPT:*** *The SBAS-InSAR algorithm is a time-series InSAR approach that generates a set of small-baseline interferograms using a multi-master strategy with appropriately defined spatiotemporal baseline thresholds, enhancing interferometric coherence and enabling millimeter- to centimeter-scale monitoring of surface deformation. N+1 single-look complex (SLC) SAR images acquired over the study area at consecutive time points* $t0,\cdots,tN$ *are processed by selecting a single master image, to which all other images are co-registered and a unified reference grid is established. Differential interferograms are generated from the SLC images, and topographic phase is removed using an external digital elevation model (e.g., AW3D 30 m). Flat and highly coherent stable points (e.g., bedrock) are used as zero-displacement reference points. Following filtering and phase unwrapping, continuous phase time series are obtained for each coherent target, comprising deformation, residual topography, atmospheric, orbital, and noise components. Orbital errors are corrected via least-squares fitting, residual topography is estimated from the linear relationship between perpendicular baseline and elevation, and atmospheric contributions are separated using temporal high-pass and spatial low-pass filtering. Singular value decomposition (SVD) is then applied to convert the multi-master interferogram set into a single-reference time series*[74]*, with unwrapped phase converted into LOS displacement. In the LOS direction, negative values indicate motion away from the satellite, whereas positive values indicate*

motion toward it. In this study, these deformation patterns manifest as subsidence and deposition in the glacier terminus zone, respectively. Given that InSAR measurements are acquired along the LOS direction and based on the geometric projection relationship, the vertical displacement can be approximated as $dvertical = dLOS/COS\theta$, *where* θ *is the incidence angle*[75]*. All processing was performed using GAMMA software.*

Comment 2.64: Lines 256-276: The reported values of change in this section seems very small, in all cases <0.5 m. I remain unclear if this is vertical or horizontal change, and over what timescales, but also the source data used appears to be Sentinel 1/2 - which has considerably larger pixel sizes than the reported changes. I presume there is a methodological explanation for this, but having read the associated methods section I am still struggling to understand this (the methods largely relate to ice velocities).

Response: Thank you for your comment. In this study, we primarily employed the InSAR method. By utilizing the continuity of interferometric phase and time-series analysis, it is possible to achieve sub-pixel level monitoring of surface displacement. Therefore, the measured displacement can be much smaller than the size of a single pixel and is not equivalent to pixel resolution. We have revised the relevant description in the Methods section to ensure that readers can clearly understand our approach and the accuracy of the displacement measurements *(For details, please see the revised manuscript: Lines 557-576 (This has also been presented in this response)).*

We have revised the paragraph as follows (Comments 2.4 & 2.28 & 2.61 – 2.64 are similar issues):

Lines 557-576 IN THE REVISED MANUSCRIPT: *The SBAS-InSAR algorithm is a time-series InSAR approach that generates a set of small-baseline interferograms using a multi-master strategy with appropriately defined spatiotemporal baseline thresholds, enhancing interferometric coherence and enabling millimeter- to centimeter-scale monitoring of surface deformation. N+1 single-look complex (SLC) SAR images acquired over the study area at consecutive time points* $t0,\cdots,tN$ *are processed by selecting a single master*

image, to which all other images are co-registered and a unified reference grid is established. Differential interferograms are generated from the SLC images, and topographic phase is removed using an external digital elevation model (e.g., AW3D 30 m). Flat and highly coherent stable points (e.g., bedrock) are used as zero-displacement reference points. Following filtering and phase unwrapping, continuous phase time series are obtained for each coherent target, comprising deformation, residual topography, atmospheric, orbital, and noise components. Orbital errors are corrected via least-squares fitting, residual topography is estimated from the linear relationship between perpendicular baseline and elevation, and atmospheric contributions are separated using temporal high-pass and spatial low-pass filtering. Singular value decomposition (SVD) is then applied to convert the multi-master interferogram set into a single-reference time series[74], with unwrapped phase converted into LOS displacement. In the LOS direction, negative values indicate motion away from the satellite, whereas positive values indicate motion toward it. In this study, these deformation patterns manifest as subsidence and deposition in the glacier terminus zone, respectively. Given that InSAR measurements are acquired along the LOS direction and based on the geometric projection relationship, the vertical displacement can be approximated as $dvertical = dLOS/COS\theta$*, where* θ *is the incidence angle[75]. All processing was performed using GAMMA software.*

Comment 2.65: Lines 284-288: I am finding it hard to follow these values as they are reported here. What dates are being compared? I understand one of the values is 2022-23 in each case, but what is the other?

Response: Thank you for pointing out this issue. One of the values here corresponds to 2022–23, while the other represents the highest year excluding 2022–23, that is, the glacial lake area in the year with the second-highest area. We apologize for not explaining this clearly, which may have caused confusion. In response to your comment, we have made the necessary revisions *(For details, please see the revised manuscript: Lines 314-317 (This has also been presented in this response))*.

We have revised the paragraph as follows:

Lines 314-317 IN THE REVISED MANUSCRIPT: *To highlight the anomalous expansions in 2022–2023, we report, for each SGL on a glacier, the maximum lake area observed during 2022–2023, alongside the maximum area recorded in all other years (i.e., the second-largest recorded extent). Corresponding values were as follows:*

Discussion

Comment 2.66: Line 325: "in turn reshapes the bedrock at glacier termini" - what evidence is this based on? Where are you measuring bedrock changes?

Response: Thank you for your comment. The phrase "in turn reshapes the bedrock at glacier termini" was originally cited from previous studies to support our results *(Widespread seasonal speed-up of west Antarctic Peninsula glaciers from 2014 to 2021, https://doi.org/10.1038/s41561-023-01131-4. Contrasting geometric and dynamic evolution of lake and land-terminating glaciers in the central Himalaya, https://doi.org/10.1016/j.gloplacha.2018.05.006).* However, our presentation did not clearly convey the full logical chain, making this statement appear abrupt. The complete process is that meltwater reduces basal traction and induces hydraulic uplift, thereby promoting glacier flow acceleration. Under the combined effects of accelerated flow, enhanced longitudinal strain, dynamic thinning, and fracture-driven frontal retreat, calving becomes dominant. During the processes of ice thinning and movement, the glacier may indirectly "reshape" the bedrock at the terminus through erosion and transport. To clarify our reasoning, we have revised the text and no longer emphasize "reshaping bedrock." Instead, we refer to changes in the geometry of the glacier terminus, which is more accurate, as it encompasses both potential bedrock modification through erosional processes and geomorphic changes caused by glacier advance or retreat *(For details, please refer to the revised manuscript: Lines 357-366(This has also been presented in this response)).*

We have revised the paragraph as follows:

Lines 357-366 IN THE REVISED MANUSCRIPT: *Previous studies indicate that seasonal melting of debris-covered surfaces and glacier ice generates substantial meltwater. which drains through crevasses into the englacial hydrological system and is routed toward the glacier margins or bed. This process reduces basal traction and induces hydraulic uplift, both of which promote ice-flow acceleration*[48]*. Under the combined effects of accelerated flow, enhanced longitudinal strain, dynamic thinning and crevasse-driven frontal retreat, calving becomes dominant. Continued extensional flow deepens crevasses in the glacier terminus, while thinning reduces freeboard height (the elevation of the ice surface above the lake level), thereby promoting mechanical calving and frontal retreat and increasing the likelihood of ice-dam instability and failure. These processes collectively alter the geometry of glacier termini and are accompanied by surface lowering driven by ice thinning and dynamic adjustment*[42,48-50]*. [...]*

Comment 2.67: Lines 325-326: "such irregular and sudden collapses..." - I'm not clear what you are referring to here. What is collapsing? The previous sentence mentions only ice acceleration. The rest of the sentence seems to suggest this is related to glacier retreat (creating room for lakes) but this is really not clear from these sentences.

Response: We sincerely apologize that our wording, expression, and logic were not clearly presented. The phrase "such irregular and sudden collapses…" refers to the mechanical disintegration at the glacier front (terminus) during glacier retreat, including ice calving, melting, retreat, and the deepening of crevasses. According to the study *(Supraglacial Ponds Regulate Runoff From Himalayan Debris-Covered Glaciers, https://agupubs.onlinelibrary.wiley.com/doi/10.1002/2017GL075398)*, debris cover on glaciers plays a critical role in modulating the response to climatic forcing. Debris can either accelerate or delay melting, thereby influencing the collapse of glacier drainage pathways and promoting the formation of supraglacial lakes and ponds *(as introduced in the Introduction, for details, please refer to the revised manuscript: Lines 96-100 (This has also been presented in this response))*. On the other hand, the seasonal increase in meltwater and the expansion of glacial lakes are often accompanied by glacier flow acceleration. During this stage, rising lake

levels or thinning of the host glacier can induce crevasse formation, which accelerates frontal retreat and steepens the glacier surface—processes that are often associated with "collapse." Therefore, the dynamic changes of lakes on debris-covered glaciers are closely linked to glacier dynamics. We sincerely apologize for the incomplete expression in this section. Following your suggestion, we have thoroughly revised the text to clearly convey our intended meaning *(For details, please see the revised manuscript: Lines 96-100, Lines 357-366 (This has also been presented in this response))*.

We have revised the paragraph as follows:

> ***Lines 96-100 IN THE REVISED MANUSCRIPT:*** *In Sagarmatha National Park, the ablation zones of glaciers are almost entirely covered by continuous debris, which plays a key role in modulating glacier responses to climatic forcing[18,32]. During the retreat of debris-covered glaciers, debris can either accelerate or retard ice melt depending on its thickness; together with the dynamic glacier surface topography, this promotes enhanced differential ablation and the formation of SGLs, thereby increasing the risk of mountain hazards[18].*
>
> ***Lines 357-366:*** *[...]Previous studies indicate that seasonal melting of debris-covered surfaces and glacier ice generates substantial meltwater. which drains through crevasses into the englacial hydrological system and is routed toward the glacier margins or bed. This process reduces basal traction and induces hydraulic uplift, both of which promote ice-flow acceleration[48]. Under the combined effects of accelerated flow, enhanced longitudinal strain, dynamic thinning and crevasse-driven frontal retreat, calving becomes dominant. Continued extensional flow deepens crevasses in the glacier terminus, while thinning reduces freeboard height (the elevation of the ice surface above the lake level), thereby promoting mechanical calving and frontal retreat and increasing the likelihood of ice-dam instability and failure. These processes collectively alter the geometry of glacier termini and are accompanied by surface lowering driven by ice thinning and dynamic adjustment[42,48-50]. [...]*

Comment 2.68: Lines 332-333: “The reduction in basal traction drives surface subsidence, with glacier advance creating the necessary bedrock depressions and moraine dams.” - I am struggling to follow this argument. Ice acceleration leads to lower surface elevations? Why is this? And how does advance create “necessary bedrock depressions and moraine dams”? This all needs to be explained in a much clearer way.

Response: Thank you for pointing out this issue. We acknowledge that the logical sequence was not clearly presented, which led to confusion. Our original intention was to explain that, under global warming, increased seasonal meltwater enhances basal sliding and thereby promotes glacier acceleration *(For details, please refer to the revised manuscript: Lines 376-385 (This has also been presented in this response)*). At the same time, during glacier mass loss and thinning, surface subsidence may occur *(reference: Widespread seasonal speed-up of west Antarctic Peninsula glaciers from 2014 to 2021, https://doi.org/10.1038/s41561-023-01131-4. Contrasting geometric and dynamic evolution of lake and land-terminating glaciers in the central Himalaya, https://doi.org/10.1016/j.gloplacha.2018.05.006)*. These processes occur simultaneously under a warming climate; however, our wording may have incorrectly suggested a causal relationship. Regarding the statement on “how glacier advance contributes to the formation of bedrock depressions and moraine dams,” when glaciers advance or retreat, the ice exerts pressure on the bedrock in the terminus region, potentially forming depressions in weaker or more fractured bedrock. Meanwhile, debris transported by the glacier can accumulate at the glacier front during advance, forming moraine dams or barriers. Upon further consideration, we found that this part deviates from the main focus of our study. This section is intended to emphasize the relationship between seasonal meltwater, supraglacial lakes, and glacier velocity. Therefore, we have removed this content and refocused on the core of our study, avoiding unnecessary extension beyond our main scope. *(For details, please refer to the revised manuscript: Lines 357-366, Lines 367-373, Lines 374-377, Lines 384-390 (This has also been presented in this response))*.

We have revised the paragraph as follows:

Lines 357-366 IN THE REVISED MANUSCRIPT: *[...]Previous studies indicate that seasonal melting of debris-covered surfaces and glacier ice generates substantial meltwater. which drains through crevasses into the englacial hydrological system and is routed toward the glacier margins or bed. This process reduces basal traction and induces hydraulic uplift, both of which promote ice-flow acceleration*[48]*. Under the combined effects of accelerated flow, enhanced longitudinal strain, dynamic thinning and crevasse-driven frontal retreat, calving becomes dominant. Continued extensional flow deepens crevasses in the glacier terminus, while thinning reduces freeboard height (the elevation of the ice surface above the lake level), thereby promoting mechanical calving and frontal retreat and increasing the likelihood of ice-dam instability and failure. These processes collectively alter the geometry of glacier termini and are accompanied by surface lowering driven by ice thinning and dynamic adjustment*[42,48-50]*. [...]*

Lines 367-373: *These patterns support King's two-stage model of glacier-lake evolution in the Himalaya*[48]*. During the lake expansion stage, rising lake levels or thinning of the host glacier induce crevassing, accelerating frontal retreat, steepening the glacier surface, and enhancing ice-flow velocity. In the later stage of lake growth, sustained ice-front retreat and glacier thinning narrow the glacier, reducing driving stresses while increasing lateral and basal resistance, thereby slowing ice flow and diminishing retreat rates*[48]*.*

Lines 374-377*: At the same time, with future mass loss of glaciers in the Himalaya, glacier retreat and thinning will be accompanied by the formation and expansion of new lakes, as well as accelerated ice flow and instability at glacier termini*[46]*.*

Lines 384-390: *In glaciers hosting SGLs and debris cover, seasonal fluctuations are primarily driven by basal sliding (subglacial water flow) , the level of effective pressure – the difference between ice overburden pressure and subglacial water pressure – and the stabilizing structure formed by supraglacial debris, ice, and terminal moraines*[52,53]*. During the monsoon, rising temperatures and increased precipitation induce melting of ice covered by supraglacial debris and ice margins, as well as meltwater erosion, which*

compromise the support of supraglacial debris and weaken the stability of lake-adjacent moraine or ice dams.

Comment 2.69: Lines 338-339: "In Sagarmatha National Park, the ablation zones of glaciers are almost entirely covered by continuous debris, which contributes to slower ice flow and frequent geohazards." - this is important context for the study glaciers that should come earlier in the paper.

Response: Thank you for your comment. Following your suggestion, we have moved this description to the Introduction.

Comment 2.70: Lines 343-344: "seasonal fluctuations are primarily driven by basal sliding (subglacial water flow) and the support provided by supraglacial debris" - what does "support provided by supraglacial debris" mean here?

Response: Thank you for your comment. In this section, we mainly refer to the viewpoints presented in Wallis et al. and King et al *(Widespread seasonal speed-up of west Antarctic Peninsula glaciers from 2014 to 2021, https://doi.org/10.1038/s41561-023-01131-4. Contrasting geometric and dynamic evolution of lake and land-terminating glaciers in the central Himalaya, https://doi.org/10.1016/j.gloplacha.2018.05.006)*. During glacier retreat (particularly thinning at the terminus), increased surface melting alters the balance between ice overburden pressure and water pressure; this change in pressure conditions may affect glacier flow velocity. In addition, the melting of debris and sediments on debris-covered glaciers contributes to increased meltwater production. This meltwater can erode and weaken the structural support provided by the combined system of surface debris, ice, and terminal moraines, thereby reducing the stability of ice dams and glacier margins surrounding the lakes. We apologize for the lack of clarity in our previous description, which may have made this section difficult to understand. Following your comment, we have revised the text accordingly. *(For details, please refer to the revised manuscript: Lines 357-366 (This has also been presented in this response))*.

We have revised the paragraph as follows:

> ***Lines 357-366 IN THE REVISED MANUSCRIPT:*** *[...]Previous studies indicate that seasonal melting of debris-covered surfaces and glacier ice generates substantial meltwater. which drains through crevasses into the englacial hydrological system and is routed toward the glacier margins or bed. This process reduces basal traction and induces hydraulic uplift, both of which promote ice-flow acceleration[48]. Under the combined effects of accelerated flow, enhanced longitudinal strain, dynamic thinning and crevasse-driven frontal retreat, calving becomes dominant. Continued extensional flow deepens crevasses in the glacier terminus, while thinning reduces freeboard height (the elevation of the ice surface above the lake level), thereby promoting mechanical calving and frontal retreat and increasing the likelihood of ice-dam instability and failure. These processes collectively alter the geometry of glacier termini and are accompanied by surface lowering driven by ice thinning and dynamic adjustment[42,48-50]. [...]*

Figures

Comment 2.71: Figure 1: – I am struggling to understand this figure. It is not clear if this is a schematic diagram or a location map. There is no spatial context beyond a global inset, which is not much help. There are no named locations/peaks/glaciers that would help the reader. I don't understand what the different numbered locations are there do not appear to be any links to supporting datasets that presumably this information has been derived from – and they do not seem to relate to the numbered glaciers in Figure 2. The significance of the precipitation/evaporation/glacial lake/glacier/snow/avalanche/GLOF risk labels is not clear – are these mapped in specific locations for specific reasons? In summary, this figure needs a substantial rethink.

Response: We sincerely apologize for the lack of clarity in the figure, which may have caused confusion. This figure is a 3D scene constructed based on real field conditions and represents the actual topography of the study area. At the same time, we referred to conceptual elements from Xu et al to highlight the impacts of climate change in high mountain regions *(Widespread*

societal and ecological impacts from projected Tibetan Plateau Lake expansion, https://doi.org/10.1038/s41561-024-01446-w). Therefore, the figure was designed as a combination of a location map and a conceptual diagram. Based on your suggestions, we have reconstructed the figure by adding key information such as well-known mountain peaks, the spatial distribution of glaciers in the study area, and specific locations. The numbering in the figure indicates the locations of historical glacial lake outburst floods (GLOFs) and avalanche events within the study area. The labels below correspond to the severity of the impacts caused by these events. In addition, we have marked lakes under artificial monitoring using different colors. These labels are independent and not linked to those in Figure 2.

The labels such as precipitation/evaporation/glacial lakes/glaciers/snow/avalanches/GLOF risk are based on concepts from *Xu et al (Widespread societal and ecological impacts from projected Tibetan Plateau Lake expansion, https://doi.org/10.1038/s41561-024-01446-w)*, illustrating that under climate warming, glacier and snow/ice retreat may lead to increased extreme precipitation, which in turn promotes the expansion of supraglacial lakes and elevates the risks of avalanches and GLOFs. Since the figure is modeled on real terrain, the white areas correspond to actual snow-covered mountains. Accordingly, "Glacier/Snow (−)" and "Avalanche/GLOF (+)" are illustrated over the snow-covered mountain regions, while precipitation is represented as snowfall in high-altitude areas and rainfall in downstream regions, all mapped to specific locations. Our intention was to allow readers to both understand the climate-related challenges in the region and recognize the real geographical context. To improve clarity, we have added more spatial information, expanded the figure caption with detailed explanations, and included five additional figures in the supplementary materials *(For details, please see the revised manuscript and Supplementary Information: Extended Data Fig. 1, Figs. S1-S4)*. Regarding the conceptual elements, we have clarified their meaning in the caption; however, if you consider them unnecessary, we are willing to remove this component and retain the figure solely as a location map.

We have revised the paragraph as follows:

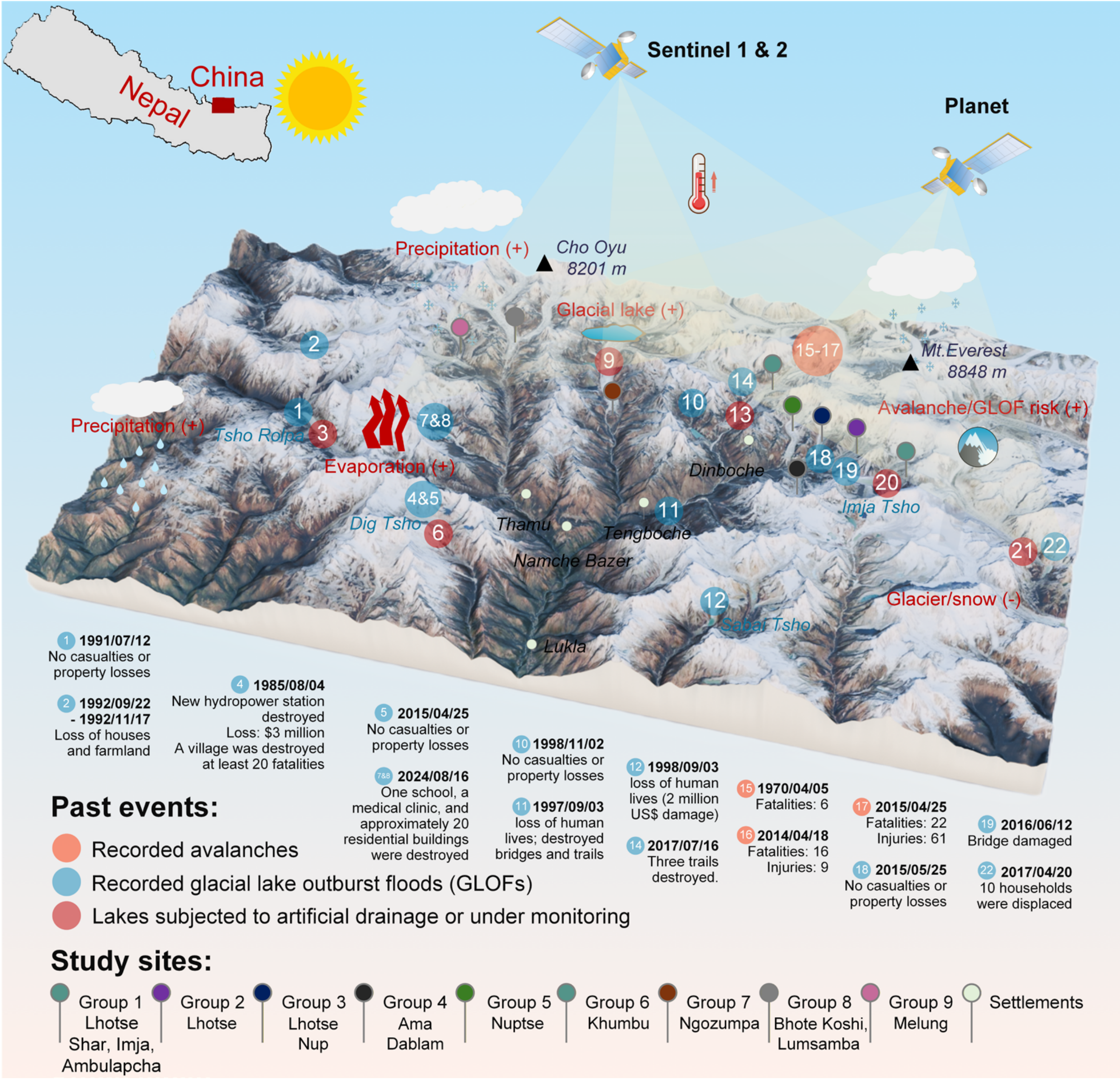

Fig. 1 | Diagram illustrating lake evolution and geological hazards in Sagarmatha National Park. The red labels and text represent the climate-related risks facing this region, namely that under global warming, increased snowfall/rainfall and the melting of glaciers and snowpack lead to the expansion of lake boundaries and heightened risks of GLOFs and avalanches. The numbers in the figure indicate the locations and associated hazard losses of past GLOFs, avalanches, and manually monitored lakes (Data source: Table S1). Glaciers sharing the same drainage outlet were grouped together and labeled as Groups 1–9. The glaciers included in each group are listed as follows: **Group 1** Lhotse Shar, Imja, and Ambulapcha; **Group 2** Lhotse; **Group 3** Lhotse Nup; **Group 4** Ama Dablam; **Group 5** Nuptse; **Group 6** Khumbu; **Group** 7 Ngozumpa; **Group 8** Bhote Koshi and Lumsamba; **Group 9** Melung Glaciers. Extended Data Fig. 1 serves as a supplement to this figure, documenting the spatial

information of clean glaciers, debris-covered glaciers, and runoff.

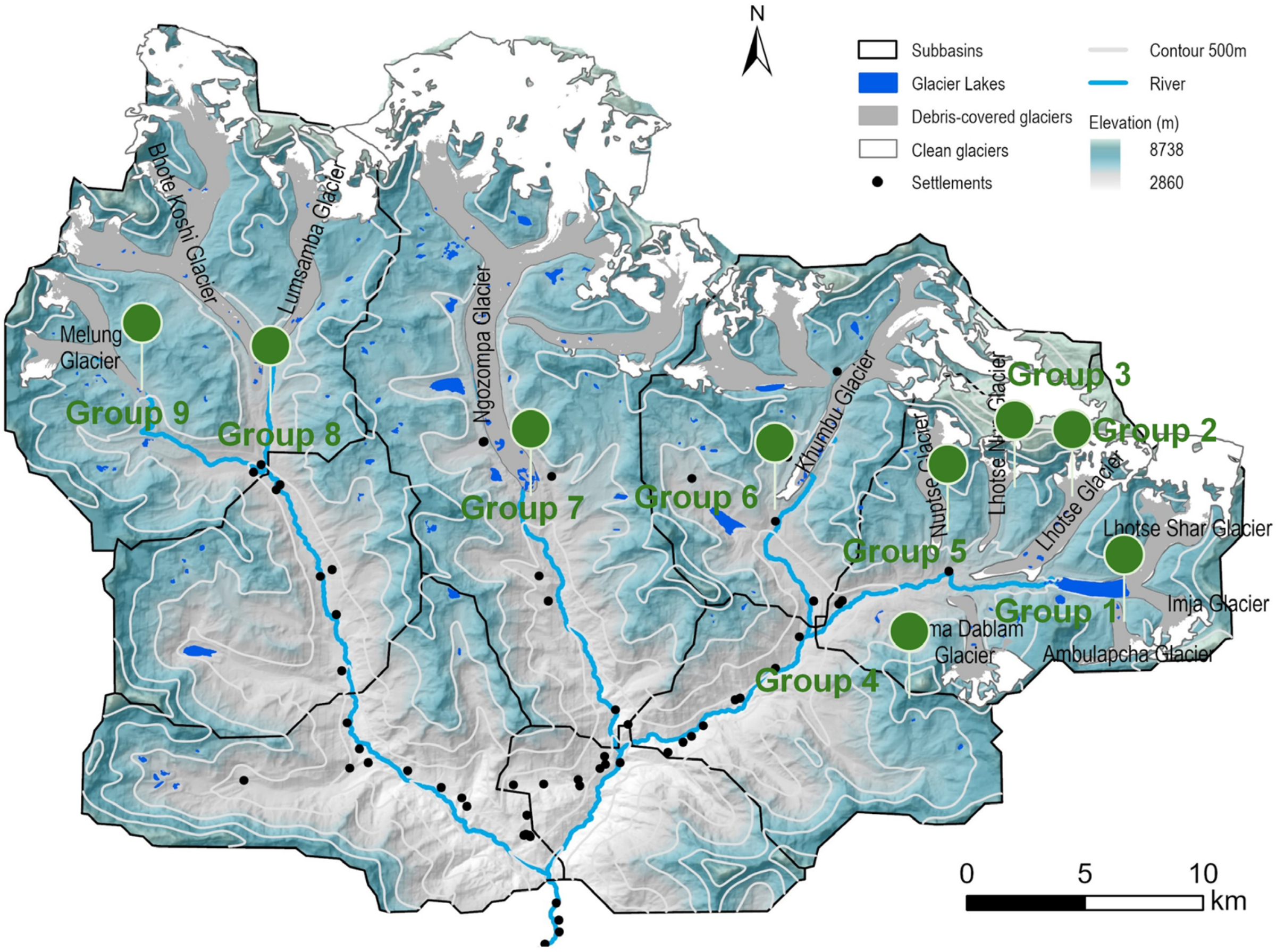

Extended Data Fig. 1 | Spatial distribution and classification of glaciers. Glaciers sharing the same drainage outlet were grouped together and labeled as Glacier Group 1–9. Except for Glacier Groups 1 and 8, which contain two and three individual glaciers, respectively, each of the remaining groups consists of a single glacier. The details are as follows: **Group 1** Lhotse Shar、Imja and Ambulapcha; **Group 2** Lhotse; **Group 3** Lhotse Nup; **Group 4** Ama Dablam; **Group 5** Nuptse; **Group 6** Khumbu; **Group 7** Ngozumpa; **Group 8** Bhote Koshi and Lumsamba and **Group 9** Melung Glaciers**.**

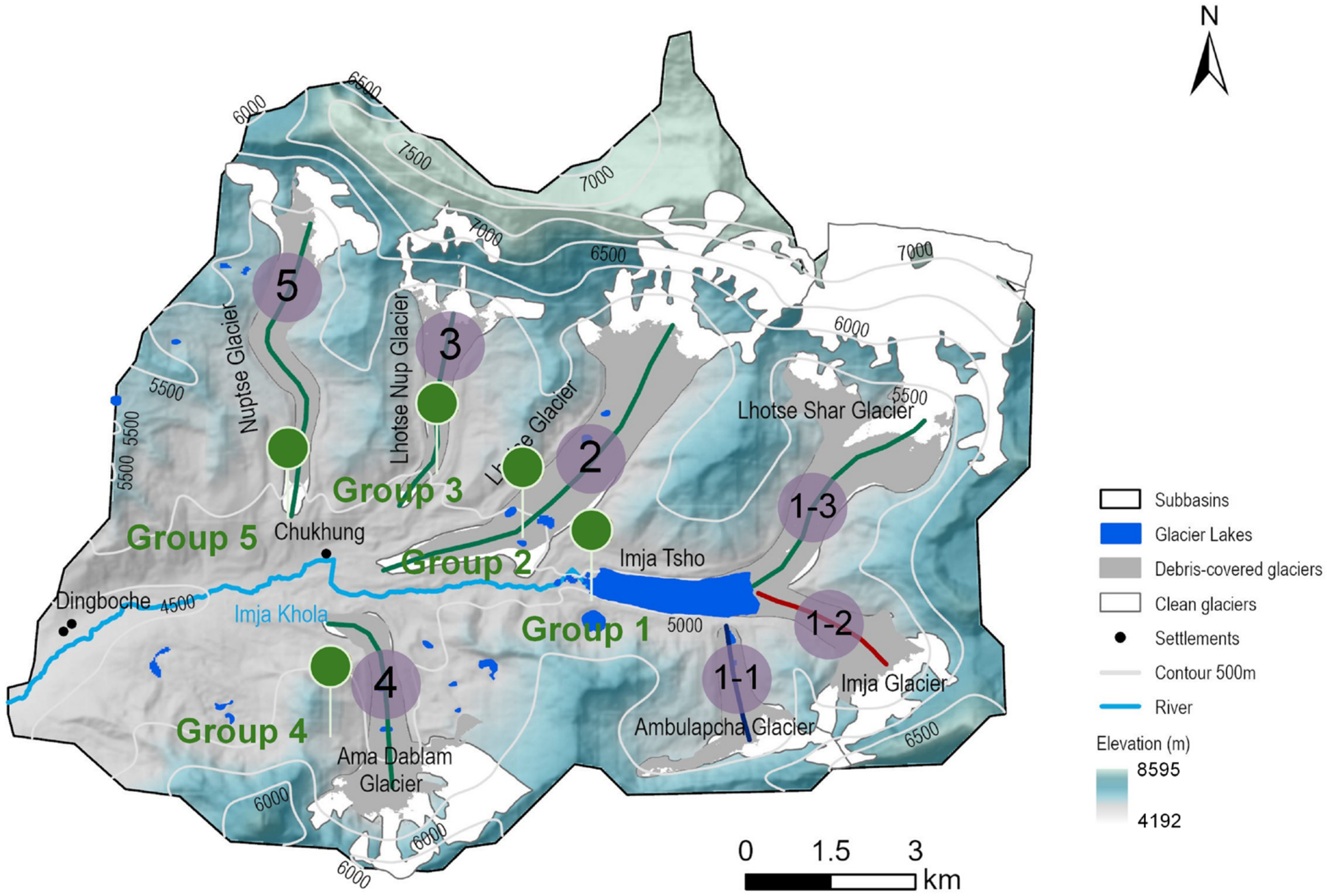

Fig. S1 | Criteria for glacier system classification and spatial distribution of glaciers. Group 1-5 represent the spatial distribution of five glacier systems, and the numbers correspond to the glacier branches in Fig. 2. The relationships are as follows: **Group 1** Lhotse Shar Glacier (circled 1-1), Imja Glacier (circled 1-2), and Ambulapcha Glacier (circled 1-3); **Group** 2 Lhotse Glacier (circled 2); **Group** 3 Lhotse Nup Glacier (circled 3); **Group** 4 Ama Dablam Glacier (circled 4); and **Group** 5 Nuptse Glacier (circled 5).

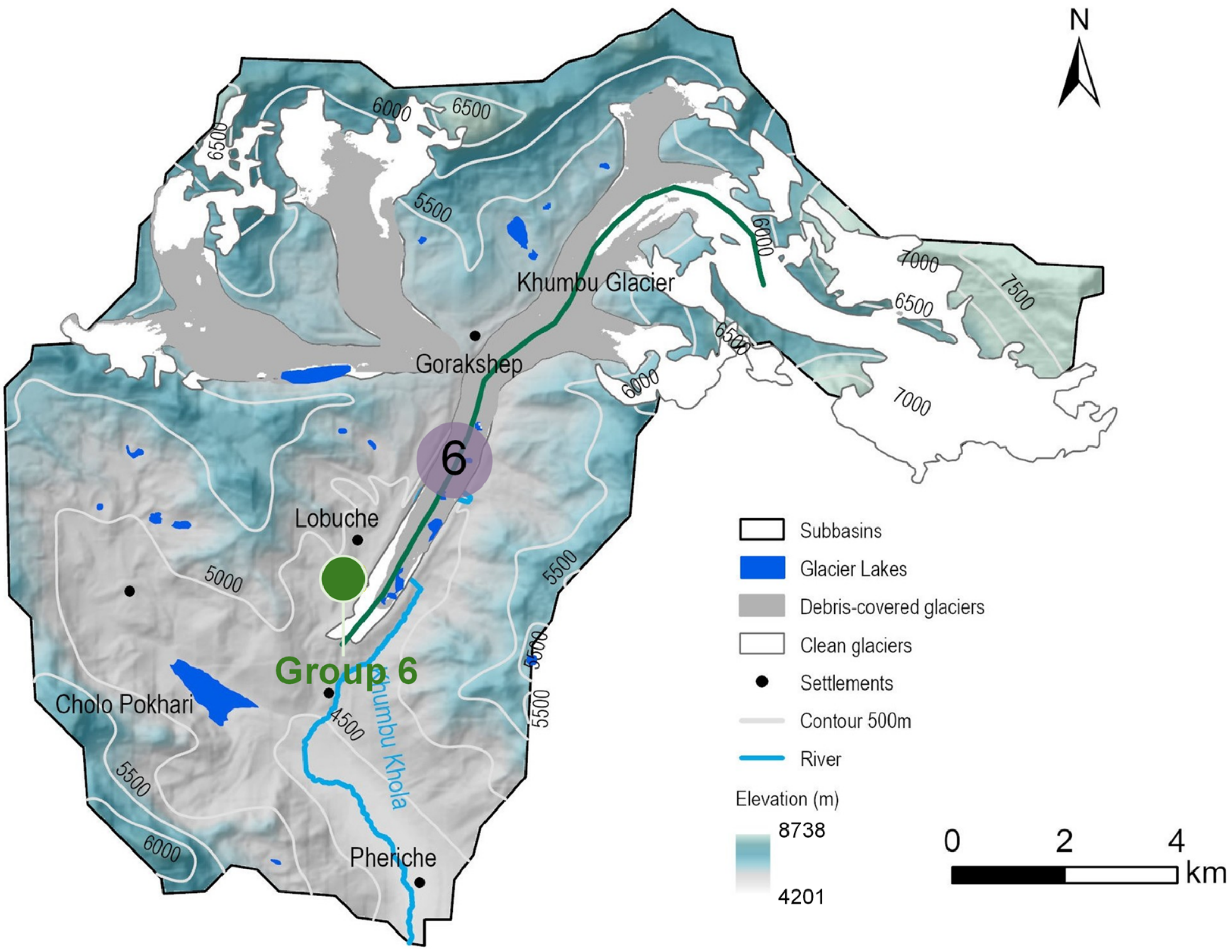

Fig. S2 | Criteria for glacier system classification and spatial distribution of glaciers. Group 6 represents the spatial distribution of the glacier group, and the number corresponds to the glacier branch in Fig. 2. The correspondence is as follows: **Group 6** Khumbu Glacier (circled 6).

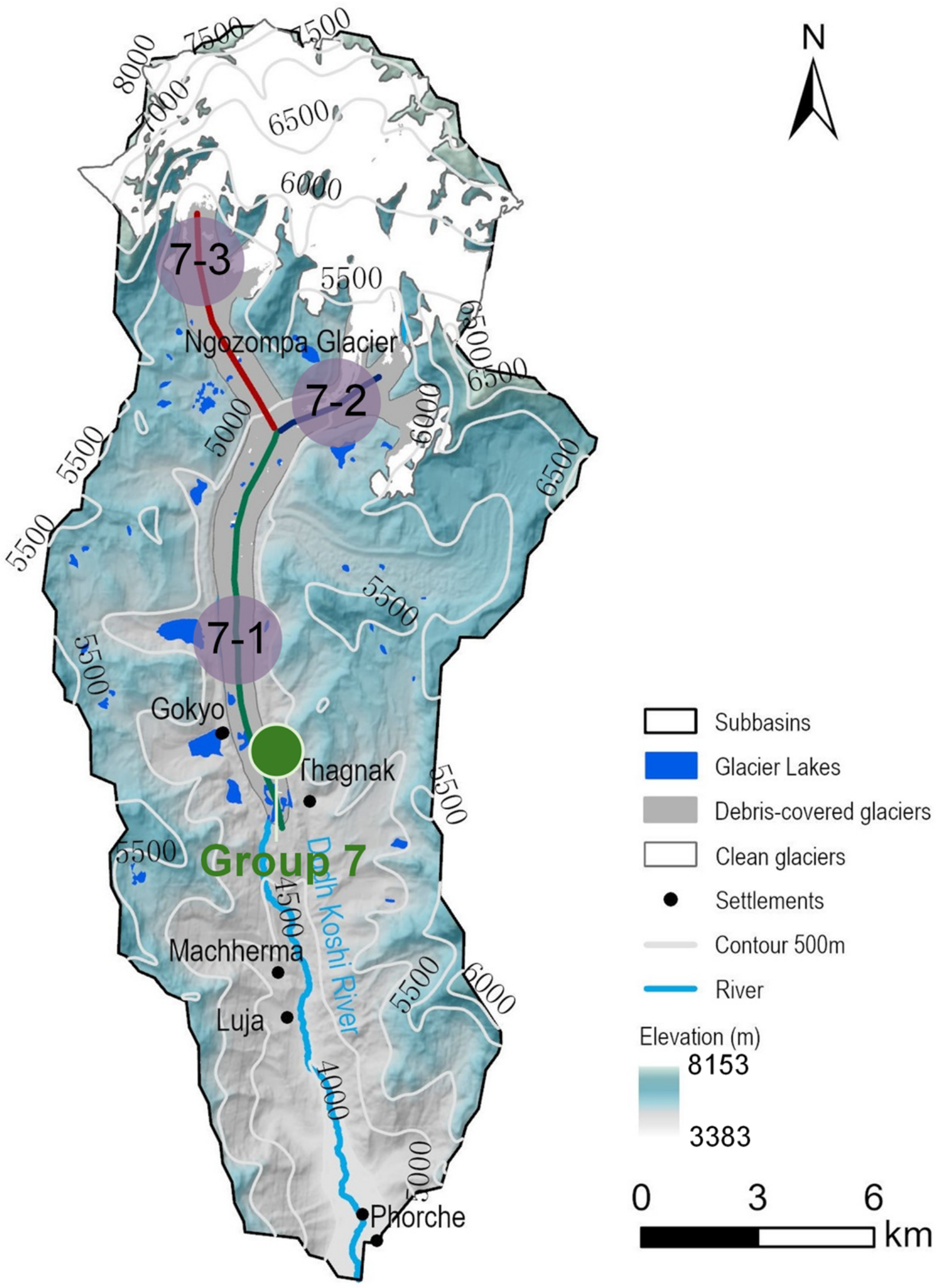

Fig. S3 | Criteria for glacier system classification and spatial distribution of glaciers. Group 7 represents the spatial distribution of the glacier group, and the number corresponds to the glacier branch in Fig. 2. The correspondence is as follows: **Group** 7 Ngozumpa Glacier (circled 7-1, 7-2 and 7-3).

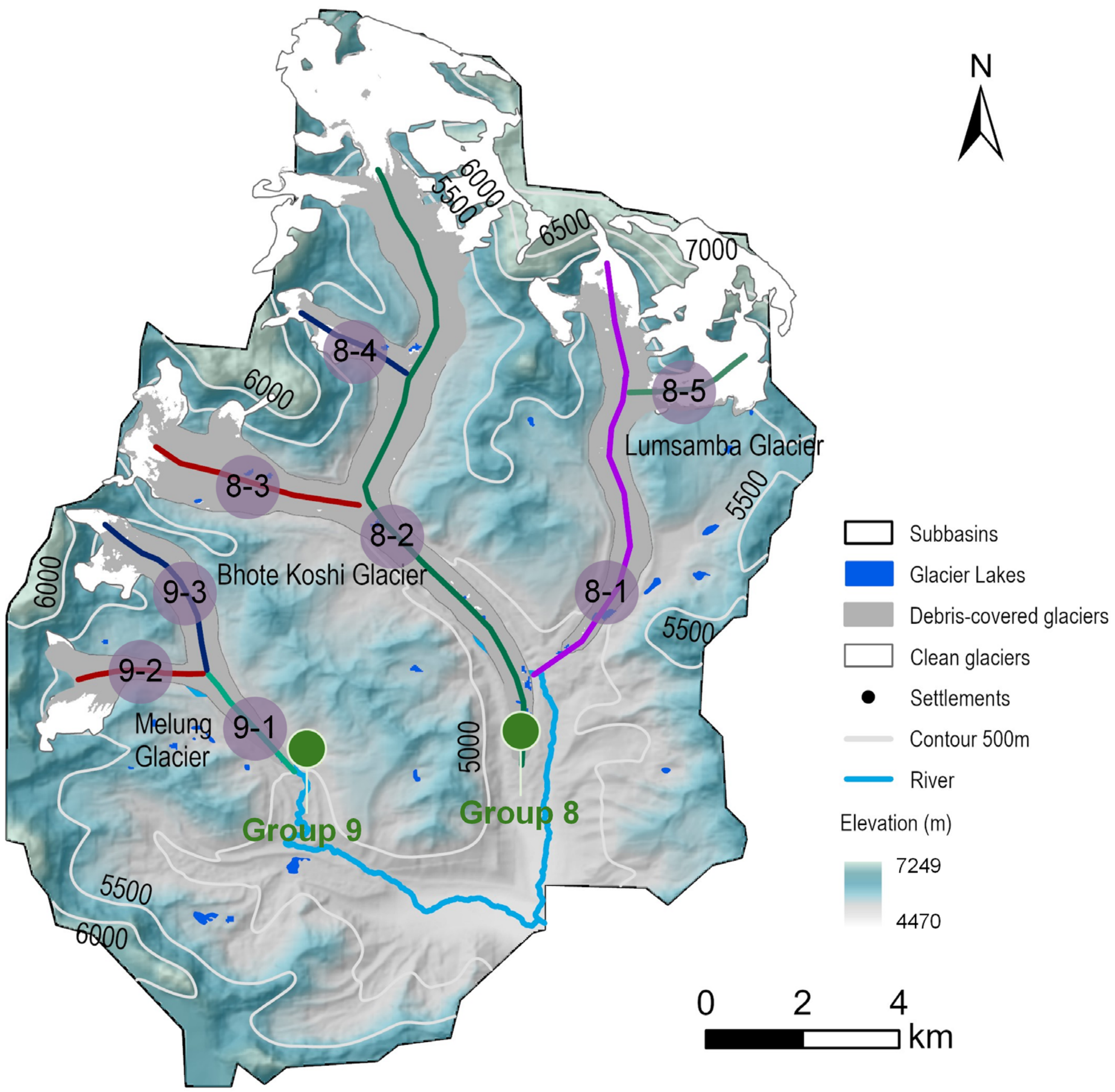

Fig. S4 | Criteria for glacier system classification and spatial distribution of glaciers. Group 8-9 represents the spatial distribution of the glacier group, and the number corresponds to the glacier branch in Fig. 2. The correspondence is as follows: **Group** 8 Bhote Koshi Glacier (circled 8-2, 8-3 and 8-4) and Lumsamba Glacier (circled 8-1 and 8-5). **Group** 9 Melung Glacier (circled 9-1, 9-2 and 9-3)

[Figure Redacted]

Comment 2.72: Figure 2: In both panels a and b, there is a yellowy-green polygon in the bottom right corner of the maps – what is this and what are the colours showing? This is not explained. Panels a-1 and b-1 are hard to see and would benefit from being made larger as separate panels below the maps. Panel a-1: what are the x axis units of this plot? The numbered purple circles are presumably individual glaciers but this is not explained anywhere - and these seem to have no connection to the numbered circles in Figure 1. Panel c: this shows "lake

centroids' migration distances and elevation gradients" according to the caption. By migration (which I am assuming is the same thing as 'displacement' mentioned elsewhere), is this the downglacier movement of the lake as the ice flows? If so, the arrow pointing upglacier "0-Max" is counterintuitive as it appear to imply upglacier movement. For the profiles in this panel, what do the different colours brown and grey-blue signify?

Response: Thank you for your comment. In panels A and B, the yellow-green polygon in the lower right corner of the map represents Imja Tsho. It is included because it has undergone artificial drainage and is the largest lake in this study; therefore, we specifically highlight it. We have added an explanation of this lake and details of the artificial drainage event in the figure caption.

Regarding the issue that panels a-1 and b-1 are difficult to see, we have followed your suggestion and separated them into independent subpanels (c and d). In subpanel c, we have also added information to the x-axis (showing the lifecycle and size distribution of lakes across glacier groups 1–9).

The purple circles here do not correspond to those in Figure 1 (the circles in Fig 1 represent the distribution and spatial locations of hazards, whereas the circles in Fig 2 represent the numbering of individual glacier branches). **To improve clarity, we have added Extended Data Table 1, which provides a detailed correspondence between glacier systems, individual glaciers, glacier branches, and their respective identifiers.**

Regarding "lake centroids' migration distances and elevation gradients," we calculated the elevation and slope before and after lake migration. Specifically, we analyzed the elevation gradient and migration distance between two points, rather than strictly following an upglacier–downglacier relationship. The arrow "0→Max" represents the magnitude of lake migration distance; it does not indicate an upglacier direction. We divided the migration distances into six classes and represented them using circles of different sizes to illustrate their spatial distribution and characteristics. In other words, "0→Max" indicates the progression from smaller to larger circle sizes. Our gradient results show that many lakes have elevation

gradients between 0 and 0.1. This suggests that lakes can erode the surrounding terrain and expand toward areas that were originally at higher elevations. This may seem counterintuitive, as lakes are generally expected to develop in depressions and lower terrain. However, observations from the development history of Imja Tsho clearly demonstrate this phenomenon: as the lake erodes toward higher elevations, it can trigger collapse at the glacier terminus, and areas that were originally at higher elevations become part of the expanding lake due to ice melt. This also explains why Imja Tsho exhibits greater depth near the glacier terminus *(references: Modeling the glacial lake outburst flood process chain in the Nepal Himalaya: reassessing Imja Tsho' s hazard, https://doi.org/10.5194/hess-22-3721-2018; Changes in Imja Tsho in the Mount Everest region of Nepal, 2014, https://doi.org/10.5194/tc-8-1661-2014)*. Since this study does not specifically investigate lake depth (we plan to address this in future work), we did not include corresponding data or figures, which may have made this part less clear. However, a conceptual figure in a previous study *(A conceptual model for glacial lake bathymetric distribution, https://doi.org/10.5194/tc-17-5137-2023, Fig. 9, As shown in the figure below)* illustrates this phenomenon well.

Finally, in the profile plots of this panel, the grey-blue color represents the elevation extracted along the glacier centerline, while the brown color represents the minimum elevation along the corresponding cross-section of the glacier centerline (This approach helps to illustrate the terrain where lakes are located and their potential development trends). Based on your comments, we have added clarifications to the figure caption *(For details, please see the revised manuscript: Fig. 2 (This has also been presented in this response))*.

We have revised the paragraph as follows:

Extended Data Table 1 | Correspondence between Glacier Systems, Glacier Names, Glacier Branches, and Lake Numbers in Each Glacier Group

Glacier System	Glacier Name	Glacier Branch (Fig.2)	Number of Lakes
Group 1	Ambulapcha	1-1	88
	Imja	1-2	
	Lhotse Shar	1-3	
Group 2	Lhotse	2	114
Group 3	Lhotse Nup	3	42
Group 4	Ama Dablam	4	42
Group 5	Nuptse	5	66
Group 6	Khumbu	6	144
Group 7	Ngozumpa	7-1	391
		7-2	
		7-3	
Group 8	Bhote Koshi	8-2	502
		8-3	
		8-4	
	Lumsamba	8-1	
		8-5	
Group 9	Melung	9-1	104
		9-2	
		9-3	

Glaciers sharing the same outlet were classified into the same group. The nine groups together contain 12 independent glaciers. Due to branching structures in some glaciers, a total of 19 glacier branch units were identified. The spatial correspondence between the identifiers and specific glacier branches is shown in Fig. 2a and Figs.S1–S4.

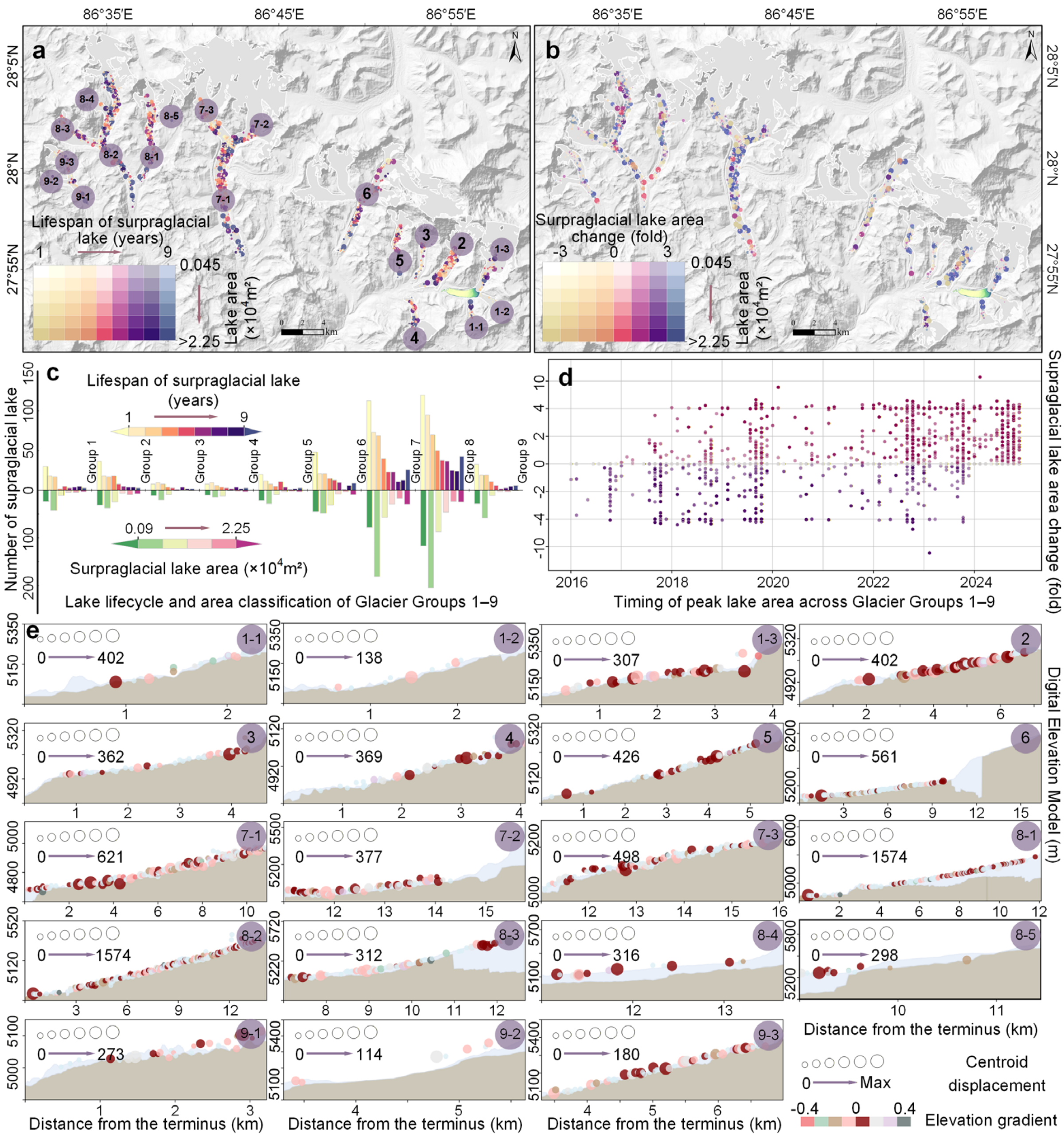

Fig.2 | Spatial distribution, temporal dynamics, and lifecycle characteristics of supraglacial lakes between 2016 and 2024. a Spatial patterns and lifecycles of SGLs of different sizes (2016–2024), due to the branching structure of some glaciers, each branch was assigned a unique identifier to facilitate analysis of the spatiotemporal distribution of lakes (Fig. S1-S4). The correspondence between glacier systems, glacier names, glacier branches, and the number of lakes in each group is provided in Extended Data Table 1. **b** Spatial patterns of lake peaks and peak-to-trough change rates. In panels **a** and **b**, circle size represents lake area, which is classified into six levels: of 0.045×10^4, 0.09×10^4, 0.36×10^4, 0.81×10^4, 1.14×10^4, 2.25×10^4,

and greater than 2.25×10^4 m², respectively. **c** Lifecycle, number, and size distribution characteristics of lakes in each glacier group **d** Timing of peak SGL area for expanding and shrinking lakes across nine glacier groups. **e** Lake centroids' migration distances and elevation gradients, with panel e labels matching glacier numbers in panel a. In panel **e**, circle size indicates lake migration distance, divided into six levels using equal intervals. In the profile plots of this panel e, the grey-blue color represents the elevation profile extracted along the glacier centerline, while the brown color represents the minimum elevation from the corresponding cross-sectional profiles. The green lake in panels a and b is Imja Tsho. To reduce the risk of its outburst, the Nepalese government, together with the United Nations Development Programme (UNDP) and the Nepal Army, constructed a drainage channel from June to September 2016. Over a cumulative period of two months, approximately 3.4 m of water was lowered, discharging about 4–5 million cubic meters.

[Figure Redacted]

Comment 2.73: Figure 3: What is the source of the meteorological data plotted for each glacier? Is this measured in the field at each site? I can't find mention of these datasets in the methods. I can't detect any difference between the shadings for pre-monsoon/monsoon and post monsoon/winter – I only see white or grey bands. What is showing on the y axes of these plots? This is not labelled. The x axes suggests the date format is YYYY-MM-DD but on the plots it is YYYY-M. Labelling the plots directly with the glacier names, and how these relate to the numbered glaciers in Figure 2, would be useful.

Response: Thank you for your comment. The meteorological data used in this study were mainly obtained from open-access datasets provided by monitoring stations. To help readers better understand the details of these datasets, we have added the relevant description in the Data Availability section *(For details, please see the revised manuscript: Lines 622-623 (This has also been presented in this response))*.

Regarding the differences in shading for the pre-monsoon, monsoon, post-monsoon, and winter periods, we sincerely apologize for the difficulty in distinguishing them due to our previous color scheme. We have now revised the color design to improve clarity. The y-axis in the figure represents the water surface area. Since both "Daily surface water" and "Monthly surface water" share the left y-axis, we have indicated the y-axis description in the figure legend. The x-axis represents dates in the format YYYY-MM-DD (Year- Month- Day), which has also been corrected in the figure *(For details, please see the revised manuscript: Fig. 3 (This has also been presented in this response))*.

Regarding the suggestion to directly label glacier names in the figures, we would like to clarify that some glacier names are relatively long, and repeatedly using full glacier names in the main text would make the manuscript harder to read. Therefore, we use glacier group numbers throughout the analysis in the text (e.g., "Glacier Group 1" instead of listing all individual glacier names such as Lhotse Shar, Imja, and Ambulapcha). In the figures, we also use a consistent labeling system with nine glacier groups (Group 1–9), while the specific glacier names are provided in the figure captions. This way, readers can refer to the captions when

needed to understand the corresponding glaciers, which helps keep the figures clearer and improves overall readability. *(For details, please see item Comment 2.6 in this Word response)*.

We have revised the paragraph as follows:

> ***Lines 622-623 IN THE REVISED MANUSCRIPT:*** *Meteorological monitoring data from stations near the glaciers are freely available at https://zenodo.org/records/15203896.*

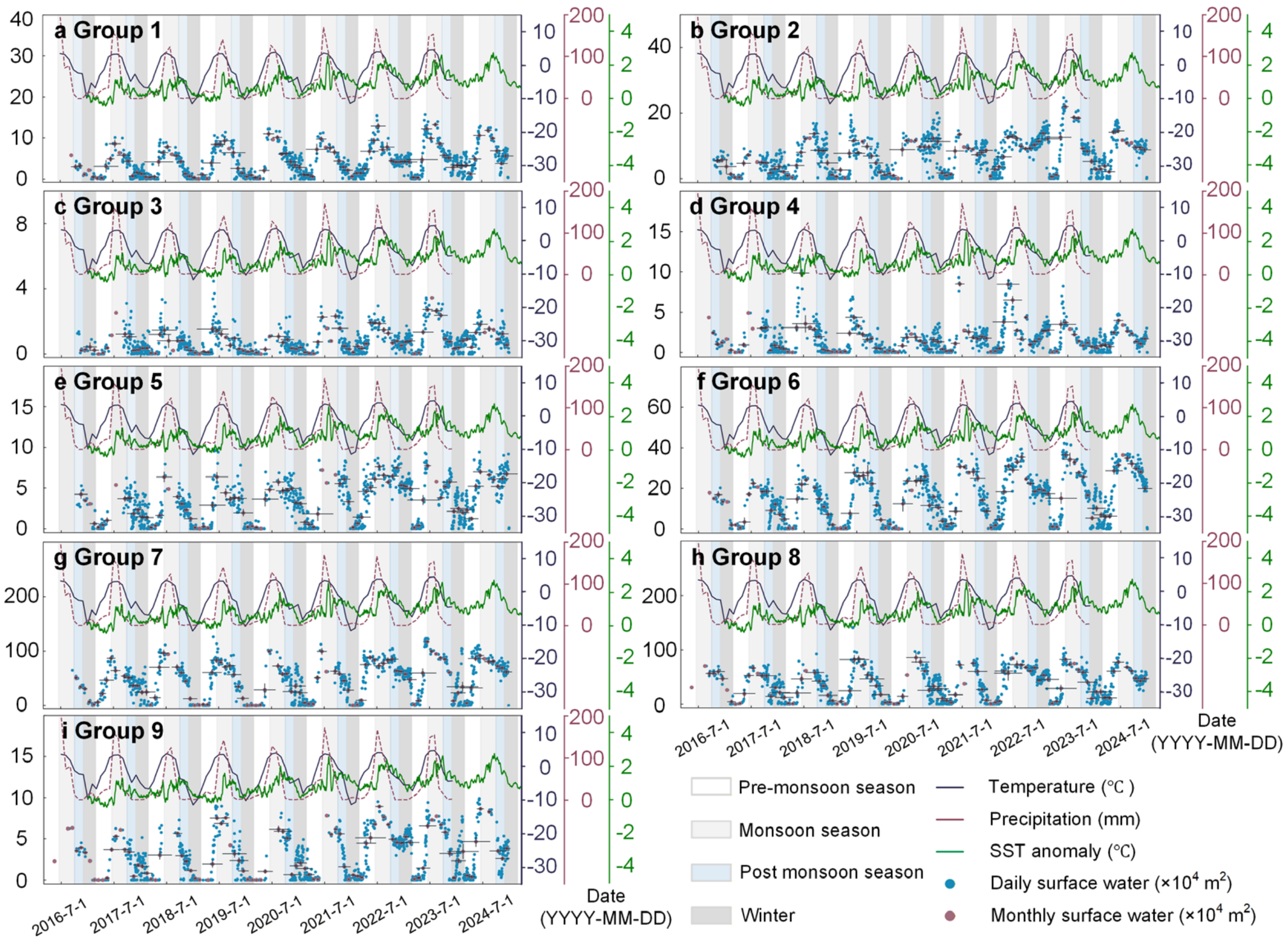

Fig. 3 | Highlight the time series of surface water resources, station temperature, precipitation, and Pacific Ocean temperature anomalies. Time series are shown for **Group 1** Lhotse Shar、Imja and Ambulapcha; **Group 2** Lhotse; **Group 3** Lhotse Nup; **Group 4** Ama Dablam; **Group 5** Nuptse; **Group 6** Khumbu; **Group 7** Ngozumpa; **Group 8** Bhote Koshi and Lumsamba and **Group 9** Melung Glaciers. Highlight SGLs in a–f were selected based on their stronger correlations with SST anomalies when leading by 1–2 months (correlation coefficients

of 0.72, 0.65, 0.64, 0.41, 0.68, 0.71, 0.63, 0.58, and 0.62, respectively). Pre-monsoon season (Mar 1–Jun 15), Monsoon season (Jun 15–Sep 30), Post-monsoon season (Oct 1–Nov 30), winter (Dec 1–Feb 28). The horizontal error bars in the scatter plot represent the normalized standard deviation, while the vertical error bars represent the standard error. They are used to measure the variability of monthly fluctuations in SGL area, and to examine day-scale differences of SGLs across different years in the long-term time series.

Comment 2.74: Figure 4: Panel a: What is the "Melung" label? Is this part of the overall glacier? This needs to be clarified (and might explain the confusion over glacier numbers elsewhere in the paper). The inclusion of the proglacial lake here indicates that this paper isn't only about supraglacial lakes, as the title seems to state. Glacier No. 9 here doesn't appear to be the same number 9 glacier on Figure 2 – is it? This could be confusing. Panel b: label the lakes here. What do the reported values here relate to? The caption does not explain this. Panels d and e: what are Lakes A, B, C and D on these diagrams? What are the curved blue arrows under the glacier signifying?

Response: Thank you for your comment. The entire glacier group is referred to as the Melung Glacier system. We acknowledge that our previous naming convention may have caused confusion. Therefore, we have redefined and standardized the classification scheme: when multiple upstream glaciers share a common drainage outlet, they are treated as a single glacier system and labeled as Group 1, Group 2, …, Group 9. This has also been clarified in our responses to other related comments ***(For details, please see the Comment 2.6, etc.)***.

Regarding the inclusion of proglacial lakes, our study primarily focuses on supraglacial lakes as well as proglacial lakes that have evolved from the expansion and coalescence of supraglacial lakes under glacier retreat. We recognize that the lack of clarity in the Introduction may have contributed to this confusion. Therefore, we have revised the second and third paragraphs of the Introduction to explicitly emphasize the connection between supraglacial and proglacial lakes.

As you correctly noted, “Glacier No. 9” here does not correspond to “9” in Figure 2. In Figure 2, the numbered units represent different glacier segments rather than entire glaciers, as some glaciers have branching structures. Therefore, each segment was assigned a number. To improve clarity, we have redefined the numbering scheme to better track lake dynamics across different glacier branches. **Each branch unit is assigned a group-consistent label, replacing the original 1–19 numbering system. For example, Group 9 consists of three branches, labeled Group 9-1, 9-2, and 9-3, whereas Group 2 has no branches and therefore remains as Group 2.** To clarify the correspondence between each label and glacier branches, we have added four figures in the Supplementary Information, which provide a detailed mapping between the numbering system and the glacier branches ***(For details, please see the Comment 2.6, 2.35 etc.)***.

In panel (b), we show the occurrence frequency and maximum area of the four largest supraglacial lakes across the entire glacier during the study period (2016–2024), these correspond to Lake 1, Lake 2, Lake 3, and Lake 4 in Figure 4-a. We have added explanations in the caption to clarify this point *(For details, please see the revised manuscript: Fig.4 (This has also been presented in this response))*.

In panels (d) and (e), A–D indicate the cascading sequence of upstream lakes transferring toward downstream areas. A represents the most upstream lake, B is the next upstream lake, and C, D, E, etc., are arranged sequentially downstream. The curved blue arrows at the bottom represent the process of subglacial meltwater transport.

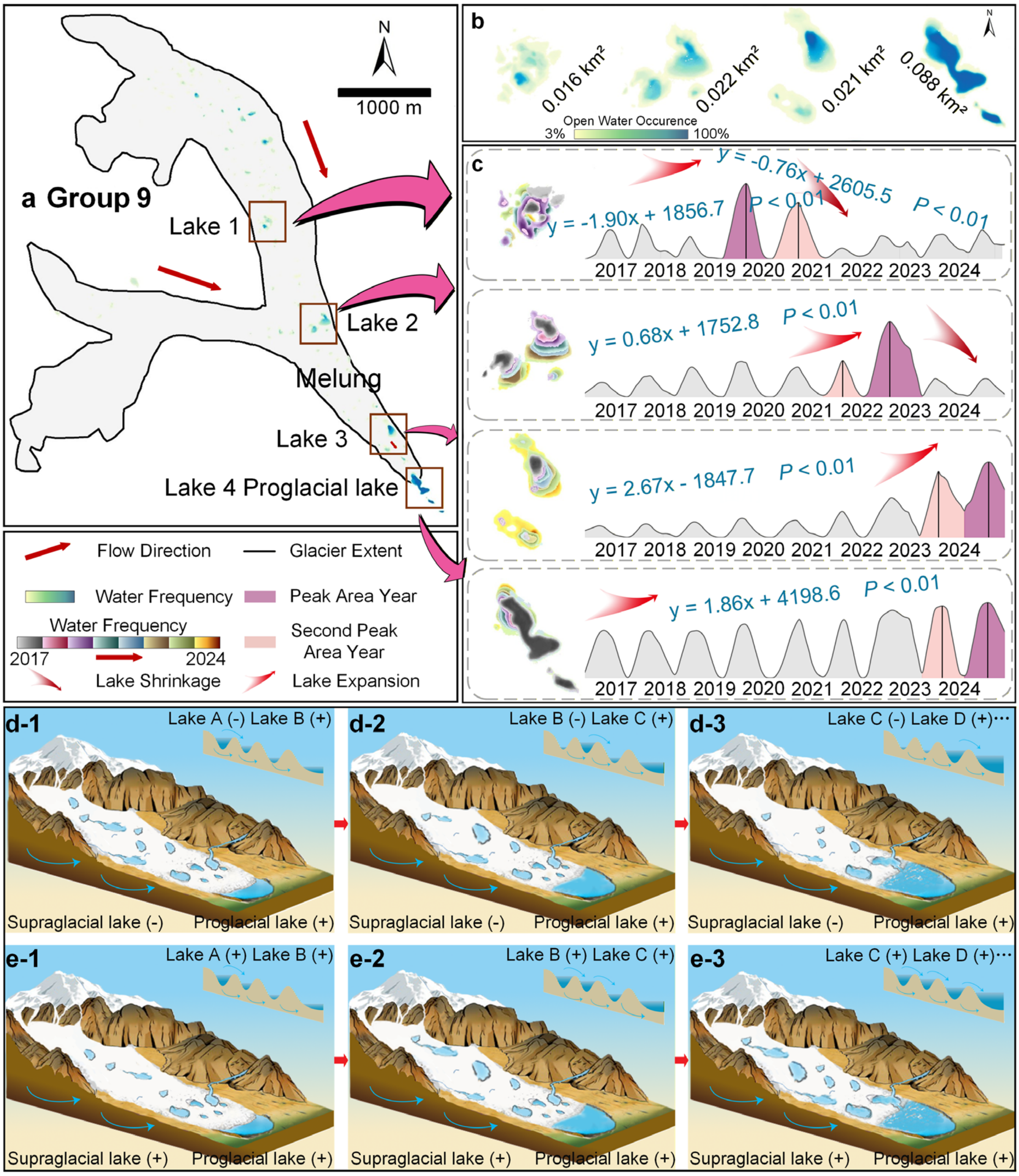

Fig.4 | Hydrological connectivity and cascading drainage among supraglacial lakes. a Frequency of lake occurrence in Glacier Group 9 from 2016 to 2024, with the four largest lakes highlighted (the four largest lakes). **b** Lakes are arranged from left to right corresponding to Lakes 1–4 in panel a, with the maximum area and occurrence frequency shown for each lake over the study period. **c** Annual frequency of SGLs and their cascading drainage processes, with locally weighted scatterplot smoothing fitted curves for lakes of interest. The x-axis of the ridgeline plot represents the time series of SGL area, with filled areas indicating the first and

second peaks of lake area. The frequency plots on the left use different gradient colors to show the annual distribution of lakes, illustrating upstream lake incision and downstream proglacial lake aggradation. **d** a pattern of downstream lake expansion accompanied by upstream lake decline. **e** a pattern of downstream lake expansion without upstream lake decline.

Comment 2.75: Figure 5: This seems to be the same plot repeated ten times. I am struggling to see any difference between the panels, or how these relate to the lakes that are labelled on each panel. It is hard to see the purple arrows "water vapor flux" or anything related to the "correlation between lake area and SST anomalies" colour scheme. The figure caption describes scatter plots but I cannot see these.

Response: Thank you very much for your comment. When we initially prepared the figure, we considered whether to include all panels or select representative ones. As you pointed out, displaying all datasets makes it difficult to clearly distinguish differences among panels, as well as to interpret details such as scatter plot colors and water vapor flux arrows. Based on your suggestion, we concluded that selecting representative glaciers for visualization is a better approach. Therefore, we have included only two representative groups in the main figure, while the remaining results have been moved to the supplementary materials. This ensures that all key information in the figure can be clearly interpreted. In addition, we have provided more detailed explanations in the figure caption to improve clarity *(For details, please see the revised manuscript: Fig. 5 (This has also been presented in this response))*.

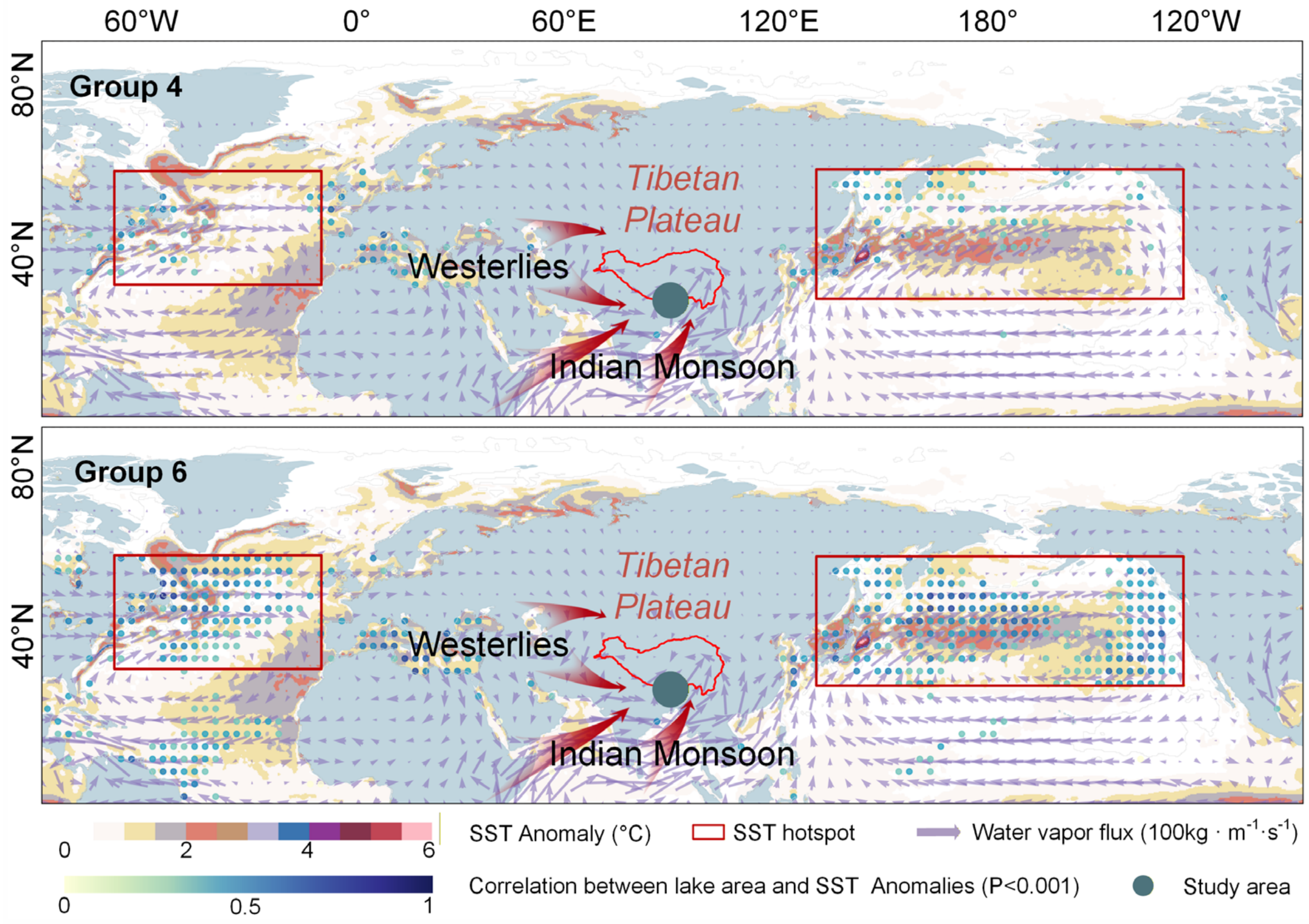

Fig. 5 | Teleconnections of supraglacial lakes area with monsoonal moisture transport and SST anomalies. Group 4 Ama Dablam Glacier (Few SGLs with weak catchment capacity); Group 6 Khumbu Glacier (Many SGLs with strong catchment capacity). Background map showing the spatial distribution of 2023 SST anomaly peaks. Purple arrows: moisture transport pathways; Red arrows: monsoon influence on HMA glaciers (Adapted from reference[21]). Scatter points indicate regions where seasonal fluctuations of lake area exhibit lagged correlations with SST anomalies.

Authors' responses to reviewers' comments

Unraveling daily dynamics of supraglacial lakes in response to hydrological pulses and anomalous climate signals

Jian Song, Haijun Qiu, Adam Emmer, Guoqing Zhang, Ninglian Wang, Ya Liu, Guoqing Yang, Yiwen Liang

Manuscript # NCOMMS-26-000007A

Note：

Response to the reviewer's comments (Reviewer 2): Please refer to Page 2-6.

Reviewer 1: All my comments in the last time have been fully addressed. Therefore, this manuscript is recommended to be accepted as its current version.

We sincerely thank you for the recommendation of acceptance!

Response to the reviewer's comments (Reviewer 2)

Reviewer 2: The author team should be commended on their clear and comprehensive response to reviewer comments. The major issues that were raised have been dealt with expertly. For example, the adjustment of the glacier naming/numbering means this is now much clearer to follow. Equally, the introduction is now much better focused on the central themes of the study, and the methods have been improved so that issues of clarity raised in later stages of the paper are now addressed. I have only a handful of minor comments on the figures, which I think could be improved further, but otherwise I am happy that this is ready for publication.

Response: We sincerely thank you for the recognition of our revision efforts. By revising the manuscript according to your comments, the quality of our manuscript has been significantly improved. We have also made corresponding revisions regarding the detailed comments you raised on some of the figures. Once again, thank you for your valuable comments on our work.

Further comments on figures

Comment 2.1: Figure 1 – I would add numbers to the study site circles on the map – perhaps in a different colour to differentiate from the numbered event circles. Better still, use a different numbered shape for the study sites, e.g. square or diamond.

Response: We sincerely thank you for your suggestion. According to your comments, we used diamond-shaped symbols to mark the study sites and labeled them with Roman numerals to avoid confusion with the numbers used for disaster events.

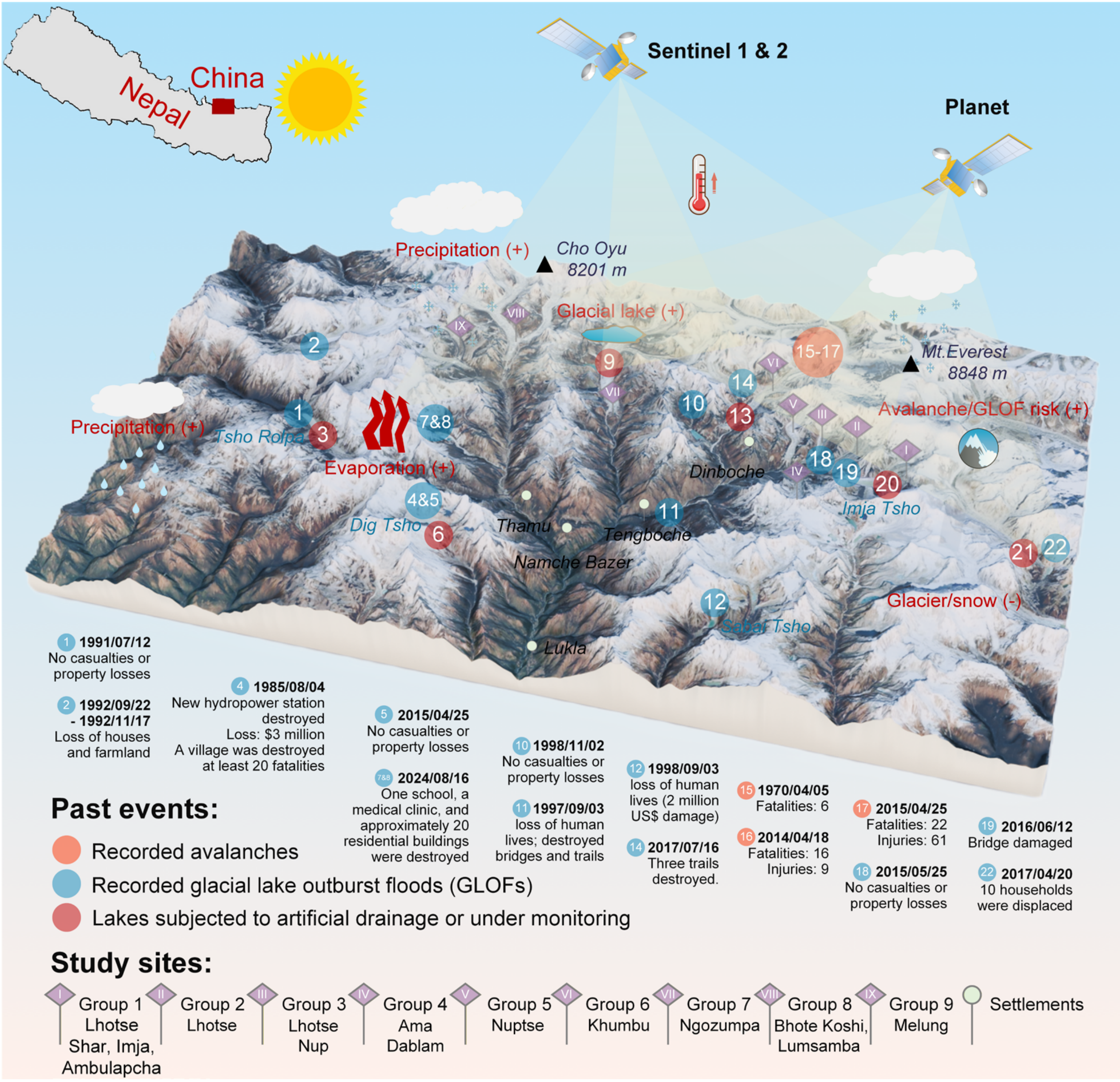

Comment 2.2: Figure 3 – technically the date format YYYY-MM-DD is not followed on the x axis – this would be, for example, 2016-07-01.

Response: Thank you very much for pointing out our problems. According to your comments, we have already made corrections.

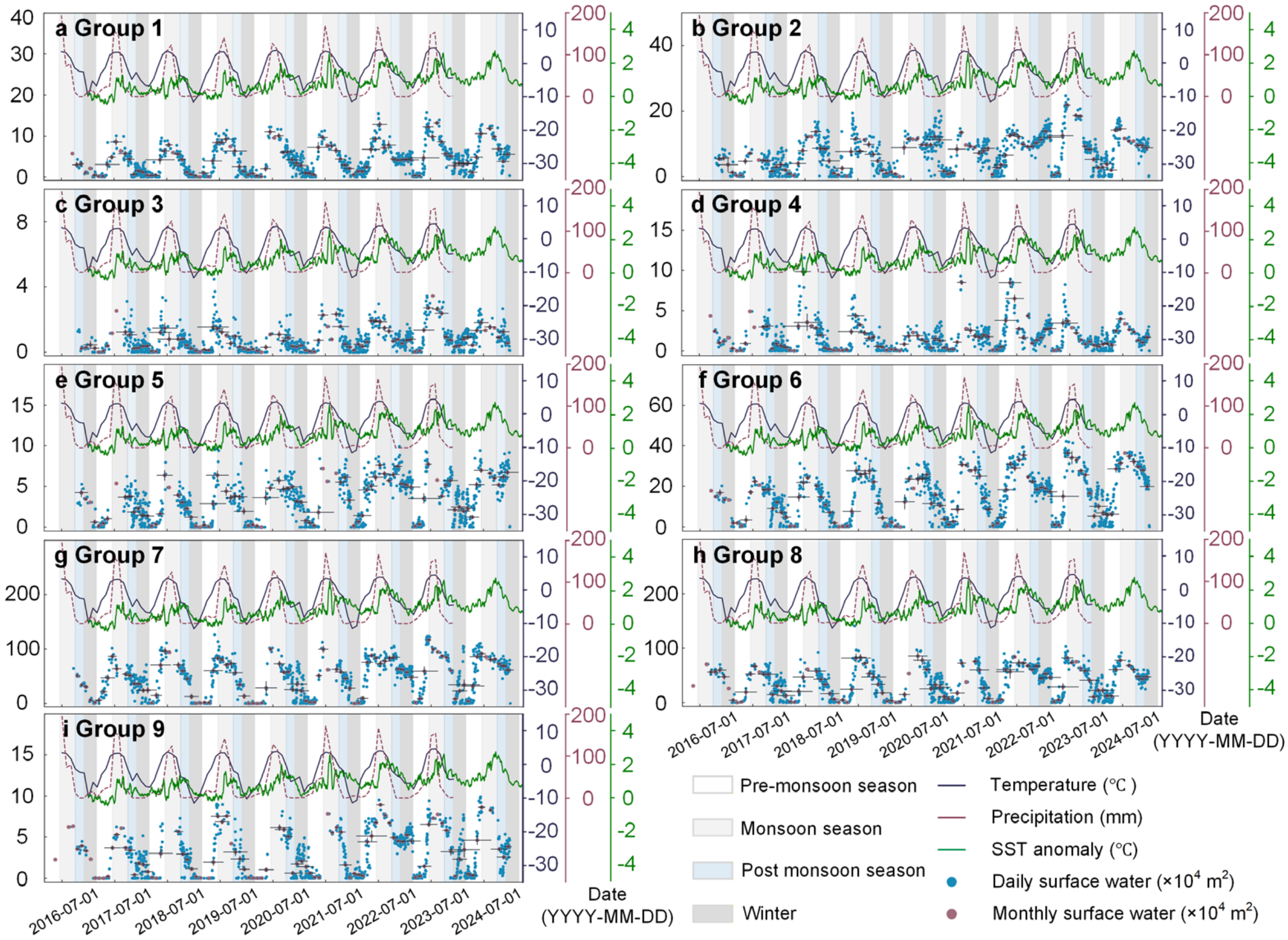

Comment 2.3: Figure 4, panels (d) and (e) – the blue line identified as subglacial meltwater transport remains confusing – this looks like groundwater flow on the diagram as it is within the substrate rather than at the ice-bed interface. I suggest some further improvements to make this clearer.

Response: Thank you very much for pointing this out. In response to your comment, we revised the cross-sectional profiles in panels (d) and (e). Since the glacier is currently undergoing degradation, we added three layers representing surface debris, ice layer, and bed. In addition, a schematic illustration of subglacial meltwater transport was included to provide a clearer and more accurate representation.

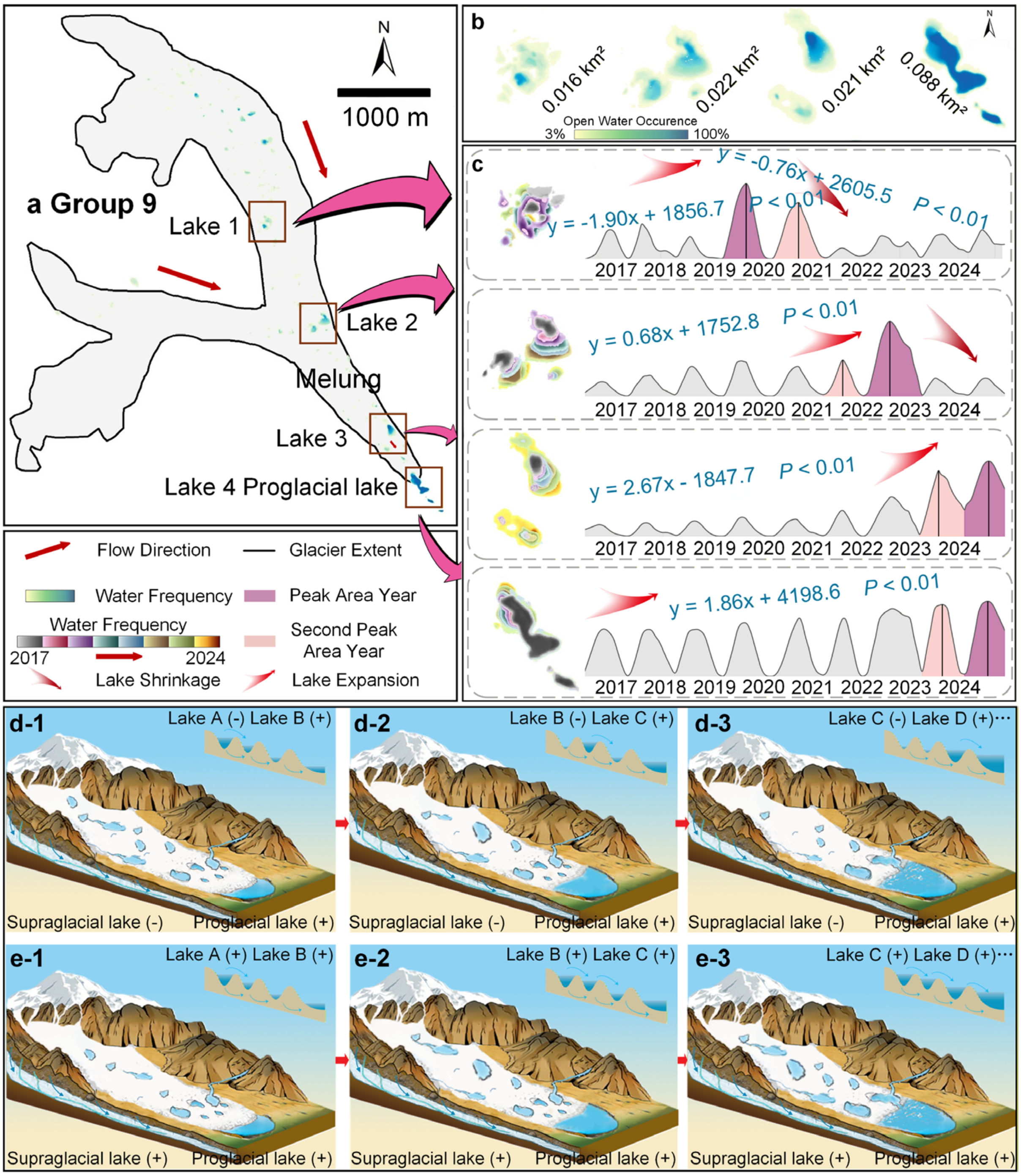

Comment 2.4: Extended Data Fig. 1 – the pin for Group 4 appears to be located in a subbasin without glaciers, and also overlays and partially obscures a glacier name. Suggest some minor adjustments to improve this. Perhaps moving the Group 4 label to immediately below the line extending from the circle would help, and moving the circle down slightly so I doesn't obscure the text.

Response: Thank you very much for pointing out the issue. In response to your suggestion, we have made the corrected revisions and also carefully checked the other markers to ensure their correct placement and to avoid any overlap or obstruction.

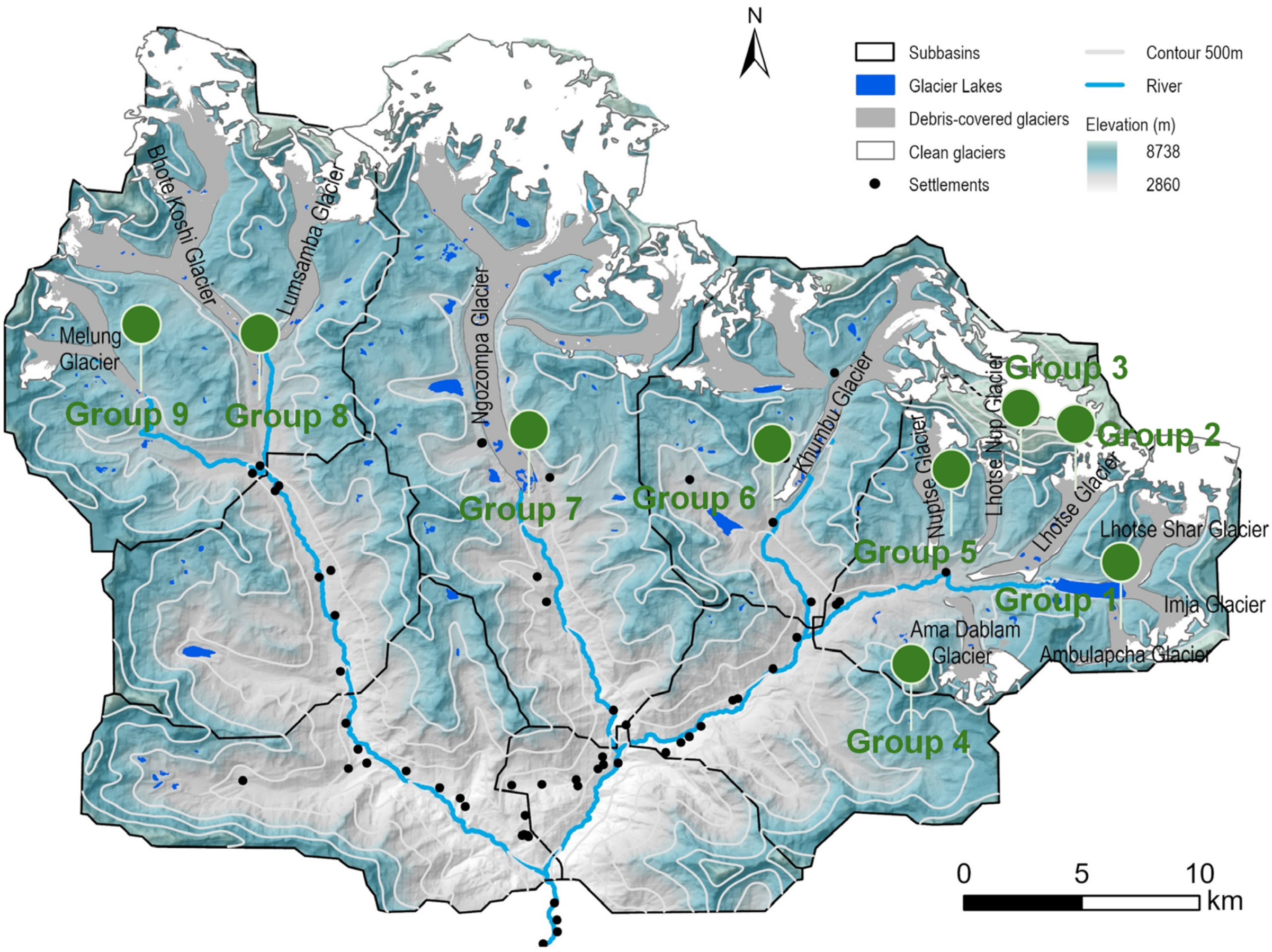

Supplement: Supplementary file 2 [file 41467_2026_75669_MOESM2_ESM.pdf]
