# Supplementary material for: Unraveling daily dynamics of supraglacial lakes in response to hydrological pulses and anomalous climate signals
Source: Nat Commun. 2026 Jul 17;17:7930. doi: 10.1038/s41467-026-75669-3 (PMC13448815; doi:10.1038/s41467-026-75669-3)
Supplement: Supplementary file 3 [file 41467_2026_75669_MOESM3_ESM.pdf]

nature portfolio

Corresponding author(s): Haijun Qiu

Last updated by author(s): Jun 3, 2026

Reporting Summary

Nature Portfolio wishes to improve the reproducibility of the work that we publish. This form provides structure for consistency and transparency in reporting. For further information on Nature Portfolio policies, see our Editorial Policies and the Editorial Policy Checklist.

Statistics

For all statistical analyses, confirm that the following items are present in the figure legend, table legend, main text, or Methods section.

n/a	Confirmed	
☐	☒	The exact sample size (*n*) for each experimental group/condition, given as a discrete number and unit of measurement
☐	☒	A statement on whether measurements were taken from distinct samples or whether the same sample was measured repeatedly
☒	☐	The statistical test(s) used AND whether they are one- or two-sided<br>*Only common tests should be described solely by name; describe more complex techniques in the Methods section.*
☒	☐	A description of all covariates tested
☒	☐	A description of any assumptions or corrections, such as tests of normality and adjustment for multiple comparisons
☐	☒	A full description of the statistical parameters including central tendency (e.g. means) or other basic estimates (e.g. regression coefficient) AND variation (e.g. standard deviation) or associated estimates of uncertainty (e.g. confidence intervals)
☒	☐	For null hypothesis testing, the test statistic (e.g. *F*, *t*, *r*) with confidence intervals, effect sizes, degrees of freedom and *P* value noted<br>*Give P values as exact values whenever suitable.*
☒	☐	For Bayesian analysis, information on the choice of priors and Markov chain Monte Carlo settings
☒	☐	For hierarchical and complex designs, identification of the appropriate level for tests and full reporting of outcomes
☒	☐	Estimates of effect sizes (e.g. Cohen's *d*, Pearson's *r*), indicating how they were calculated

Our web collection on statistics for biologists contains articles on many of the points above.

Software and code

Policy information about availability of computer code

Data collection	Data used in this study were obtained from publicly available datasets and online platforms, as follows: Copernicus Sentinel-1A/B & Sentine-2, available directly from the European Space Agency https://scihub.copernicus.eu/; Planet images are available under a basic license from the Education and Research programme from Planet Labs Inc. through the Planet Explorer https://www.planet.com/explorer/. The GLOF Database V4.1 is freely available at http://glofs.geoecology.uni-potsdam.de. The RGI V6.0 is freely available at https://www.glims.org/RGI/rgi60_dl.html. NOAA OI SST v2 data are available online at http://www.esrl.noaa.gov/psd/data/gridded/data.noaa.oisst.v2.html. Meteorological monitoring data from stations near the glaciers are freely available at https://zenodo.org/records/15203896. The specific humidity and wind speed data are freely available at https://downloads.psl.noaa.gov/Datasets/. The Copernicus DEM data are freely available at https://dataspace.copernicus.eu/. The ALOS AW3D30 DSM data are freely available at https://www.eorc.jaxa.jp/ALOS/en/dataset/aw3d30/.No custom software was used for data collection.
Data analysis	Data analysis were conducted using custom Python scripts, and the corresponding code has been deposited in a publicly accessible Zenodo repository (https://doi.org/10.5281/zenodo.19708433).

For manuscripts utilizing custom algorithms or software that are central to the research but not yet described in published literature, software must be made available to editors and reviewers. We strongly encourage code deposition in a community repository (e.g. GitHub). See the Nature Portfolio guidelines for submitting code & software for further information.

Data

Policy information about availability of data

All manuscripts must include a data availability statement. This statement should provide the following information, where applicable:

- Accession codes, unique identifiers, or web links for publicly available datasets
- A description of any restrictions on data availability
- For clinical datasets or third party data, please ensure that the statement adheres to our policy

Source data used in this study are available as follows: Copernicus Sentinel-1A/B & Sentine-2, available directly from the European Space Agency https://scihub.copernicus.eu/; Planet images are available under a basic license from the Education and Research programme from Planet Labs Inc. through the Planet Explorer https://www.planet.com/explorer/. The GLOF Database V4.1 is freely available at http://glofs.geoecology.uni-potsdam.de. The RGI V6.0 is freely available at https://www.glims.org/RGI/rgi60_dl.html. NOAA OI SST v2 data are available online at http://www.esrl.noaa.gov/psd/data/gridded/data.noaa.oisst.v2.html. Meteorological monitoring data from stations near the glaciers are freely available at https://zenodo.org/records/15203896. The specific humidity and wind speed data are freely available at https://downloads.psl.noaa.gov/Datasets/. The Copernicus DEM data are freely available at https://dataspace.copernicus.eu/. The ALOS AW3D30 DSM data are freely available at https://www.eorc.jaxa.jp/ALOS/en/dataset/aw3d30/. Source data are provided with this paper.

Research involving human participants, their data, or biological material

Policy information about studies with human participants or human data. See also policy information about sex, gender (identity/presentation), and sexual orientation and race, ethnicity and racism.

Reporting on sex and gender	none
Reporting on race, ethnicity, or other socially relevant groupings	none
Population characteristics	none
Recruitment	none
Ethics oversight	none

Note that full information on the approval of the study protocol must also be provided in the manuscript.

Field-specific reporting

Please select the one below that is the best fit for your research. If you are not sure, read the appropriate sections before making your selection.

☐ Life sciences ☐ Behavioural & social sciences ☒ Ecological, evolutionary & environmental sciences

For a reference copy of the document with all sections, see nature.com/documents/nr-reporting-summary-flat.pdf

Ecological, evolutionary & environmental sciences study design

All studies must disclose on these points even when the disclosure is negative.

Study description	This study quantifies the seasonal dynamics of supraglacial lakes using high-resolution remote sensing imagery, incorporating daily lake evolution into climate change feedback analysis. It focuses on the role of small supraglacial lakes in mountain hazards and their responses to climate warming.
Research sample	This study used 13,135 remote sensing images to construct the seasonal dynamics of supraglacial lakes across nine glacier sites. A large number of open-source datasets were also integrated for comprehensive analysis. The detailed data sources are documented in the Data Availability section.
Sampling strategy	Our analysis of sampling plots focused primarily on representative areas, such as the glacier termini of nine glacier systems, where proglacial lakes are more likely to form. These regions allow for clearer observation of the interactions between glacier flow velocity and lake development.
Data collection	Source data used in this study are available as follows: Copernicus Sentinel-1A/B & Sentine-2, available directly from the European Space Agency https://scihub.copernicus.eu/; Planet images are available under a basic license from the Education and Research programme from Planet Labs Inc. through the Planet Explorer https://www.planet.com/explorer/. The GLOF Database V4.1 is freely available at http://glofs.geoecology.uni-potsdam.de. The RGI V6.0 is freely available at https://www.glims.org/RGI/rgi60_dl.html. NOAA OI SST v2 data are available online at http://www.esrl.noaa.gov/psd/data/gridded/data.noaa.oisst.v2.html. Meteorological monitoring data from stations near the glaciers are freely available at https://zenodo.org/records/15203896. The specific humidity

	and wind speed data are freely available at https://downloads.psl.noaa.gov/Datasets/. The Copernicus DEM data are freely available at https://dataspace.copernicus.eu/. The ALOS AW3D30 DSM data are freely available at https://www.eorc.jaxa.jp/ALOS/en/dataset/aw3d30/. Source data are provided with this paper.
Timing and spatial scale	The dataset spans 2016–2024 with a daily temporal resolution and spatial resolutions of 3 m and 10 m, covering the upstream areas of nine glaciers in Sagarmatha National Park.
Data exclusions	Data affected by extensive cloud cover and NoData areas were excluded.
Reproducibility	The analyses in this study are reproducible using the methods described in this manuscript and the Supplementary Information, based on both publicly available datasets and our open-source datasets.
Randomization	Not applicable
Blinding	Not applicable

Did the study involve field work? ☐ Yes ☒ No

Reporting for specific materials, systems and methods

We require information from authors about some types of materials, experimental systems and methods used in many studies. Here, indicate whether each material, system or method listed is relevant to your study. If you are not sure if a list item applies to your research, read the appropriate section before selecting a response.

Materials & experimental systems

n/a	Involved in the study
☒	☐ Antibodies
☒	☐ Eukaryotic cell lines
☒	☐ Palaeontology and archaeology
☒	☐ Animals and other organisms
☒	☐ Clinical data
☒	☐ Dual use research of concern
☒	☐ Plants

Methods

n/a	Involved in the study
☒	☐ ChIP-seq
☒	☐ Flow cytometry
☒	☐ MRI-based neuroimaging

Plants

Seed stocks	none
Novel plant genotypes	none
Authentication	none
